# Time-series analysis reveals genetic responses to intensive management of razorback sucker (*Xyrauchen texanus*)

**DOI:** 10.1111/eva.12125

**Published:** 2013-11-15

**Authors:** Thomas E Dowling, Thomas F Turner, Evan W Carson, Melody J Saltzgiver, Deborah Adams, Brian Kesner, Paul C Marsh

**Affiliations:** 1 School of Life Sciences, Arizona State UniversityTempe, AZ, USA; 2 Department of Biology and Museum of Southwestern Biology, University of New MexicoAlbuquerque, NM, USA; 3 Marsh & AssociatesTempe, AZ, USA

**Keywords:** age structure, census size, effective number of breeders, genetic diversity, genetic monitoring

## Abstract

Time-series analysis is used widely in ecology to study complex phenomena and may have considerable potential to clarify relationships of genetic and demographic processes in natural and exploited populations. We explored the utility of this approach to evaluate population responses to management in razorback sucker, a long-lived and fecund, but declining freshwater fish species. A core population in Lake Mohave (Arizona-Nevada, USA) has experienced no natural recruitment for decades and is maintained by harvesting naturally produced larvae from the lake, rearing them in protective custody, and repatriating them at sizes less vulnerable to predation. Analyses of mtDNA and 15 microsatellites characterized for sequential larval cohorts collected over a 15-year time series revealed no changes in geographic structuring but indicated significant increase in mtDNA diversity for the entire population over time. Likewise, ratios of annual effective breeders to annual census size (*N*_b_*/N*_a_) increased significantly despite sevenfold reduction of *N*_a_. These results indicated that conservation actions diminished near-term extinction risk due to genetic factors and should now focus on increasing numbers of fish in Lake Mohave to ameliorate longer-term risks. More generally, time-series analysis permitted robust testing of trends in genetic diversity, despite low precision of some metrics.

## Introduction

The value of time-series analysis of long-term datasets is well appreciated in ecology (Lindenmayer et al. 2012). The recently introduced and now widely adopted ‘genetic monitoring’ approach (Schwartz et al. 2007) promises to increase accumulation, taxonomic breadth, and depth of genetic time series as applied to evolutionary investigations of wild or managed species. In ecology, time-series and long-term datasets have provided powerful insights into acute and chronic responses to disturbance, processes with high temporal variability, and complex phenomena (Franklin 1989). Genetic monitoring could provide comparable insight into complex relationships of ecological, demographic, and genetic factors (e.g., Schwartz et al. 2007; Antao et al. 2011; Osborne et al. 2012), and longer time series could be particularly valuable for study of species with long generation times, high inter-annual variability in reproductive success, and high reproductive potential (e.g., bony fishes). This is because species with these characteristics have broad capacity for differential response to fluctuating environmental conditions, whether natural (e.g., climate cycles or wildfire) or human-mediated (e.g., harvesting, habitat alteration, climate change).

Genetic monitoring (Schwartz et al. 2007) consists of sampling a population at regular intervals and estimating metrics (i.e., heterozygosity, relatedness, allelic richness, effective population size) via standardized analysis that permits meaningful characterization of change in populations over time. For an actively managed population, there are essentially three temporal trends of interest, namely metrics remain unchanged, they decrease, or they increase over time. If unchanged, we may conclude that demographic features that determine genetic diversity are stable and consistent through time. Decreasing metrics are indicators of lowered abundance or the action of other demographic factors that may indicate recruitment failure or unexpected mortality that has decreased levels of genetic diversity. If metrics are increasing, we may conclude that demographic limitations that previously impinged upon the population have been ameliorated over time, perhaps as a result of management action.

Of metrics commonly evaluated in genetic monitoring, genetic effective population size (*N*_e_) and the annual effective number of breeders (*N*_b_) are arguably most useful because they determine, in part, the evolutionary capacity of a population to respond to novel or fluctuating environmental conditions (e.g., Newman and Pilson 1997). Moreover, simulation study shows that *N*_b_ and *N*_e_ are sensitive indicators of population decline (Antao et al. 2011). However, it is the relationship of the effective size (*N*_b_ or *N*_e_) to census size (*N*), or *per capita* effective size, that explicitly links evolutionary and demographic processes (Frankham 1995). This is because features intrinsic to particular species (i.e., life history and mating system) affect *N*_b_*/N* or *N*_e_*/N*, as do extrinsic features such as habitat alteration (e.g., Turner et al. 2006) and overharvesting (e.g., Hauser et al. 2002). Thus, evaluation of *N*_b_*/N* or *N*_e_*/N* over a time series could help identify specific factors that limit genetic diversity, particularly in exploited and managed species.

Sequential estimation of *N*_b_ or *N*_e_ and *N* is rarely done for any species and estimates of *N*_b_, *N*_e_, and *N* can have large variances (Palstra and Fraser 2012). Consequently, it is not known whether a time-series incorporating *N*_b_*/N* will be generally useful for monitoring population responses to management action. In this paper, we explored the utility of this approach through a case study of a long-lived and fecund, yet endangered freshwater fish.

### Case study

Desert rivers of the southwestern United States provide the backdrop for this study. Many of the more than 70 fish species native to the region are in steep decline and essentially all are imperiled (Minckley and Marsh 2009). Fishes of the mainstem Colorado River have been especially impacted. The environmental and geological dynamics of the Colorado River basin have led to one of the higher levels of endemism and lower species diversities of any North American river (Smith et al. 2010). This fauna has recently been severely affected by anthropogenic disturbances (Minckley and Marsh 2009), which have dramatically altered community structure. Six of 10 species native to the mainstem Colorado River are depleted and listed as endangered, including the four ‘big-river’ fishes (humpback chub, *Gila cypha*; bonytail, *G. elegans*; Colorado pikeminnow*, Ptychocheilus lucius*; razorback sucker, *Xyrauchen texanus*) – large, long-lived, endemics that once were abundant and broadly distributed throughout the basin (Minckley et al. 2003). These species are now the target of considerable conservation action (Wydoski and Hamill 1991; Lower Colorado River Multi-Species Conservation Program 2004), making them excellent systems for study of the impact of these actions on genetic variation and demography of endangered fishes.

Our focus is on one of the four ‘big-river’ fishes, the razorback sucker. This species is a large [>750 mm total length (TL)], long-lived (>50 years) member of the family Catostomidae. The biology of this species has been extensively studied (reviewed by Minckley and Marsh 2009). Males typically achieve sexual maturity at 2–3 years, whereas females generally become reproductive at ages 3–5. Females can be extremely fecund, with some larger individuals capable of producing more than 100 000 ova per year. Telemetry studies have shown individual movement of 10s to >100 km, including movement to multiple spawning areas within a season (Tyus and Karp 1990; Mueller et al. 2000; Karam et al. 2008). While razorback sucker was once abundant in large rivers and associated floodplains throughout the Colorado River drainage, human-induced impacts (especially the introduction of non-native fishes that prey on larvae, juveniles, and adults) have resulted in dramatic reduction in numbers of individuals throughout the basin (Dowling et al. 1996a; Marsh et al. 2003; Minckley and Marsh 2009), which ultimately led to listing this species as endangered (U.S. Fish and Wildlife Service [USFWS] 1991).

Unlike other remnant subpopulations, the one in Lake Mohave, a main stream reservoir in Arizona and Nevada, remained large (>60 000 individuals) into the late 1980s and thus became a focal point for study and conservation action (Minckley 1983; Minckley et al. 1991). In the early 1990s, managers initiated a strategy that deviated from the traditional hatchery-based approach of captive propagation but instead utilized naturally produced larvae that were captured in Lake Mohave, reared in protective custody, and released back into the lake as they achieved a size presumed sufficient to escape predation (Mueller 1995; Minckley et al. 2003). This approach was selected to maximize the number of individuals contributing larvae, presumably maintaining genetic variation for future generations. Larval-to-juvenile survivorship in the wild is nil, and natural recruitment has been undetected since the 1950s (Minckley 1983; Minckley et al. 1991). Survivorship in captivity of wild-produced larvae was relatively low during the early years of the program but has improved substantially as rearing methods were revised and refined, new artificial foods were developed, and infrastructure was modified to provide better conditions; survivorship now typically exceeds 90%. Mortality during the posthatchery grow-out phase is variable due to stochastic environmental factors, such as disease, predation (both avian and piscine), environmental extremes, etc., and likely is typical of other fish species under similar conditions (e.g., Stroud 1986); post-stocking mortality in the lake is driven by natural factors exacerbated by the presence of large-bodied, non-native striped bass (*Morone saxatilis*) that prey on adult razorback sucker of all sizes (Marsh et al. 2003; Karam et al. 2008).

During the 20-year period through 2011, more than 750 000 larvae have been harvested, and more than 155 000 fish have been stocked into Lake Mohave. Most early stockings were of relatively small individuals that were apparently lost to predation (Marsh et al. 2005). In recent years, size of stocked fish has been increased, but this has only marginally improved long-term survival (Kesner et al. 2008). Given natural recruitment failure and limited success of the stocking program, the population has dwindled to approximately 3000 individuals (Pacey and Marsh 2011).

Genetic implications were considered critically important in design and implementation of the Lake Mohave repatriation program (Mueller 1995; Dowling et al. 1996a,b1996b) but not of programs elsewhere in the lower Colorado River basin, where hatchery-produced fish have been used to support extensive but unsuccessful stocking (Marsh and Brooks 1989; Minckley et al. 1991; Jahrke and Clark 1999). High reproductive capability of the species is a positive attribute because large numbers of individuals for repatriation can be rapidly produced using artificial means, and relevant hatchery methods and protocols have long been worked out (Inslee 1982; Hamman 1985). This positive characteristic, however, also has a potentially significant negative aspect. Because of the high reproductive output of single individuals, large numbers of repatriates can derive from a small number of parents (Hedgecock 1994). This could result in contribution of a small and/or nonrepresentative parental population, leading to reduced genetic variability and effective population size, and ultimately increased risk of extinction (Ryman and Laikre 1991). A significant impact of reduced genetic variation and viability has been documented for razorback sucker (Dowling et al. 1996b), making consideration of genetic concerns particularly critical.

Our study was designed to monitor impacts of the Lake Mohave razorback sucker conservation program. This was achieved by continuing analyses of mtDNA (Dowling et al. 2005) and adding characterization of microsatellite loci from samples of larvae that spanned the temporal and geographic breadth of the spawning population over a 15-year period. Because census size, genetic diversity, and genetic effective size are positively related in a Wright–Fisher idealized population (reviewed in Frankham et al. 2010), we predicted that genetic metrics should track census size and thus decline over the time series. The rate of decline could be great because of the possibility of large variance in reproductive success in species like razorback sucker (Hedgecock 1994), or because of nonrandom sampling of larvae for rearing in protective custody. This continuous time series is among the longest available for any fish species (Palstra and Fraser 2012) and is also one of the few to include relatively precise estimates of annual census size and genetic diversity (see Duong et al. 2013 for another recent example), thus making it an excellent model system for understanding how demographic changes affect genetic variation in long-lived, iteroparous species.

## Materials and methods

### Sampling for molecular analyses

Dowling et al. (2005) observed 28 haplotypes among 2423 larvae that were characterized in 99 collections from 1997–2003. An additional 160 collections were made in Lake Mohave from 2004–2011, represented by a total of 3913 wild-caught larvae. Therefore, a total of 259 collections (*N* = 6336 wild-caught larvae) were made from 1997–2011, representing four major regions in Lake Mohave (Nine Mile Area, Tequila Cove, Wrong Cove, and Yuma Cove) where razorback sucker aggregate to spawn (Dowling et al. 2005). Larval samples were obtained between late January and late April, with each of these regions represented by an average of 4.6, 4.7, 2.9, and 5.1 collections/year, respectively. Whole larvae were immersed in 95% ethanol in individual snap cap tubes, and genomic DNA was extracted as described by Tibbets and Dowling (1996).

All individuals were characterized for variation in mtDNA, allowing us to extend the previous study (Dowling et al. 2005) and to test for temporal and regional differences within years as well as comparison of levels of variation over time. Because of the expense to analyze such a large number of samples, 120 individuals were randomly selected each year for characterization of microsatellite variation, using the randomization function in excel, yielding a total of 1800 individuals for 15 years. This method allowed us to examine levels of microsatellite variation over time. Because random subsamples from individual temporal collections were typically small (<10 individuals), it was not possible to examine temporal and regional differences within years for microsatellites.

### Characterization of mtDNA variation

Mitochondrial DNA variation was characterized through analysis of single-stranded conformational polymorphisms (SSCPs) in a 311 bp fragment from the more rapidly evolving 3′ end of cytochrome *b*, as described by Dowling et al. (2005). Briefly, each sample was run under two different sets of electrophoretic conditions, with standard samples (the four most common haplotypes) included on every gel. At least one representative of each mobility variant was sequenced from each pair of gels, and the haplotype for each group of mobility variants was determined by comparison with all known haplotypes by parsimony analysis using PAUP*. New haplotypes were designated alphabetically in order of discovery, not mobility similarity, and added to the template file for future analyses.

Gene diversity and number of mtDNA haplotypes were obtained using the program Arlequin 3.5.1.2 (Excoffier and Lischer 2010). Significance of deviation in the number of haplotypes and haplotype diversity for each sample was examined by comparison with expected values derived from a baseline sample of wild adults collected in Lake Mohave, using a bootstrap resampling program written in fortran (Dowling et al. 2005). This program generated null distributions of the appropriate size (e.g., matching the size of each sample examined here and thus correcting for sample size) by randomly resampling haplotypes (with replacement) from a source population followed by counting haplotypes and calculating haplotype diversity for each replicate. The source was represented by a sample of 272 individuals that were among remnants of the large wild population that persisted into the early 2000s (see Marsh et al. 2003; Dowling et al. 2005). Confidence intervals (CIs) were based on distributions of these two parameters for 10 000 of these replicates, and observed values were considered significant if they fell within the tails of these distributions (defined as the extreme 250 values on each side of the distribution, i.e., *P *<* *0.05).

The number of haplotypes was corrected for sample size (*H*_R_) by rarefaction (HP-Rare, Kalinowski 2005), with all samples smaller than 20 individuals excluded from the analysis. Because this is a haploid marker, one allele was coded as missing (‘00’) as recommended by the author (Kalinowski, *personal communication*). Variation in *H*_R_ among locations was tested by anova (IBM spss Statistics for Windows, version 20, Armonk, NY, USA). Arlequin also was used to examine the distribution of genetic variation within and among samples (periods and regions) by molecular analysis of variance (amova; Excoffier et al. 1992), allowing us to partition population level variance (*F*_ST_) among geographic regions (*F*_CT_) and among temporal samples within regions (*F*_SC_) for each year. Change in levels of variation over time was examined by ordinary least-squares regression (Microsoft excel), using individual sample estimates of *H*_R_ and haplotype diversity.

### Characterization of microsatellite variation

Fifteen dinucleotide microsatellite loci were used to characterize variation in the nuclear genome (Turner et al. 2009; Dowling et al. 2012a,b2012b). One primer of each pair was labeled with an infrared dye (with absorption at wavelengths of 700 nm or 800 nm) that allowed for the visualization of PCR products. Fragments were amplified in multiplexed reactions (as many as four sets of primers/reaction), using an Eppendorf Mastercycler thermal cycler. Fragment analysis was performed using a Li-Cor 4300 DNA Analyzer. Four lanes of sizing standard [50–350 bp in length and labeled with appropriate infrared dye (purchased from Li-Cor)] were included on each run and each gel was scored using the computer software saga (version 3.3; Li-Cor, Lincoln, NE, USA). saga assesses fragment allele sizes, as based on the sizing standards and provides the genotype for each individual. The software was used to manually compare results from multiple gels at the same time to insure consistency of allele assignment across gels.

Deviations from Hardy–Weinberg equilibrium (HWE; *F*_IS_), average heterozygosity per locus (h), and multilocus equilibrium were tested using fstat version 2.9.3.2 (Goudet 2001). Significance values from single-locus tests of HWE were adjusted using the B-Y correction as described by Narum (2006) (adjusted critical value of 0.0083). Allelic richness (*A*_R_) was calculated by rarefaction via the program HP-Rare as described by Kalinowski (2005). Variation in annual estimates of *A*_R_ and gene diversity over time was tested by anova (spss, version 20) and by regression (excel). Distribution of genetic variation within and among years followed Weir and Cockerham (1984) *F*-statistics as implemented by fstat. Significance of values was tested by jackknifing (over individuals and all loci) and bootstrapping (loci only).

### Annual census estimates

Annual census size (*N*_a_) for razorback sucker in Lake Mohave, defined as the number of reproductively capable adult individuals (fish that have been at large for at least 1 year postrelease and thus have grown to sexual maturity) was evaluated using the single census modified Peterson formula for closed populations (Ricker 1975). Separate estimates for wild and repatriate razorback sucker are calculated based on PIT (passive integrated transponder) tag encounter data collected during annual monitoring in March each year. All repatriate fish are PIT tagged prior to release and all wild fish have been tagged upon capture since 1991. Average repatriate size at stocking was increased from about 27 cm TL in 1997 to 37 cm in 2012 (Marsh et al. 2005; C. A. Pacey, unpublished data) as new information about predation vulnerability became available (Marsh et al. 2003, 2005; Karam and Marsh 2010); the current recommended target length is 50 cm (Pacey and Marsh 2007). Once annual monitoring data are verified and entered, the list of PIT tags encountered is compared with the previous year's sample to calculate the estimate. Repatriates released between the mark and capture years are removed from the estimate calculation for the repatriate population, and the separate estimates for wild and repatriate fish are combined to assess the entire population. Tag loss can bias mark-recapture population estimates (Ricker 1975), but year-to-year loss for this study was considered negligible (CA Pacey unpublished data; Ward and David 2006; Zelasko et al. 2011).

Calculations for repatriate population estimates used specifically for genetic analysis were adjusted to account for the sexual immaturity of most razorback sucker at the time of repatriation into Lake Mohave (i.e., not expressing gametes). The standard estimate of the repatriate population includes these potentially immature fish as long as they were released prior to March of the marking year, and they can make up a significant portion (up to 50%) of the fish encountered during March surveys. The adjusted ‘at large’ estimate excludes razorback sucker encountered that were released within a year of the marking year. This adjustment increases the likelihood that the estimate represents sexually mature razorback sucker. Because razorback sucker in Lake Mohave do not differ significantly from a 1:1 sex ratio (Turner et al. 2007), we estimated annual female census size (*N*_af_) as 0.5*N*_a_.

### *Evaluation of relationships of N*_b,_ *N*_e,_ *and N*_a_

Using the program AgeNe (Waples et al. 2011) and a static life table previously developed for razorback sucker (Turner et al. 2007), we calculated values of *N*_b_, *N*_e_, and *N*_a_ to assess their expected interrelationships. agene uses a hybrid Felsenstein–Hill model to estimate these parameters for species with overlapping generations (Waples et al. 2011, 2013). The model assumes that individuals of the same age and sex have random reproductive success. We also employed Jorde and Ryman's (1995, 1996) computational method to estimate values for *C *=* *40.37 and *G *=* *9.03 from the razorback life table (Turner et al. 2007), where *C* is a correction factor for overlapping generations and *G* is mean generation length. These values are used in computation of *N*_e_ from standardized variances in allele frequency shifts (*F′* – corrected for sampling variance) across sequential cohorts. Although specifically designed to estimate *N*_e_ for species with overlapping generations, the Jorde and Ryman method relies on a static life table for calculating *C* and *G*, thus resulting estimates of *N*_e_ should be viewed with caution (Robinson and Moyer 2012).

### Estimation of *N*_b_ and *N*_bf_

Analysis with agene indicated that *N*_b_* *= *N*_e_ for razorback sucker given the life table and model assumptions (see also Waples et al. 2013). We thus focus on *N*_b_ hereafter in the paper, but note that an estimate of *N*_b_ is also an estimate of *N*_e_, and vice versa. Estimates of *N*_b_ and associated 95% CIs were obtained for all samples obtained from 1997 to 2011, using two-sample (temporal method applied to sequential cohorts) and single-sample (analysis of linkage disequilibrium applied to individual cohorts) approaches. Initially, *N*_b_ was estimated using moment-based and pseudo-maximum likelihood procedures in mlne (Wang 2001), which require two samples at distinct time points (in between temporally adjacent larval cohorts). Because microsatellite loci typically possess many rare alleles that can upwardly bias estimates of effective size, the unbiased estimator *F*_S_ (Jorde and Ryman 2007) was used, as implemented in tempofs (http//www.zoologi.su.se/_ryman). For tempofs estimation, we used sampling plan 1 (Waples 1989), which requires an estimate of *N*_a_ in calculations.

The annual effective number of female breeders, *N*_bf_, was estimated from mtDNA haplotype frequency shifts across sequential larval cohorts, pooled across sampling localities within each sample year. We used Nei and Tajima's (1981) method to estimate the standardized variance of haplotype frequency shifts across cohort pairs, *F*_c_, corrected for sampling variance (using eq. 2 in Turner et al. 1999) to yield *F*_c_*′*. In this case, *N*_bf_ is proportional to *1*/*F*_c_*′*. Upper and lower-bound 95% confidence limits (CLs) around *F’* (and *N*_bf_) were calculated following Waples (1989, eq. 16). We also used mlne for estimation of *N*_bf_ and 95% CIs.

All two-sample estimates of *N*_b_, *N*_bf_ and 95% CLs described above were corrected for downward bias due to effects of overlapping generations via the Jorde and Ryman (1995) method. Although the method was developed for the estimation of *N*_e_, application to *N*_b_ in this case is justified because *N*_e _= *N*_b_ in razorback sucker. For estimation methods that do not return an *F*’ as standard output (e.g., mlne), we multiplied estimates by the ratio *C/G* to calculate corrected *N*_b_ or *N*_bf_ (e.g., Turner et al. 2006). Two-sample approaches produce values that most closely approximate the variance effective size of the population.

Single-sample estimates of *N*_b_ were generated using the program ldne (Waples and Do 2008), which most closely approximates the inbreeding effective size. The 2% allele frequency threshold was used, and 95% CLs were obtained from the jackknifing method (Waples and Do 2010). Point estimates of *N*_b_ based on an allele frequency threshold of 1% appeared to be biased upward with no appreciable gains in precision compared with those based on the 2% threshold. Values of *N*_b_ generated from ldne were not corrected via the Jorde and Ryman method.

Once obtained, all estimates of *N*_b_ and *N*_bf_ were paired to the appropriate time to which they refer (Waples 2005). For species with discrete generations, two-sample estimates were calculated using samples from time *i* to time *i +* 1, with the estimate of *N*_b_ corresponding to time *i* (Waples 2005). The reference time for species with overlapping generations is the harmonic mean between times *i* and *i + *1 (Hare et al. 2011). In contrast, single-sample estimates obtained from larvae sampled in time *i* are matched to the *N*_b_ in time *j*−1 (Waples 2005). For example, two-sample estimates of *N*_b_ obtained between years 1999 and 2000 reflects *N*_b_ in 1999 (discrete generations) or the harmonic mean between 1999 and 2000 (overlapping generations), whereas the single-sample estimate of *N*_b_ obtained from larvae sampled in 1999 represents *N*_b_ in 1998.

### Testing temporal trends

To test for nonrandom trends in *N*_b_*/N*_a_ and *N*_bf_*/N* af across time, we used a nonparametric ‘runs test above and below the median’ as described by Sokal and Rohlf (1995). First, separate tests were conducted for values obtained from each estimation method. Then, point estimates at each time step were combined within marker types (i.e., microsatellites [*Ñ*_b_] and mtDNA [*Ñ*_bf_]), by computing a weighted (by the coefficient of variation) harmonic mean value for each marker class (appendix A in Waples and Do 2010). Combined estimates were then subjected to nonparametric runs tests. The null hypothesis was that values of *N*_b_/*N*_a_ or *N*_bf_*/N*_af_ were randomly distributed across the time series (i.e., did not significantly increase or decrease). Probability values for the number of runs above and below the median were assigned via normal-distribution approximation (formulated based on Sokal and Rohlf 1995 and implemented in Microsoft excel). We evaluated the trajectory of deviations from the median value by plotting the difference of observed and median *N*_b_*/N*_a_ and *N*_bf_*/N*_af_.

## Results

### Mitochondrial DNA

Analysis of an additional 160 collections (3916 larvae) made from 2004–2011 yielded seven new variants, differing by only single substitutions from previously reported haplotypes (Tables S1 and S2). These new haplotypes are very rare, with the most common (CC) found at a frequency of 0.3%. Therefore, 35 haplotypes were identified in the 6347 total larvae from 259 collections.

Levels of genetic variation in samples collected from 2004–2011 were characterized for haplotypic richness and haplotype diversity (Table S3). Average haplotypic richness across all samples was 5.00, with a range of 1.00–8.20. Average haplotype diversity was 0.56, ranging from 0.00 to 0.82. Of 310 total comparisons analyzed statistically, 37 and 22 of the samples exhibited significantly reduced (*P *<* *0.05) levels of haplotypic richness and haplotype diversity, respectively, relative to remnant wild adult population, for a total of 19.0% of all tests. The number of significant tests is similar to 1997–2003 (Dowling et al. 2005), where 17 and 18 tests of haplotypic richness and gene diversity, respectively, were significant, representing 18.8% of all tests. anova identified significant differences in haplotypic richness among regions (*P *=* *0.004), with Tequila Cove exhibiting the fewest haplotypes per sample (average = 4.41) and Nine Mile area the most (average = 5.46). Bootstrap analysis of gene diversity among the four regions identified none of the six possible pairwise comparisons were significant after B-Y correction (Narum 2006).

Distribution of variation within and among samples from 1997–2011 was characterized using amova (Table 1). Estimates of *F*_ST_ were significant for each year except 2011, with an average of 0.061 (range 0.011 in 2011 to 0.147 in 2004). Most of this variation was explained by differences among temporal samples within regions (average *F*_SC _= 0.064; range 0.011–0.138), with insignificant levels of regional structure (average *F*_CT _= −0.003; range −0.021–0.023). The 2011 sample is noteworthy because it is the only year that we failed to identify differences among temporal samples.

**Table 1:** Results from amova analysis of mtDNA haplotype frequencies for razorback sucker from Lake Mohave, Arizona and Nevada, for each of the years represented.

Year	# of collections	*N*	*F*_ST_	*P*	*F*_CT_	*P*	*F*_SC_	*P*
1997	13	338	0.088	<0.0001	−0.021	0.845	0.110	<0.0001
1998	19	484	0.043	<0.0001	−0.002	0.512	0.045	<0.0001
1999	13	291	0.039	<0.0001	−0.012	0.715	0.050	0.001
2000	16	366	0.049	<0.0001	−0.009	0.758	0.058	<0.0001
2001	10	230	0.102	<0.0001	−0.001	0.522	0.103	0.001
2002	14	344	0.020	0.015	−0.004	0.651	0.024	0.016
2003	14	370	0.060	<0.0001	0.023	0.069	0.037	0.004
2004	24	559	0.147	<0.0001	0.010	0.240	0.138	<0.0001
2005	17	437	0.059	<0.0001	0.001	0.380	0.058	<0.0001
2006	23	571	0.062	<0.0001	0.000	0.430	0.063	<0.0001
2007	13	308	0.043	<0.0001	−0.012	0.740	0.054	<0.0001
2008	24	576	0.057	<0.0001	0.004	0.275	0.053	<0.0001
2009	21	515	0.097	<0.0001	−0.019	0.994	0.113	<0.0001
2010	19	478	0.042	<0.0001	−0.006	0.761	0.047	<0.0001
2011	19	469	0.011	0.059	0.000	0.51	0.011	0.074

**Figure 1:**
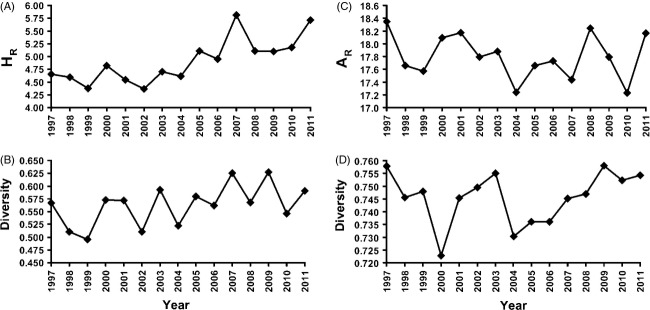
Mean haplotypic richness (A) and mean gene diversity (B) calculated from mtDNA haplotype data for razorback sucker from Lake Mohave, Arizona and Nevada. Data for 1997–2003 are provided in Dowling et al. (2005), with 2004–2011 provided in Table S1. Mean allelic richness (C) and mean gene diversity (D) calculated from microsatellite data for razorback sucker from Lake Mohave, Arizona and Nevada.

Distribution of variation across the 15-year period also was evaluated. Regression analysis indicated that haplotypic richness of samples pooled within year increased with time (*r* = 0.212, *P *=* *0.001, Fig. 1A). Likewise, haplotype diversity exhibited some covariation with time, but the comparison was not significant (*r *= 0.119, *P *=* *0.065, Fig. 1B).

### Microsatellites

Microsatellite variation was characterized using 15 loci on random subsamples of 120 individuals from each year (Table S4). Of 27 000 amplifications (15 loci per individual), only 37 (ca. 0.1%) failed to yield product. There were considerable differences in levels of variation among loci, with estimates of allelic richness (Table S5) ranging from 2.0 (Xte1) to 34.1 (Xte20). Allelic richness (averaged across loci) was less variable than mtDNA over time, ranging from 16.2 (2010) to 17.3 (1997), and differences in mean allelic richness among years (Table 2) were not significant (single factor anova,*P *=* *0.584). Analysis of gene diversity revealed similar results (Table S6), with estimates for loci ranging from 0.16 (Xte2) to 0.96 (Xte20) and years ranging from 0.72 (2000) to 0.76 (1997). As with allelic richness, a single factor anova failed to identify significant differences in gene diversity (Table 2) among years (*P *=* *0.083), with no pattern in variation over time.

**Table 2:** Mean and ranges of allelic richness (*A*_R_) and gene diversity from 15 microsatellite loci for razorback sucker from Lake Mohave, Arizona and Nevada, for each of the years represented.

Year	*A*_r_		Gene diversity	
	Mean	Range	Mean	Range
1997	17.3	2.0–34.9	0.758	0.171–0.955
1998	16.6	2.0–33.9	0.745	0.150–0.959
1999	16.5	2.0–35.8	0.748	0.104–0.957
2000	17.0	2.0–36.0	0.723	0.132–0.963
2001	17.1	2.0–33.0	0.745	0.112–0.960
2002	16.7	2.0–35.0	0.750	0.172–0.962
2003	16.8	2.0–34.9	0.755	0.171–0.958
2004	16.2	2.0–35.0	0.730	0.112–0.959
2005	16.6	2.0–34.9	0.736	0.146–0.959
2006	16.7	2.0–33.9	0.736	0.08–0.955
2007	16.4	2.0–35.9	0.745	0.187–0.962
2008	17.2	2.0–35.9	0.747	0.157–0.959
2009	16.7	2.0–34.0	0.758	0.192–0.963
2010	16.2	2.0–32.9	0.752	0.226–0.961
2011	17.1	2.0–32.9	0.754	0.165–0.957

Tests for deviation from Hardy–Weinberg Equilibrium (HWE) indicated a significant deficiency of heterozygotes (after B-Y correction) at 56 of the 225 year by locus comparisons. This result is not surprising as sample sizes are large and the sampling strategy pooled across significantly different samples within years, resulting in a Wahlund effect. Such patterns of deviation could also indicate existence of null alleles; therefore, several different approaches were examined to address this issue. Significant deviations were found to be scattered across 11 of the 15 loci, indicating results reflected a sample level phenomenon (such as the Wahlund effect) and not problems with individual loci. Regression of number of significant tests per locus against allelic richness identified a significant relationship (*r* = 0.698, *P *=* *0.004), indicating that loci with more alleles were more likely to exhibit deviation from HWE. The most variable locus, Xte22, accounted for 25% (14 of 56) of observed deviations from HWE. We dropped this locus from the dataset, recalculated all estimates of genetic diversity and *N*_e_ and compared values between original and truncated datasets. Results were nearly identical between datasets, suggesting negligible effect of HWE deviations on accuracy or precision of estimates, or the validity of our conclusions. In addition, Dowling et al. (2012b) identified only two of 65 comparisons exhibiting deviations from HWE in a basin wide study of razorback sucker. One of those comparisons involved Xte7, which is known to have null alleles (Saltzgiver, personal communication); however, these were rare (<6%). Thus, we report values from the original dataset throughout.

The distribution of variation across the 15-year period was also evaluated. Regression analyses did not identify a relationship between time and measures of genetic variation (allelic richness, *r* = 0.007, *P *=* *0.914, Fig. 1C; arcsine transformed gene diversity, *r* = 0.007, *P *=* *0.918, Fig. 1D).

Microsatellite variation was partitioned into within-and among-year components (Table S7). Jackknife estimates of total genetic variation (*F *≈ *F*_IT_) for each locus ranged from −0.055 to 0.165 (Xte2 and Xte22, respectively), with a jackknife average across loci of 0.063 (95% bootstrap CI 0.043–0.085). When examining individual loci, the within population component (*f *≈ *F*_IS_) ranged from −0.055 to 0.164 (Xte2 and Xte22, respectively) with a jackknife average of 0.058, which was significantly different from zero (95% bootstrap CI 0.039–0.080), a result that is consistent with HWE results discussed above. The among-year component (Θ ≈ *F*_ST_) was an order of magnitude smaller than that within years, ranging from 0 (Xte2 and Xte25) to 0.027 (Xte10), with a significant jackknife average of 0.005 (95% bootstrap CI 0.002–0.010).

### Annual census estimates

**Figure 2:**
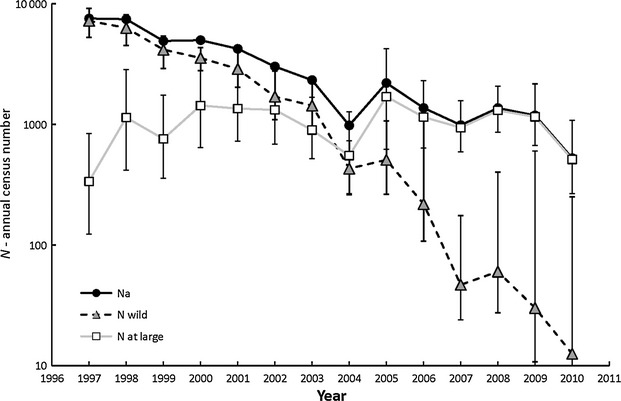
Annual estimates of the number of reproductively capable wild razorback sucker (*N*_wild_ – gray triangles) and repatriated fish (*N*_at large_ – open squares) in Lake Mohave, Arizona and Nevada, over a 14-year time series. The total annual census size (*N*_a_ – closed circles) is the sum of *N*_wild_ and *N*_at large._ Estimates of *N*_wild_ and *N*_at large_ and 95% confidence intervals (CIs) (error bars) are based on mark–capture–recapture data.

Adult wild razorback sucker abundance estimates declined consistently after the early 1980s to the point indicative of functional extirpation. The Lake Mohave population, initially estimated at more than 60 000 fish in the late 1980s, declined to fewer than 50 wild fish in the past 20 years (Fig. 2). Meanwhile, although more than 155 000 razorback sucker have been repatriated, the repatriate population estimates have remained fairly static between 1000 and 3000 fish. The ‘at large’ repatriate estimate was generally greater than 75% of the repatriate population, except in 2004 and 2010 when it represented 50% and 21% of the total repatriate population, respectively. Both followed a year when relatively large numbers of razorback sucker were repatriated to Lake Mohave (16 844 in 2003 and 12 531 in 2009).

In 1997, wild fish comprised >95% of the annual spawning stock (*N*_a_) in Lake Mohave; However, by 2003 wild fish comprised roughly 50%, and by 2007 represented less than 5% of *N*_a_ (Fig. 2). Although their numbers remained relatively constant over the same period, reproductively mature repatriated fishes (*N*_at large_) now make up more than 95% of *N*_a_. Overall, *N*_a_ has declined sevenfold, from approximately 7500 to about 1000 over the study period (Fig. 2). Arithmetic mean *N*_a_ evaluated over the time series is 3072 and harmonic mean *Ñ*_a_ is 1670; arithmetic mean *N*_af_ was 1536 and harmonic mean *Ñ*_af_ was 835.

### *Estimates of N*_b_ and *N*_bf_

**Figure 3:**
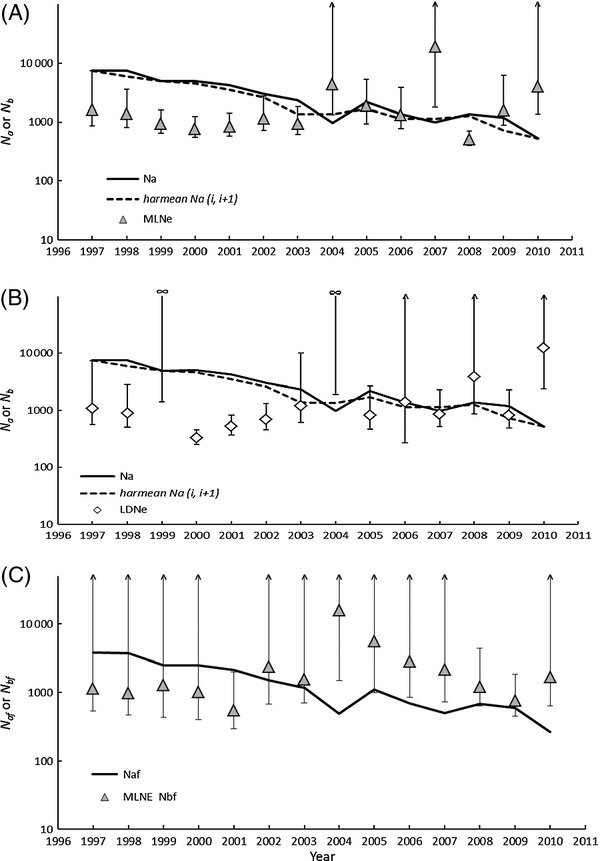
Annual estimates of *N*_a_ or *N*_af_ (solid black line), *N*_b_*,* and *N*_bf_ plotted by sample year for razorback sucker in Lake Mohave, Arizona and Nevada. Panel (A) is mlne estimates based on microsatellites, panel (B) ldne estimates based on microsatellites, and panel (C) is mlne based on mtDNA. The dashed line is harmonic mean *N*_a_ calculated between sample years *i* and *i *+* *1 (*Ñ*_a(i, i+1)_). Error bars are 95% CLs. The infinity symbol represents an estimate equal to infinity. Likewise, an error bar with an upward arrow represents an upper 95% CL equal to infinity.

Two-sample and single-sample estimates of *N*_b_ were similar in magnitude (Table 3), and all tended to remain stable or increase (depending on the estimator) over the time series (Table 3, Fig. 3). Upper-bound confidence limits for *N*_b_ estimates were typically less than *N*_a_ (and its lower-bound CI) early in the time series (from 1997 to 2003, Fig. 3A,B) but generally exceeded *N*_a_ after 2004. General trends in the relationship of *N*_b_ and annual census size did not change when *Ñ*_ai,i+1_ was substituted for *N*_a_ (Fig. 3A,B). Estimates of *N*_bf_ based on mlne and *F*_c_ showed a similar trajectory [Table 3, Fig. 3C (*F*_c_ not shown)] to those revealed by microsatellite data. However, estimates of *N*_bf_ were less precise than *N*_b_ because they are based on a single genetic locus with many rare haplotypes (Fig. 3C).

**Table 3:** Annual census sizes (*N*_a_), estimates of the effective number of breeders (*N*_b_) based on microsatellites, and effective number of female breeders (*N*_bf_) based on mtDNA data. Parentheses indicate lower-and upper-bound 95% confidence intervals (CIs) for each estimate.

Year*	*N*_a_†	Microsatellites – *N*_b_			MtDNA – *N*_bf_	
		mlne‡	Tempo *F*_S_§	ldne¶	mlne‡	*F*_c_**
1997	7532 (5359, 9997)	1609 (867, 7408)	711 (398, 3362)	1072 (565, 7388)	1136 (528, ∞)	876 (291, 4341)
1998	7427 (4910, 10 927)	1364 (814, 3644)	291 (165, 1180)	882 (512, 2822)	966 (465, ∞)	1149 (335, 32 493)
1999	4910 (3263, 7147)	934 (644, 1600)	143 (94, 277)	∞ (1398, ∞)	1261 (429, ∞)	711 (215, 4582)
2000	4978 (3426, 7885)	769 (550, 1243)	165 (98, 528)	326 (251, 456)	1006 (398, ∞)	921 (250, ∞)
2001	4224 (2748, 6986)	832 (572, 1435)	250 (148, 903)	520 (373, 833)	536 (291, 1989)	398 (130, ∞)
2002	3012 (1779, 5525)	1162 (720, 2758)	291 (165, 1113)	688 (459, 1320)	2334 (666, ∞)	733 (237, ∞)
2003	2323 (1405, 4109)	943 (621, 1828)	246 (112, ∞)	1188 (618, 10 040)	1551 (702, ∞)	1203 (353, 9634)
2004	979 (526, 1999)	4377 (1337, ∞)	1341 (367, ∞)	∞ (1901, ∞)	15 647 (1475, ∞)	13 832 (943, ∞)
2005	2200 (883, 5301)	1909 (934, 5311)	608 (282, ∞)	814 (469, 2678)	5566 (984, ∞)	2320 (528, ∞)
2006	1366 (741, 3387)	1314 (764, 3867)	389 (232, 1274)	1390 (269, ∞)	2817 (845, ∞)	1574 (331, ∞)
2007	984 (614, 1738)	19 054 (1775, ∞)	429 (188, ∞)	852 (515, 2268)	2146 (724, ∞)	2597 (496, ∞)
2008	1364 (888, 2474)	514 (402, 711)	107 (54, ∞)	3876 (860, ∞)	1189 (630, 4390)	773 (264, 2119)
2009	1183 (679, 2763)	1587 (881, 6219)	840 (425, 34 487)	821 (491, 2295)	747 (443, 1824)	581 (228, 1498)
2010	524 (270, 1326)	3970 (1355, ∞)	5494 (671, ∞)	12 391 (1393, ∞)	1677 (630, ∞)	1395 (429, 14 405)

* Results are matched to the year to which the estimates apply following Waples (2005).

† Annual census size is the sum of wild and ‘at large’ repatriated fish, see text for more details.

‡ mlne is the pseudo-likelihood estimator of Wang (2001).

§ TempoFS is the moments-based estimator of Jorde and Ryman (2007).

¶ ldne is the linkage-disequilibrium-based method of Waples and Do (2008).

** *F*_c_ is the moments-based estimator of Nei and Tajima (1981).

### *Temporal Trends in N*_b_*/N*_a_ and *N*_bf_*/N*_af_

**Figure 4:**
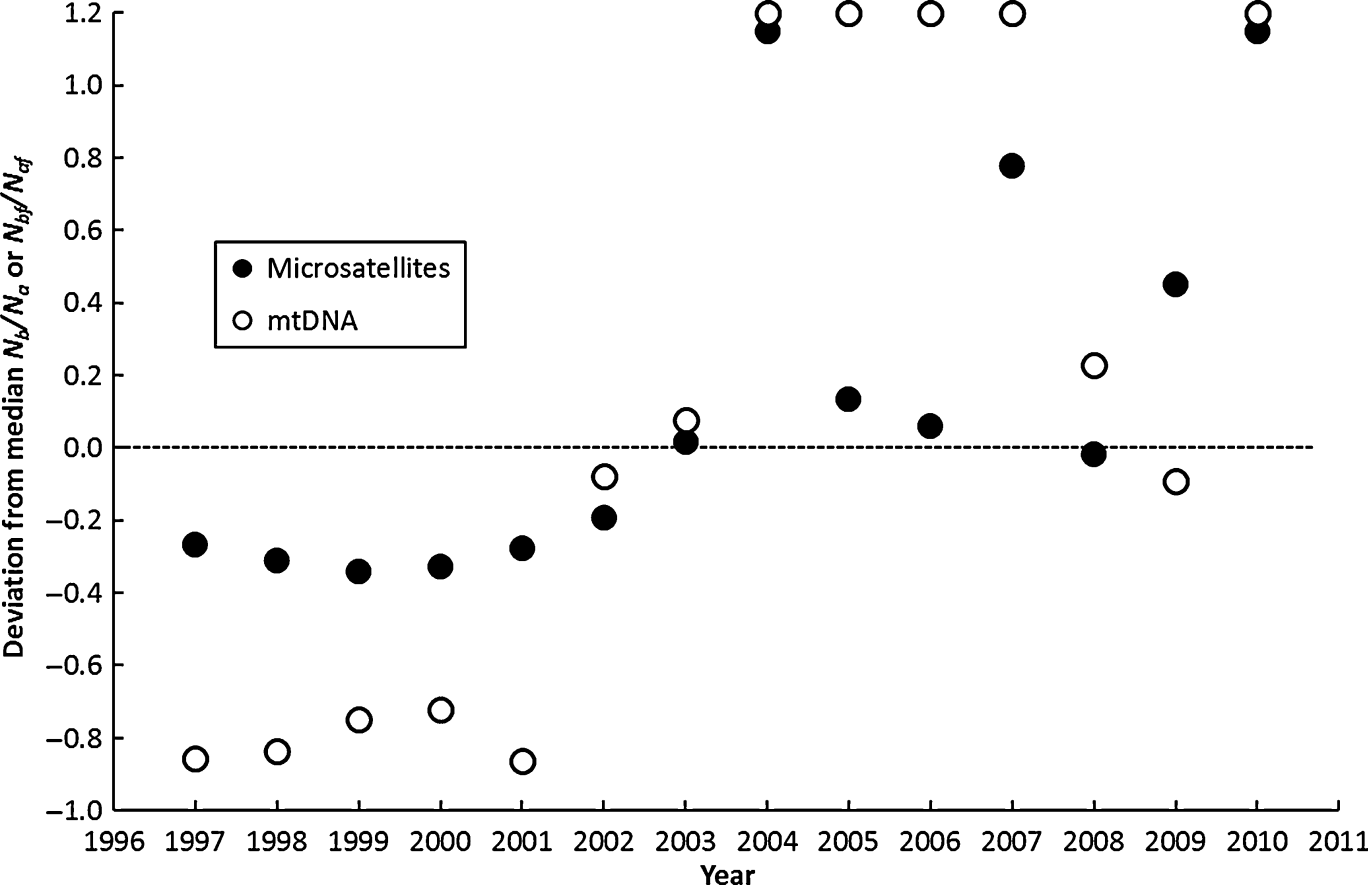
Deviations of observed and median *Ñ*_b_*/N*_a_ or *Ñ*_bf_*/N*_af_ plotted by time. The dashed line indicates null expectation, that is, that observed and median values are identical. Two combined estimators (using the method of Waples and Do 2010, appendix A) are depicted (for simplicity), and both show significant departure from a random sequence of ratios across time (with 4 runs, 7 observations below and 7 above the median, *P *<* *0.035 in both cases). Ratios greater than 1 were included runs tests, but are depicted on the figure at an arbitrary maximum value of 1.2 to simplify presentation. Observed values are always below the median prior to 2003, and nearly always above the median value later in the time series.

The ratio of effective number of breeders and annual spawning stock increased significantly over time, for *N*_b_*/N*_a_ based on mlne,TempoFS,ldne, and *N*_b_*/N*_af_ based on *F*_c_. Results of nonparametric runs tests indicated significant deviation from a random sequence for these four estimators used (*P *<* *0.035 for all tests). Only the estimate *N*_bf_*/N*_af_ based on mlne (mtDNA) was not significantly different from random dispersion across the time series (*P *=* *0.21). Likewise, combined and weighted estimates for both marker classes also exhibited significant increases (*P *<* *0.035) in *Ñ*_b_*/N*_a_ or *Ñ*_bf_*/N*_af_ over time (Fig. 4). Plots of deviations from the median by time revealed that estimates of *N*_b_*/N*_a_ or *N*_bf_*/N*_af_ were nearly always lower than the median value before 2003 but were nearly always above the median after 2003 for combined estimates (Fig. 4) and all estimates plotted separately (not shown).

## Discussion

Management plans for exploited (i.e., commercially important) or rare and endangered fishes increasingly include goals and targets that aim toward sustainability and recovery of census numbers (i.e., the size of the spawning stock) and genetic diversity. In general, such plans usually implicitly assume that census size and genetic diversity are directly correlated, but this is usually not the case because of intrinsic differences among species (e.g., life-history, mating system, etc.), differences in extrinsic forces that affect recruitment and survival (e.g., biotic and abiotic environmental features), and/or interactions between intrinsic and extrinsic forces that alter the relationship of census size and diversity across species (e.g., Turner et al. 2006). Coupled demographic and genetic time-series data offer the potential to elucidate the relationship of spawning stock size and genetic diversity within a particular species, providing the critical link of specific management actions to trends in key metrics identified as important to secure long-term sustainability of the species.

This study illustrates some advantages and potential pitfalls of a time-series approach as applied to a long-lived, iteroparous fish species. The 15-year period of 1997–2011 was critical for razorback sucker in Lake Mohave, as census size declined precipitously and the population is currently in the low 1000s and composed almost entirely of repatriates. Dowling et al. (2005) previously characterized mtDNA variation in temporal samples from 1997–2003. They found that individual temporal samples were significantly different from each other in terms of levels of variation and allele frequency; however, there were no differences among different spawning areas in the lake or years. The most plausible explanation for this pattern is that each temporally and spatially discrete sample of larvae is produced by a relatively small number of females (Dowling et al. 2005; Turner et al. 2007). Comparison of groups of samples from each year with samples of wild and repatriate adults failed to detect significant differences, indicating the proscribed strategy for collecting larvae is maintaining genetic variation found in the original wild population and it is being retained in the repatriates.

This analysis represents an extension of that previous work, focused specifically on genetic variation in larvae, including samples from 2004–2011. Analyses of mtDNA variation in this set of samples yielded similar patterns of variation within samples and again identified significant differences among individual temporal samples within regions each year (with the notable exception of 2011) but not among geographic regions. Our inability to detect such differences in 2011 is consistent with an overall increase in number of females producing offspring, which thereby reduced impacts of sampling that had previously led to observed differences among individual samples.

Seven new haplotypes were identified relative to the previous seven-year period, each at low frequency (<1%, Table S1), and differing by a single mutation from previously identified haplotypes. The adjacent upstream population in Lake Mead is small and, to our knowledge, does not contain these haplotypes (Dowling et al. 2012a,b2012b); therefore, these haplotypes likely do not result from immigration into the system. Given the large number of samples assayed and rapid rate of mtDNA evolution, these haplotypes could have arisen by mutation during this period; however, a more likely explanation is that rarity of these haplotypes makes them more difficult to sample, and they simply were not represented in earlier samples. Microsatellite variation was also assayed from a subset of these samples, providing results generally consistent with mtDNA. Analyses of variation failed to detect significant differences among years at microsatellites, indicating the conservation program is reliably representing levels and patterns of neutral genetic variation each year.

Analyses of levels of mtDNA variation over this period provided some especially interesting results, identifying a significant increase in average number of haplotypes and genetic diversity per temporal sample over time. Observed increases in measures of diversity reflect an increase in frequency and number of rare haplotypes over time, consistent with an increase in number of females that are contributing progeny during each individual time period. In contrast to results for mtDNA, the analyses of nuclear DNA variation (as represented by 15 loci) from a subset of individuals (120 randomly selected wild-caught larvae per year) did not reveal increased diversity, as allelic richness and gene diversity were effectively unchanged over the 15-year period. This is similar to results of Osborne et al. (2012) who reported increases in haplotype diversity, but stability of microsatellite diversity over a 12-year continuous time series for endangered Rio Grande Silvery Minnow (*Hybognathus amarus*). Unlike long-lived and age-structured razorback sucker populations, this species is essentially annual. Both of these examples illustrate the significance of long-term genetic monitoring for evaluation of trends in genetic diversity.

As noted above, differences in levels of variation exhibited by mtDNA and microsatellites observed here are likely explained by differences in modes of inheritance between mtDNA and nuclear markers. MtDNA is strictly maternally inherited, and only a single variant can be transmitted to progeny (per family); therefore, assuming equal sex ratios and no selection, the effective number of breeders measured by mtDNA is expected to be about ¼ that of an autosomal nuclear gene over evolutionary time (Hedrick 2005; Charlesworth 2009). Thus, mtDNA is expected to be more sensitive to random effects of sampling during reproduction than are nuclear genes. Unfortunately, the contrast may not be this simple. Frankham (2012) noted that levels of genetic diversity correlated closely with population size except for mtDNA, where results were mixed. Mixed results from mtDNA likely reflect variation in mutation rates among genes and taxa as well as potential impacts of selection for this nonrecombinant molecule. When specific taxa were the focus of comparison (as in this study), mtDNA results were consistent with nuclear genome markers, and there was a positive relationship between levels of genetic diversity and population size.

Estimates of annual number of effective breeders (*N*_b_ and *N*_bf_) tended to remain stable, or perhaps increase over the time series (Fig. 3) despite precipitous decline in *N*_a_ due to loss of wild fish from the system. This means that more fish are breeding *per capita* after implementation of the repatriation program and loss of wild fish. However, some individual point estimates of *N*_b_ and *N*_bf_ are larger than expected (Fig. 3). For example, in 2004, all estimators of *N*_b_ and *N*_bf_ equaled or exceeded *N*_a_ and *N*_af_, respectively, resulting in ratios that exceeded one. Years 2003 and 2004 were two of the largest repatriation events in the time series (34 112 fish total) and 2003 represented a key tipping point where repatriates made up > 50% of the total spawning stock for the first time. We also observed a number of cases where *N*_bf_ exceeded *N*_b_ in 2003 through 2005. A decrease in variance of female reproductive success (or an increase in variance of male reproductive success, or both) during this period may have driven *N*_bf_ to higher than expected levels. It is also possible that *N*_bf_* *> *N*_b_ because many low-frequency alleles were present in the mtDNA dataset, which can upwardly bias estimates of *N*_bf_ (e.g., Turner et al. 2001). Neither mlne (Wang 2001) nor *F*_c_ (Nei and Tajima 1981) estimators are explicitly formulated to accommodate this bias. Taken together, these results suggest that pinpointing a specific cause for higher than expected values in the time series is difficult because estimators *N*_b_*/N*_a_ and *N*_bf_*/N*_af_ are imprecise and it is difficult to distinguish biological effects from noise under these conditions.

Outliers notwithstanding, there was strong statistical support for a trend of increasing *N*_b_*/N*_a_ over the time series based on nonparametric runs tests. There are at least two explanations for this observation. First, a genetic ‘compensatory’ effect (Ardren and Kapuscinski 2003) may have yielded lowered variance in reproductive success among families when density of spawners was reduced, such that *N*_b_ increased (or stabilized) despite sharp decline of *N*_a_ over the time series. Second, the repatriation program has reduced the realized maximum life span and generation time in Lake Mohave razorback sucker, which is expected to alter relationships of *N*_b_, *N*_e_, and *N* in theory (Waples et al. 2011). Because of predation, maximum age is now about 20 years compared with 44 years (or older) historically (McCarthy and Minckley 1987).

To explore the possibility that changes in age structure led to increases in *N*_b_*/N*_a_, we conducted additional analysis in agene with a life table truncated to a maximum life span of 20 years. Output from the program indicated that reducing maximum age to 20 years (while holding all other variables equal to conditions described in the materials and methods) yielded *N*_b _= 0.95*N_e_* *= *N*, which is not appreciably different from results where maximum age was 44 years (i.e., *N*_b_* = N*_e _*= N*). Thus, we conclude that changes in age structure do not fully explain increased *N*_b_*/N*_a_ over the 15-year study period and that compensatory effects are likely the primary cause for increase in this ratio over time in razorback sucker.

### Importance of genetic diversity and population size in conservation

The case for razorback sucker illustrates the efficacy of a time-series approach as a tool to study long-lived species in quasi-natural conditions; our study clearly demonstrates maintenance (and perhaps increase) of neutral genetic diversity over time despite population decline and leads to several important hypotheses about effects of age structure, population stability, and compensation as factors in maintenance of diversity.

Other studies have demonstrated an increase in genetic variation over time by the addition of individuals to the system [e.g., Swedish adder (*Vipera berus*), Madsen et al. 1999; chinook salmon (*Oncorhynchus tshawytscha*), Hedrick et al. 2000; Florida panther (*Puma concolor coryi*), Hostetler et al. 2013], demonstrating the importance of increasing genetic variation. The increase in genetic variation in razorback sucker has been accomplished without input of individuals from external sources—only larvae produced in the system are repatriated to the lake. To our knowledge, this is among the first examples where conservationists have been able to use a management strategy involving protective custody to increase levels of neutral genetic variation in a wild (versus captive) population (see also Osborne et al. 2012). This is truly remarkable given large size of the lake, large number of individuals involved, and high levels of annual variance in reproductive success typically associated with large, highly fecund, long-lived species (Hedgecock 1994).

Despite our ability to successfully manage neutral genetic variation in the razorback sucker population of Lake Mohave, we are still constrained in our efforts to enhance genetic variation and need to guard against risks associated with low levels of genetic variability. Persistently low census size will ultimately result in loss of genetic variation due to random sampling of genetic variation during the process of transmission of variants from adults to progeny, such that adaptive potential of the species would be compromised and extinction processes associated with stochastic factors would be accelerated (reviewed in Palstra and Ruzzante 2008). Rate of loss is directly related to number of individuals contributing each generation, with smaller populations losing genetic variation more rapidly than larger ones. Furthermore, a more strongly age-structured population has more genetic resiliency due to a ‘genetic storage effect’ that tends to preserve diversity even when recruitment is poor in a particular year (Berkeley et al. 2004).

It is also important to note that our estimates of *N*_b_ and *N*_e_ are presumably based on the amount of neutral genetic variation, which is variation that has no effect on reproductive success of individuals within the population. Number of individuals necessary to maintain genetic variation at quantitative traits (those most likely to respond to selection and result in differences in reproductive success among individuals) is much larger (perhaps more than an order of magnitude – Lynch 1996) than for neutral traits like those represented by these mtDNA sequence variants and microsatellites. This means that even though we have been able to maintain (or perhaps increase) standing levels of genetic diversity of razorback sucker within Lake Mohave, which is of course the best possible scenario, population size is small enough that we still need to be concerned about our ability to maintain genetic variation that enhances adaptability.

The above points illustrate potential problems with use of genetic variation and effective population size in formulating conservation plans. While estimates of these parameters are important in developing conservation strategies (reviewed in Leberg 2005; Schwartz et al. 2007), it is crucial to keep in mind the above considerations when generating target census population numbers. In addition, patterns of change in these estimates are also critically important and must be considered when developing conservation strategies. In the case of razorback sucker from Lake Mohave, levels of genetic variation and *per capita* effective number of breeders have increased over time, placing us in the position of being able to maintain or increase levels of genetic variation, at least for the time being. It is important to note, however, our ability to increase genetic variation will be constrained by the actual number of fish in the lake. The current census number is low, and we are at risk of continued loss of essential variation. This problem can only be remedied by increasing the number of genetically diverse individuals in the wild. Due to the perilous state of the population, it is crucial that we implement whatever means necessary to increase the number of genetically diverse razorback sucker in the wild.

### General conclusions

Our case study demonstrates utility of time-series analysis to study trends in metrics commonly employed in genetic monitoring studies, even for long-lived and iteroparous species. For metrics that can be measured fairly precisely, such as genetic diversity and allelic richness, statistical methods like ordinary least-squares regression may be sufficient to test for changes over time. However, for more complex metrics like *N*_b_*/N*_a_, lack of precision of point estimates may limit utility of linear models for trend detection, and for outlier analysis of specific time periods. As an alternative, we suggest that nonparametric test procedures are much more powerful because they are distribution-free and statistical power to reject the null is positively related to number of observations in the time series and accuracy, rather than precision, of point estimates of metrics (Sokal and Rohlf 1995). As a case in point, we demonstrated that a statistically significant positive trend of *N*_b_*/N*_a_ and *N*_bf_*/N*_af_ could be detected for two genetic marker classes (microsatellites and mtDNA), and a number of estimation procedures that are known to differ widely in the precision of estimates that they return (Waples and Do 2010). Uniformity of trend detection for these ratios is especially interesting given a positive trend for increasing genetic diversity was only detected in the mtDNA dataset for razorback sucker.

Nevertheless, our analysis suggests that there may be cases where low precision of estimates could become an important limiting factor for time-series analysis of *N*_b_*/N*_a_ ratios. For example, two of 14 point estimates of *N*_b_ (and 5 of 14 upper-bound CIs) based on ldne could not be distinguished from infinitely large values, despite relatively large sample sizes (minimum sample sizes of 120 individuals per larval cohort) and a reasonable number of marker loci (15 microsatellite loci). It is thus possible that *N*_b_*/N*_a_ of razorback sucker may be approaching an upper limit that would preclude detectability of increasing trends in the future. In this case, limited statistical power could lead a manager to erroneously draw a conclusion of ‘no temporal change’ in the ratio when, in fact, it was responding positively to management action. In practice, failure to detect differences between ‘no change’ and ‘positive response’ may not be critical for a very large population, or when *N*_b_*/N*_a _≥ 1.

Simulation study (Antao et al. 2011) indicates it is much less likely that a decreasing trend in *N*_b_ or *N*_b_*/N*_a_ would go undetected if a population bottleneck were relatively severe. This is because precision of estimates increase as *N*_b_ becomes smaller, and precision increases as *N*_a_ gets larger (analysis not shown, see also Beebee 2009). Thus, statistical power to detect significant changes will be highest when *N*_b_ is relatively small and *N*_a_ is large (and *N*_b_/*N*_a_ < 1).

For most conservation applications, a decreasing trend in *N*_b_*/N*_a_ is an important case where ‘genetic’ intervention becomes high priority. Conversely, when *N*_a_ is relatively small and *N*_b_ is large (*N*_b_*/N*_a_ > 1), demographic intervention may be more important for immediate conservation action as described above for the razorback sucker. Thus, based on time-series data for razorback sucker, we emphasize increases in actual fish numbers as the highest management priority based on time-series analysis of genetic metrics.

## Data archiving

Data for this study are available from the Dryad Digital Repository: http://dx.doi.org/10.5061/dryad.2g9p0

## Supporting Information

**Table S1.** Frequency of each mtDNA haplotype for each year. **Table S2.** MtDNA haplotypes for each individual. Collection # provides the abbreviated locality information and sample number. **Table S3.** Population genetic statistics for mtDNA haplotypes from each sample collected from 2004–2011 for razorback sucker in Lake Mohave, Arizona and Nevada. **Table S4.** Microsatellite genotypes for individuals examined in this study. **Table S5.** Estimates of allelic richness estimated from 15 microsatellite loci for razorback sucker from Lake Mohave, Arizona and Nevada, from the years 1997–2011. **Table S6.** Estimates of gene diversity estimated from 15 microsatellite loci for razorback sucker from Lake Mohave, Arizona and Nevada, from the years 1997–2011. **Table S7.** Mean F-statistics and their standard errors for razorback sucker from Lake Mohave, Arizona and Nevada, (obtained by jackknifing across populations) calculated for each locus, including minimum and maximum values.
