# Supplementary material for: Time-series analysis reveals genetic responses to intensive management of razorback sucker (Xyrauchen texanus)
Source: Evol Appl. 2013 Nov 15;7(3):339–54. doi: 10.1111/eva.12125 (PMC3962295; doi:10.1111/eva.12125)
Supplement: Supplementary file 1 — Table S1. Frequency of each mtDNA haplotype for each year. Table S2. MtDNA haplotypes for each individual. Collection # provides the abbreviated locality information and sample number. Table S3. Population genetic statistics for mtDNA haplotypes from each sample collected from 2004–2011 for razorback sucker in Lake Mohave, Arizona and Nevada. Table S4. Microsatellite genotypes for individuals examined in this study. Table S5. Estimates of allelic richness estimated from 15 microsatellite loci for razorback sucker from Lake Mohave, Arizona and Nevada, from the years 1997–2011. Table S6. Estimates of gene diversity estimated from 15 microsatellite loci for razorback sucker from Lake Mohave, Arizona and Nevada, from the years 1997–2011. Table S7. Mean F-statistics and their standard errors for razorback sucker from Lake Mohave, Arizona and Nevada, (obtained by jackknifing across populations) calculated for each locus, including minimum and maximum values. [file eva0007-0339-sd1.pdf]

Supplemental Table 1. Frequency of each mtDNA haplotype for each year.

	total		1997		1998		1999	
Haplotype	#	FREQUENCY	#	FREQUENCY	#	FREQUENCY	#	FREQUENCY
A	288	0.045	9	0.027	12	0.025	3	0.010
B	548	0.088	47	0.139	36	0.074	27	0.093
C	130	0.020	18	0.053	8	0.017	0	0.000
D	5	0.001	0	0.000	0	0.000	0	0.000
E	3899	0.616	197	0.583	321	0.663	199	0.684
F	138	0.022	13	0.038	14	0.029	13	0.045
G	49	0.007	1	0.003	1	0.002	2	0.007
H	52	0.009	2	0.006	6	0.012	1	0.003
I	23	0.003	1	0.003	2	0.004	0	0.000
J	57	0.009	7	0.021	9	0.019	1	0.003
K	39	0.006	3	0.009	4	0.008	0	0.000
L	5	0.001	2	0.006	0	0.000	0	0.000
M	32	0.005	3	0.009	2	0.004	1	0.003
N	3	0.001	1	0.003	0	0.000	2	0.007
O	5	0.001	1	0.003	0	0.000	0	0.000
P	69	0.010	8	0.024	5	0.010	4	0.014
Q	20	0.003	1	0.003	2	0.004	0	0.000
R	440	0.070	18	0.053	29	0.060	18	0.062
S	380	0.060	6	0.018	22	0.045	12	0.041
T	1	0.000	0	0.000	0	0.000	0	0.000
U	19	0.003	0	0.000	0	0.000	3	0.010
V	54	0.009	0	0.000	5	0.010	3	0.010
W	4	0.001	0	0.000	0	0.000	2	0.007
X	2	0.000	0	0.000	0	0.000	0	0.000
Y	1	0.000	0	0.000	0	0.000	0	0.000
Z	19	0.003	0	0.000	4	0.008	0	0.000
AA	1	0.000	0	0.000	1	0.002	0	0.000
BB	21	0.003	0	0.000	1	0.002	0	0.000
CC	16	0.003	0	0.000	0	0.000	0	0.000
DD	1	0.000	0	0.000	0	0.000	0	0.000
EE	1	0.000	0	0.000	0	0.000	0	0.000
FF	4	0.001	0	0.000	0	0.000	0	0.000
GG	4	0.001	0	0.000	0	0.000	0	0.000
HH	5	0.001	0	0.000	0	0.000	0	0.000
II	1	0.000	0	0.000	0	0.000	0	0.000
	6336	1.000	338	1.000	484	1.000	291	1.000

Supplemental Table 1. Continued

	2000		2001		2002		2003	
Haplotype	#	FREQUENCY	#	FREQUENCY	#	FREQUENCY	#	FREQUENCY
A	20	0.055	5	0.022	14	0.041	21	0.057
B	39	0.107	33	0.143	19	0.055	35	0.095
C	4	0.011	0	0.000	6	0.017	8	0.022
D	0	0.000	0	0.000	0	0.000	1	0.003
E	229	0.626	136	0.591	237	0.689	214	0.578
F	4	0.011	7	0.030	9	0.026	3	0.008
G	3	0.008	0	0.000	1	0.003	4	0.011
H	3	0.008	0	0.000	1	0.003	0	0.000
I	0	0.000	0	0.000	0	0.000	1	0.003
J	3	0.008	1	0.004	2	0.006	1	0.003
K	0	0.000	3	0.013	1	0.003	4	0.011
L	0	0.000	0	0.000	0	0.000	0	0.000
M	8	0.022	1	0.004	2	0.006	2	0.005
N	0	0.000	0	0.000	0	0.000	0	0.000
O	0	0.000	2	0.009	0	0.000	0	0.000
P	3	0.008	1	0.004	6	0.017	0	0.000
Q	1	0.003	0	0.000	0	0.000	5	0.014
R	17	0.046	11	0.048	22	0.064	30	0.081
S	21	0.057	20	0.087	23	0.067	35	0.095
T	1	0.003	0	0.000	0	0.000	0	0.000
U	5	0.014	4	0.017	0	0.000	1	0.003
V	4	0.011	5	0.022	1	0.003	2	0.005
W	1	0.003	0	0.000	0	0.000	1	0.003
X	0	0.000	0	0.000	0	0.000	2	0.005
Y	0	0.000	1	0.004	0	0.000	0	0.000
Z	0	0.000	0	0.000	0	0.000	0	0.000
AA	0	0.000	0	0.000	0	0.000	0	0.000
BB	0	0.000	0	0.000	0	0.000	0	0.000
CC	0	0.000	0	0.000	0	0.000	0	0.000
DD	0	0.000	0	0.000	0	0.000	0	0.000
EE	0	0.000	0	0.000	0	0.000	0	0.000
FF	0	0.000	0	0.000	0	0.000	0	0.000
GG	0	0.000	0	0.000	0	0.000	0	0.000
HH	0	0.000	0	0.000	0	0.000	0	0.000
II	0	0.000	0	0.000	0	0.000	0	0.000
	366	1.000	230	1.000	344	1.000	370	1.000

Supplemental Table 1. Continued

	2004		2005		2006		2007	
Haplotype	#	FREQUENCY	#	FREQUENCY	#	FREQUENCY	#	FREQUENCY
A	31	0.055	32	0.073	20	0.035	14	0.045
B	31	0.055	36	0.082	66	0.116	22	0.071
C	13	0.023	3	0.007	8	0.014	10	0.032
D	0	0.000	1	0.002	1	0.002	0	0.000
E	358	0.640	267	0.611	344	0.602	178	0.578
F	12	0.021	8	0.018	13	0.023	8	0.026
G	3	0.005	2	0.005	4	0.007	1	0.003
H	3	0.005	6	0.014	5	0.009	8	0.026
I	1	0.002	2	0.005	0	0.000	1	0.003
J	2	0.004	4	0.009	6	0.011	2	0.006
K	2	0.004	4	0.009	3	0.005	3	0.010
L	0	0.000	0	0.000	1	0.002	0	0.000
M	0	0.000	1	0.002	3	0.005	2	0.006
N	0	0.000	0	0.000	0	0.000	0	0.000
O	0	0.000	0	0.000	0	0.000	1	0.003
P	2	0.004	4	0.009	1	0.002	2	0.006
Q	1	0.002	0	0.000	0	0.000	1	0.003
R	48	0.086	33	0.076	45	0.079	20	0.065
S	33	0.059	25	0.057	26	0.046	30	0.097
T	0	0.000	0	0.000	0	0.000	0	0.000
U	1	0.002	0	0.000	0	0.000	1	0.003
V	14	0.025	6	0.014	3	0.005	0	0.000
W	0	0.000	0	0.000	0	0.000	0	0.000
X	0	0.000	0	0.000	0	0.000	0	0.000
Y	0	0.000	0	0.000	0	0.000	0	0.000
Z	1	0.002	1	0.002	2	0.004	0	0.000
AA	0	0.000	0	0.000	0	0.000	0	0.000
BB	0	0.000	1	0.002	6	0.011	2	0.006
CC	2	0.004	1	0.002	8	0.014	1	0.003
DD	1	0.002	0	0.000	0	0.000	0	0.000
EE	0	0.000	0	0.000	1	0.002	0	0.000
FF	0	0.000	0	0.000	3	0.005	1	0.003
GG	0	0.000	0	0.000	2	0.004	0	0.000
HH	0	0.000	0	0.000	0	0.000	0	0.000
II	0	0.000	0	0.000	0	0.000	0	0.000
	559	1.000	437	1.000	571	1.000	308	1.000

Supplemental Table 1. Continued

	2008		2009		2010		2011	
Haplotype	#	FREQUENCY	#	FREQUENCY	#	FREQUENCY	#	FREQUENCY
A	42	0.070	30	0.058	15	0.031	20	0.043
B	28	0.048	60	0.117	37	0.077	32	0.068
C	13	0.022	8	0.016	18	0.038	13	0.028
D	2	0.003	0	0.000	0	0.000	0	0.000
E	358	0.625	270	0.524	301	0.630	290	0.618
F	10	0.017	9	0.017	8	0.017	7	0.015
G	3	0.005	15	0.029	4	0.008	5	0.011
H	5	0.008	2	0.004	9	0.019	1	0.002
I	2	0.003	6	0.012	1	0.002	6	0.013
J	6	0.012	4	0.008	5	0.010	4	0.009
K	4	0.007	4	0.008	0	0.000	4	0.009
L	0	0.000	2	0.004	0	0.000	0	0.000
M	0	0.000	1	0.002	1	0.002	5	0.011
N	0	0.000	0	0.000	0	0.000	0	0.000
O	1	0.002	0	0.000	0	0.000	0	0.000
P	12	0.020	6	0.012	2	0.004	13	0.028
Q	0	0.000	2	0.004	5	0.010	2	0.004
R	38	0.070	48	0.093	33	0.069	30	0.064
S	39	0.066	36	0.070	27	0.056	25	0.053
T	0	0.000	0	0.000	0	0.000	0	0.000
U	1	0.002	0	0.000	0	0.000	3	0.006
V	5	0.008	1	0.002	2	0.004	3	0.006
W	0	0.000	0	0.000	0	0.000	0	0.000
X	0	0.000	0	0.000	0	0.000	0	0.000
Y	0	0.000	0	0.000	0	0.000	0	0.000
Z	3	0.005	8	0.016	0	0.000	0	0.000
AA	0	0.000	0	0.000	0	0.000	0	0.000
BB	2	0.003	2	0.004	5	0.010	2	0.004
CC	0	0.002	1	0.002	1	0.002	2	0.004
DD	0	0.000	0	0.000	0	0.000	0	0.000
EE	0	0.000	0	0.000	0	0.000	0	0.000
FF	0	0.000	0	0.000	0	0.000	0	0.000
GG	0	0.000	0	0.000	2	0.004	0	0.000
HH	2	0.003	0	0.000	2	0.004	1	0.002
II	0	0.000	0	0.000	0	0.000	1	0.002
	576	1.000	515	1.000	478	1.000	469	1.000

Supplemental Table 2. MtDNA haplotypes for each individual. Collection # provides the abbreviated locality information and sample number. Sample # indicates the laboratory assigned identification.

Collection #	Sample #	Haplotype
NM13097.02	RBL97.002	S
NM13097.03	RBL97.003	J
NM13097.04	RBL97.004	B
NM13097.05	RBL97.005	E
NM13097.06	RBL97.006	E
NM13097.07	RBL97.007	E
NM13097.08	RBL97.008	K
NM13097.09	RBL97.009	F
NM13097.10	RBL97.010	E
NM13097.11	RBL97.011	E
NM13097.12	RBL97.012	E
NM13097.13	RBL97.013	F
NM13097.14	RBL97.014	E
NM13097.15	RBL97.015	E
NM13097.16	RBL97.016	F
NM13097.17	RBL97.017	E
NM13097.18	RBL97.018	E
NM13097.19	RBL97.019	E
NM13097.20	RBL97.020	F
NM13097.21	RBL97.021	F
NM13097.22	RBL97.022	J
NM13097.23	RBL97.023	E
NM13097.26	RBL97.026	E
HH3197.01	RBL97.027	E
HH3197.02	RBL97.028	E
HH3197.03	RBL97.029	E
HH3197.04	RBL97.030	B
HH3197.05	RBL97.031	B
HH3197.06	RBL97.032	E
HH3197.07	RBL97.033	B
HH3197.08	RBL97.034	K
HH3197.09	RBL97.035	C
HH3197.10	RBL97.036	B
HH3197.11	RBL97.037	E
HH3197.12	RBL97.038	E
HH3197.13	RBL97.039	E

Collection #	Sample #	Haplotype
TC3705.21	RBL05.230	E
TC3705.22	RBL05.231	E
TC3705.23	RBL05.232	E
TC3705.24	RBL05.233	E
TC3705.25	RBL05.234	E
TC3705.26	RBL05.235	E
TC3705.27	RBL05.236	E
TC3705.28	RBL05.237	E
TC32105.01	RBL05.238	E
TC32105.02	RBL05.239	E
TC32105.03	RBL05.240	A
TC32105.04	RBL05.241	E
TC32105.05	RBL05.242	E
TC32105.06	RBL05.243	E
TC32105.07	RBL05.244	E
TC32105.08	RBL05.245	E
TC32105.09	RBL05.246	E
TC32105.10	RBL05.247	E
TC32105.11	RBL05.248	A
TC32105.12	RBL05.249	A
TC32105.13	RBL05.250	J
TC32105.14	RBL05.251	A
TC32105.15	RBL05.252	C
TC32105.16	RBL05.253	E
TC32105.17	RBL05.254	H
TC32105.18	RBL05.255	S
TC32105.19	RBL05.256	E
TC32105.20	RBL05.257	E
TC32105.21	RBL05.258	J
TC32105.22	RBL05.259	E
TC32105.23	RBL05.260	E
TC32105.24	RBL05.261	CC
TC32105.25	RBL05.262	J
TC32105.26	RBL05.263	E
TC4505.01	RBL05.264	R
TC4505.02	RBL05.265	E

HH3197.14	RBL97.040	B	TC4505.03	RBL05.266	E
HH3197.15	RBL97.041	E	TC4505.04	RBL05.267	E
HH3197.16	RBL97.042	A	TC4505.05	RBL05.268	E
HH3197.17	RBL97.043	E	TC4505.06	RBL05.269	R
HH3197.18	RBL97.044	B	TC4505.07	RBL05.270	E
HH3197.19	RBL97.045	B	TC4505.08	RBL05.271	E
HH3197.20	RBL97.046	E	TC4505.09	RBL05.272	E
HH3197.21	RBL97.047	E	TC4505.10	RBL05.273	R
HH3197.22	RBL97.048	R	TC4505.11	RBL05.274	E
HH3197.23	RBL97.049	E	TC4505.12	RBL05.275	E
HH3197.24	RBL97.050	E	TC4505.13	RBL05.276	E
HH3197.25	RBL97.051	B	TC4505.14	RBL05.277	E
HH3197.26	RBL97.052	E	TC4505.15	RBL05.278	R
HH3197.27	RBL97.053	E	TC4505.16	RBL05.279	E
HH3197.28	RBL97.054	E	TC4505.17	RBL05.280	E
HH3197.29	RBL97.055	E	TC4505.18	RBL05.281	P
HH3197.30	RBL97.056	E	TC4505.19	RBL05.282	E
HF32997.01	RBL97.057	E	TC4505.20	RBL05.283	E
HF32997.02	RBL97.058	B	TC4505.21	RBL05.284	E
HF32997.03	RBL97.059	S	TC4505.22	RBL05.285	E
HF32997.04	RBL97.060	F	TC4505.24	RBL05.287	E
HF32997.05	RBL97.061	B	TC4505.25	RBL05.288	E
HF32997.06	RBL97.062	E	YC12505.01	RBL05.289	R
HF32997.07	RBL97.063	E	YC12505.02	RBL05.290	E
HF32997.08	RBL97.064	E	YC12505.03	RBL05.291	E
HF32997.09	RBL97.065	P	YC12505.04	RBL05.292	E
HF32997.10	RBL97.066	E	YC12505.05	RBL05.293	R
HF32997.11	RBL97.067	E	YC12505.06	RBL05.294	E
HF32997.12	RBL97.068	L	YC12505.07	RBL05.295	S
HF32997.13	RBL97.069	E	YC12505.08	RBL05.296	E
HF32997.14	RBL97.070	E	YC12505.09	RBL05.297	E
HF32997.15	RBL97.071	E	YC12505.10	RBL05.298	E
HF32997.16	RBL97.072	S	YC12505.11	RBL05.299	E
HF32997.17	RBL97.073	E	YC12505.12	RBL05.300	B
HF32997.18	RBL97.074	E	YC12505.13	RBL05.301	B
HF32997.19	RBL97.075	C	YC12505.14	RBL05.302	H
HF32997.20	RBL97.076	E	YC12505.15	RBL05.303	E
HF32997.21	RBL97.077	E	YC12505.16	RBL05.304	E
HF32997.22	RBL97.078	C	YC12505.17	RBL05.305	B
HF32997.23	RBL97.079	F	YC12505.18	RBL05.306	F
HF32997.24	RBL97.080	E	YC12505.19	RBL05.307	E

HF32997.25	RBL97.081	E	YC12505.20	RBL05.308	E
HF32997.26	RBL97.082	E	YC12505.21	RBL05.309	E
HF32997.27	RBL97.083	S	YC12505.22	RBL05.310	B
HF32997.28	RBL97.084	E	YC12505.23	RBL05.311	B
HF32997.29	RBL97.085	F	YC12505.24	RBL05.312	E
HF32997.30	RBL97.086	E	YC12505.25	RBL05.313	J
TC12897.01	RBL97.087	E	YC12505.26	RBL05.314	B
TC12897.02	RBL97.088	E	YC2805.01	RBL05.315	E
TC12897.03	RBL97.089	F	YC2805.02	RBL05.316	B
TC12897.06	RBL97.092	E	YC2805.03	RBL05.317	E
TC12897.08	RBL97.094	E	YC2805.04	RBL05.318	E
TC12897.09	RBL97.095	F	YC2805.05	RBL05.319	E
TC12897.10	RBL97.096	E	YC2805.06	RBL05.320	S
TC12897.12	RBL97.098	J	YC2805.07	RBL05.321	B
TC12897.13	RBL97.099	E	YC2805.08	RBL05.322	R
TC12897.15	RBL97.101	E	YC2805.09	RBL05.323	B
TC12897.16	RBL97.102	F	YC2805.10	RBL05.324	E
TC12897.18	RBL97.104	K	YC2805.11	RBL05.325	E
TC12897.19	RBL97.105	E	YC2805.12	RBL05.326	E
TC12897.20	RBL97.106	J	YC2805.13	RBL05.327	E
TC12897.21	RBL97.107	E	YC2805.14	RBL05.328	S
TC12897.22	RBL97.108	E	YC2805.15	RBL05.329	E
TC12897.23	RBL97.109	F	YC2805.16	RBL05.330	E
TC3197.01	RBL97.110	E	YC2805.17	RBL05.331	S
TC3197.02	RBL97.111	H	YC2805.18	RBL05.332	B
TC3197.03	RBL97.112	E	YC2805.19	RBL05.333	E
TC3197.04	RBL97.113	E	YC2805.20	RBL05.334	B
TC3197.05	RBL97.114	E	YC2805.21	RBL05.335	E
TC3197.06	RBL97.115	E	YC2805.22	RBL05.336	E
TC3197.07	RBL97.116	E	YC2805.23	RBL05.337	E
TC3197.08	RBL97.117	E	YC2805.24	RBL05.338	B
TC3197.09	RBL97.118	E	YC2805.25	RBL05.339	E
TC3197.10	RBL97.119	E	YC2805.26	RBL05.340	B
TC3197.11	RBL97.120	E	YC22305.01	RBL05.341	P
TC3197.12	RBL97.121	E	YC22305.02	RBL05.342	B
TC3197.13	RBL97.122	B	YC22305.03	RBL05.343	E
TC3197.14	RBL97.123	R	YC22305.04	RBL05.344	E
TC3197.15	RBL97.124	P	YC22305.05	RBL05.345	E
TC3197.16	RBL97.125	E	YC22305.06	RBL05.346	E
TC3197.17	RBL97.126	E	YC22305.07	RBL05.347	B
TC3197.18	RBL97.127	E	YC22305.08	RBL05.348	E

TC3197.19	RBL97.128	R	YC22305.09	RBL05.349	E
TC3197.20	RBL97.129	E	YC22305.10	RBL05.350	E
TC3197.21	RBL97.130	R	YC22305.11	RBL05.351	E
TC3197.22	RBL97.131	O	YC22305.12	RBL05.352	E
TC3197.23	RBL97.132	A	YC22305.13	RBL05.353	E
TC3197.24	RBL97.133	E	YC22305.14	RBL05.354	E
TC3197.25	RBL97.134	B	YC22305.15	RBL05.355	K
TC3197.26	RBL97.135	S	YC22305.16	RBL05.356	R
TC3197.27	RBL97.136	E	YC22305.17	RBL05.357	E
TC3197.28	RBL97.137	B	YC22305.18	RBL05.358	E
TC3197.29	RBL97.138	E	YC22305.19	RBL05.359	B
TC3197.30	RBL97.139	E	YC22305.20	RBL05.360	F
TC3197.31	RBL97.140	E	YC22305.21	RBL05.361	E
TC32997.01	RBL97.141	R	YC22305.22	RBL05.362	E
TC32997.02	RBL97.142	E	YC22305.23	RBL05.363	E
TC32997.03	RBL97.143	E	YC22305.24	RBL05.364	V
TC32997.04	RBL97.144	B	YC22305.25	RBL05.365	R
TC32997.05	RBL97.145	E	YC3805.01	RBL05.366	E
TC32997.06	RBL97.146	E	YC3805.02	RBL05.367	R
TC32997.07	RBL97.147	R	YC3805.03	RBL05.368	A
TC32997.08	RBL97.148	E	YC3805.04	RBL05.369	R
TC32997.09	RBL97.149	C	YC3805.05	RBL05.370	R
TC32997.10	RBL97.150	C	YC3805.06	RBL05.371	E
TC32997.11	RBL97.151	E	YC3805.07	RBL05.372	A
TC32997.12	RBL97.152	E	YC3805.08	RBL05.373	R
TC32997.14	RBL97.154	E	YC3805.09	RBL05.374	A
TC32997.15	RBL97.155	C	YC3805.10	RBL05.375	F
TC32997.16	RBL97.156	E	YC3805.11	RBL05.376	E
TC32997.17	RBL97.157	R	YC3805.12	RBL05.377	E
TC32997.19	RBL97.159	R	YC3805.13	RBL05.378	E
TC32997.20	RBL97.160	E	YC3805.14	RBL05.379	B
TC32997.21	RBL97.161	R	YC3805.15	RBL05.380	E
TC32997.22	RBL97.162	R	YC3805.16	RBL05.381	E
TC32997.23	RBL97.163	E	YC3805.17	RBL05.382	R
TC32997.24	RBL97.164	N	YC3805.18	RBL05.383	E
TC32997.25	RBL97.165	E	YC3805.19	RBL05.384	E
TC32997.26	RBL97.166	E	YC3805.20	RBL05.385	E
TC32997.27	RBL97.167	C	YC3805.21	RBL05.386	E
TC32997.28	RBL97.168	E	YC3805.22	RBL05.387	E
TC32997.29	RBL97.169	C	YC3805.23	RBL05.388	E
TC32997.30	RBL97.170	E	YC3805.24	RBL05.389	B

RTC33197.01	RBL97.171	E	YC3805.25	RBL05.390	E
RTC33197.02	RBL97.172	E	YC32205.01	RBL05.391	E
RTC33197.03	RBL97.173	E	YC32205.02	RBL05.392	E
RTC33197.04	RBL97.174	E	YC32205.03	RBL05.393	E
RTC33197.05	RBL97.175	P	YC32205.04	RBL05.394	E
RTC33197.07	RBL97.177	E	YC32205.05	RBL05.395	R
RTC33197.08	RBL97.178	E	YC32205.06	RBL05.396	A
RTC33197.09	RBL97.179	E	YC32205.07	RBL05.397	V
RTC33197.10	RBL97.180	E	YC32205.08	RBL05.398	E
RTC33197.11	RBL97.181	E	YC32205.09	RBL05.399	E
RTC33197.12	RBL97.182	P	YC32205.10	RBL05.400	E
RTC33197.14	RBL97.184	M	YC32205.11	RBL05.401	E
RTC33197.15	RBL97.185	B	YC32205.12	RBL05.402	A
RTC33197.16	RBL97.186	M	YC32205.13	RBL05.403	H
RTC33197.17	RBL97.187	A	YC32205.14	RBL05.404	E
RTC33197.18	RBL97.188	E	YC32205.15	RBL05.405	E
RTC33197.19	RBL97.189	E	YC32205.16	RBL05.406	A
RTC33197.21	RBL97.191	P	YC32205.17	RBL05.407	V
RTC33197.23	RBL97.193	M	YC32205.18	RBL05.408	R
RTC33197.24	RBL97.194	E	YC32205.19	RBL05.409	R
RTC33197.25	RBL97.195	E	YC32205.20	RBL05.410	E
RTC33197.26	RBL97.196	A	YC32205.21	RBL05.411	E
RTC33197.27	RBL97.197	P	YC32205.22	RBL05.412	E
RTC33197.28	RBL97.198	E	YC32205.23	RBL05.413	B
RTC33197.29	RBL97.199	E	YC32205.24	RBL05.414	R
RTC33197.30	RBL97.200	P	YC32205.25	RBL05.415	E
RTC33197.31	RBL97.201	E	YC4505.01	RBL05.416	E
WC32497.02	RBL97.203	J	YC4505.02	RBL05.417	E
WC32497.03	RBL97.204	B	YC4505.03	RBL05.418	M
WC32497.04	RBL97.205	B	YC4505.04	RBL05.419	E
WC32497.05	RBL97.206	B	YC4505.05	RBL05.420	A
WC32497.06	RBL97.207	B	YC4505.06	RBL05.421	E
WC32497.07	RBL97.208	E	YC4505.07	RBL05.422	E
WC32497.08	RBL97.209	B	YC4505.08	RBL05.423	E
WC32497.10	RBL97.211	B	YC4505.09	RBL05.424	E
WC32497.11	RBL97.212	B	YC4505.10	RBL05.425	A
WC32497.12	RBL97.213	B	YC4505.11	RBL05.426	A
WC32497.13	RBL97.214	B	YC4505.12	RBL05.427	A
WC32497.14	RBL97.215	E	YC4505.13	RBL05.428	E
WC32497.15	RBL97.216	J	YC4505.14	RBL05.429	F
WC32497.17	RBL97.218	B	YC4505.15	RBL05.430	E

WC32497.18	RBL97.219	B	YC4505.16	RBL05.431	E
WC32497.19	RBL97.220	B	YC4505.17	RBL05.432	R
WC32497.20	RBL97.221	J	YC4505.18	RBL05.433	E
WC32497.22	RBL97.223	B	YC4505.20	RBL05.435	S
WC32497.23	RBL97.224	R	YC4505.21	RBL05.436	A
WC32497.24	RBL97.225	B	YC4505.22	RBL05.437	E
WC32497.25	RBL97.226	B	YC4505.23	RBL05.438	E
WC4197.01	RBL97.227	E	YC4505.24	RBL05.439	E
WC4197.02	RBL97.228	E	YC4505.25	RBL05.440	E
WC4197.03	RBL97.229	E	RT032206.01	RBL06.028	E
WC4197.05	RBL97.231	E	RT032206.02	RBL06.029	E
WC4197.06	RBL97.232	E	RT032206.03	RBL06.030	E
WC4197.07	RBL97.233	E	RT032206.04	RBL06.031	E
WC4197.08	RBL97.234	E	RT032206.05	RBL06.032	J
WC4197.09	RBL97.235	E	RT032206.06	RBL06.033	EE
WC4197.11	RBL97.237	E	RT032206.07	RBL06.034	E
WC4197.12	RBL97.238	E	RT032206.08	RBL06.035	E
WC4197.13	RBL97.239	E	RT032206.09	RBL06.036	E
WC4197.15	RBL97.241	E	RT032206.10	RBL06.037	S
WC4197.17	RBL97.243	E	RT032206.11	RBL06.038	E
WC4197.18	RBL97.244	R	RT032206.12	RBL06.039	E
WC4197.19	RBL97.245	E	RT032206.13	RBL06.040	B
WC4197.20	RBL97.246	B	RT032206.14	RBL06.041	E
WC4197.21	RBL97.247	E	RT032206.15	RBL06.042	B
WC4197.22	RBL97.248	B	RT032206.16	RBL06.043	E
WC4197.23	RBL97.249	B	RT032206.17	RBL06.044	R
WC4197.24	RBL97.250	A	RT032206.18	RBL06.045	B
WC4197.25	RBL97.251	E	RT032206.19	RBL06.046	E
WC4197.26	RBL97.252	E	RT032206.20	RBL06.047	E
WC4197.27	RBL97.253	E	RT032206.21	RBL06.048	E
WC4197.28	RBL97.254	E	RT032206.22	RBL06.049	E
WC4197.29	RBL97.255	C	RT032206.23	RBL06.050	B
WC4197.30	RBL97.256	C	RT032206.24	RBL06.051	S
YC2397.01	RBL97.257	R	HH020706.01	RBL06.052	E
YC2397.02	RBL97.258	E	HH020706.02	RBL06.053	G
YC2397.04	RBL97.260	S	HH020706.03	RBL06.054	E
YC2397.05	RBL97.261	B	HH020706.04	RBL06.055	CC
YC2397.06	RBL97.262	C	HH020706.05	RBL06.056	E
YC2397.09	RBL97.265	A	HH020706.06	RBL06.057	B
YC2397.10	RBL97.266	A	HH020706.07	RBL06.058	E
YC2397.11	RBL97.267	E	HH020706.08	RBL06.059	R

YC2397.12	RBL97.268	E	HH020706.09	RBL06.060	E
YC2397.13	RBL97.269	C	HH020706.10	RBL06.061	f
YC2397.15	RBL97.271	H	HH020706.11	RBL06.062	C
YC2397.17	RBL97.273	B	HH020706.12	RBL06.063	G
YC2397.19	RBL97.275	B	HH020706.13	RBL06.064	F
YC2397.21	RBL97.277	E	HH020706.14	RBL06.065	F
YC2397.22	RBL97.278	E	HH020706.15	RBL06.066	E
YC2397.23	RBL97.279	E	HH020706.16	RBL06.067	G
YC2397.24	RBL97.280	E	HH020706.17	RBL06.068	G
YC22897.01	RBL97.282	C	HH020706.18	RBL06.069	E
YC22897.02	RBL97.283	E	HH020706.19	RBL06.070	H
YC22897.03	RBL97.284	R	HH020706.20	RBL06.071	E
YC22897.04	RBL97.285	B	HH020706.21	RBL06.072	E
YC22897.05	RBL97.286	B	HH020706.22	RBL06.073	H
YC22897.06	RBL97.287	E	HH020706.23	RBL06.074	H
YC22897.08	RBL97.289	C	HH020706.24	RBL06.075	H
YC22897.09	RBL97.290	R	HH020706.25	RBL06.076	A
YC22897.10	RBL97.291	E	HH022306.01	RBL06.077	A
YC22897.11	RBL97.292	E	HH022306.02	RBL06.078	E
YC22897.13	RBL97.294	C	HH022306.03	RBL06.079	E
YC22897.14	RBL97.295	B	HH022306.04	RBL06.080	E
YC22897.15	RBL97.296	B	HH022306.05	RBL06.081	E
YC22897.16	RBL97.297	E	HH022306.06	RBL06.082	M
YC22897.17	RBL97.298	E	HH022306.07	RBL06.083	F
YC22897.18	RBL97.299	A	HH022306.08	RBL06.084	E
YC22897.19	RBL97.300	R	HH022306.09	RBL06.085	R
YC22897.20	RBL97.301	Q	HH022306.10	RBL06.086	R
YC22897.21	RBL97.302	E	HH022306.11	RBL06.087	E
YC22897.22	RBL97.303	E	HH022306.12	RBL06.088	E
YC22897.23	RBL97.304	A	HH022306.13	RBL06.089	S
YC22897.24	RBL97.305	E	HH022306.14	RBL06.090	E
YC22897.25	RBL97.306	E	HH022306.15	RBL06.091	B
YC22897.26	RBL97.307	E	HH022306.16	RBL06.092	B
YC22897.27	RBL97.308	E	HH022306.17	RBL06.093	E
YC22897.28	RBL97.309	E	HH022306.18	RBL06.094	E
YC22897.29	RBL97.310	C	HH022306.19	RBL06.095	B
YC22897.30	RBL97.311	E	HH022306.20	RBL06.096	E
YC22897.31	RBL97.312	B	HH022306.21	RBL06.097	Z
YC32997.01	RBL97.313	E	HH022306.22	RBL06.098	B
YC32997.02	RBL97.314	E	HH022306.23	RBL06.099	B
YC32997.03	RBL97.315	E	HH022306.24	RBL06.100	E

YC32997.04	RBL97.316	C	HH022306.25	RBL06.101	B
YC32997.05	RBL97.317	E	HF030706.01	RBL06.102	E
YC32997.06	RBL97.318	E	HF030706.02	RBL06.103	E
YC32997.07	RBL97.319	E	HF030706.03	RBL06.104	S
YC32997.08	RBL97.320	E	HF030706.04	RBL06.105	E
YC32997.09	RBL97.321	B	HF030706.05	RBL06.106	E
YC32997.10	RBL97.322	E	HF030706.06	RBL06.107	E
YC32997.11	RBL97.323	E	HF030706.07	RBL06.108	A
YC32997.12	RBL97.324	E	HF030706.08	RBL06.109	E
YC32997.13	RBL97.325	E	HF030706.09	RBL06.110	FF
YC32997.14	RBL97.326	E	HF030706.10	RBL06.111	E
YC32997.15	RBL97.327	E	HF030706.11	RBL06.112	E
YC32997.16	RBL97.328	E	HF030706.12	RBL06.113	FF
YC32997.17	RBL97.329	E	HF030706.13	RBL06.114	E
YC32997.18	RBL97.330	E	HF030706.14	RBL06.115	E
YC32997.19	RBL97.331	E	HF030706.15	RBL06.116	E
YC32997.20	RBL97.332	E	HF030706.16	RBL06.117	E
YC32997.21	RBL97.333	E	HF030706.17	RBL06.118	E
YC32997.22	RBL97.334	E	HF030706.18	RBL06.119	B
YC32997.23	RBL97.335	E	HF030706.19	RBL06.120	E
YC32997.24	RBL97.336	R	HF030706.20	RBL06.121	E
YC32997.25	RBL97.337	G	HF030706.21	RBL06.122	FF
YC32997.26	RBL97.338	C	HF030706.22	RBL06.123	E
YC32997.27	RBL97.339	B	HF030706.23	RBL06.124	E
YC32997.28	RBL97.340	E	HF030706.24	RBL06.125	E
YC32997.29	RBL97.341	E	HF030706.25	RBL06.126	S
YC32997.30	RBL97.342	E	NM031506.01	RBL06.127	E
LC32997.01	RBL97.343	E	NM031506.02	RBL06.128	E
LC32997.02	RBL97.344	E	NM031506.03	RBL06.129	E
LC32997.03	RBL97.345	E	NM031506.04	RBL06.130	E
LC32997.04	RBL97.346	E	NM031506.05	RBL06.131	E
LC32997.05	RBL97.347	E	NM031506.06	RBL06.132	E
LC32997.06	RBL97.348	B	NM031506.07	RBL06.133	E
LC32997.07	RBL97.349	E	NM031506.08	RBL06.134	E
LC32997.08	RBL97.350	E	NM031506.09	RBL06.135	E
LC32997.09	RBL97.351	E	NM031506.10	RBL06.136	E
LC32997.10	RBL97.352	E	NM031506.11	RBL06.137	S
LC32997.11	RBL97.353	E	NM031506.12	RBL06.138	E
LC32997.12	RBL97.354	B	NM031506.13	RBL06.139	R
LC32997.13	RBL97.355	E	NM031506.14	RBL06.140	S
LC32997.14	RBL97.356	L	NM031506.15	RBL06.141	E

LC32997.15	RBL97.357	E	NM031506.16	RBL06.142	R
LC32997.16	RBL97.358	E	NM031506.17	RBL06.143	E
LC32997.17	RBL97.359	P	NM031506.18	RBL06.144	E
LC32997.18	RBL97.360	E	NM031506.19	RBL06.145	E
LC32997.19	RBL97.361	E	NM031506.20	RBL06.146	E
LC32997.20	RBL97.362	E	NM031506.21	RBL06.147	R
LC32997.21	RBL97.363	I	NM031506.22	RBL06.148	S
LC32997.22	RBL97.364	B	NM031506.23	RBL06.149	S
LC32997.23	RBL97.365	E	NM031506.24	RBL06.150	E
LC32997.24	RBL97.366	F	NM031506.25	RBL06.151	E
LC32997.26	RBL97.368	E	NM031506.26	RBL06.152	E
LC32997.27	RBL97.369	R	HH032106.01	RBL06.153	E
LC32997.28	RBL97.370	E	HH032106.02	RBL06.154	E
LC32997.29	RBL97.371	E	HH032106.03	RBL06.155	E
LC32997.30	RBL97.372	E	HH032106.04	RBL06.156	E
HH21298.01	RBL98.001	H	HH032106.05	RBL06.157	E
HH21298.02	RBL98.002	M	HH032106.06	RBL06.158	E
HH21298.03	RBL98.003	E	HH032106.07	RBL06.159	E
HH21298.04	RBL98.004	R	HH032106.08	RBL06.160	E
HH21298.05	RBL98.005	E	HH032106.09	RBL06.161	E
HH21298.06	RBL98.006	E	HH032106.10	RBL06.162	E
HH21298.07	RBL98.007	J	HH032106.11	RBL06.163	E
HH21298.08	RBL98.008	E	HH032106.12	RBL06.164	E
HH21298.09	RBL98.009	J	HH032106.13	RBL06.165	B
HH21298.10	RBL98.010	E	HH032106.14	RBL06.166	F
HH21298.11	RBL98.011	E	HH032106.15	RBL06.167	E
HH21298.12	RBL98.012	K	HH032106.16	RBL06.168	E
HH21298.13	RBL98.013	B	HH032106.17	RBL06.169	E
HH21298.14	RBL98.014	S	HH032106.18	RBL06.170	C
HH21298.15	RBL98.015	E	HH032106.19	RBL06.171	E
HH21298.16	RBL98.016	E	HH032106.20	RBL06.172	E
HH21298.17	RBL98.017	E	HH032106.21	RBL06.173	E
HH21298.18	RBL98.018	F	HH032106.22	RBL06.174	F
HH21298.19	RBL98.019	R	HH032106.23	RBL06.175	Z
HH21298.20	RBL98.020	E	HH032106.24	RBL06.176	A
HH21298.21	RBL98.021	R	HH032106.25	RBL06.177	E
HH21298.22	RBL98.022	E	HF040606.01	RBL06.178	S
HH21298.23	RBL98.023	E	HF040606.02	RBL06.179	E
HH21298.24	RBL98.024	E	HF040606.03	RBL06.180	S
HH21298.25	RBL98.025	E	HF040606.04	RBL06.181	E
HH21298.26	RBL98.026	E	HF040606.05	RBL06.182	E

HH21298.27	RBL98.027	E	HF040606.06	RBL06.183	E
HH21298.28	RBL98.028	E	HF040606.07	RBL06.184	E
HH21298.29	RBL98.029	E	HF040606.08	RBL06.185	E
HH21298.30	RBL98.030	E	HF040606.09	RBL06.186	C
HH22798.01	RBL98.031	E	HF040606.10	RBL06.187	E
HH22798.02	RBL98.032	E	HF040606.11	RBL06.188	R
HH22798.03	RBL98.033	E	HF040606.12	RBL06.189	S
HH22798.04	RBL98.034	E	HF040606.13	RBL06.190	E
HH22798.05	RBL98.035	R	HF040606.14	RBL06.191	A
HH22798.06	RBL98.036	E	HF040606.15	RBL06.192	E
HH22798.07	RBL98.037	E	HF040606.16	RBL06.193	E
HH22798.08	RBL98.038	V	HF040606.17	RBL06.194	E
HH22798.09	RBL98.039	E	HF040606.18	RBL06.195	E
HH22798.10	RBL98.040	G	HF040606.19	RBL06.196	E
HH22798.11	RBL98.041	S	HF040606.20	RBL06.197	E
HH22798.12	RBL98.042	S	HF040606.21	RBL06.198	C
HH22798.13	RBL98.043	E	HF040606.22	RBL06.199	C
HH22798.14	RBL98.044	E	HF040606.23	RBL06.200	E
HH22798.15	RBL98.045	Z	HF040606.24	RBL06.201	E
HH22798.16	RBL98.046	E	HF040606.25	RBL06.202	E
HH22798.17	RBL98.047	E	NWC040406.01	RBL06.203	J
HH22798.18	RBL98.048	E	NWC040406.02	RBL06.204	A
HH22798.19	RBL98.049	E	NWC040406.03	RBL06.205	E
HH22798.20	RBL98.050	E	NWC040406.04	RBL06.206	E
HH22798.21	RBL98.051	E	NWC040406.05	RBL06.207	B
HH22798.22	RBL98.052	E	NWC040406.06	RBL06.208	S
HH22798.23	RBL98.053	E	NWC040406.07	RBL06.209	B
HH22798.24	RBL98.054	F	NWC040406.08	RBL06.210	F
HH22798.25	RBL98.055	E	NWC040406.09	RBL06.211	B
HH22798.26	RBL98.056	E	NWC040406.10	RBL06.212	E
HH22798.27	RBL98.057	E	NWC040406.11	RBL06.213	E
HH22798.28	RBL98.058	E	NWC040406.12	RBL06.214	B
HH22798.29	RBL98.059	E	NWC040406.13	RBL06.215	E
HH22798.30	RBL98.060	M	NWC040406.14	RBL06.216	M
HH31798.01	RBL98.061	E	NWC040406.15	RBL06.217	M
HH31798.02	RBL98.062	E	NWC040406.16	RBL06.218	S
HH31798.03	RBL98.063	S	NWC040406.17	RBL06.219	E
HH31798.04	RBL98.064	E	NWC040406.18	RBL06.220	F
HH31798.05	RBL98.065	C	NWC040406.19	RBL06.221	S
HH31798.06	RBL98.066	E	NWC040406.20	RBL06.222	E
HH31798.07	RBL98.067	E	NWC040406.21	RBL06.223	E

HH31798.08	RBL98.068	R	NWC040406.22	RBL06.224	E
HH31798.09	RBL98.069	E	LC042706.01	RBL06.225	E
HH31798.10	RBL98.070	F	LC042706.02	RBL06.226	E
HH31798.11	RBL98.071	B	LC042706.03	RBL06.227	E
HH31798.12	RBL98.072	E	LC042706.04	RBL06.228	E
HH31798.13	RBL98.073	E	LC042706.05	RBL06.229	E
HH31798.14	RBL98.074	E	LC042706.06	RBL06.230	E
HH31798.15	RBL98.075	E	LC042706.07	RBL06.231	E
HH31798.16	RBL98.076	R	LC042706.08	RBL06.232	E
HH31798.17	RBL98.077	F	LC042706.09	RBL06.233	E
HH31798.18	RBL98.078	F	LC042706.10	RBL06.234	E
HH31798.19	RBL98.079	B	LC042706.11	RBL06.235	E
HH31798.20	RBL98.080	E	LC042706.12	RBL06.236	E
HH31798.21	RBL98.081	E	LC042706.13	RBL06.237	E
HH31798.22	RBL98.082	E	LC042706.14	RBL06.238	R
HH31798.23	RBL98.083	E	LC042706.15	RBL06.239	E
HH31798.24	RBL98.084	I	LC042706.16	RBL06.240	E
HH31798.25	RBL98.085	E	LC042706.17	RBL06.241	E
HH4698.01	RBL98.086	A	LC042706.18	RBL06.242	E
HH4698.02	RBL98.087	E	LC042706.19	RBL06.243	E
HH4698.03	RBL98.088	S	LC042706.20	RBL06.244	E
HH4698.04	RBL98.089	E	LC042706.21	RBL06.245	BB
HH4698.05	RBL98.090	F	LC042706.22	RBL06.246	E
TC13198.01	RBL98.091	E	LC042706.23	RBL06.247	E
TC13198.02	RBL98.092	E	LC042706.24	RBL06.248	E
TC13198.03	RBL98.093	E	LC042706.25	RBL06.249	E
TC13198.04	RBL98.094	E	TC020806.01	RBL06.250	E
TC13198.05	RBL98.095	E	TC020806.02	RBL06.251	B
TC13198.06	RBL98.096	E	TC020806.03	RBL06.252	A
TC13198.07	RBL98.097	E	TC020806.04	RBL06.253	E
TC13198.08	RBL98.098	E	TC020806.05	RBL06.254	A
TC13198.09	RBL98.099	E	TC020806.06	RBL06.255	E
TC13198.10	RBL98.100	E	TC020806.07	RBL06.256	R
TC13198.11	RBL98.101	E	TC020806.08	RBL06.257	E
TC13198.12	RBL98.102	E	TC020806.09	RBL06.258	E
TC13198.13	RBL98.103	E	TC020806.10	RBL06.259	E
TC13198.14	RBL98.104	E	TC020806.11	RBL06.260	A
TC13198.15	RBL98.105	E	TC020806.12	RBL06.261	E
TC13198.16	RBL98.106	E	TC020806.13	RBL06.262	B
TC13198.17	RBL98.107	E	TC020806.14	RBL06.263	E
TC13198.19	RBL98.109	E	TC020806.15	RBL06.264	R

TC13198.20	RBL98.110	E	TC020806.16	RBL06.265	E
TC13198.21	RBL98.111	E	TC020806.17	RBL06.266	E
TC13198.22	RBL98.112	E	TC020806.18	RBL06.267	E
TC13198.23	RBL98.113	E	TC020806.19	RBL06.268	E
TC13198.24	RBL98.114	E	TC020806.20	RBL06.269	B
TC13198.25	RBL98.115	E	TC020806.21	RBL06.270	B
TC13198.26	RBL98.116	E	TC020806.22	RBL06.271	E
TC13198.27	RBL98.117	E	TC020806.23	RBL06.272	E
TC13198.28	RBL98.118	E	TC020806.24	RBL06.273	A
TC13198.29	RBL98.119	E	TC020806.25	RBL06.274	B
TC13198.30	RBL98.120	E	TC022106.01	RBL06.275	E
TC21298.01	RBL98.121	K	TC022106.02	RBL06.276	E
TC21298.02	RBL98.122	B	TC022106.03	RBL06.277	E
TC21298.03	RBL98.123	R	TC022106.04	RBL06.278	E
TC21298.04	RBL98.124	K	TC022106.05	RBL06.279	E
TC21298.05	RBL98.125	E	TC022106.06	RBL06.280	E
TC21298.06	RBL98.126	S	TC022106.07	RBL06.281	E
TC21298.07	RBL98.127	R	TC022106.08	RBL06.282	E
TC21298.08	RBL98.128	E	TC022106.09	RBL06.283	E
TC21298.09	RBL98.129	H	TC022106.10	RBL06.284	R
TC21298.10	RBL98.130	E	TC022106.11	RBL06.285	E
TC21298.11	RBL98.131	B	TC022106.12	RBL06.286	BB
TC21298.12	RBL98.132	E	TC022106.13	RBL06.287	B
TC21298.13	RBL98.133	K	TC022106.14	RBL06.288	B
TC21298.14	RBL98.134	R	TC022106.15	RBL06.289	B
TC21298.15	RBL98.135	R	TC022106.16	RBL06.290	E
TC21298.16	RBL98.136	F	TC022106.17	RBL06.291	BB
TC21298.17	RBL98.137	E	TC022106.18	RBL06.292	BB
TC21298.18	RBL98.138	E	TC022106.19	RBL06.293	B
TC21298.19	RBL98.139	E	TC022106.20	RBL06.294	BB
TC21298.20	RBL98.140	E	TC022106.21	RBL06.295	E
TC21298.21	RBL98.141	E	TC022106.22	RBL06.296	R
TC21298.22	RBL98.142	R	TC022106.23	RBL06.297	E
TC21298.23	RBL98.143	E	TC022106.24	RBL06.298	B
TC21298.24	RBL98.144	E	TC022106.25	RBL06.299	B
TC21298.25	RBL98.145	E	TC030706.01	RBL06.300	S
TC21298.26	RBL98.146	E	TC030706.02	RBL06.301	S
TC21298.27	RBL98.147	E	TC030706.03	RBL06.302	E
TC21298.28	RBL98.148	E	TC030706.04	RBL06.303	S
TC21298.29	RBL98.149	A	TC030706.05	RBL06.304	E
TC21298.30	RBL98.150	B	TC030706.06	RBL06.305	E

TC22798.01	RBL98.151	E	TC030706.07	RBL06.306	E
TC22798.02	RBL98.152	E	TC030706.08	RBL06.307	E
TC22798.03	RBL98.153	E	TC030706.09	RBL06.308	CC
TC22798.04	RBL98.154	E	TC030706.10	RBL06.309	E
TC22798.05	RBL98.155	E	TC030706.11	RBL06.310	GG
TC22798.06	RBL98.156	E	TC030706.12	RBL06.311	CC
TC22798.07	RBL98.157	E	TC030706.13	RBL06.312	S
TC22798.08	RBL98.158	E	TC030706.14	RBL06.313	S
TC22798.09	RBL98.159	E	TC030706.15	RBL06.314	CC
TC22798.10	RBL98.160	E	TC030706.16	RBL06.315	C
TC22798.11	RBL98.161	E	TC030706.17	RBL06.316	CC
TC22798.12	RBL98.162	E	TC030706.18	RBL06.317	R
TC22798.13	RBL98.163	E	TC030706.19	RBL06.318	E
TC22798.14	RBL98.164	E	TC030706.20	RBL06.319	E
TC22798.15	RBL98.165	E	TC030706.21	RBL06.320	GG
TC22798.16	RBL98.166	E	TC030706.22	RBL06.321	S
TC22798.17	RBL98.167	E	TC030706.23	RBL06.322	E
TC22798.18	RBL98.168	E	TC030706.24	RBL06.323	E
TC22798.19	RBL98.169	E	TC031506.01	RBL06.324	B
TC22798.20	RBL98.170	E	TC031506.02	RBL06.325	E
TC22798.21	RBL98.171	S	TC031506.03	RBL06.326	E
TC22798.22	RBL98.172	E	TC031506.04	RBL06.327	E
TC22798.23	RBL98.173	E	TC031506.05	RBL06.328	E
TC22798.24	RBL98.174	E	TC031506.06	RBL06.329	E
TC22798.25	RBL98.175	E	TC031506.07	RBL06.330	E
TC22798.26	RBL98.176	E	TC031506.08	RBL06.331	S
TC22798.27	RBL98.177	E	TC031506.09	RBL06.332	E
TC22798.28	RBL98.178	S	TC031506.10	RBL06.333	E
TC22798.29	RBL98.179	E	TC031506.11	RBL06.334	D
TC22798.30	RBL98.180	E	TC031506.12	RBL06.335	R
TC31798.01	RBL98.181	A	TC031506.13	RBL06.336	R
TC31798.02	RBL98.182	E	TC031506.14	RBL06.337	E
TC31798.03	RBL98.183	E	TC031506.16	RBL06.339	E
TC31798.04	RBL98.184	E	TC031506.18	RBL06.341	E
TC31798.05	RBL98.185	J	TC031506.19	RBL06.342	K
TC31798.06	RBL98.186	E	TC031506.20	RBL06.343	B
TC31798.07	RBL98.187	BB	TC031506.22	RBL06.345	E
TC31798.08	RBL98.188	E	TC031506.23	RBL06.346	E
TC31798.09	RBL98.189	E	TC031506.24	RBL06.347	E
TC31798.10	RBL98.190	A	TC031506.25	RBL06.348	E
TC31798.11	RBL98.191	E	TC031506.26	RBL06.349	B

TC31798.12	RBL98.192	E	TC032206.01	RBL06.350	E
TC31798.13	RBL98.193	F	TC032206.02	RBL06.351	A
TC31798.14	RBL98.194	B	TC032206.03	RBL06.352	E
TC31798.15	RBL98.195	P	TC032206.04	RBL06.353	E
TC31798.16	RBL98.196	E	TC032206.05	RBL06.354	E
TC31798.17	RBL98.197	B	TC032206.06	RBL06.355	E
TC31798.18	RBL98.198	E	TC032206.07	RBL06.356	E
TC31798.19	RBL98.199	E	TC032206.08	RBL06.357	E
TC31798.20	RBL98.200	E	TC032206.09	RBL06.358	E
TC31798.21	RBL98.201	E	TC032206.10	RBL06.359	E
TC31798.22	RBL98.202	F	TC032206.11	RBL06.360	S
TC31798.23	RBL98.203	E	TC032206.12	RBL06.361	E
TC31798.24	RBL98.204	E	TC032206.13	RBL06.362	E
TC31798.25	RBL98.205	B	TC032206.14	RBL06.363	E
TC31798.26	RBL98.206	E	TC032206.15	RBL06.364	R
TC31798.27	RBL98.207	E	TC032206.16	RBL06.365	R
TC31798.28	RBL98.208	E	TC032206.17	RBL06.366	CC
TC31798.29	RBL98.209	E	TC032206.18	RBL06.367	E
TC31798.30	RBL98.210	E	TC032206.19	RBL06.368	E
TC31798.31	RBL98.211	B	TC032206.20	RBL06.369	E
TC4698.01	RBL98.212	E	TC032206.21	RBL06.370	E
TC4698.02	RBL98.213	B	TC032206.22	RBL06.371	S
TC4698.03	RBL98.214	E	TC032206.23	RBL06.372	E
TC4698.04	RBL98.215	F	TC032206.24	RBL06.373	E
TC4698.05	RBL98.216	E	TC032206.25	RBL06.374	E
TC4698.06	RBL98.217	E	TC040506.01	RBL06.375	E
TC4698.07	RBL98.218	S	TC040506.02	RBL06.376	E
TC4698.08	RBL98.219	E	TC040506.03	RBL06.377	B
TC4698.09	RBL98.220	S	TC040506.05	RBL06.379	E
TC4698.10	RBL98.221	E	TC040506.06	RBL06.380	E
TC4698.11	RBL98.222	E	TC040506.07	RBL06.381	A
TC4698.12	RBL98.223	E	TC040506.08	RBL06.382	E
TC4698.13	RBL98.224	S	TC040506.09	RBL06.383	E
TC4698.14	RBL98.225	P	TC040506.10	RBL06.384	E
TC4698.15	RBL98.226	F	TC040506.11	RBL06.385	E
TC4698.16	RBL98.227	E	TC040506.12	RBL06.386	R
TC4698.17	RBL98.228	E	TC040506.13	RBL06.387	E
TC4698.18	RBL98.229	R	TC040506.14	RBL06.388	E
TC4698.20	RBL98.231	E	TC040506.15	RBL06.389	E
TC4698.21	RBL98.232	E	TC040506.16	RBL06.390	E
TC4698.22	RBL98.233	P	TC040506.17	RBL06.391	E

TC4698.23	RBL98.234	E	TC040506.18	RBL06.392	R
TC4698.24	RBL98.235	B	TC040506.19	RBL06.393	A
TC4698.25	RBL98.236	Q	TC040506.20	RBL06.394	E
WC13198.01	RBL98.237	C	TC040506.21	RBL06.395	E
WC13198.02	RBL98.238	F	TC040506.22	RBL06.396	E
WC13198.03	RBL98.239	R	TC040506.23	RBL06.397	E
WC21298.01	RBL98.240	R	TC040506.24	RBL06.398	B
WC21298.02	RBL98.241	E	TC040506.25	RBL06.399	E
WC21298.03	RBL98.242	Z	TC042706.01	RBL06.400	A
WC21298.04	RBL98.243	E	TC042706.02	RBL06.401	A
WC21298.05	RBL98.244	R	TC042706.03	RBL06.402	B
WC21298.06	RBL98.245	E	TC042706.04	RBL06.403	B
WC21298.07	RBL98.246	E	TC042706.05	RBL06.404	R
WC21298.08	RBL98.247	R	TC042706.06	RBL06.405	B
WC21298.09	RBL98.248	R	TC042706.07	RBL06.406	R
WC21298.10	RBL98.249	A	TC042706.09	RBL06.408	B
WC21298.11	RBL98.250	E	TC042706.10	RBL06.409	B
WC21298.12	RBL98.251	E	TC042706.11	RBL06.410	B
WC21298.13	RBL98.252	E	TC042706.12	RBL06.411	B
WC21298.14	RBL98.253	Z	TC042706.13	RBL06.412	B
WC21298.15	RBL98.254	E	TC042706.14	RBL06.413	B
WC21298.16	RBL98.255	Q	TC042706.15	RBL06.414	B
WC21298.17	RBL98.256	E	TC042706.16	RBL06.415	B
WC21298.18	RBL98.257	A	TC042706.17	RBL06.416	R
WC21298.19	RBL98.258	E	TC042706.18	RBL06.417	R
WC21298.20	RBL98.259	E	TC042706.19	RBL06.418	B
WC21298.21	RBL98.260	E	TC042706.20	RBL06.419	B
WC21298.22	RBL98.261	E	TC042706.21	RBL06.420	R
WC21298.23	RBL98.262	E	TC042706.22	RBL06.421	B
WC21298.24	RBL98.263	E	TC042706.23	RBL06.422	B
WC21298.25	RBL98.264	E	TC042706.24	RBL06.423	E
WC21298.26	RBL98.265	R	TC042706.25	RBL06.424	R
WC22798.01	RBL98.266	S	YC020706.01	RBL06.452	E
WC22798.02	RBL98.267	E	YC020706.02	RBL06.453	E
WC22798.03	RBL98.268	S	YC020706.03	RBL06.454	E
WC22798.04	RBL98.269	S	YC020706.04	RBL06.455	E
WC22798.05	RBL98.270	R	YC020706.05	RBL06.456	E
WC22798.06	RBL98.271	A	YC020706.06	RBL06.457	E
WC22798.07	RBL98.272	E	YC020706.07	RBL06.458	E
WC22798.08	RBL98.273	E	YC020706.08	RBL06.459	E
WC22798.09	RBL98.274	E	YC020706.09	RBL06.460	E

WC22798.10	RBL98.275	S	YC020706.10	RBL06.461	E
WC22798.11	RBL98.276	E	YC020706.11	RBL06.462	V
WC22798.12	RBL98.277	A	YC020706.12	RBL06.463	E
WC22798.13	RBL98.278	S	YC020706.13	RBL06.464	E
WC22798.14	RBL98.279	S	YC020706.14	RBL06.465	E
WC22798.15	RBL98.280	E	YC020706.15	RBL06.466	J
WC22798.16	RBL98.281	E	YC020706.16	RBL06.467	E
WC22798.17	RBL98.282	E	YC020706.17	RBL06.468	C
WC22798.18	RBL98.283	E	YC020706.18	RBL06.469	E
WC22798.19	RBL98.284	E	YC020706.19	RBL06.470	E
WC22798.20	RBL98.285	A	YC020706.20	RBL06.471	F
WC22798.21	RBL98.286	E	YC020706.21	RBL06.472	B
WC22798.22	RBL98.287	E	YC020706.22	RBL06.473	E
WC22798.23	RBL98.288	E	YC020706.23	RBL06.474	E
WC22798.24	RBL98.289	E	YC020706.24	RBL06.475	E
WC22798.25	RBL98.290	E	YC020706.25	RBL06.476	E
WC22798.26	RBL98.291	E	YC020706.26	RBL06.477	E
WC22798.27	RBL98.292	E	YC022306.01	RBL06.478	E
WC22798.28	RBL98.293	E	YC022306.02	RBL06.479	J
WC22798.29	RBL98.294	S	YC022306.03	RBL06.480	H
WC22798.30	RBL98.295	B	YC022306.04	RBL06.481	E
WC31798.01	RBL98.296	E	YC022306.05	RBL06.482	E
WC31798.02	RBL98.297	H	YC022306.06	RBL06.483	E
WC31798.03	RBL98.298	E	YC022306.07	RBL06.484	E
WC31798.04	RBL98.299	F	YC022306.08	RBL06.485	R
WC31798.05	RBL98.300	E	YC022306.09	RBL06.486	E
WC31798.06	RBL98.301	E	YC022306.10	RBL06.487	E
WC31798.07	RBL98.302	A	YC022306.11	RBL06.488	CC
WC31798.09	RBL98.304	C	YC022306.12	RBL06.489	R
WC31798.10	RBL98.305	E	YC022306.13	RBL06.490	S
WC31798.11	RBL98.306	E	YC022306.14	RBL06.491	E
WC31798.12	RBL98.307	B	YC022306.15	RBL06.492	CC
WC31798.13	RBL98.308	E	YC022306.16	RBL06.493	E
WC31798.14	RBL98.309	E	YC022306.17	RBL06.494	E
WC31798.15	RBL98.310	B	YC022306.18	RBL06.495	B
WC31798.16	RBL98.311	E	YC022306.19	RBL06.496	E
WC31798.17	RBL98.312	E	YC022306.20	RBL06.497	E
WC31798.18	RBL98.313	E	YC022306.21	RBL06.498	E
WC31798.19	RBL98.314	E	YC022306.22	RBL06.499	R
WC31798.21	RBL98.316	I	YC022306.23	RBL06.500	E
WC31798.22	RBL98.317	E	YC022306.24	RBL06.501	S

WC31798.23	RBL98.318	E	YC022306.25	RBL06.502	E
WC31798.24	RBL98.319	E	YC030806.01	RBL06.503	E
WC31798.25	RBL98.320	E	YC030806.02	RBL06.504	K
WC4698.01	RBL98.321	E	YC030806.03	RBL06.505	E
WC4698.02	RBL98.322	E	YC030806.04	RBL06.506	E
WC4698.03	RBL98.323	E	YC030806.05	RBL06.507	E
WC4698.04	RBL98.324	E	YC030806.06	RBL06.508	E
WC4698.05	RBL98.325	B	YC030806.07	RBL06.509	P
WC4698.06	RBL98.326	E	YC030806.08	RBL06.510	E
WC4698.07	RBL98.327	E	YC030806.09	RBL06.511	E
WC4698.08	RBL98.328	E	YC030806.10	RBL06.512	R
WC4698.09	RBL98.329	E	YC030806.11	RBL06.513	K
WC4698.10	RBL98.330	E	YC030806.12	RBL06.514	R
WC4698.11	RBL98.331	E	YC030806.13	RBL06.515	E
WC4698.12	RBL98.332	E	YC030806.14	RBL06.516	E
WC4698.13	RBL98.333	S	YC030806.15	RBL06.517	R
WC4698.14	RBL98.334	E	YC030806.16	RBL06.518	E
WC4698.15	RBL98.335	E	YC030806.17	RBL06.519	V
WC4698.16	RBL98.336	E	YC030806.18	RBL06.520	R
WC4698.17	RBL98.337	J	YC030806.19	RBL06.521	B
WC4698.18	RBL98.338	J	YC030806.20	RBL06.522	F
WC4698.19	RBL98.339	E	YC030806.21	RBL06.523	E
WC4698.20	RBL98.340	E	YC030806.22	RBL06.524	E
WC4698.21	RBL98.341	E	YC030806.23	RBL06.525	C
WC4698.22	RBL98.342	E	YC030806.24	RBL06.526	F
WC4698.23	RBL98.343	E	YC030806.25	RBL06.527	E
WC4698.24	RBL98.344	E	YC031406.01	RBL06.528	E
WC4698.25	RBL98.345	C	YC031406.02	RBL06.529	E
YC13198.01	RBL98.346	E	YC031406.03	RBL06.530	R
YC13198.02	RBL98.347	J	YC031406.04	RBL06.531	V
YC13198.03	RBL98.348	E	YC031406.05	RBL06.532	E
YC13198.04	RBL98.349	J	YC031406.06	RBL06.533	E
YC13198.05	RBL98.350	E	YC031406.07	RBL06.534	F
YC13198.06	RBL98.351	E	YC031406.08	RBL06.535	R
YC13198.07	RBL98.352	E	YC031406.09	RBL06.536	E
YC13198.08	RBL98.353	B	YC031406.10	RBL06.537	E
YC13198.09	RBL98.354	E	YC031406.11	RBL06.538	E
YC13198.10	RBL98.355	E	YC031406.12	RBL06.539	R
YC13198.11	RBL98.356	J	YC031406.13	RBL06.540	E
YC13198.12	RBL98.357	V	YC031406.14	RBL06.541	R
YC13198.13	RBL98.358	R	YC031406.15	RBL06.542	E

YC13198.14	RBL98.359	E	YC031406.16	RBL06.543	F
YC13198.15	RBL98.360	E	YC031406.17	RBL06.544	E
YC13198.16	RBL98.361	E	YC031406.18	RBL06.545	E
YC13198.17	RBL98.362	E	YC031406.19	RBL06.546	E
YC13198.18	RBL98.363	E	YC031406.20	RBL06.547	R
YC13198.19	RBL98.364	B	YC031406.21	RBL06.548	E
YC13198.20	RBL98.365	E	YC031406.22	RBL06.549	E
YC13198.21	RBL98.366	B	YC031406.23	RBL06.550	E
YC13198.22	RBL98.367	B	YC031406.24	RBL06.551	E
YC13198.23	RBL98.368	V	YC031406.25	RBL06.552	E
YC13198.24	RBL98.369	B	YC031406.26	RBL06.553	E
YC13198.25	RBL98.370	H	YC040506.01	RBL06.554	A
YC13198.26	RBL98.371	E	YC040506.02	RBL06.555	E
YC13198.27	RBL98.372	B	YC040506.03	RBL06.556	E
YC13198.28	RBL98.373	E	YC040506.04	RBL06.557	J
YC13198.29	RBL98.374	V	YC040506.05	RBL06.558	E
YC13198.30	RBL98.375	R	YC040506.06	RBL06.559	A
YC21298.01	RBL98.376	H	YC040506.07	RBL06.560	E
YC21298.02	RBL98.377	E	YC040506.08	RBL06.561	E
YC21298.03	RBL98.378	E	YC040506.09	RBL06.562	E
YC21298.04	RBL98.379	R	YC040506.10	RBL06.563	B
YC21298.05	RBL98.380	E	YC040506.11	RBL06.564	E
YC21298.06	RBL98.381	E	YC040506.12	RBL06.565	R
YC21298.07	RBL98.382	R	YC040506.13	RBL06.566	J
YC21298.08	RBL98.383	E	YC040506.14	RBL06.567	L
YC21298.09	RBL98.384	E	YC040506.15	RBL06.568	E
YC21298.10	RBL98.385	C	YC040506.16	RBL06.569	E
YC21298.11	RBL98.386	P	YC040506.17	RBL06.570	E
YC21298.12	RBL98.387	R	YC040506.18	RBL06.571	E
YC21298.13	RBL98.388	E	YC040506.19	RBL06.572	E
YC21298.14	RBL98.389	E	YC040506.20	RBL06.573	B
YC21298.15	RBL98.390	B	YC040506.21	RBL06.574	E
YC21298.16	RBL98.391	E	YC040506.22	RBL06.575	E
YC21298.17	RBL98.392	E	YC040506.23	RBL06.576	E
YC21298.18	RBL98.393	C	YC040506.24	RBL06.577	E
YC21298.19	RBL98.394	E	YC042506.01	RBL06.578	B
YC21298.20	RBL98.395	B	YC042506.02	RBL06.579	E
YC21298.21	RBL98.396	A	YC042506.03	RBL06.580	R
YC21298.22	RBL98.397	H	YC042506.04	RBL06.581	B
YC21298.23	RBL98.398	E	YC042506.05	RBL06.582	E
YC21298.24	RBL98.399	R	YC042506.06	RBL06.583	E

YC21298.25	RBL98.400	B	YC042506.07	RBL06.584	B
YC21298.26	RBL98.401	E	YC042506.08	RBL06.585	B
YC21298.27	RBL98.402	A	YC042506.09	RBL06.586	B
YC21298.28	RBL98.403	R	YC042506.10	RBL06.587	B
YC21298.29	RBL98.404	R	YC042506.11	RBL06.588	E
YC22798.01	RBL98.406	E	YC042506.12	RBL06.589	B
YC22798.02	RBL98.407	E	YC042506.13	RBL06.590	B
YC22798.03	RBL98.408	E	YC042506.14	RBL06.591	B
YC22798.04	RBL98.409	R	YC042506.15	RBL06.592	E
YC22798.05	RBL98.410	B	YC042506.16	RBL06.593	E
YC22798.06	RBL98.411	E	YC042506.17	RBL06.594	E
YC22798.07	RBL98.412	E	YC042506.18	RBL06.595	E
YC22798.08	RBL98.413	J	YC042506.19	RBL06.596	R
YC22798.09	RBL98.414	E	YC042506.20	RBL06.597	B
YC22798.10	RBL98.415	V	YC042506.21	RBL06.598	R
YC22798.11	RBL98.416	E	YC042506.22	RBL06.599	B
YC22798.12	RBL98.417	E	YC042506.23	RBL06.600	E
YC22798.13	RBL98.418	B	YC042506.24	RBL06.601	E
YC22798.14	RBL98.419	E	YC042506.25	RBL06.602	B
YC22798.15	RBL98.420	E	YC042506.26	RBL06.603	E
YC22798.16	RBL98.421	E	YC042506.27	RBL06.604	B
YC22798.17	RBL98.422	B	YC042506.28	RBL06.605	E
YC22798.18	RBL98.423	E	YC032206.01	RBL06.606	E
YC22798.19	RBL98.424	E	YC032206.02	RBL06.607	A
YC22798.20	RBL98.425	E	YC032206.03	RBL06.608	BB
YC22798.21	RBL98.426	B	YC032206.04	RBL06.609	E
YC22798.22	RBL98.427	E	YC032206.05	RBL06.610	E
YC22798.23	RBL98.428	B	YC032206.06	RBL06.611	E
YC22798.24	RBL98.429	B	YC032206.07	RBL06.612	R
YC22798.26	RBL98.431	S	YC032206.08	RBL06.613	E
YC22798.27	RBL98.432	E	YC032206.09	RBL06.614	E
YC22798.28	RBL98.433	E	YC032206.10	RBL06.615	E
YC22798.29	RBL98.434	B	YC032206.11	RBL06.616	E
YC22798.30	RBL98.435	E	YC032206.12	RBL06.617	H
YC31798.01	RBL98.436	E	YC032206.13	RBL06.618	R
YC31798.02	RBL98.437	E	YC032206.14	RBL06.619	E
YC31798.03	RBL98.438	R	YC032206.15	RBL06.620	E
YC31798.04	RBL98.439	E	YC032206.16	RBL06.621	E
YC31798.05	RBL98.440	E	YC032206.17	RBL06.622	A
YC31798.06	RBL98.441	E	YC032206.18	RBL06.623	E
YC31798.07	RBL98.442	E	YC032206.19	RBL06.624	E

YC31798.08	RBL98.443	E	YC032206.20	RBL06.625	R
YC31798.09	RBL98.444	B	YC032206.21	RBL06.626	E
YC31798.10	RBL98.445	E	YC032206.22	RBL06.627	A
YC31798.11	RBL98.446	E	YC032206.23	RBL06.628	E
YC31798.12	RBL98.447	E	YC032206.24	RBL06.629	E
YC31798.13	RBL98.448	E	YC032206.25	RBL06.630	E
YC31798.14	RBL98.449	E	YC020507.01	RBL07.001	E
YC31798.15	RBL98.450	E	YC020507.02	RBL07.002	H
YC31798.16	RBL98.451	Z	YC020507.03	RBL07.003	E
YC31798.17	RBL98.452	E	YC020507.04	RBL07.004	E
YC31798.18	RBL98.453	E	YC020507.05	RBL07.005	E
YC31798.19	RBL98.454	B	YC020507.06	RBL07.006	E
YC31798.20	RBL98.455	E	YC020507.07	RBL07.007	E
YC31798.21	RBL98.456	E	YC020507.08	RBL07.008	E
YC31798.22	RBL98.457	F	YC020507.09	RBL07.009	M
YC31798.23	RBL98.458	E	YC020507.10	RBL07.010	C
YC31798.24	RBL98.459	E	YC020507.11	RBL07.011	B
YC31798.25	RBL98.460	E	YC020507.12	RBL07.012	E
YC31798.26	RBL98.461	E	YC020507.13	RBL07.013	H
YC31798.27	RBL98.462	AA	YC020507.14	RBL07.014	E
YC31798.28	RBL98.463	E	YC020507.15	RBL07.015	H
YC31798.29	RBL98.464	E	YC020507.16	RBL07.016	H
YC31798.30	RBL98.465	E	YC020507.17	RBL07.017	H
YC4698.01	RBL98.466	S	YC020507.18	RBL07.018	B
YC4698.02	RBL98.467	B	YC020507.19	RBL07.019	E
YC4698.03	RBL98.468	E	YC020507.20	RBL07.020	E
YC4698.04	RBL98.469	E	YC020507.21	RBL07.021	R
YC4698.05	RBL98.470	E	YC020507.22	RBL07.022	E
YC4698.06	RBL98.471	S	YC020507.23	RBL07.023	E
YC4698.07	RBL98.472	C	YC020507.24	RBL07.024	E
YC4698.08	RBL98.473	E	YC020507.25	RBL07.025	E
YC4698.09	RBL98.474	E	YC022007.01	RBL07.026	F
YC4698.10	RBL98.475	E	YC022007.02	RBL07.027	C
YC4698.11	RBL98.476	E	YC022007.03	RBL07.028	H
YC4698.12	RBL98.477	B	YC022007.04	RBL07.029	E
YC4698.13	RBL98.478	E	YC022007.05	RBL07.030	E
YC4698.14	RBL98.479	E	YC022007.06	RBL07.031	FF
YC4698.15	RBL98.480	E	YC022007.07	RBL07.032	E
YC4698.16	RBL98.481	E	YC022007.08	RBL07.033	E
YC4698.17	RBL98.482	P	YC022007.09	RBL07.034	E
YC4698.18	RBL98.483	E	YC022007.10	RBL07.035	E

YC4698.19	RBL98.484	E	YC022007.11	RBL07.036	E
YC4698.20	RBL98.485	E	YC022007.12	RBL07.037	B
YC4698.21	RBL98.486	E	YC022007.13	RBL07.038	E
YC4698.22	RBL98.487	C	YC022007.14	RBL07.039	F
YC4698.23	RBL98.488	E	YC022007.15	RBL07.040	K
YC4698.24	RBL98.489	E	YC022007.16	RBL07.041	E
YC4698.25	RBL98.490	E	YC022007.17	RBL07.042	F
WC021999.01	RBL99.001	E	YC022007.18	RBL07.043	E
WC021999.02	RBL99.002	R	YC022007.19	RBL07.044	E
WC021999.03	RBL99.003	R	YC022007.20	RBL07.045	R
WC021999.04	RBL99.004	E	YC022007.21	RBL07.046	E
WC021999.05	RBL99.005	P	YC022007.22	RBL07.047	K
WC021999.06	RBL99.006	E	YC022007.23	RBL07.048	C
WC021999.07	RBL99.007	B	YC022007.24	RBL07.049	E
HH022099.01	RBL99.008	E	YC022007.25	RBL07.050	A
HH022099.02	RBL99.009	H	YC031207.01	RBL07.051	S
HH022099.03	RBL99.010	R	YC031207.02	RBL07.052	E
HH022099.04	RBL99.011	S	YC031207.03	RBL07.053	B
HH022099.05	RBL99.012	E	YC031207.04	RBL07.054	A
HH022099.06	RBL99.013	E	YC031207.05	RBL07.055	E
HH022099.07	RBL99.014	R	YC031207.06	RBL07.056	H
HH022099.08	RBL99.015	E	YC031207.07	RBL07.057	E
HH022099.09	RBL99.016	E	YC031207.08	RBL07.058	B
HH022099.10	RBL99.017	E	YC031207.09	RBL07.059	E
HH022099.11	RBL99.018	A	YC031207.10	RBL07.060	E
HH022099.12	RBL99.019	V	YC031207.11	RBL07.061	E
HH022099.13	RBL99.020	E	YC031207.12	RBL07.062	R
HH022099.14	RBL99.021	E	YC031207.13	RBL07.063	A
HH022099.15	RBL99.022	E	YC031207.14	RBL07.064	E
HH022099.16	RBL99.023	B	YC031207.15	RBL07.065	E
HH022099.17	RBL99.024	S	YC031207.16	RBL07.066	E
HH022099.18	RBL99.025	S	YC031207.17	RBL07.067	E
HH022099.19	RBL99.026	V	YC031207.18	RBL07.068	E
HH022099.20	RBL99.027	E	YC031207.19	RBL07.069	E
HH022099.21	RBL99.028	E	YC031207.20	RBL07.070	R
HH022099.22	RBL99.029	E	YC031207.21	RBL07.071	E
HH022099.23	RBL99.030	E	YC031207.22	RBL07.072	R
HH022099.24	RBL99.031	E	YC031207.23	RBL07.073	B
HH022099.25	RBL99.032	E	YC031207.24	RBL07.074	R
TC022099.01	RBL99.033	E	YC031207.25	RBL07.075	E
TC022099.02	RBL99.034	A	YC031207.26	RBL07.076	E

TC022099.03	RBL99.035	E	YC031207.27	RBL07.077	B
TC022099.04	RBL99.036	E	YC031207.28	RBL07.078	A
TC022099.05	RBL99.037	E	YC031207.29	RBL07.079	E
TC022099.06	RBL99.038	E	YC031207.30	RBL07.080	A
TC022099.07	RBL99.039	E	YC031207.31	RBL07.081	C
TC022099.08	RBL99.040	E	YC031207.32	RBL07.082	E
TC022099.09	RBL99.041	E	YC031207.33	RBL07.083	C
TC022099.10	RBL99.042	E	YC031207.34	RBL07.084	B
TC022099.11	RBL99.043	E	YC031207.35	RBL07.085	B
TC022099.12	RBL99.044	E	NM020607.01	RBL07.086	E
TC022099.13	RBL99.045	E	NM020607.02	RBL07.087	E
TC022099.14	RBL99.046	E	NM020607.03	RBL07.088	H
TC022099.15	RBL99.047	A	NM020607.04	RBL07.089	F
TC022099.16	RBL99.048	E	NM020607.05	RBL07.090	S
TC022099.17	RBL99.049	E	NM020607.06	RBL07.091	S
TC022099.18	RBL99.050	E	NM020607.07	RBL07.092	F
TC022099.19	RBL99.051	B	NM020607.08	RBL07.093	E
TC022099.20	RBL99.052	E	NM020607.09	RBL07.094	E
TC022099.21	RBL99.053	E	NM020607.10	RBL07.095	E
TC022099.22	RBL99.054	E	NM020607.11	RBL07.096	E
TC022099.23	RBL99.055	E	NM020607.12	RBL07.097	S
TC022099.24	RBL99.056	B	NM020607.13	RBL07.098	S
TC022099.25	RBL99.057	F	NM020607.14	RBL07.099	P
YC022099.01	RBL99.058	E	NM020607.15	RBL07.100	E
YC022099.02	RBL99.059	E	NM020607.16	RBL07.101	B
YC022099.03	RBL99.060	E	NM020607.17	RBL07.102	E
YC022099.04	RBL99.061	E	NM020607.18	RBL07.103	I
YC022099.05	RBL99.062	E	NM020607.19	RBL07.104	p
YC022099.06	RBL99.063	B	NM020607.20	RBL07.105	E
YC022099.07	RBL99.064	E	NM020607.21	RBL07.106	S
YC022099.08	RBL99.065	E	NM020607.22	RBL07.107	S
YC022099.09	RBL99.066	E	NM020607.23	RBL07.108	E
YC022099.10	RBL99.067	B	NM020607.24	RBL07.109	F
YC022099.11	RBL99.068	R	NM020607.25	RBL07.110	E
YC022099.12	RBL99.069	E	NM022707.01	RBL07.111	E
YC022099.13	RBL99.070	V	NM022707.02	RBL07.112	S
YC022099.14	RBL99.071	S	NM022707.03	RBL07.113	C
YC022099.15	RBL99.072	E	NM022707.04	RBL07.114	S
YC022099.16	RBL99.073	U	NM022707.05	RBL07.115	S
YC022099.17	RBL99.074	E	NM022707.06	RBL07.116	E
YC022099.18	RBL99.075	E	NM022707.07	RBL07.117	E

YC022099.19	RBL99.076	E	NM022707.08	RBL07.118	B
YC022099.20	RBL99.077	E	NM022707.09	RBL07.119	E
YC022099.21	RBL99.078	E	NM022707.10	RBL07.120	A
YC022099.22	RBL99.079	E	NM022707.11	RBL07.121	S
YC022099.23	RBL99.080	B	NM022707.12	RBL07.122	S
YC022099.24	RBL99.081	E	NM022707.13	RBL07.123	A
YC022099.25	RBL99.082	E	NM022707.14	RBL07.124	E
WC030699.01	RBL99.083	E	NM022707.15	RBL07.125	E
WC030699.02	RBL99.084	E	NM022707.16	RBL07.126	B
WC030699.03	RBL99.085	E	NM022707.17	RBL07.127	A
WC030699.04	RBL99.086	E	NM022707.18	RBL07.128	R
WC030699.05	RBL99.087	B	NM022707.19	RBL07.129	E
WC030699.06	RBL99.088	E	NM022707.20	RBL07.130	A
WC030699.07	RBL99.089	E	NM031507.01	RBL07.132	R
WC030699.08	RBL99.090	F	NM031507.02	RBL07.133	E
WC030699.09	RBL99.091	E	NM031507.03	RBL07.134	E
WC030699.10	RBL99.092	B	NM031507.04	RBL07.135	B
WC030699.11	RBL99.093	E	NM031507.05	RBL07.136	E
WC030699.12	RBL99.094	E	NM031507.06	RBL07.137	E
WC030699.13	RBL99.095	E	NM031507.07	RBL07.138	S
WC030699.14	RBL99.096	B	NM031507.08	RBL07.139	E
WC030699.15	RBL99.097	E	NM031507.09	RBL07.140	E
WC030699.16	RBL99.098	E	NM031507.10	RBL07.141	E
WC030699.17	RBL99.099	B	NM031507.11	RBL07.142	C
WC030699.18	RBL99.100	E	NM031507.12	RBL07.143	E
WC030699.19	RBL99.101	E	NM031507.13	RBL07.144	E
WC030699.20	RBL99.102	B	NM031507.14	RBL07.145	E
WC030699.21	RBL99.103	E	NM031507.15	RBL07.146	E
WC030699.22	RBL99.104	E	NM031507.16	RBL07.147	R
WC030699.23	RBL99.105	M	NM031507.17	RBL07.148	E
WC030699.24	RBL99.106	F	NM031507.18	RBL07.149	S
WC030699.25	RBL99.107	E	NM031507.19	RBL07.150	E
TC030699.01	RBL99.108	E	NM031507.20	RBL07.151	E
TC030699.02	RBL99.109	E	NM031507.21	RBL07.152	E
TC030699.03	RBL99.110	E	NM031507.22	RBL07.153	E
TC030699.04	RBL99.111	E	NM031507.23	RBL07.154	E
TC030699.05	RBL99.112	E	NM031507.24	RBL07.155	E
TC030699.06	RBL99.113	E	NM031507.25	RBL07.156	E
TC030699.07	RBL99.114	E	NM022307.01	RBL07.157	E
TC030699.08	RBL99.115	R	NM022307.02	RBL07.158	E
TC030699.09	RBL99.116	E	NM022307.03	RBL07.159	E

TC030699.11	RBL99.118	E	NM022307.04	RBL07.160	R
TC030699.12	RBL99.119	E	NM022307.05	RBL07.161	Q
TC030699.13	RBL99.120	E	TC031407.01	RBL07.162	E
TC030699.14	RBL99.121	E	TC031407.02	RBL07.163	E
TC030699.15	RBL99.122	E	TC031407.03	RBL07.164	E
TC030699.16	RBL99.123	E	TC031407.04	RBL07.165	CC
TC030699.17	RBL99.124	E	TC031407.05	RBL07.166	R
TC030699.18	RBL99.125	E	TC031407.06	RBL07.167	E
TC030699.19	RBL99.126	E	TC031407.07	RBL07.168	C
TC030699.20	RBL99.127	E	TC031407.08	RBL07.169	E
TC030699.21	RBL99.128	R	TC031407.09	RBL07.170	E
TC030699.22	RBL99.129	E	TC031407.10	RBL07.171	R
TC030699.23	RBL99.130	E	TC031407.11	RBL07.172	E
TC030699.24	RBL99.131	E	TC031407.12	RBL07.173	E
TC030699.25	RBL99.132	E	TC031407.13	RBL07.174	E
TC030699.26	RBL99.133	E	TC031407.14	RBL07.175	R
YC030699.01	RBL99.134	B	TC031407.15	RBL07.176	E
YC030699.02	RBL99.135	E	TC031407.16	RBL07.177	E
YC030699.03	RBL99.136	E	TC031407.17	RBL07.178	E
YC030699.04	RBL99.137	B	TC031407.18	RBL07.179	E
YC030699.05	RBL99.138	E	TC031407.19	RBL07.180	E
YC030699.06	RBL99.139	E	TC031407.20	RBL07.181	R
YC030699.07	RBL99.140	B	TC031407.21	RBL07.182	E
YC030699.08	RBL99.141	F	TC031407.22	RBL07.183	F
YC030699.09	RBL99.142	E	TC031407.23	RBL07.184	E
YC030699.10	RBL99.143	R	TC031407.24	RBL07.185	E
YC030699.11	RBL99.144	E	TC031407.25	RBL07.186	E
YC030699.12	RBL99.145	R	TC020707.01	RBL07.187	E
YC030699.13	RBL99.146	W	TC020707.02	RBL07.188	E
YC030699.14	RBL99.147	J	TC020707.03	RBL07.189	B
YC030699.15	RBL99.148	S	TC020707.04	RBL07.190	E
YC030699.16	RBL99.149	E	TC020707.05	RBL07.191	E
YC030699.17	RBL99.150	S	TC020707.06	RBL07.192	E
YC030699.18	RBL99.151	S	TC020707.07	RBL07.193	E
YC030699.19	RBL99.152	E	TC020707.08	RBL07.194	E
YC030699.20	RBL99.153	W	TC020707.09	RBL07.195	E
YC030699.21	RBL99.154	E	TC020707.10	RBL07.196	S
YC030699.22	RBL99.155	E	TC020707.11	RBL07.197	S
YC030699.23	RBL99.156	B	TC020707.12	RBL07.198	E
YC030699.24	RBL99.157	R	TC020707.13	RBL07.199	E
YC030699.25	RBL99.158	E	TC020707.14	RBL07.200	S

YC031699.01	RBL99.159	E	TC020707.15	RBL07.201	S
YC031699.02	RBL99.160	R	TC020707.16	RBL07.202	S
YC031699.03	RBL99.161	E	TC020707.17	RBL07.203	E
YC031699.04	RBL99.162	E	TC020707.18	RBL07.204	E
YC031699.06	RBL99.164	E	TC020707.19	RBL07.205	E
YC031699.07	RBL99.165	E	TC020707.20	RBL07.206	E
YC031699.08	RBL99.166	E	TC020707.21	RBL07.207	B
YC031699.09	RBL99.167	E	TC020707.22	RBL07.208	A
YC031699.10	RBL99.168	E	TC020707.23	RBL07.209	E
YC031699.11	RBL99.169	E	TC020707.24	RBL07.210	A
YC031699.12	RBL99.170	E	TC020707.25	RBL07.211	B
YC031699.13	RBL99.171	E	TC022007.01	RBL07.212	O
YC031699.14	RBL99.172	E	TC022007.02	RBL07.213	B
YC031699.15	RBL99.173	E	TC022007.03	RBL07.214	E
YC031699.16	RBL99.174	E	TC022007.04	RBL07.215	R
YC031699.17	RBL99.175	B	TC022007.05	RBL07.216	R
YC031699.18	RBL99.176	E	TC022007.06	RBL07.217	E
YC031699.19	RBL99.177	F	TC022007.07	RBL07.218	B
YC031699.20	RBL99.178	E	TC022007.08	RBL07.219	B
YC031699.21	RBL99.179	E	TC022007.09	RBL07.220	B
YC031699.22	RBL99.180	E	TC022007.10	RBL07.221	A
YC031699.23	RBL99.181	E	TC022007.11	RBL07.222	E
YC031699.24	RBL99.182	E	TC022007.12	RBL07.223	E
YC031699.25	RBL99.183	F	TC022007.13	RBL07.224	E
YC031699.26	RBL99.184	E	TC022007.14	RBL07.225	S
WC031899.02	RBL99.187	E	TC022007.15	RBL07.226	S
WC031899.03	RBL99.188	E	TC022007.16	RBL07.227	S
WC031899.04	RBL99.189	E	TC022007.17	RBL07.228	BB
WC031899.05	RBL99.190	E	TC022007.18	RBL07.229	E
WC031899.06	RBL99.191	E	TC022007.19	RBL07.230	E
WC031899.07	RBL99.192	E	TC022007.20	RBL07.231	E
WC031899.08	RBL99.193	E	TC022007.21	RBL07.232	R
WC031899.09	RBL99.194	E	TC022007.22	RBL07.233	A
WC031899.10	RBL99.195	E	TC022007.23	RBL07.234	E
WC031899.11	RBL99.196	E	CC031407.01	RBL07.235	F
WC031899.12	RBL99.197	E	CC031407.02	RBL07.236	E
WC031899.13	RBL99.198	B	CC031407.03	RBL07.237	E
WC031899.14	RBL99.199	F	CC031407.04	RBL07.238	E
WC031899.15	RBL99.200	E	CC031407.05	RBL07.239	B
WC031899.16	RBL99.201	E	CC031407.06	RBL07.240	E
WC031899.17	RBL99.202	P	CC031407.07	RBL07.241	E

WC031899.19	RBL99.204	E	CC031407.08	RBL07.242	E
WC031899.20	RBL99.205	E	CC031407.09	RBL07.243	C
WC031899.21	RBL99.206	E	CC031407.10	RBL07.244	S
WC031899.22	RBL99.207	E	CC031407.11	RBL07.245	E
WC031899.23	RBL99.208	E	CC031407.12	RBL07.246	E
WC031899.24	RBL99.209	E	CC031407.13	RBL07.247	E
WC031899.25	RBL99.210	E	CC031407.14	RBL07.248	E
WC031899.26	RBL99.211	E	CC031407.15	RBL07.249	BB
HH031899.01	RBL99.212	R	CC031407.16	RBL07.250	E
HH031899.02	RBL99.213	E	CC031407.17	RBL07.251	J
HH031899.03	RBL99.214	E	CC031407.18	RBL07.252	E
HH031899.04	RBL99.215	E	CC031407.19	RBL07.253	E
HH031899.05	RBL99.216	B	CC031407.20	RBL07.254	E
HH031899.06	RBL99.217	E	CC031407.21	RBL07.255	E
HH031899.07	RBL99.218	S	CC031407.22	RBL07.256	E
HH031899.08	RBL99.219	E	CC031407.23	RBL07.257	E
HH031899.09	RBL99.220	B	CC031407.24	RBL07.258	R
HH031899.10	RBL99.221	B	CC031407.25	RBL07.259	E
HH031899.11	RBL99.222	E	WC040507.01	RBL07.260	E
HH031899.12	RBL99.223	E	WC040507.02	RBL07.261	C
HH031899.13	RBL99.224	E	WC040507.03	RBL07.262	E
HH031899.14	RBL99.225	E	WC040507.04	RBL07.263	E
HH031899.15	RBL99.226	E	WC040507.05	RBL07.264	E
HH031899.16	RBL99.227	S	WC040507.06	RBL07.265	E
HH031899.17	RBL99.228	S	WC040507.07	RBL07.266	E
HH031899.18	RBL99.229	E	WC040507.08	RBL07.267	j
HH031899.19	RBL99.230	E	WC040507.09	RBL07.268	E
HH031899.20	RBL99.231	E	WC040507.10	RBL07.269	R
HH031899.21	RBL99.232	R	WC040507.11	RBL07.270	E
HH031899.22	RBL99.233	B	WC040507.12	RBL07.271	E
HH031899.23	RBL99.234	F	WC040507.13	RBL07.272	E
HH031899.24	RBL99.235	E	WC040507.14	RBL07.273	S
HH031899.25	RBL99.236	E	WC040507.15	RBL07.274	E
HH031899.26	RBL99.237	E	WC040507.16	RBL07.275	E
TC031899.01	RBL99.238	E	WC040507.17	RBL07.276	E
TC031899.02	RBL99.239	E	WC040507.18	RBL07.277	S
TC031899.03	RBL99.240	P	WC040507.19	RBL07.278	M
TC031899.04	RBL99.241	E	WC040507.20	RBL07.279	E
TC031899.05	RBL99.242	E	WC040507.21	RBL07.280	E
TC031899.06	RBL99.243	B	WC040507.22	RBL07.281	E
TC031899.07	RBL99.244	F	WC040507.23	RBL07.282	E

TC031899.08	RBL99.245	E	WC040507.24	RBL07.283	A
TC031899.09	RBL99.246	B	WC040507.25	RBL07.284	E
TC031899.10	RBL99.247	E	WC041707.01	RBL07.285	B
TC031899.11	RBL99.248	E	WC041707.02	RBL07.286	S
TC031899.12	RBL99.249	E	WC041707.03	RBL07.287	E
TC031899.13	RBL99.250	S	WC041707.04	RBL07.288	K
TC031899.14	RBL99.251	E	WC041707.05	RBL07.289	E
TC031899.15	RBL99.252	E	WC041707.06	RBL07.290	E
TC031899.16	RBL99.253	R	WC041707.07	RBL07.291	E
TC031899.17	RBL99.254	E	WC041707.08	RBL07.292	E
TC031899.18	RBL99.255	B	WC041707.09	RBL07.293	S
TC031899.19	RBL99.256	F	WC041707.10	RBL07.294	R
TC031899.20	RBL99.257	E	WC041707.11	RBL07.295	E
TC031899.21	RBL99.258	E	WC041707.12	RBL07.296	E
TC031899.22	RBL99.259	E	WC041707.13	RBL07.297	E
TC031899.23	RBL99.260	F	WC041707.14	RBL07.298	E
TC031899.24	RBL99.261	E	WC041707.15	RBL07.299	G
TC031899.25	RBL99.262	P	WC041707.16	RBL07.300	E
TC031899.26	RBL99.263	E	WC041707.17	RBL07.301	E
HH040299.01	RBL99.264	E	WC041707.18	RBL07.302	E
HH040299.04	RBL99.267	G	WC041707.19	RBL07.303	E
HH040299.05	RBL99.268	G	WC041707.20	RBL07.304	E
HH040299.06	RBL99.269	E	WC041707.21	RBL07.305	E
HH040299.07	RBL99.270	E	WC041707.22	RBL07.306	S
HH040299.08	RBL99.271	E	WC041707.23	RBL07.307	S
HH040299.09	RBL99.272	E	WC041707.24	RBL07.308	U
HH040299.10	RBL99.273	E	WC041707.25	RBL07.309	S
TC040299.01	RBL99.274	E	TC020508.01	RBL08.001	E
TC040299.02	RBL99.275	B	TC020508.02	RBL08.002	E
TC040299.03	RBL99.276	R	TC020508.03	RBL08.003	A
TC040299.04	RBL99.277	F	TC020508.04	RBL08.004	E
TC040299.05	RBL99.278	U	TC020508.05	RBL08.005	S
TC040299.06	RBL99.279	E	TC020508.06	RBL08.006	E
TC040299.07	RBL99.280	E	TC020508.07	RBL08.007	E
TC040299.08	RBL99.281	F	TC020508.08	RBL08.008	E
TC040299.09	RBL99.282	N	TC020508.09	RBL08.009	E
TC040299.10	RBL99.283	E	TC020508.10	RBL08.010	F
TC040299.11	RBL99.284	N	TC020508.11	RBL08.011	E
TC040299.12	RBL99.285	E	TC020508.12	RBL08.012	E
TC040299.13	RBL99.286	E	TC020508.13	RBL08.013	E
TC040299.14	RBL99.287	E	TC020508.14	RBL08.014	E

TC040299.15	RBL99.288	E	TC020508.15	RBL08.015	A
TC040299.16	RBL99.289	E	TC020508.16	RBL08.016	E
TC040299.17	RBL99.290	E	TC020508.17	RBL08.017	A
TC040299.18	RBL99.291	E	TC020508.18	RBL08.018	B
TC040299.19	RBL99.292	B	TC020508.19	RBL08.019	B
TC040299.20	RBL99.293	S	TC020508.20	RBL08.020	E
TC040299.21	RBL99.294	R	TC020508.21	RBL08.021	E
TC040299.22	RBL99.295	R	TC020508.22	RBL08.022	E
TC040299.23	RBL99.296	R	TC020508.23	RBL08.023	E
TC040299.24	RBL99.297	E	TC020508.24	RBL08.024	B
TC040299.25	RBL99.298	U	TC020508.25	RBL08.025	E
HF02150001	RBL00.001	B	NM020508.01	RBL08.026	E
HF02150002	RBL00.002	E	NM020508.02	RBL08.027	S
HF02150003	RBL00.003	R	NM020508.03	RBL08.028	E
HF02150004	RBL00.004	F	NM020508.04	RBL08.029	E
HF02150005	RBL00.005	E	NM020508.05	RBL08.030	R
HF02150006	RBL00.006	E	NM020508.06	RBL08.031	E
HF02150007	RBL00.007	E	NM020508.07	RBL08.032	E
HF02150008	RBL00.008	E	NM020508.08	RBL08.033	E
HF02150009	RBL00.009	E	NM020508.09	RBL08.034	E
HF02150010	RBL00.010	E	NM020508.10	RBL08.035	E
HF02150011	RBL00.011	E	NM020508.11	RBL08.036	H
HF02150012	RBL00.012	B	NM020508.12	RBL08.037	G
HF02150013	RBL00.013	E	NM020508.13	RBL08.038	P
HF02150014	RBL00.014	B	NM020508.14	RBL08.039	E
HF02150015	RBL00.015	S	NM020508.15	RBL08.040	E
HF02150016	RBL00.016	S	NM020508.16	RBL08.041	E
HF02150017	RBL00.017	S	NM020508.17	RBL08.042	B
HF02150019	RBL00.018	S	NM020508.18	RBL08.043	B
HF02150020	RBL00.019	E	NM020508.19	RBL08.044	H
HF02150021	RBL00.021	E	NM020508.20	RBL08.045	E
HF02150022	RBL00.022	S	NM020508.21	RBL08.046	S
HF02150023	RBL00.023	S	NM020508.22	RBL08.047	Z
HF02150024	RBL00.024	B	NM020508.23	RBL08.048	E
HF02150025	RBL00.025	E	NM020508.24	RBL08.049	R
TC02150001	RBL00.026	A	NM020508.25	RBL08.050	C
TC02150002	RBL00.027	R	YC020608.01	RBL08.051	E
TC02150003	RBL00.028	B	YC020608.02	RBL08.052	F
TC02150004	RBL00.029	B	YC020608.03	RBL08.053	E
TC02150005	RBL00.030	R	YC020608.04	RBL08.054	E
TC02150006	RBL00.031	E	YC020608.05	RBL08.055	R

TC02150007	RBL00.032	E	YC020608.06	RBL08.056	B
TC02150008	RBL00.033	B	YC020608.07	RBL08.057	E
TC02150009	RBL00.034	B	YC020608.08	RBL08.058	E
TC02150010	RBL00.035	E	YC020608.09	RBL08.059	E
TC02150011	RBL00.036	R	YC020608.10	RBL08.060	E
TC02150012	RBL00.037	R	YC020608.11	RBL08.061	E
TC02150013	RBL00.038	A	YC020608.12	RBL08.062	R
TC02150015	RBL00.040	A	YC020608.13	RBL08.063	C
TC02150016	RBL00.041	E	YC020608.14	RBL08.064	E
TC02150017	RBL00.042	B	YC020608.15	RBL08.065	E
TC02150018	RBL00.043	E	YC020608.16	RBL08.066	R
TC02150019	RBL00.044	E	YC020608.17	RBL08.067	E
TC02150020	RBL00.045	B	YC020608.18	RBL08.068	E
TC02150021	RBL00.046	E	YC020608.19	RBL08.069	C
TC02150022	RBL00.047	E	YC020608.20	RBL08.070	E
TC02150023	RBL00.048	E	YC020608.21	RBL08.071	F
TC02150024	RBL00.049	B	YC020608.22	RBL08.072	S
TC02150025	RBL00.050	E	YC020608.23	RBL08.073	E
WC02150001	RBL00.051	E	YC020608.24	RBL08.074	E
WC02150002	RBL00.052	E	YC020608.25	RBL08.075	E
WC02150003	RBL00.053	J	YC021908.01	RBL08.076	E
WC02150004	RBL00.054	E	YC021908.02	RBL08.077	E
WC02150005	RBL00.055	E	YC021908.03	RBL08.078	R
YC02150001	RBL00.056	E	YC021908.04	RBL08.079	E
YC02150002	RBL00.057	E	YC021908.05	RBL08.080	K
YC02150003	RBL00.058	E	YC021908.06	RBL08.081	C
YC02150004	RBL00.059	B	YC021908.07	RBL08.082	H
YC02150005	RBL00.060	M	YC021908.08	RBL08.083	E
YC02150006	RBL00.061	E	YC021908.09	RBL08.084	E
YC02150007	RBL00.062	H	YC021908.10	RBL08.085	f
YC02150008	RBL00.063	M	YC021908.11	RBL08.086	R
YC02150009	RBL00.064	R	YC021908.12	RBL08.087	E
YC02150010	RBL00.065	E	YC021908.13	RBL08.088	A
YC02150011	RBL00.066	E	YC021908.14	RBL08.089	H
YC02150012	RBL00.067	E	YC021908.15	RBL08.090	E
YC02150013	RBL00.068	E	YC021908.16	RBL08.091	E
YC02150014	RBL00.069	E	YC021908.17	RBL08.092	E
YC02150015	RBL00.070	E	YC021908.18	RBL08.093	V
YC02150016	RBL00.071	E	YC021908.19	RBL08.094	E
YC02150017	RBL00.072	E	YC021908.20	RBL08.095	A
YC02150018	RBL00.073	E	YC021908.21	RBL08.096	E

YC02150019	RBL00.074	P	TC022008.01	RBL08.097	E
YC02150020	RBL00.075	H	TC022008.02	RBL08.098	E
YC02150021	RBL00.076	E	TC022008.03	RBL08.099	E
YC02150022	RBL00.077	E	TC022008.04	RBL08.100	E
YC02150023	RBL00.078	M	TC022008.05	RBL08.101	R
YC02150024	RBL00.079	M	TC022008.06	RBL08.102	E
YC02150025	RBL00.080	E	TC022008.07	RBL08.103	E
HF02290001	RBL00.081	U	TC022008.08	RBL08.104	E
HF02290002	RBL00.082	A	TC022008.09	RBL08.105	E
HF02290003	RBL00.083	E	TC022008.10	RBL08.106	A
HF02290004	RBL00.084	B	TC022008.11	RBL08.107	E
HF02290005	RBL00.085	A	TC022008.12	RBL08.108	A
HF02290006	RBL00.086	G	TC022008.13	RBL08.109	E
HF02290007	RBL00.087	E	TC022008.14	RBL08.110	E
HF02290008	RBL00.088	E	TC022008.15	RBL08.111	E
HF02290009	RBL00.089	G	TC022008.16	RBL08.112	E
HF02290010	RBL00.090	E	TC022008.17	RBL08.113	E
HF02290011	RBL00.091	B	TC022008.18	RBL08.114	E
HF02290012	RBL00.092	E	TC022008.19	RBL08.115	S
HF02290013	RBL00.093	E	TC022008.20	RBL08.116	E
HF02290014	RBL00.094	B	TC022008.21	RBL08.117	H
HF02290015	RBL00.095	E	TC022008.22	RBL08.118	E
HF02290016	RBL00.096	E	TC022008.23	RBL08.119	A
HF02290017	RBL00.097	F	TC022008.24	RBL08.120	S
HF02290018	RBL00.098	B	TC022008.25	RBL08.121	S
HF02290019	RBL00.099	E	NM022608.01	RBL08.122	A
HF02290020	RBL00.100	E	NM022608.02	RBL08.123	B
HF02290021	RBL00.101	E	NM022608.03	RBL08.124	E
HF02290022	RBL00.102	E	NM022608.04	RBL08.125	E
HF02290023	RBL00.103	T	NM022608.05	RBL08.126	B
HF02290024	RBL00.104	E	NM022608.06	RBL08.127	E
HF02290025	RBL00.105	E	NM022608.07	RBL08.128	K
TC02290001	RBL00.106	E	NM022608.08	RBL08.129	E
TC02290002	RBL00.107	A	NM022608.09	RBL08.130	E
TC02290003	RBL00.108	B	NM022608.10	RBL08.131	E
TC02290004	RBL00.109	F	NM022608.11	RBL08.132	E
TC02290005	RBL00.110	E	NM022608.12	RBL08.133	C
TC02290006	RBL00.111	E	NM022608.13	RBL08.134	E
TC02290007	RBL00.112	E	NM022608.14	RBL08.135	E
TC02290008	RBL00.113	A	NM022608.15	RBL08.136	E
TC02290009	RBL00.114	E	NM022608.16	RBL08.137	A

TC02290010	RBL00.115	E	NM022608.17	RBL08.138	C
TC02290011	RBL00.116	A	NM022608.18	RBL08.139	A
TC02290012	RBL00.117	E	NM022608.19	RBL08.140	E
TC02290013	RBL00.118	E	NM022608.20	RBL08.141	E
TC02290014	RBL00.119	S	NM022608.21	RBL08.142	E
TC02290015	RBL00.120	A	NM022608.22	RBL08.143	E
TC02290016	RBL00.121	E	NM022608.23	RBL08.144	F
TC02290017	RBL00.122	U	NM022608.24	RBL08.145	E
TC02290018	RBL00.123	S	NM022608.25	RBL08.146	E
TC02290019	RBL00.124	A	NM030308.01	RBL08.147	E
TC02290020	RBL00.125	S	NM030308.02	RBL08.148	E
TC02290021	RBL00.126	S	NM030308.03	RBL08.149	F
TC02290022	RBL00.127	J	NM030308.04	RBL08.150	E
TC02290023	RBL00.128	E	NM030308.05	RBL08.151	E
TC02290024	RBL00.129	E	NM030308.06	RBL08.152	E
TC02290025	RBL00.130	E	NM030308.07	RBL08.153	E
WC02290001	RBL00.131	V	NM030308.08	RBL08.154	E
WC02290002	RBL00.132	E	NM030308.09	RBL08.155	E
WC02290003	RBL00.133	E	NM030308.10	RBL08.156	E
WC02290004	RBL00.134	E	NM030308.11	RBL08.157	E
WC02290005	RBL00.135	E	NM030308.12	RBL08.158	A
WC02290006	RBL00.136	E	NM030308.13	RBL08.159	E
WC02290007	RBL00.137	R	NM030308.14	RBL08.160	E
WC02290008	RBL00.138	E	NM030308.15	RBL08.161	Z
WC02290009	RBL00.139	E	NM030308.16	RBL08.162	E
WC02290010	RBL00.140	M	NM030308.17	RBL08.163	A
WC02290011	RBL00.141	E	NM030308.18	RBL08.164	E
WC02290012	RBL00.142	E	NM030308.19	RBL08.165	J
WC02290013	RBL00.143	E	NM030308.20	RBL08.166	R
WC02290014	RBL00.144	E	NM030308.21	RBL08.167	E
WC02290015	RBL00.145	B	NM030308.22	RBL08.168	E
WC02290016	RBL00.146	E	NM030308.23	RBL08.169	P
WC02290017	RBL00.147	E	NM030308.24	RBL08.170	E
WC02290018	RBL00.148	E	NM030308.25	RBL08.171	A
WC02290019	RBL00.149	E	YC030408.01	RBL08.172	E
WC02290020	RBL00.150	M	YC030408.02	RBL08.173	S
WC02290021	RBL00.151	E	YC030408.03	RBL08.174	E
WC02290022	RBL00.152	E	YC030408.04	RBL08.175	E
WC02290023	RBL00.153	E	YC030408.05	RBL08.176	E
WC02290024	RBL00.154	E	YC030408.06	RBL08.177	E
WC02290025	RBL00.155	V	YC030408.07	RBL08.178	E

YC02290001	RBL00.156	E	YC030408.08	RBL08.179	E
YC02290002	RBL00.157	E	YC030408.09	RBL08.180	E
YC02290003	RBL00.158	B	YC030408.10	RBL08.181	E
YC02290004	RBL00.159	E	YC030408.11	RBL08.182	E
YC02290005	RBL00.160	E	YC030408.12	RBL08.183	E
YC02290006	RBL00.161	E	YC030408.13	RBL08.184	E
YC02290007	RBL00.162	E	YC030408.14	RBL08.185	E
YC02290008	RBL00.163	U	YC030408.15	RBL08.186	E
YC02290009	RBL00.164	B	YC030408.16	RBL08.187	E
YC02290010	RBL00.165	E	YC030408.17	RBL08.188	E
YC02290011	RBL00.166	E	YC030408.18	RBL08.189	E
YC02290012	RBL00.167	E	YC030408.19	RBL08.190	R
YC02290013	RBL00.168	E	YC030408.20	RBL08.191	E
YC02290014	RBL00.169	E	YC030408.21	RBL08.192	E
YC02290015	RBL00.170	E	YC030408.22	RBL08.193	S
YC02290016	RBL00.171	E	YC030408.23	RBL08.194	R
YC02290017	RBL00.172	B	YC030408.24	RBL08.195	E
YC02290018	RBL00.173	R	YC030408.25	RBL08.196	E
YC02290019	RBL00.174	E	TC030408.01	RBL08.197	C
YC02290020	RBL00.175	E	TC030408.02	RBL08.198	S
YC02290021	RBL00.176	E	TC030408.03	RBL08.199	E
YC02290022	RBL00.177	E	TC030408.04	RBL08.200	A
YC02290023	RBL00.178	E	TC030408.05	RBL08.201	E
YC02290024	RBL00.179	E	TC030408.06	RBL08.202	E
YC02290025	RBL00.180	B	TC030408.07	RBL08.203	C
HH03160001	RBL00.181	E	TC030408.08	RBL08.204	D
HH03160002	RBL00.182	B	TC030408.09	RBL08.205	J
HH03160003	RBL00.183	R	TC030408.10	RBL08.206	J
HH03160004	RBL00.184	E	TC030408.11	RBL08.207	E
HH03160005	RBL00.185	E	TC030408.12	RBL08.208	HH
HH03160006	RBL00.186	E	TC030408.13	RBL08.209	E
HH03160007	RBL00.187	R	TC030408.14	RBL08.210	D
HH03160008	RBL00.188	E	TC030408.15	RBL08.211	E
HH03160009	RBL00.189	B	TC030408.16	RBL08.212	E
HH03160010	RBL00.190	E	TC030408.17	RBL08.213	E
HH03160011	RBL00.191	E	TC030408.18	RBL08.214	E
HH03160012	RBL00.192	E	TC030408.19	RBL08.215	C
HH03160013	RBL00.193	R	TC030408.20	RBL08.216	E
HH03160014	RBL00.194	E	TC030408.21	RBL08.217	E
HH03160015	RBL00.195	H	TC030408.22	RBL08.218	E
HH03160016	RBL00.196	E	TC030408.23	RBL08.219	E

HH03160017	RBL00.197	E
HH03160018	RBL00.198	A
HH03160019	RBL00.199	E
HH03160020	RBL00.200	E
HH03160022	RBL00.202	S
HH03160023	RBL00.203	E
HH03160024	RBL00.204	E
TC03160001	RBL00.205	E
TC03160002	RBL00.206	P
TC03160003	RBL00.207	E
TC03160005	RBL00.209	E
TC03160006	RBL00.210	E
TC03160008	RBL00.212	E
TC03160009	RBL00.213	E
TC03160010	RBL00.214	A
TC03160011	RBL00.215	E
TC03160012	RBL00.216	E
TC03160013	RBL00.217	U
TC03160014	RBL00.218	E
TC03160015	RBL00.219	V
TC03160017	RBL00.221	E
TC03160018	RBL00.222	E
TC03160019	RBL00.223	E
TC03160020	RBL00.224	E
TC03160021	RBL00.225	E
TC03160022	RBL00.226	C
TC03160023	RBL00.227	E
TC03160024	RBL00.228	M
TC03160025	RBL00.229	E
WC03160001	RBL00.230	E
WC03160002	RBL00.231	E
WC03160003	RBL00.232	E
WC03160004	RBL00.233	C
WC03160005	RBL00.234	R
WC03160006	RBL00.235	B
WC03160007	RBL00.236	E
WC03160008	RBL00.237	V
WC03160009	RBL00.238	E
WC03160010	RBL00.239	E
WC03160011	RBL00.240	B
WC03160012	RBL00.241	B

TC030408.24	RBL08.220	J
TC030408.25	RBL08.221	E
NM031808.01	RBL08.222	E
NM031808.02	RBL08.223	E
NM031808.03	RBL08.224	A
NM031808.04	RBL08.225	E
NM031808.05	RBL08.226	E
NM031808.06	RBL08.227	S
NM031808.07	RBL08.228	S
NM031808.08	RBL08.229	E
NM031808.09	RBL08.230	E
NM031808.10	RBL08.231	F
NM031808.11	RBL08.232	E
NM031808.12	RBL08.233	E
NM031808.13	RBL08.234	E
NM031808.14	RBL08.235	I
NM031808.15	RBL08.236	E
NM031808.16	RBL08.237	E
NM031808.17	RBL08.238	E
NM031808.18	RBL08.239	E
NM031808.19	RBL08.240	E
NM031808.20	RBL08.241	P
NM031808.21	RBL08.242	P
NM031808.22	RBL08.243	E
NM031808.23	RBL08.244	R
NM031808.24	RBL08.245	I
NM031808.25	RBL08.246	E
TC031808.01	RBL08.247	E
TC031808.02	RBL08.248	E
TC031808.03	RBL08.249	R
TC031808.04	RBL08.250	E
TC031808.05	RBL08.251	R
TC031808.06	RBL08.252	R
TC031808.07	RBL08.253	R
TC031808.08	RBL08.254	G
TC031808.09	RBL08.255	E
TC031808.10	RBL08.256	E
TC031808.11	RBL08.257	E
TC031808.12	RBL08.258	E
TC031808.13	RBL08.259	R
TC031808.14	RBL08.260	E

WC03160013	RBL00.242	E	TC031808.15	RBL08.261	E
WC03160014	RBL00.243	R	TC031808.16	RBL08.262	E
WC03160015	RBL00.244	B	TC031808.17	RBL08.263	R
WC03160016	RBL00.245	S	TC031808.18	RBL08.264	E
WC03160017	RBL00.246	B	TC031808.19	RBL08.265	E
WC03160018	RBL00.247	B	TC031808.20	RBL08.266	E
WC03160019	RBL00.248	B	TC031808.21	RBL08.267	E
WC03160020	RBL00.249	E	TC031808.22	RBL08.268	R
WC03160021	RBL00.250	E	TC031808.23	RBL08.269	F
WC03160022	RBL00.251	E	TC031808.24	RBL08.270	E
WC03160023	RBL00.252	E	TC031808.25	RBL08.271	E
WC03160024	RBL00.253	J	YC031808.01	RBL08.272	E
YC03160001	RBL00.255	S	YC031808.02	RBL08.273	E
YC03160002	RBL00.256	E	YC031808.03	RBL08.274	C
YC03160003	RBL00.257	E	YC031808.04	RBL08.275	E
YC03160004	RBL00.258	E	YC031808.05	RBL08.276	E
YC03160005	RBL00.259	S	YC031808.06	RBL08.277	E
YC03160006	RBL00.260	E	YC031808.07	RBL08.278	E
YC03160007	RBL00.261	E	YC031808.08	RBL08.279	E
YC03160008	RBL00.262	E	YC031808.09	RBL08.280	E
YC03160009	RBL00.263	E	YC031808.10	RBL08.281	E
YC03160010	RBL00.264	S	YC031808.11	RBL08.282	E
YC03160011	RBL00.265	E	YC031808.12	RBL08.283	E
YC03160012	RBL00.266	R	YC031808.13	RBL08.284	E
YC03160013	RBL00.267	E	YC031808.14	RBL08.285	E
YC03160014	RBL00.268	S	YC031808.15	RBL08.286	HH
YC03160015	RBL00.269	E	YC031808.16	RBL08.287	E
YC03160016	RBL00.270	A	YC031808.17	RBL08.288	E
YC03160017	RBL00.271	S	YC031808.18	RBL08.289	B
YC03160018	RBL00.272	E	YC031808.19	RBL08.290	E
YC03160019	RBL00.273	S	YC031808.20	RBL08.291	E
YC03160020	RBL00.274	A	YC031808.21	RBL08.292	E
YC03160021	RBL00.275	A	YC031808.22	RBL08.293	F
YC03160022	RBL00.276	A	YC031808.23	RBL08.294	E
YC03160023	RBL00.277	E	YC031808.24	RBL08.295	A
YC03160024	RBL00.278	F	YC031808.25	RBL08.296	R
YC03160025	RBL00.279	E	NM033108.01	RBL08.297	A
HF03280001	RBL00.280	E	NM033108.02	RBL08.298	E
HF03280002	RBL00.281	E	NM033108.03	RBL08.299	E
HF03280003	RBL00.282	E	NM033108.04	RBL08.300	P
HF03280004	RBL00.283	E	NM033108.05	RBL08.301	E

HF03280005	RBL00.284	E	NM033108.06	RBL08.302	E
HF03280006	RBL00.285	E	NM033108.07	RBL08.303	V
HF03280007	RBL00.286	E	NM033108.08	RBL08.304	E
HF03280008	RBL00.287	E	NM033108.09	RBL08.305	E
HF03280009	RBL00.288	E	NM033108.10	RBL08.306	E
HF03280010	RBL00.289	E	NM033108.11	RBL08.307	V
HF03280011	RBL00.290	A	NM033108.12	RBL08.308	E
HF03280012	RBL00.291	R	NM033108.13	RBL08.309	E
HF03280013	RBL00.292	A	NM033108.14	RBL08.310	E
HF03280014	RBL00.293	A	NM033108.15	RBL08.311	C
HF03280015	RBL00.294	E	NM033108.16	RBL08.312	Z
HF03280016	RBL00.295	E	NM033108.17	RBL08.313	E
HF03280017	RBL00.296	S	NM033108.18	RBL08.314	E
HF03280018	RBL00.297	E	NM033108.19	RBL08.315	V
HF03280019	RBL00.298	S	NM033108.20	RBL08.316	V
HF03280020	RBL00.299	E	NM033108.21	RBL08.317	E
HF03280021	RBL00.300	P	NM033108.22	RBL08.318	E
HF03280022	RBL00.301	B	NM033108.23	RBL08.319	E
HF03280023	RBL00.302	B	NM033108.24	RBL08.320	E
HF03280024	RBL00.303	E	NM033108.25	RBL08.321	A
HF03280025	RBL00.304	E	TC033108.01	RBL08.322	E
TC03280001	RBL00.305	E	TC033108.02	RBL08.323	E
TC03280002	RBL00.306	E	TC033108.03	RBL08.324	E
TC03280003	RBL00.307	E	TC033108.04	RBL08.325	E
TC03280004	RBL00.308	E	TC033108.05	RBL08.326	E
TC03280005	RBL00.309	E	TC033108.06	RBL08.327	E
TC03280006	RBL00.310	E	TC033108.07	RBL08.328	E
TC03280007	RBL00.311	W	TC033108.08	RBL08.329	E
TC03280008	RBL00.312	S	TC033108.09	RBL08.330	E
TC03280009	RBL00.313	A	TC033108.10	RBL08.331	R
TC03280010	RBL00.314	E	TC033108.11	RBL08.332	P
TC03280011	RBL00.315	E	TC033108.12	RBL08.333	R
TC03280012	RBL00.316	E	TC033108.13	RBL08.334	B
TC03280013	RBL00.317	E	TC033108.14	RBL08.335	R
TC03280014	RBL00.318	Q	TC033108.15	RBL08.336	E
TC03280015	RBL00.319	E	TC033108.16	RBL08.337	E
TC03280016	RBL00.320	E	TC033108.17	RBL08.338	B
TC03280017	RBL00.321	G	TC033108.18	RBL08.339	E
TC03280018	RBL00.322	U	TC033108.19	RBL08.340	C
TC03280019	RBL00.323	E	TC033108.20	RBL08.341	E
TC03280020	RBL00.324	E	TC033108.21	RBL08.342	S

TC03280021	RBL00.325	E	TC033108.22	RBL08.343	E
TC03280022	RBL00.326	E	TC033108.23	RBL08.344	E
TC03280023	RBL00.327	E	TC033108.24	RBL08.345	E
TC03280024	RBL00.328	E	TC033108.25	RBL08.346	E
TC03280025	RBL00.329	E	YC040108.01	RBL08.347	E
WC03280001	RBL00.330	E	YC040108.02	RBL08.348	E
WC03280002	RBL00.331	B	YC040108.03	RBL08.349	E
WC03280003	RBL00.332	E	YC040108.04	RBL08.350	E
WC03280004	RBL00.333	E	YC040108.05	RBL08.351	E
WC03280005	RBL00.334	B	YC040108.06	RBL08.352	E
WC03280006	RBL00.335	B	YC040108.07	RBL08.353	E
WC03280007	RBL00.336	E	YC040108.08	RBL08.354	O
WC03280008	RBL00.337	B	YC040108.09	RBL08.355	E
WC03280009	RBL00.338	E	YC040108.10	RBL08.356	E
WC03280010	RBL00.339	E	YC040108.11	RBL08.357	E
WC03280011	RBL00.340	E	YC040108.12	RBL08.358	E
WC03280012	RBL00.341	E	YC040108.13	RBL08.359	E
WC03280013	RBL00.342	E	YC040108.14	RBL08.360	E
WC03280014	RBL00.343	E	YC040108.15	RBL08.361	E
WC03280015	RBL00.344	R	YC040108.16	RBL08.362	E
WC03280016	RBL00.345	E	YC040108.17	RBL08.363	E
WC03280017	RBL00.346	E	YC040108.18	RBL08.364	E
WC03280018	RBL00.347	E	YC040108.19	RBL08.365	E
WC03280019	RBL00.348	B	YC040108.20	RBL08.366	E
YC03280001	RBL00.349	M	YC040108.21	RBL08.367	E
YC03280002	RBL00.350	E	YC040108.22	RBL08.368	E
YC03280003	RBL00.351	E	YC040108.23	RBL08.369	E
YC03280004	RBL00.352	R	YC040108.24	RBL08.370	E
YC03280005	RBL00.353	E	YC040108.25	RBL08.371	E
YC03280006	RBL00.354	B	WC040308.01	RBL08.372	BB
YC03280007	RBL00.355	E	WC040308.02	RBL08.373	U
YC03280008	RBL00.356	E	WC040308.03	RBL08.374	A
YC03280009	RBL00.357	C	WC040308.04	RBL08.375	E
YC03280010	RBL00.358	E	WC040308.05	RBL08.376	E
YC03280011	RBL00.359	E	WC040308.06	RBL08.377	A
YC03280012	RBL00.360	E	WC040308.07	RBL08.378	E
YC03280013	RBL00.361	E	WC040308.08	RBL08.379	R
YC03280014	RBL00.362	E	WC040308.09	RBL08.380	E
YC03280015	RBL00.363	E	WC040308.10	RBL08.381	B
YC03280016	RBL00.364	E	WC040308.11	RBL08.382	E
YC03280017	RBL00.365	E	WC040308.12	RBL08.383	A

YC03280018	RBL00.366	E
YC03280019	RBL00.367	E
YC03280020	RBL00.368	E
YC03280021	RBL00.369	E
YC03280022	RBL00.370	E
YC03280023	RBL00.371	E
YC03280024	RBL00.372	C
YC03280025	RBL00.373	E
WC03050101	RBL01.001	B
WC03050102	RBL01.002	B
WC03050103	RBL01.003	B
WC03050104	RBL01.004	B
WC03050105	RBL01.005	E
WC03050106	RBL01.006	U
WC03050107	RBL01.007	E
WC03050108	RBL01.008	U
WC03050109	RBL01.009	B
WC03050110	RBL01.010	B
WC03050112	RBL01.012	E
WC03050113	RBL01.013	B
WC03050114	RBL01.014	E
WC03050115	RBL01.015	B
WC03050116	RBL01.016	E
WC03050117	RBL01.017	B
WC03050118	RBL01.018	E
WC03050119	RBL01.019	E
WC03050120	RBL01.020	E
WC03050121	RBL01.021	S
WC03050122	RBL01.022	B
WC03050123	RBL01.023	E
WC03050124	RBL01.024	B
WC03050125	RBL01.025	B
NM03060101	RBL01.026	E
NM03060102	RBL01.027	E
NM03060103	RBL01.028	E
NM03060104	RBL01.029	O
NM03060105	RBL01.030	E
NM03060106	RBL01.031	E
NM03060107	RBL01.032	A
NM03060108	RBL01.033	R
NM03060109	RBL01.034	B
WC040308.13	RBL08.384	F
WC040308.14	RBL08.385	E
WC040308.15	RBL08.386	E
WC040308.16	RBL08.387	E
WC040308.17	RBL08.388	S
WC040308.18	RBL08.389	J
WC040308.19	RBL08.390	BB
WC040308.20	RBL08.391	E
WC040308.21	RBL08.392	E
WC040308.22	RBL08.393	J
WC040308.23	RBL08.394	A
WC040308.24	RBL08.395	R
WC040308.25	RBL08.396	R
YC041408.01	RBL08.397	B
YC041408.02	RBL08.398	E
YC041408.03	RBL08.399	A
YC041408.04	RBL08.400	E
YC041408.05	RBL08.401	A
YC041408.06	RBL08.402	P
YC041408.07	RBL08.403	P
YC041408.08	RBL08.404	A
YC041408.09	RBL08.405	E
YC041408.10	RBL08.406	P
YC041408.11	RBL08.407	E
YC041408.12	RBL08.408	P
YC041408.13	RBL08.409	P
YC041408.14	RBL08.410	E
YC041408.15	RBL08.411	R
YC041408.16	RBL08.412	A
YC041408.17	RBL08.413	A
YC041408.18	RBL08.414	E
YC041408.19	RBL08.415	E
YC041408.20	RBL08.416	E
YC041408.21	RBL08.417	A
YC041408.22	RBL08.418	P
WC041408.01	RBL08.419	E
WC041408.02	RBL08.420	E
WC041408.03	RBL08.421	E
WC041408.04	RBL08.422	S
WC041408.05	RBL08.423	R
WC041408.06	RBL08.424	R

NM03060110	RBL01.035	B	WC041408.07	RBL08.425	E
NM03060111	RBL01.036	E	WC041408.08	RBL08.426	R
NM03060112	RBL01.037	E	WC041408.09	RBL08.427	B
NM03060113	RBL01.038	E	WC041408.10	RBL08.428	E
NM03060114	RBL01.039	E	WC041408.11	RBL08.429	R
NM03060115	RBL01.040	E	WC041408.12	RBL08.430	E
NM03060116	RBL01.041	E	WC041408.13	RBL08.431	B
NM03060117	RBL01.042	E	WC041408.14	RBL08.432	S
NM03060118	RBL01.043	E	WC041408.15	RBL08.433	E
NM03060119	RBL01.044	O	WC041408.16	RBL08.434	B
NM03060120	RBL01.045	E	WC041408.17	RBL08.435	E
NM03060121	RBL01.046	E	WC041408.18	RBL08.436	E
NM03060122	RBL01.047	E	WC041408.19	RBL08.437	E
NM03060123	RBL01.048	E	WC041408.20	RBL08.438	B
NM03060124	RBL01.049	E	WC041408.21	RBL08.439	E
NM03060125	RBL01.050	A	WC041408.22	RBL08.440	R
TC03150101	RBL01.051	S	WC041408.23	RBL08.441	E
TC03150102	RBL01.052	E	WC041408.24	RBL08.442	E
TC03150103	RBL01.053	E	WC041408.25	RBL08.443	E
TC03150104	RBL01.054	E	TC041508.01	RBL08.444	E
TC03150105	RBL01.055	E	TC041508.02	RBL08.445	E
TC03150106	RBL01.056	E	TC041508.03	RBL08.446	E
TC03150107	RBL01.057	E	TC041508.04	RBL08.447	E
TC03150108	RBL01.058	S	TC041508.05	RBL08.448	S
TC03150109	RBL01.059	R	TC041508.06	RBL08.449	E
TC03150110	RBL01.060	E	TC041508.07	RBL08.450	E
TC03150111	RBL01.061	E	TC041508.08	RBL08.451	E
TC03150112	RBL01.062	E	TC041508.09	RBL08.452	S
TC03150113	RBL01.063	E	TC041508.10	RBL08.453	E
TC03150114	RBL01.064	E	TC041508.11	RBL08.454	E
TC03150115	RBL01.065	S	TC041508.12	RBL08.455	S
TC03150116	RBL01.066	E	TC041508.13	RBL08.456	S
TC03150117	RBL01.067	R	TC041508.14	RBL08.457	E
TC03150118	RBL01.068	S	TC041508.15	RBL08.458	S
TC03150119	RBL01.069	B	TC041508.16	RBL08.459	S
TC03150120	RBL01.070	S	TC041508.17	RBL08.460	S
TC03150121	RBL01.071	E	TC041508.18	RBL08.461	S
TC03150122	RBL01.072	E	TC041508.19	RBL08.462	E
TC03150123	RBL01.073	E	TC041508.20	RBL08.463	E
TC03150124	RBL01.074	E	TC041508.22	RBL08.465	S
TC03150125	RBL01.075	E	TC041508.23	RBL08.466	E

TC03150126	RBL01.076	A
WC03150101	RBL01.077	S
WC03150102	RBL01.078	B
WC03150103	RBL01.079	E
WC03150104	RBL01.080	E
WC03150105	RBL01.081	E
WC03150106	RBL01.082	B
WC03150107	RBL01.083	E
WC03150108	RBL01.084	E
WC03150109	RBL01.085	E
WC03150110	RBL01.086	E
WC03150111	RBL01.087	E
WC03150112	RBL01.088	E
WC03150113	RBL01.089	E
WC03150114	RBL01.090	E
WC03150115	RBL01.091	V
WC03150116	RBL01.092	E
WC03150117	RBL01.093	S
WC03150118	RBL01.094	E
WC03150119	RBL01.095	E
WC03150120	RBL01.096	R
WC03150121	RBL01.097	E
WC03150122	RBL01.098	B
WC03150123	RBL01.099	F
WC03150124	RBL01.100	E
WC03150125	RBL01.101	E
CL0101	RBL01.102	E
CL0102	RBL01.103	P
CL0103	RBL01.104	E
CL0104	RBL01.105	E
CL0105	RBL01.106	E
CL0106	RBL01.107	K
CL0107	RBL01.108	B
CL0108	RBL01.109	B
CL0109	RBL01.110	E
CL0110	RBL01.111	K
CL0111	RBL01.112	B
CL0112	RBL01.113	B
CL0113	RBL01.114	S
CL0114	RBL01.115	E
CL0116	RBL01.117	E
TC041508.24	RBL08.467	S
TC041508.25	RBL08.468	S
NM042908.01	RBL08.469	E
NM042908.02	RBL08.470	E
NM042908.03	RBL08.471	E
NM042908.04	RBL08.472	E
NM042908.05	RBL08.473	S
NM042908.06	RBL08.474	E
NM042908.07	RBL08.475	E
NM042908.08	RBL08.476	E
NM042908.09	RBL08.477	E
NM042908.10	RBL08.478	S
NM042908.11	RBL08.479	E
NM042908.12	RBL08.480	E
NM042908.13	RBL08.481	E
NM042908.14	RBL08.482	E
NM042908.15	RBL08.483	E
NM042908.16	RBL08.484	E
NM042908.17	RBL08.485	E
NM042908.18	RBL08.486	E
NM042908.19	RBL08.487	E
NM042908.20	RBL08.488	E
NM042908.21	RBL08.489	B
NM042908.22	RBL08.490	E
NM042908.23	RBL08.491	E
NM042908.24	RBL08.492	E
NM042908.25	RBL08.493	E
WC043008.01	RBL08.494	E
WC043008.02	RBL08.495	E
WC043008.03	RBL08.496	E
WC043008.04	RBL08.497	E
WC043008.05	RBL08.498	S
WC043008.06	RBL08.499	E
WC043008.07	RBL08.500	E
WC043008.08	RBL08.501	S
WC043008.09	RBL08.502	A
WC043008.10	RBL08.503	S
WC043008.11	RBL08.504	E
WC043008.12	RBL08.505	B
WC043008.13	RBL08.506	B
WC043008.14	RBL08.507	E

CL0117	RBL01.118	E	WC043008.15	RBL08.508	E
CL0118	RBL01.119	E	WC043008.16	RBL08.509	R
CL0119	RBL01.120	U	WC043008.17	RBL08.510	A
CL0120	RBL01.121	U	WC043008.18	RBL08.511	E
CL0121	RBL01.122	R	WC043008.19	RBL08.512	G
CL0122	RBL01.123	E	WC043008.20	RBL08.513	E
CL0123	RBL01.124	E	WC043008.21	RBL08.514	E
CL0124	RBL01.125	E	WC043008.22	RBL08.515	E
WC0101	RBL01.126	E	WC043008.23	RBL08.516	B
WC0102	RBL01.127	E	WC043008.24	RBL08.517	B
WC0103	RBL01.128	B	WC043008.25	RBL08.518	B
WC0104	RBL01.129	K	YC043008.01	RBL08.520	A
WC0105	RBL01.130	E	YC043008.02	RBL08.521	E
WC040201.01	RBL01.131	R	YC043008.03	RBL08.522	E
WC040201.02	RBL01.132	F	YC043008.04	RBL08.523	S
WC040201.03	RBL01.133	E	YC043008.05	RBL08.524	E
WC040201.04	RBL01.134	E	YC043008.06	RBL08.525	A
WC040201.05	RBL01.135	V	YC043008.07	RBL08.526	E
WC040201.06	RBL01.136	S	YC043008.08	RBL08.527	E
WC040201.07	RBL01.137	F	YC043008.09	RBL08.528	A
WC040201.08	RBL01.138	B	YC043008.10	RBL08.529	R
WC040201.09	RBL01.139	B	YC043008.11	RBL08.530	E
WC040201.10	RBL01.140	E	YC043008.12	RBL08.531	A
WC040201.11	RBL01.141	E	YC043008.13	RBL08.532	E
WC040201.12	RBL01.142	V	YC043008.14	RBL08.533	S
WC040201.13	RBL01.143	S	YC043008.15	RBL08.534	S
WC040201.14	RBL01.144	E	YC043008.16	RBL08.535	A
WC040201.15	RBL01.145	F	YC043008.17	RBL08.536	A
WC040201.16	RBL01.146	F	YC043008.18	RBL08.537	A
WC040201.17	RBL01.147	V	YC043008.19	RBL08.538	E
WC040201.18	RBL01.148	F	YC043008.20	RBL08.539	E
WC040201.19	RBL01.149	E	YC043008.21	RBL08.540	E
WC040201.20	RBL01.150	E	YC043008.22	RBL08.541	E
WC040201.21	RBL01.151	E	YC043008.23	RBL08.542	K
WC040201.22	RBL01.152	F	YC043008.24	RBL08.543	K
WC040201.23	RBL01.153	V	YC043008.25	RBL08.544	E
WC040201.24	RBL01.154	E	LL031008.01	RBL08.545	E
WC040201.25	RBL01.155	M	LL031008.02	RBL08.546	E
TC040901.01	RBL01.156	E	LL031008.03	RBL08.547	E
TC040901.02	RBL01.157	E	LL031008.04	RBL08.548	R
TC040901.03	RBL01.158	R	LL031008.05	RBL08.549	E

TC040901.04	RBL01.159	E	LL031008.06	RBL08.550	E
TC040901.05	RBL01.160	E	LL031008.07	RBL08.551	E
TC040901.06	RBL01.161	E	LL031008.08	RBL08.552	R
TC040901.07	RBL01.162	E	LL031008.09	RBL08.553	R
TC040901.08	RBL01.163	E	LL031008.10	RBL08.554	J
TC040901.09	RBL01.164	E	LL031008.11	RBL08.555	E
TC040901.10	RBL01.165	E	LL031008.12	RBL08.556	E
TC040901.11	RBL01.166	R	LL031008.13	RBL08.557	E
TC040901.12	RBL01.167	E	LL031008.14	RBL08.558	E
TC040901.13	RBL01.168	E	LL031008.15	RBL08.559	S
TC040901.14	RBL01.169	E	LL031008.16	RBL08.560	E
TC040901.15	RBL01.170	J	LL031008.17	RBL08.561	E
TC040901.16	RBL01.171	S	LL031008.18	RBL08.562	E
TC040901.17	RBL01.172	S	LL031008.19	RBL08.563	E
TC040901.18	RBL01.173	E	LL031008.20	RBL08.564	E
TC040901.19	RBL01.174	E	LL031008.21	RBL08.565	CC
TC040901.20	RBL01.175	E	LL031008.22	RBL08.566	E
TC040901.21	RBL01.176	E	LL031008.23	RBL08.567	E
TC040901.22	RBL01.177	B	LL031008.24	RBL08.568	R
TC040901.23	RBL01.178	E	LL031008.25	RBL08.569	B
TC040901.24	RBL01.179	E	LL031008.26	RBL08.570	E
TC040901.25	RBL01.180	R	LL031008.27	RBL08.571	B
TC040901.26	RBL01.181	E	WC051208.01	RBL08.572	E
TC040901.27	RBL01.182	E	WC051208.02	RBL08.573	E
YC040901.01	RBL01.183	E	WC051208.03	RBL08.574	E
YC040901.02	RBL01.184	E	WC051208.04	RBL08.575	E
YC040901.03	RBL01.185	E	WC051208.05	RBL08.576	E
YC040901.04	RBL01.186	E	WC051208.06	RBL08.577	R
YC040901.05	RBL01.187	E	WC051208.07	RBL08.578	E
YC040901.06	RBL01.188	E	WC051208.08	RBL08.579	E
YC040901.07	RBL01.189	E	TC051408.01	RBL08.607	E
YC040901.08	RBL01.190	E	TC051408.02	RBL08.608	E
YC040901.09	RBL01.191	E	TC051408.03	RBL08.609	A
YC040901.10	RBL01.192	E	TC051408.04	RBL08.610	S
YC040901.11	RBL01.193	B	TC051408.05	RBL08.611	R
YC040901.12	RBL01.194	A	TC051408.06	RBL08.612	E
YC040901.13	RBL01.195	E	TC051408.07	RBL08.613	E
YC040901.14	RBL01.196	E	TC051408.08	RBL08.614	B
YC040901.15	RBL01.197	E	TC051408.09	RBL08.615	A
YC040901.16	RBL01.198	B	TC051408.10	RBL08.616	B
YC040901.17	RBL01.199	E	TC051408.11	RBL08.617	R

YC040901.18	RBL01.200	E	TC051408.12	RBL08.618	C
YC040901.19	RBL01.201	E	TC051408.13	RBL08.619	E
YC040901.20	RBL01.202	E	TC051408.14	RBL08.620	S
YC040901.21	RBL01.203	E	TC051408.15	RBL08.621	S
YC040901.22	RBL01.204	E	TC051408.16	RBL08.622	S
YC040901.23	RBL01.205	E	TC051408.17	RBL08.623	E
YC040901.24	RBL01.206	E	TC051508.01	RBL08.624	A
YC040901.25	RBL01.207	E	TC051508.02	RBL08.625	A
HF041201.01	RBL01.208	A	TC051508.03	RBL08.626	R
HF041201.02	RBL01.209	E	TC051508.04	RBL08.627	E
HF041201.03	RBL01.210	S	TC051508.05	RBL08.628	E
HF041201.04	RBL01.211	B	TC051508.06	RBL08.629	E
HF041201.05	RBL01.212	B	TC051508.07	RBL08.630	B
HF041201.06	RBL01.213	B	TC051508.08	RBL08.631	B
HF041201.07	RBL01.214	S	HF020609.01	RBL09.001	S
HF041201.08	RBL01.215	E	HF020609.02	RBL09.002	S
HF041201.09	RBL01.216	E	HF020609.03	RBL09.003	BB
HF041201.10	RBL01.217	E	HF020609.04	RBL09.004	E
HF041201.11	RBL01.218	R	HF020609.05	RBL09.005	E
HF041201.12	RBL01.219	S	HF020609.06	RBL09.006	S
HF041201.13	RBL01.220	S	HF020609.07	RBL09.007	E
HF041201.14	RBL01.221	B	HF020609.08	RBL09.008	E
HF041201.15	RBL01.222	B	HF020609.09	RBL09.009	E
HF041201.16	RBL01.223	E	HF020609.10	RBL09.010	E
HF041201.17	RBL01.224	E	HF020609.11	RBL09.011	B
HF041201.18	RBL01.225	E	HF020609.12	RBL09.012	E
HF041201.19	RBL01.226	E	HF020609.13	RBL09.013	B
HF041201.20	RBL01.227	Y	HF020609.14	RBL09.014	E
HF041201.21	RBL01.228	R	HF020609.15	RBL09.015	K
HF041201.22	RBL01.229	E	HF020609.16	RBL09.016	S
HF041201.23	RBL01.230	S	HF020609.17	RBL09.017	A
HF041201.24	RBL01.231	S	HF020609.18	RBL09.018	E
HF041201.25	RBL01.232	S	HF020609.19	RBL09.019	C
HH21402.01	RBL02.001	A	HF020609.20	RBL09.020	E
HH21402.02	RBL02.002	E	HF020609.21	RBL09.021	E
HH21402.03	RBL02.003	E	HF020609.22	RBL09.022	B
HH21402.04	RBL02.004	E	HF020609.23	RBL09.023	E
HH21402.05	RBL02.005	E	HF020609.24	RBL09.024	C
HH21402.06	RBL02.006	E	HF020609.25	RBL09.025	E
HH21402.07	RBL02.007	E	TC020609.01	RBL09.026	B
HH21402.08	RBL02.008	P	TC020609.02	RBL09.027	B

HH21402.09	RBL02.009	S	TC020609.03	RBL09.028	A
HH21402.10	RBL02.010	E	TC020609.04	RBL09.029	B
HH21402.11	RBL02.011	E	TC020609.05	RBL09.030	E
HH21402.12	RBL02.012	E	TC020609.06	RBL09.031	B
HH21402.13	RBL02.013	E	TC020609.07	RBL09.032	B
HH21402.14	RBL02.014	E	TC020609.08	RBL09.033	E
HH21402.15	RBL02.015	P	TC020609.09	RBL09.034	B
HH21402.16	RBL02.016	E	TC020609.10	RBL09.035	E
HH21402.17	RBL02.017	E	TC020609.11	RBL09.036	B
HH21402.18	RBL02.018	E	TC020609.12	RBL09.037	B
HH21402.19	RBL02.019	E	TC020609.13	RBL09.038	E
HH21402.20	RBL02.020	S	TC020609.14	RBL09.039	A
HH21402.21	RBL02.021	E	TC020609.15	RBL09.040	E
HH21402.22	RBL02.022	E	TC020609.16	RBL09.041	B
HH21402.23	RBL02.023	E	TC020609.17	RBL09.042	B
HH21402.24	RBL02.024	E	TC020609.18	RBL09.043	A
HH21402.25	RBL02.025	E	TC020609.19	RBL09.044	B
HH21402.26	RBL02.026	E	TC020609.20	RBL09.045	B
HH21402.27	RBL02.027	E	TC020609.21	RBL09.046	A
HH21402.28	RBL02.028	E	TC020609.22	RBL09.047	A
HH21402.29	RBL02.029	E	TC020609.23	RBL09.048	A
HH21402.30	RBL02.030	E	TC020609.24	RBL09.049	B
HH21402.31	RBL02.031	E	TC020609.25	RBL09.050	B
YC21402.01	RBL02.032	E	YC020609.01	RBL09.051	E
YC21402.02	RBL02.033	E	YC020609.02	RBL09.052	E
YC21402.03	RBL02.034	E	YC020609.03	RBL09.053	R
YC21402.04	RBL02.035	A	YC020609.04	RBL09.054	E
YC21402.05	RBL02.036	E	YC020609.05	RBL09.055	E
YC21402.06	RBL02.037	M	YC020609.06	RBL09.056	E
YC21402.07	RBL02.038	E	YC020609.07	RBL09.057	E
YC21402.08	RBL02.039	M	YC020609.08	RBL09.058	E
YC21402.09	RBL02.040	E	YC020609.09	RBL09.059	E
YC21402.10	RBL02.041	E	YC020609.11	RBL09.061	F
YC21402.11	RBL02.042	E	YC020609.12	RBL09.062	B
YC21402.12	RBL02.043	E	YC020609.13	RBL09.063	E
YC21402.13	RBL02.044	E	YC020609.14	RBL09.064	E
YC21402.16	RBL02.047	F	YC020609.15	RBL09.065	S
YC21402.17	RBL02.048	E	YC020609.16	RBL09.066	E
YC21402.18	RBL02.049	E	YC020609.17	RBL09.067	E
YC21402.19	RBL02.050	E	YC020609.18	RBL09.068	E
YC21402.20	RBL02.051	F	YC020609.19	RBL09.069	F

YC21402.21	RBL02.052	E	YC020609.20	RBL09.070	E
YC21402.22	RBL02.053	E	YC020609.21	RBL09.071	E
YC21402.23	RBL02.054	E	YC020609.22	RBL09.072	E
YC21402.24	RBL02.055	E	LC021709.01	RBL09.075	S
YC21402.25	RBL02.056	E	LC021709.02	RBL09.076	S
HH22602.01	RBL02.057	B	LC021709.03	RBL09.077	E
HH22602.02	RBL02.058	E	LC021709.04	RBL09.078	S
HH22602.03	RBL02.059	E	LC021709.05	RBL09.079	B
HH22602.04	RBL02.060	E	LC021709.06	RBL09.080	C
HH22602.05	RBL02.061	E	LC021709.07	RBL09.081	E
HH22602.06	RBL02.062	F	LC021709.08	RBL09.082	E
HH22602.07	RBL02.063	E	LC021709.09	RBL09.083	E
HH22602.08	RBL02.064	S	LC021709.10	RBL09.084	E
HH22602.09	RBL02.065	E	LC021709.11	RBL09.085	B
HH22602.10	RBL02.066	E	LC021709.12	RBL09.086	A
HH22602.11	RBL02.067	E	LC021709.13	RBL09.087	E
HH22602.12	RBL02.068	E	LC021709.14	RBL09.088	R
HH22602.13	RBL02.069	B	LC021709.15	RBL09.089	C
HH22602.14	RBL02.070	S	LC021709.16	RBL09.090	E
HH22602.15	RBL02.071	B	LC021709.17	RBL09.091	A
HH22602.16	RBL02.072	E	LC021709.18	RBL09.092	E
HH22602.17	RBL02.073	E	LC021709.19	RBL09.093	S
HH22602.18	RBL02.074	S	LC021709.20	RBL09.094	K
HH22602.19	RBL02.075	E	LC021709.21	RBL09.095	A
HH22602.20	RBL02.076	E	LC021709.22	RBL09.096	K
HH22602.21	RBL02.077	E	LC021709.23	RBL09.097	A
HH22602.22	RBL02.078	B	LC021709.24	RBL09.098	E
HH22602.23	RBL02.079	E	LC021709.25	RBL09.099	K
TC22602.01	RBL02.080	E	TB021709.01	RBL09.100	E
TC22602.02	RBL02.081	E	TB021709.02	RBL09.101	Z
TC22602.03	RBL02.082	E	TB021709.03	RBL09.102	E
TC22602.04	RBL02.083	S	TB021709.04	RBL09.103	E
TC22602.05	RBL02.084	E	TB021709.05	RBL09.104	R
TC22602.07	RBL02.086	S	TB021709.06	RBL09.105	R
TC22602.08	RBL02.087	E	TB021709.07	RBL09.106	E
TC22602.09	RBL02.088	E	TB021709.08	RBL09.107	Z
TC22602.10	RBL02.089	F	TB021709.09	RBL09.108	B
TC22602.11	RBL02.090	P	TB021709.10	RBL09.109	Z
TC22602.12	RBL02.091	R	TB021709.11	RBL09.110	E
TC22602.13	RBL02.092	E	TB021709.12	RBL09.111	B
TC22602.14	RBL02.093	E	TB021709.13	RBL09.112	E

TC22602.15	RBL02.094	B	TB021709.14	RBL09.113	E
TC22602.16	RBL02.095	E	TB021709.15	RBL09.114	E
TC22602.17	RBL02.096	P	TB021709.16	RBL09.115	Z
TC22602.18	RBL02.097	E	TB021709.17	RBL09.116	E
TC22602.19	RBL02.098	E	TB021709.18	RBL09.117	S
TC22602.21	RBL02.100	B	TB021709.19	RBL09.118	E
TC22602.22	RBL02.101	B	TB021709.20	RBL09.119	Z
TC22602.23	RBL02.102	E	TB021709.21	RBL09.120	Z
TC22602.24	RBL02.103	E	TB021709.22	RBL09.121	Z
YC22602.01	RBL02.104	E	TB021709.23	RBL09.122	R
YC22602.02	RBL02.105	S	TB021709.24	RBL09.123	Z
YC22602.03	RBL02.106	E	TB021709.25	RBL09.124	BB
YC22602.04	RBL02.107	E	YC021709.01	RBL09.125	E
YC22602.05	RBL02.108	B	YC021709.02	RBL09.126	F
YC22602.08	RBL02.111	B	YC021709.03	RBL09.127	E
YC22602.09	RBL02.112	E	YC021709.04	RBL09.128	H
YC22602.10	RBL02.113	E	YC021709.05	RBL09.129	E
YC22602.11	RBL02.114	E	YC021709.06	RBL09.130	S
YC22602.12	RBL02.115	E	YC021709.07	RBL09.131	E
YC22602.13	RBL02.116	E	YC021709.08	RBL09.132	S
YC22602.14	RBL02.117	E	YC021709.09	RBL09.133	E
YC22602.15	RBL02.118	B	YC021709.10	RBL09.134	E
YC22602.16	RBL02.119	R	YC021709.11	RBL09.135	S
YC22602.17	RBL02.120	E	YC021709.12	RBL09.136	E
YC22602.18	RBL02.121	E	YC021709.13	RBL09.137	E
YC22602.19	RBL02.122	E	YC021709.14	RBL09.138	R
YC22602.20	RBL02.123	E	YC021709.15	RBL09.139	R
YC22602.21	RBL02.124	E	YC021709.16	RBL09.140	E
YC22602.22	RBL02.125	E	YC021709.17	RBL09.141	E
YC22602.23	RBL02.126	E	YC021709.18	RBL09.142	E
YC22602.24	RBL02.127	J	YC021709.19	RBL09.143	E
YC22602.25	RBL02.128	B	YC021709.20	RBL09.144	E
TC31102.01	RBL02.129	E	YC021709.21	RBL09.145	E
TC31102.02	RBL02.130	C	YC021709.22	RBL09.146	E
TC31102.03	RBL02.131	E	YC021709.23	RBL09.147	E
TC31102.04	RBL02.132	B	YC021709.24	RBL09.148	E
TC31102.05	RBL02.133	A	YC021709.25	RBL09.149	E
TC31102.06	RBL02.134	E	TC030209.01	RBL09.150	E
TC31102.08	RBL02.136	E	TC030209.02	RBL09.151	E
TC31102.09	RBL02.137	A	TC030209.03	RBL09.152	E
TC31102.10	RBL02.138	B	TC030209.04	RBL09.153	E

TC31102.11	RBL02.139	R	TC030209.05	RBL09.154	C
TC31102.12	RBL02.140	R	TC030209.06	RBL09.155	E
TC31102.13	RBL02.141	E	TC030209.07	RBL09.156	E
TC31102.14	RBL02.142	E	TC030209.08	RBL09.157	E
TC31102.15	RBL02.143	E	TC030209.09	RBL09.158	E
TC31102.16	RBL02.144	E	TC030209.10	RBL09.159	E
TC31102.17	RBL02.145	R	TC030209.11	RBL09.160	E
TC31102.18	RBL02.146	E	TC030209.12	RBL09.161	E
TC31102.19	RBL02.147	E	TC030209.13	RBL09.162	R
TC31102.20	RBL02.148	F	TC030209.14	RBL09.163	E
TC31102.21	RBL02.149	E	TC030209.15	RBL09.164	B
TC31102.22	RBL02.150	R	TC030209.16	RBL09.165	E
TC31102.23	RBL02.151	E	TC030209.17	RBL09.166	E
TC31102.24	RBL02.152	E	TC030209.18	RBL09.167	S
TC31102.25	RBL02.153	E	TC030209.19	RBL09.168	E
TC31102.26	RBL02.154	R	TC030209.20	RBL09.169	S
NM31202.01	RBL02.155	E	TC030209.21	RBL09.170	B
NM31202.02	RBL02.156	E	TC030209.22	RBL09.171	E
NM31202.03	RBL02.157	R	TC030209.23	RBL09.172	E
NM31202.04	RBL02.158	S	TC030209.24	RBL09.173	R
NM31202.05	RBL02.159	E	TC030209.25	RBL09.174	E
NM31202.06	RBL02.160	R	YC030209.01	RBL09.175	E
NM31202.07	RBL02.161	E	YC030209.02	RBL09.176	E
NM31202.08	RBL02.162	C	YC030209.03	RBL09.177	R
NM31202.09	RBL02.163	R	YC030209.04	RBL09.178	V
NM31202.10	RBL02.164	E	YC030209.05	RBL09.179	S
NM31202.11	RBL02.165	H	YC030209.06	RBL09.180	S
NM31202.12	RBL02.166	S	YC030209.07	RBL09.181	S
NM31202.13	RBL02.167	E	YC030209.08	RBL09.182	B
NM31202.14	RBL02.168	E	YC030209.09	RBL09.183	R
NM31202.15	RBL02.169	E	YC030209.10	RBL09.184	S
NM31202.16	RBL02.170	E	YC030209.11	RBL09.185	S
NM31202.17	RBL02.171	E	YC030209.12	RBL09.186	E
NM31202.18	RBL02.172	B	YC030209.13	RBL09.187	E
NM31202.19	RBL02.173	F	YC030209.14	RBL09.188	A
NM31202.20	RBL02.174	E	YC030209.15	RBL09.189	Q
NM31202.21	RBL02.175	B	YC030209.16	RBL09.190	S
NM31202.22	RBL02.176	E	YC030209.17	RBL09.191	E
NM31202.23	RBL02.177	B	YC030209.18	RBL09.192	S
NM31202.24	RBL02.178	R	YC030209.19	RBL09.193	R
YC31202.01	RBL02.179	E	YC030209.20	RBL09.194	B

YC31202.02	RBL02.180	E	YC030209.23	RBL09.197	R
YC31202.03	RBL02.181	A	YC030209.24	RBL09.198	E
YC31202.04	RBL02.182	E	YC030209.25	RBL09.199	R
YC31202.05	RBL02.183	E	HF030309.01	RBL09.200	E
YC31202.06	RBL02.184	E	HF030309.02	RBL09.201	B
YC31202.07	RBL02.185	E	HF030309.03	RBL09.202	E
YC31202.08	RBL02.186	A	HF030309.04	RBL09.203	B
YC31202.09	RBL02.187	E	HF030309.05	RBL09.204	E
YC31202.10	RBL02.188	E	HF030309.06	RBL09.205	R
YC31202.11	RBL02.189	E	HF030309.07	RBL09.206	E
YC31202.12	RBL02.190	B	HF030309.08	RBL09.207	E
YC31202.13	RBL02.191	E	HF030309.09	RBL09.208	E
YC31202.14	RBL02.192	E	HF030309.10	RBL09.209	S
YC31202.15	RBL02.193	E	HF030309.11	RBL09.210	B
YC31202.16	RBL02.194	J	HF030309.12	RBL09.211	E
YC31202.18	RBL02.196	E	HF030309.13	RBL09.212	E
YC31202.19	RBL02.197	E	HF030309.14	RBL09.213	S
YC31202.20	RBL02.198	E	HF030309.15	RBL09.214	B
YC31202.21	RBL02.199	F	HF030309.16	RBL09.215	E
YC31202.22	RBL02.200	E	HF030309.17	RBL09.216	B
YC31202.23	RBL02.201	A	HF030309.18	RBL09.217	E
YC31202.24	RBL02.202	E	HF030309.19	RBL09.218	A
YC31202.25	RBL02.203	A	HF030309.20	RBL09.219	E
WC32502.01	RBL02.204	R	HF030309.21	RBL09.220	E
WC32502.02	RBL02.205	E	HF030309.22	RBL09.221	E
WC32502.03	RBL02.206	E	HF030309.23	RBL09.222	E
WC32502.04	RBL02.207	A	HF030309.24	RBL09.223	E
WC32502.05	RBL02.208	A	HF030309.25	RBL09.224	B
WC32502.06	RBL02.209	E	TC031609.01	RBL09.225	E
WC32502.07	RBL02.210	E	TC031609.02	RBL09.226	F
WC32502.08	RBL02.211	E	TC031609.03	RBL09.227	G
WC32502.09	RBL02.212	E	TC031609.04	RBL09.228	E
WC32502.10	RBL02.213	E	TC031609.05	RBL09.229	E
WC32502.11	RBL02.214	E	TC031609.06	RBL09.230	B
WC32502.12	RBL02.215	R	TC031609.07	RBL09.231	B
WC32502.13	RBL02.216	E	TC031609.08	RBL09.232	G
WC32502.14	RBL02.217	E	TC031609.09	RBL09.233	E
WC32502.15	RBL02.218	E	TC031609.10	RBL09.234	E
WC32502.16	RBL02.219	E	TC031609.11	RBL09.235	E
WC32502.17	RBL02.220	F	TC031609.12	RBL09.236	B
WC32502.18	RBL02.221	E	TC031609.13	RBL09.237	G

WC32502.19	RBL02.222	E	TC031609.14	RBL09.238	E
WC32502.20	RBL02.223	B	TC031609.15	RBL09.239	B
WC32502.22	RBL02.225	E	TC031609.16	RBL09.240	E
WC32502.23	RBL02.226	E	TC031609.17	RBL09.241	E
WC32502.24	RBL02.227	E	TC031609.18	RBL09.242	E
WC32502.25	RBL02.228	E	TC031609.19	RBL09.243	E
TC32702.01	RBL02.229	E	TC031609.20	RBL09.244	E
TC32702.02	RBL02.230	A	TC031609.21	RBL09.245	E
TC32702.03	RBL02.231	R	TC031609.22	RBL09.246	B
TC32702.04	RBL02.232	E	TC031609.23	RBL09.247	E
TC32702.05	RBL02.233	S	TC031609.24	RBL09.248	E
TC32702.06	RBL02.234	E	TC031609.25	RBL09.249	B
TC32702.07	RBL02.235	E	WR031709.01	RBL09.250	E
TC32702.08	RBL02.236	E	WR031709.02	RBL09.251	B
TC32702.09	RBL02.237	E	WR031709.03	RBL09.252	R
TC32702.10	RBL02.238	C	WR031709.04	RBL09.253	A
TC32702.11	RBL02.239	E	WR031709.05	RBL09.254	E
TC32702.12	RBL02.240	E	WR031709.06	RBL09.255	B
TC32702.13	RBL02.241	E	WR031709.07	RBL09.256	E
TC32702.14	RBL02.242	S	WR031709.08	RBL09.257	A
TC32702.15	RBL02.243	S	WR031709.09	RBL09.258	E
TC32702.16	RBL02.244	S	WR031709.10	RBL09.259	E
TC32702.17	RBL02.245	E	WR031709.11	RBL09.260	E
TC32702.18	RBL02.246	S	WR031709.12	RBL09.261	B
TC32702.19	RBL02.247	E	WR031709.13	RBL09.262	E
TC32702.20	RBL02.248	E	WR031709.14	RBL09.263	R
TC32702.21	RBL02.249	S	WR031709.15	RBL09.264	A
TC32702.22	RBL02.250	E	WR031709.16	RBL09.265	E
TC32702.23	RBL02.251	C	WR031709.17	RBL09.266	B
TC32702.24	RBL02.252	E	WR031709.18	RBL09.267	J
TC32702.25	RBL02.253	E	WR031709.19	RBL09.268	A
YC32902.01	RBL02.254	S	WR031709.20	RBL09.269	E
YC32902.02	RBL02.255	E	WR031709.21	RBL09.270	E
YC32902.03	RBL02.256	E	WR031709.22	RBL09.271	H
YC32902.04	RBL02.257	E	WR031709.23	RBL09.272	E
YC32902.05	RBL02.258	B	WR031709.24	RBL09.273	E
YC32902.06	RBL02.259	E	WR031709.25	RBL09.274	E
YC32902.07	RBL02.260	E	VC031809.01	RBL09.275	E
YC32902.08	RBL02.261	E	VC031809.02	RBL09.276	E
YC32902.09	RBL02.262	E	VC031809.03	RBL09.277	E
YC32902.10	RBL02.263	E	VC031809.04	RBL09.278	E

YC32902.11	RBL02.264	E	VC031809.05	RBL09.279	B
YC32902.12	RBL02.265	E	VC031809.06	RBL09.280	R
YC32902.13	RBL02.266	P	VC031809.07	RBL09.281	E
YC32902.14	RBL02.267	E	VC031809.08	RBL09.282	E
YC32902.15	RBL02.268	S	VC031809.09	RBL09.283	E
YC32902.16	RBL02.269	E	VC031809.10	RBL09.284	E
YC32902.17	RBL02.270	F	VC031809.11	RBL09.285	E
YC32902.18	RBL02.271	A	VC031809.12	RBL09.286	E
YC32902.19	RBL02.272	E	VC031809.13	RBL09.287	B
YC32902.20	RBL02.273	S	VC031809.14	RBL09.288	B
YC32902.21	RBL02.274	K	VC031809.15	RBL09.289	P
YC32902.22	RBL02.275	R	VC031809.16	RBL09.290	E
YC32902.23	RBL02.276	E	VC031809.17	RBL09.291	E
YC32902.24	RBL02.277	A	VC031809.18	RBL09.292	S
YC32902.25	RBL02.278	E	VC031809.19	RBL09.293	E
WC40502.01	RBL02.279	E	VC031809.20	RBL09.294	E
WC40502.02	RBL02.280	E	VC031809.21	RBL09.295	E
WC40502.03	RBL02.281	E	VC031809.22	RBL09.296	R
WC40502.04	RBL02.282	E	VC031809.23	RBL09.297	E
WC40502.05	RBL02.283	S	VC031809.24	RBL09.298	P
WC40502.06	RBL02.284	E	VC031809.25	RBL09.299	E
WC40502.07	RBL02.285	E	YC031809.01	RBL09.300	E
WC40502.08	RBL02.286	E	YC031809.02	RBL09.301	E
WC40502.09	RBL02.287	R	YC031809.03	RBL09.302	F
WC40502.10	RBL02.288	E	YC031809.04	RBL09.303	E
WC40502.11	RBL02.289	E	YC031809.05	RBL09.304	E
WC40502.12	RBL02.290	E	YC031809.06	RBL09.305	J
WC40502.13	RBL02.291	E	YC031809.07	RBL09.306	L
WC40502.14	RBL02.292	E	YC031809.08	RBL09.307	E
WC40502.15	RBL02.293	E	YC031809.09	RBL09.308	E
WC40502.16	RBL02.294	E	YC031809.10	RBL09.309	E
WC40502.17	RBL02.295	E	YC031809.11	RBL09.310	P
WC40502.18	RBL02.296	E	YC031809.12	RBL09.311	E
WC40502.19	RBL02.297	R	YC031809.13	RBL09.312	E
WC40502.20	RBL02.298	E	YC031809.14	RBL09.313	E
WC40502.21	RBL02.299	E	YC031809.15	RBL09.314	S
WC40502.22	RBL02.300	E	YC031809.16	RBL09.315	E
WC40502.23	RBL02.301	E	YC031809.17	RBL09.316	E
WC40502.24	RBL02.302	E	YC031809.18	RBL09.317	B
WC40502.25	RBL02.303	E	YC031809.19	RBL09.318	E
HF40902.01	RBL02.304	S	YC031809.20	RBL09.319	P

HF40902.02	RBL02.305	E	YC031809.21	RBL09.320	E
HF40902.03	RBL02.306	E	YC031809.22	RBL09.321	E
HF40902.04	RBL02.307	A	YC031809.23	RBL09.322	L
HF40902.05	RBL02.308	E	YC031809.24	RBL09.323	P
HF40902.06	RBL02.309	E	YC031809.25	RBL09.324	E
HF40902.07	RBL02.310	E	CC032009.01	RBL09.325	E
HF40902.08	RBL02.311	E	CC032009.02	RBL09.326	B
HF40902.09	RBL02.312	E	CC032009.03	RBL09.327	R
HF40902.10	RBL02.313	E	CC032009.04	RBL09.328	E
HF40902.11	RBL02.314	V	CC032009.05	RBL09.329	E
HF40902.12	RBL02.315	E	CC032009.06	RBL09.330	E
HF40902.13	RBL02.316	G	CC032009.07	RBL09.331	B
HF40902.14	RBL02.317	S	CC032009.08	RBL09.332	E
HF40902.15	RBL02.318	E	CC032009.09	RBL09.333	E
HF40902.16	RBL02.319	S	CC032009.10	RBL09.334	E
HF40902.17	RBL02.320	E	CC032009.11	RBL09.335	J
HF40902.18	RBL02.321	E	CC032009.12	RBL09.336	E
HF40902.19	RBL02.322	E	CC032009.13	RBL09.337	F
HF40902.20	RBL02.323	E	CC032009.14	RBL09.338	E
HF40902.21	RBL02.324	E	CC032009.15	RBL09.339	E
HF40902.22	RBL02.325	E	CC032009.16	RBL09.340	E
HF40902.23	RBL02.326	E	CC032009.17	RBL09.341	CC
HF40902.24	RBL02.327	E	CC032009.18	RBL09.342	P
HF40902.25	RBL02.328	E	CC032009.19	RBL09.343	J
YC41002.01	RBL02.329	E	CC032009.20	RBL09.344	G
YC41002.02	RBL02.330	R	CC032009.21	RBL09.345	A
YC41002.03	RBL02.331	E	CC032009.22	RBL09.346	E
YC41002.04	RBL02.332	E	CC032009.23	RBL09.347	B
YC41002.05	RBL02.333	E	VC033109.01	RBL09.348	E
YC41002.06	RBL02.334	E	VC033109.02	RBL09.349	B
YC41002.07	RBL02.335	E	VC033109.03	RBL09.350	E
YC41002.08	RBL02.336	E	VC033109.04	RBL09.351	E
YC41002.09	RBL02.337	E	VC033109.05	RBL09.352	A
YC41002.10	RBL02.338	E	VC033109.06	RBL09.353	E
YC41002.11	RBL02.339	E	VC033109.07	RBL09.354	E
YC41002.12	RBL02.340	E	VC033109.08	RBL09.355	E
YC41002.13	RBL02.341	R	VC033109.09	RBL09.356	E
YC41002.14	RBL02.342	E	VC033109.10	RBL09.357	E
YC41002.15	RBL02.343	R	VC033109.11	RBL09.358	E
YC41002.16	RBL02.344	C	VC033109.12	RBL09.359	B
YC41002.17	RBL02.345	E	VC033109.13	RBL09.360	E

YC41002.18	RBL02.346	R	VC033109.14	RBL09.361	E
YC41002.19	RBL02.347	P	VC033109.15	RBL09.362	E
YC41002.20	RBL02.348	E	VC033109.16	RBL09.363	E
YC41002.21	RBL02.349	C	VC033109.17	RBL09.364	E
YC41002.22	RBL02.350	E	VC033109.18	RBL09.365	B
YC41002.23	RBL02.351	E	VC033109.19	RBL09.366	E
YC41002.24	RBL02.352	R	VC033109.20	RBL09.367	R
YC41002.25	RBL02.353	E	VC033109.21	RBL09.368	E
NM21203.01	RBL03.001	E	VC033109.22	RBL09.369	E
NM21203.02	RBL03.002	E	VC033109.23	RBL09.370	E
NM21203.03	RBL03.003	R	VC033109.24	RBL09.371	E
NM21203.04	RBL03.004	E	TC033109.01	RBL09.372	E
NM21203.05	RBL03.005	E	TC033109.02	RBL09.373	E
NM21203.06	RBL03.006	E	TC033109.03	RBL09.374	E
NM21203.07	RBL03.007	E	TC033109.04	RBL09.375	R
NM21203.08	RBL03.008	E	TC033109.05	RBL09.376	E
NM21203.09	RBL03.009	E	TC033109.06	RBL09.377	R
NM21203.10	RBL03.010	R	TC033109.07	RBL09.378	E
NM21203.11	RBL03.011	E	TC033109.08	RBL09.379	E
NM21203.12	RBL03.012	E	TC033109.09	RBL09.380	E
NM21203.13	RBL03.013	R	TC033109.10	RBL09.381	E
NM21203.14	RBL03.014	R	TC033109.11	RBL09.382	E
NM21203.15	RBL03.015	E	TC033109.12	RBL09.383	R
NM21203.16	RBL03.016	E	TC033109.13	RBL09.384	R
NM21203.17	RBL03.017	K	TC033109.14	RBL09.385	E
NM21203.18	RBL03.018	E	TC033109.15	RBL09.386	E
NM21203.19	RBL03.019	E	TC033109.16	RBL09.387	E
NM21203.20	RBL03.020	B	TC033109.17	RBL09.388	R
NM21203.21	RBL03.021	E	TC033109.18	RBL09.389	E
NM21203.22	RBL03.022	E	TC033109.19	RBL09.390	E
NM21203.23	RBL03.023	R	TC033109.20	RBL09.391	E
NM21203.24	RBL03.024	E	TC033109.21	RBL09.392	E
NM21203.25	RBL03.025	K	TC033109.22	RBL09.393	E
NM22603.01	RBL03.026	K	TC033109.23	RBL09.394	R
NM22603.02	RBL03.027	E	TC033109.24	RBL09.395	E
NM22603.03	RBL03.028	E	TC033109.25	RBL09.396	E
NM22603.04	RBL03.029	B	LC033109.01	RBL09.397	R
NM22603.05	RBL03.030	B	LC033109.02	RBL09.398	E
NM22603.06	RBL03.031	B	LC033109.03	RBL09.399	B
NM22603.07	RBL03.032	E	LC033109.04	RBL09.400	E
NM22603.08	RBL03.033	B	LC033109.05	RBL09.401	E

NM22603.09	RBL03.034	I	LC033109.06	RBL09.402	C
NM22603.10	RBL03.035	R	LC033109.07	RBL09.403	E
NM22603.11	RBL03.036	E	LC033109.08	RBL09.404	S
NM22603.12	RBL03.037	E	LC033109.09	RBL09.405	E
NM22603.13	RBL03.038	G	LC033109.10	RBL09.406	B
NM22603.14	RBL03.039	E	LC033109.11	RBL09.407	E
NM22603.15	RBL03.040	F	LC033109.12	RBL09.408	E
NM22603.16	RBL03.041	B	LC033109.13	RBL09.409	E
NM22603.17	RBL03.042	F	LC033109.14	RBL09.410	B
NM22603.18	RBL03.043	S	LC033109.15	RBL09.411	M
NM22603.19	RBL03.044	E	LC033109.16	RBL09.412	B
NM22603.20	RBL03.045	E	LC033109.17	RBL09.413	E
NM22603.21	RBL03.046	G	LC033109.18	RBL09.414	S
NM22603.22	RBL03.047	A	LC033109.19	RBL09.415	B
NM22603.23	RBL03.048	B	LC033109.20	RBL09.416	Q
NM22603.24	RBL03.049	E	LC033109.21	RBL09.417	E
NM22603.25	RBL03.050	S	LC033109.22	RBL09.418	E
NM31203.01	RBL03.051	E	LC033109.23	RBL09.419	S
NM31203.02	RBL03.052	S	LC033109.24	RBL09.420	S
NM31203.03	RBL03.053	E	YC033109.01	RBL09.421	R
NM31203.04	RBL03.054	B	YC033109.02	RBL09.422	G
NM31203.05	RBL03.055	A	YC033109.03	RBL09.423	R
NM31203.06	RBL03.056	E	YC033109.04	RBL09.424	E
NM31203.07	RBL03.057	S	YC033109.05	RBL09.425	E
NM31203.08	RBL03.058	E	YC033109.06	RBL09.426	R
NM31203.09	RBL03.059	E	YC033109.07	RBL09.427	S
NM31203.10	RBL03.060	E	YC033109.08	RBL09.428	E
NM31203.11	RBL03.061	B	YC033109.09	RBL09.429	R
NM31203.12	RBL03.062	E	YC033109.10	RBL09.430	R
NM31203.13	RBL03.063	E	YC033109.11	RBL09.431	B
NM31203.14	RBL03.064	R	YC033109.12	RBL09.432	R
NM31203.15	RBL03.065	A	YC033109.13	RBL09.433	C
NM31203.16	RBL03.066	E	YC033109.14	RBL09.434	E
NM31203.17	RBL03.067	R	YC033109.15	RBL09.435	R
NM31203.18	RBL03.068	B	YC033109.16	RBL09.436	E
NM31203.19	RBL03.069	E	YC033109.17	RBL09.437	E
NM31203.20	RBL03.070	U	YC033109.18	RBL09.438	E
NM31203.21	RBL03.071	E	YC033109.19	RBL09.439	G
NM31203.22	RBL03.072	E	YC033109.20	RBL09.440	E
NM31203.23	RBL03.073	E	YC033109.21	RBL09.441	R
NM31203.24	RBL03.074	E	YC033109.22	RBL09.442	G

NM31203.25	RBL03.075	E	YC033109.23	RBL09.443	G
NM32603.01	RBL03.076	E	YC033109.24	RBL09.444	B
NM32603.02	RBL03.077	C	YC033109.25	RBL09.445	E
NM32603.03	RBL03.078	E	LS041309.01	RBL09.446	S
NM32603.04	RBL03.079	E	LS041309.02	RBL09.447	E
NM32603.05	RBL03.080	E	LS041309.03	RBL09.448	B
NM32603.06	RBL03.081	Q	LS041309.04	RBL09.449	E
NM32603.07	RBL03.082	E	LS041309.05	RBL09.450	E
NM32603.08	RBL03.083	G	LS041309.06	RBL09.451	E
NM32603.09	RBL03.084	S	LS041309.07	RBL09.452	R
NM32603.10	RBL03.085	E	LS041309.08	RBL09.453	E
NM32603.11	RBL03.086	C	LS041309.09	RBL09.454	F
NM32603.12	RBL03.087	E	LS041309.10	RBL09.455	E
NM32603.13	RBL03.088	E	LS041309.11	RBL09.456	E
NM32603.14	RBL03.089	A	LS041309.12	RBL09.457	E
NM32603.15	RBL03.090	K	LS041309.13	RBL09.458	E
NM32603.16	RBL03.091	E	LS041309.14	RBL09.459	C
NM32603.17	RBL03.092	S	LS041309.15	RBL09.460	R
NM32603.18	RBL03.093	E	LS041309.16	RBL09.461	E
NM32603.19	RBL03.094	E	LS041309.17	RBL09.462	E
NM32603.20	RBL03.095	E	LS041309.18	RBL09.463	F
NM32603.21	RBL03.096	E	LS041309.19	RBL09.464	G
NM32603.22	RBL03.097	E	LS041309.20	RBL09.465	E
NM32603.23	RBL03.098	E	LS041309.21	RBL09.466	E
NM32603.24	RBL03.099	S	LS041309.22	RBL09.467	E
NM32603.25	RBL03.100	E	LS041309.23	RBL09.468	S
WC32903.01	RBL03.101	B	LS041309.24	RBL09.469	F
WC32903.02	RBL03.102	E	LS041309.25	RBL09.470	E
WC32903.03	RBL03.103	E	TC041309.01	RBL09.471	R
WC32903.04	RBL03.104	E	TC041309.02	RBL09.472	E
WC32903.05	RBL03.105	E	TC041309.03	RBL09.473	R
WC32903.06	RBL03.106	E	TC041309.04	RBL09.474	I
WC32903.07	RBL03.107	E	TC041309.05	RBL09.475	A
WC32903.08	RBL03.108	E	TC041309.06	RBL09.476	R
WC32903.09	RBL03.109	E	TC041309.07	RBL09.477	A
WC32903.10	RBL03.110	E	TC041309.08	RBL09.478	I
WC32903.11	RBL03.111	E	TC041309.09	RBL09.479	R
WC32903.12	RBL03.112	E	TC041309.10	RBL09.480	I
WC32903.13	RBL03.113	E	TC041309.11	RBL09.481	I
WC32903.14	RBL03.114	E	TC041309.12	RBL09.482	R
WC32903.15	RBL03.115	E	TC041309.13	RBL09.483	R

WC32903.16	RBL03.116	E	TC041309.14	RBL09.484	E
WC32903.17	RBL03.117	E	TC041309.15	RBL09.485	E
WC32903.18	RBL03.118	E	TC041309.16	RBL09.486	E
WC32903.19	RBL03.119	E	TC041309.17	RBL09.487	R
WC32903.20	RBL03.120	E	TC041309.18	RBL09.488	I
WC32903.21	RBL03.121	E	TC041309.19	RBL09.489	A
WC32903.22	RBL03.122	E	TC041309.20	RBL09.490	R
WC32903.23	RBL03.123	E	TC041309.21	RBL09.491	R
WC32903.24	RBL03.124	E	TC041309.22	RBL09.492	R
WC32903.25	RBL03.125	E	TC041309.23	RBL09.493	E
TC21103.01	RBL03.126	E	TC041309.24	RBL09.494	E
TC21103.02	RBL03.127	E	TC041309.25	RBL09.495	I
TC21103.03	RBL03.128	E	YC041709.01	RBL09.496	E
TC21103.04	RBL03.129	E	YC041709.02	RBL09.497	A
TC21103.05	RBL03.130	A	YC041709.03	RBL09.498	E
TC21103.06	RBL03.131	A	YC041709.04	RBL09.499	A
TC21103.07	RBL03.132	E	YC041709.05	RBL09.500	A
TC21103.08	RBL03.133	E	YC041709.06	RBL09.501	E
TC21103.09	RBL03.134	A	YC041709.07	RBL09.502	A
TC21103.10	RBL03.135	A	YC041709.08	RBL09.503	E
TC21103.11	RBL03.136	E	YC041709.09	RBL09.504	E
TC21103.12	RBL03.137	E	YC041709.10	RBL09.505	S
TC21103.13	RBL03.138	E	YC041709.11	RBL09.506	E
TC21103.14	RBL03.139	E	YC041709.12	RBL09.507	A
TC21103.15	RBL03.140	E	YC041709.13	RBL09.508	G
TC21103.16	RBL03.141	A	YC041709.14	RBL09.509	G
TC21103.17	RBL03.142	E	YC041709.15	RBL09.510	A
TC21103.18	RBL03.143	E	YC041709.16	RBL09.511	S
TC21103.19	RBL03.144	A	YC041709.17	RBL09.512	G
TC21103.20	RBL03.145	E	YC041709.18	RBL09.513	A
TC21103.21	RBL03.146	E	YC041709.19	RBL09.514	S
TC21103.22	RBL03.147	S	YC041709.20	RBL09.515	G
TC21103.23	RBL03.148	S	YC041709.21	RBL09.516	G
TC21103.24	RBL03.149	E	YC041709.22	RBL09.517	E
TC21103.25	RBL03.150	S	YC041709.23	RBL09.518	A
TC22603.01	RBL03.151	E	YC041709.24	RBL09.519	G
TC22603.02	RBL03.152	A	YC041709.25	RBL09.520	E
TC22603.03	RBL03.153	B	TC020110.01	RBL10.001	A
TC22603.04	RBL03.154	E	TC020110.02	RBL10.002	E
TC22603.05	RBL03.155	E	TC020110.03	RBL10.003	B
TC22603.06	RBL03.156	E	TC020110.04	RBL10.004	B

TC22603.07	RBL03.157	E	TC020110.05	RBL10.005	E
TC22603.08	RBL03.158	S	TC020110.06	RBL10.006	B
TC22603.09	RBL03.159	B	TC020110.07	RBL10.007	B
TC22603.10	RBL03.160	F	TC020110.08	RBL10.008	B
TC22603.11	RBL03.161	J	TC020110.09	RBL10.009	E
TC22603.12	RBL03.162	S	TC020110.10	RBL10.010	E
TC22603.14	RBL03.164	E	TC020110.11	RBL10.011	B
TC22603.15	RBL03.165	A	TC020110.12	RBL10.012	E
TC22603.16	RBL03.166	S	TC020110.13	RBL10.013	E
TC22603.17	RBL03.167	S	TC020110.14	RBL10.014	E
TC22603.18	RBL03.168	S	TC020110.15	RBL10.015	B
TC22603.19	RBL03.169	E	TC020110.16	RBL10.016	B
TC22603.20	RBL03.170	S	TC020110.17	RBL10.017	B
TC22603.21	RBL03.171	A	TC020110.18	RBL10.018	B
TC22603.22	RBL03.172	E	TC020110.19	RBL10.019	E
TC22603.23	RBL03.173	A	TC020110.20	RBL10.020	B
TC22603.24	RBL03.174	E	TC020110.21	RBL10.021	E
TC22603.25	RBL03.175	S	TC020110.22	RBL10.022	B
TC32403.01	RBL03.176	E	TC020110.23	RBL10.023	B
TC32403.02	RBL03.177	M	TC020110.24	RBL10.024	B
TC32403.03	RBL03.178	R	TC020110.25	RBL10.025	E
TC32403.04	RBL03.179	A	TC020110.01	RBL10.026	E
TC32403.05	RBL03.180	C	TC020110.02	RBL10.027	E
TC32403.06	RBL03.181	E	TC020110.03	RBL10.028	R
TC32403.07	RBL03.182	E	TC020110.04	RBL10.029	E
TC32403.08	RBL03.183	E	TC020110.05	RBL10.030	E
TC32403.09	RBL03.184	S	TC020110.06	RBL10.031	E
TC32403.10	RBL03.185	E	TC020110.07	RBL10.032	R
TC32403.11	RBL03.186	E	TC020110.08	RBL10.033	A
TC32403.12	RBL03.187	R	TC020110.09	RBL10.034	E
TC32403.13	RBL03.188	S	TC020110.10	RBL10.035	E
TC32403.14	RBL03.189	E	TC020110.11	RBL10.036	E
TC32403.15	RBL03.190	E	TC020110.12	RBL10.037	E
TC32403.16	RBL03.191	E	TC020110.13	RBL10.038	E
TC32403.17	RBL03.192	E	TC020110.14	RBL10.039	E
TC32403.18	RBL03.193	E	TC020110.15	RBL10.040	E
TC32403.19	RBL03.194	E	TC020110.16	RBL10.041	E
TC32403.20	RBL03.195	E	TC020110.17	RBL10.042	E
TC32403.21	RBL03.196	E	TC020110.18	RBL10.043	E
TC32403.22	RBL03.197	E	TC020110.19	RBL10.044	B
TC32403.23	RBL03.198	G	TC020110.20	RBL10.045	E

TC32403.24	RBL03.199	E	TC020110.21	RBL10.046	R
TC32403.25	RBL03.200	E	TC020110.22	RBL10.047	S
TC32603.01	RBL03.201	S	TC020110.23	RBL10.048	E
TC32603.02	RBL03.202	E	TC020110.24	RBL10.049	E
TC32603.03	RBL03.203	E	TC020110.25	RBL10.050	E
TC32603.04	RBL03.204	B	HH020310.01	RBL10.051	E
TC32603.05	RBL03.205	E	HH020310.02	RBL10.052	C
TC32603.06	RBL03.206	B	HH020310.03	RBL10.053	F
TC32603.07	RBL03.207	B	HH020310.04	RBL10.054	E
TC32603.08	RBL03.208	Q	HH020310.05	RBL10.055	B
TC32603.09	RBL03.209	S	HH020310.06	RBL10.056	E
TC32603.10	RBL03.210	B	HH020310.07	RBL10.057	E
TC32603.11	RBL03.211	B	HH020310.08	RBL10.058	A
TC32603.12	RBL03.212	E	HH020310.09	RBL10.059	H
TC32603.13	RBL03.213	E	HH020310.10	RBL10.060	F
TC32603.14	RBL03.214	X	HH020310.11	RBL10.061	E
TC32603.15	RBL03.215	Q	HH020310.12	RBL10.062	E
TC32603.16	RBL03.216	E	HH020310.13	RBL10.063	E
TC32603.17	RBL03.217	E	HH020310.14	RBL10.064	H
TC32603.18	RBL03.218	E	HH020310.15	RBL10.065	C
TC32603.19	RBL03.219	E	HH020310.16	RBL10.066	E
TC32603.20	RBL03.220	B	HH020310.17	RBL10.067	C
TC32603.21	RBL03.221	B	HH020310.18	RBL10.068	H
TC32603.22	RBL03.222	B	HH020310.19	RBL10.069	I
TC32603.23	RBL03.223	A	HH020310.20	RBL10.070	E
TC32603.24	RBL03.224	E	HH020310.21	RBL10.071	C
TC32603.25	RBL03.225	E	HH020310.22	RBL10.072	E
TC32603.26	RBL03.226	W	HH020310.23	RBL10.073	B
TC32603.27	RBL03.227	B	HH020310.24	RBL10.074	E
TC32603.28	RBL03.228	E	HH020310.25	RBL10.075	E
TC32603.29	RBL03.229	X	HW021610.01	RBL10.076	C
TC32603.30	RBL03.230	E	HW021610.02	RBL10.077	A
TC32603.31	RBL03.231	A	HW021610.03	RBL10.078	E
TC32603.32	RBL03.232	B	HW021610.04	RBL10.079	B
TC32603.33	RBL03.233	E	HW021610.05	RBL10.080	F
TC32603.34	RBL03.234	B	HW021610.06	RBL10.081	C
TC32603.35	RBL03.235	E	HW021610.07	RBL10.082	S
YC21103.01	RBL03.236	A	HW021610.08	RBL10.083	F
YC21103.02	RBL03.237	E	HW021610.09	RBL10.084	E
YC21103.03	RBL03.238	E	HW021610.10	RBL10.085	S
YC21103.04	RBL03.239	E	HW021610.11	RBL10.086	S

YC21103.05	RBL03.240	E	HW021610.12	RBL10.087	C
YC21103.06	RBL03.241	E	HW021610.13	RBL10.088	E
YC21103.07	RBL03.242	E	HW021610.14	RBL10.089	G
YC21103.08	RBL03.243	E	HW021610.15	RBL10.090	E
YC21103.09	RBL03.244	V	HW021610.16	RBL10.091	E
YC21103.10	RBL03.245	A	HW021610.17	RBL10.092	E
YC21103.11	RBL03.246	E	HW021610.18	RBL10.093	E
YC21103.12	RBL03.247	E	HW021610.19	RBL10.094	S
YC21103.13	RBL03.248	E	HW021610.20	RBL10.095	E
YC21103.14	RBL03.249	E	HW021610.21	RBL10.096	E
YC21103.15	RBL03.250	A	HW021610.22	RBL10.097	S
YC21103.16	RBL03.251	E	HW021610.23	RBL10.098	E
YC21103.17	RBL03.252	E	HW021610.24	RBL10.099	E
YC21103.18	RBL03.253	E	HW021610.25	RBL10.100	S
YC21103.19	RBL03.254	E	TC021610.01	RBL10.101	E
YC21103.20	RBL03.255	R	TC021610.02	RBL10.102	E
YC21103.21	RBL03.256	V	TC021610.03	RBL10.103	E
YC21103.22	RBL03.257	E	TC021610.04	RBL10.104	E
YC21103.23	RBL03.258	E	TC021610.05	RBL10.105	E
YC21103.24	RBL03.259	E	TC021610.06	RBL10.106	E
YC21103.25	RBL03.260	R	TC021610.07	RBL10.107	E
YC22803.01	RBL03.261	E	TC021610.08	RBL10.108	E
YC22803.02	RBL03.262	S	TC021610.09	RBL10.109	E
YC22803.03	RBL03.263	E	TC021610.10	RBL10.110	E
YC22803.04	RBL03.264	E	TC021610.11	RBL10.111	E
YC22803.05	RBL03.265	E	TC021610.12	RBL10.112	E
YC22803.06	RBL03.266	E	TC021610.13	RBL10.113	E
YC22803.07	RBL03.267	B	TC021610.14	RBL10.114	E
YC22803.08	RBL03.268	R	TC021610.15	RBL10.115	E
YC22803.09	RBL03.269	R	TC021610.16	RBL10.116	E
YC22803.10	RBL03.270	E	TC021610.17	RBL10.117	E
YC22803.11	RBL03.271	E	TC021610.18	RBL10.118	E
YC22803.12	RBL03.272	E	TC021610.19	RBL10.119	E
YC22803.13	RBL03.273	E	TC021610.20	RBL10.120	E
YC22803.14	RBL03.274	E	TC021610.21	RBL10.121	E
YC22803.15	RBL03.275	E	TC021610.22	RBL10.122	E
YC22803.16	RBL03.276	E	TC021610.23	RBL10.123	E
YC22803.17	RBL03.277	E	TC021610.24	RBL10.124	E
YC22803.18	RBL03.278	E	TC021610.25	RBL10.125	E
YC22803.19	RBL03.279	S	YC021810.01	RBL10.126	E
YC22803.20	RBL03.280	E	YC021810.02	RBL10.127	E

YC22803.21	RBL03.281	E	YC021810.03	RBL10.128	R
YC22803.22	RBL03.282	E	YC021810.04	RBL10.129	E
YC22803.23	RBL03.283	R	YC021810.05	RBL10.130	E
YC22803.24	RBL03.284	E	YC021810.06	RBL10.131	C
YC22803.25	RBL03.285	B	YC021810.07	RBL10.132	E
YC31203.01	RBL03.286	R	YC021810.08	RBL10.133	B
YC31203.02	RBL03.287	B	YC021810.09	RBL10.134	E
YC31203.03	RBL03.288	E	YC021810.10	RBL10.135	J
YC31203.04	RBL03.289	Q	YC021810.11	RBL10.136	C
YC31203.05	RBL03.290	E	YC021810.12	RBL10.137	E
YC31203.06	RBL03.291	E	YC021810.13	RBL10.138	E
YC31203.07	RBL03.292	E	YC021810.14	RBL10.139	E
YC31203.08	RBL03.293	S	YC021810.15	RBL10.140	B
YC31203.09	RBL03.294	S	YC021810.16	RBL10.141	E
YC31203.10	RBL03.295	E	YC021810.17	RBL10.142	S
YC31203.11	RBL03.296	R	YC021810.18	RBL10.143	E
YC31203.12	RBL03.297	C	YC021810.19	RBL10.144	E
YC31203.13	RBL03.298	E	YC021810.20	RBL10.145	E
YC31203.14	RBL03.299	E	YC021810.21	RBL10.146	E
YC31203.15	RBL03.300	A	YC021810.22	RBL10.147	E
YC31203.16	RBL03.301	R	YC021810.23	RBL10.148	E
YC31203.18	RBL03.303	E	YC021810.24	RBL10.149	E
YC31203.19	RBL03.304	R	YC021810.25	RBL10.150	S
YC31203.20	RBL03.305	S	YC030110.01	RBL10.151	E
YC31203.21	RBL03.306	R	YC030110.02	RBL10.152	H
YC31203.22	RBL03.307	E	YC030110.03	RBL10.153	E
YC31203.23	RBL03.308	B	YC030110.04	RBL10.154	E
YC31203.24	RBL03.309	E	YC030110.05	RBL10.155	E
YC31203.25	RBL03.310	R	YC030110.06	RBL10.156	A
YC31203.26	RBL03.311	Q	YC030110.07	RBL10.157	E
YC31203.27	RBL03.312	E	YC030110.08	RBL10.158	E
YC31203.28	RBL03.313	E	YC030110.09	RBL10.159	E
YC31203.29	RBL03.314	D	YC030110.10	RBL10.160	M
YC32503.01	RBL03.315	E	YC030110.11	RBL10.161	E
YC32503.02	RBL03.316	E	YC030110.12	RBL10.162	E
YC32503.03	RBL03.317	E	YC030110.13	RBL10.163	E
YC32503.04	RBL03.318	B	YC030110.14	RBL10.164	R
YC32503.05	RBL03.319	R	YC030110.15	RBL10.165	E
YC32503.06	RBL03.320	R	YC030110.16	RBL10.166	E
YC32503.07	RBL03.321	R	YC030110.17	RBL10.167	E
YC32503.08	RBL03.322	R	YC030110.18	RBL10.168	H

YC32503.09	RBL03.323	B	YC030110.19	RBL10.169	E
YC32503.10	RBL03.324	S	YC030110.20	RBL10.170	R
YC32503.11	RBL03.325	E	YC030110.21	RBL10.171	HH
YC32503.12	RBL03.326	E	YC030110.22	RBL10.172	B
YC32503.13	RBL03.327	B	YC030110.23	RBL10.173	E
YC32503.14	RBL03.328	E	YC030110.24	RBL10.174	R
YC32503.15	RBL03.329	R	YC030110.25	RBL10.175	S
YC32503.16	RBL03.330	B	TC030210.01	RBL10.176	E
YC32503.17	RBL03.331	R	TC030210.02	RBL10.177	Q
YC32503.18	RBL03.332	E	TC030210.03	RBL10.178	A
YC32503.19	RBL03.333	E	TC030210.04	RBL10.179	E
YC32503.20	RBL03.334	S	TC030210.05	RBL10.180	E
YC32503.21	RBL03.335	R	TC030210.06	RBL10.181	E
YC32503.22	RBL03.336	M	TC030210.07	RBL10.182	E
YC32503.23	RBL03.337	E	TC030210.08	RBL10.183	E
YC32503.24	RBL03.338	E	TC030210.09	RBL10.184	E
YC32503.25	RBL03.339	E	TC030210.10	RBL10.185	E
YC32503.26	RBL03.340	E	TC030210.11	RBL10.186	E
YC32503.27	RBL03.341	E	TC030210.12	RBL10.187	F
YC32503.28	RBL03.342	B	TC030210.13	RBL10.188	E
YC32503.29	RBL03.343	S	TC030210.14	RBL10.189	BB
YC32503.30	RBL03.344	E	TC030210.15	RBL10.190	E
YC32503.31	RBL03.345	B	TC030210.16	RBL10.191	S
YC32503.32	RBL03.346	C	TC030210.17	RBL10.192	E
YC32503.33	RBL03.347	E	TC030210.18	RBL10.193	E
YC32503.35	RBL03.349	B	TC030210.19	RBL10.194	E
TC31301.01	RBL03.350	E	TC030210.20	RBL10.195	E
TC31301.02	RBL03.351	E	TC030210.21	RBL10.196	E
TC31301.03	RBL03.352	E	TC030210.22	RBL10.197	E
TC31301.04	RBL03.353	E	TC030210.23	RBL10.198	E
TC31301.05	RBL03.354	E	TC030210.24	RBL10.199	S
TC31301.06	RBL03.355	S	TC030210.25	RBL10.200	E
TC31301.07	RBL03.356	C	HH030310.01	RBL10.201	E
TC31301.08	RBL03.357	R	HH030310.02	RBL10.202	E
TC31301.10	RBL03.359	S	HH030310.03	RBL10.203	B
TC31301.11	RBL03.360	E	HH030310.04	RBL10.204	R
TC31301.12	RBL03.361	S	HH030310.05	RBL10.205	R
TC31301.13	RBL03.362	R	HH030310.06	RBL10.206	A
TC31301.14	RBL03.363	E	HH030310.07	RBL10.207	E
TC31301.15	RBL03.364	S	HH030310.08	RBL10.208	E
TC31301.16	RBL03.365	E	HH030310.09	RBL10.209	E

TC31301.17	RBL03.366	E	HH030310.10	RBL10.210	B
TC31301.18	RBL03.367	S	HH030310.11	RBL10.211	E
TC31301.19	RBL03.368	S	HH030310.12	RBL10.212	E
TC31301.20	RBL03.369	E	HH030310.13	RBL10.213	A
TC31301.21	RBL03.370	C	HH030310.14	RBL10.214	A
TC31301.22	RBL03.371	E	HH030310.15	RBL10.215	E
TC31301.23	RBL03.372	E	HH030310.16	RBL10.216	E
TC31301.24	RBL03.373	E	HH030310.17	RBL10.217	E
TC31301.25	RBL03.374	C	HH030310.18	RBL10.218	E
HH020604.02	RBL04.002	F	HH030310.19	RBL10.219	H
HH020604.03	RBL04.003	B	HH030310.20	RBL10.220	E
HH020604.04	RBL04.004	E	HH030310.21	RBL10.221	E
HH020604.05	RBL04.005	E	HH030310.22	RBL10.222	E
HH020604.06	RBL04.006	C	HH030310.23	RBL10.223	E
HH020604.07	RBL04.007	DD	HH030310.24	RBL10.224	E
HH020604.08	RBL04.008	H	HH030310.25	RBL10.225	R
HH020604.09	RBL04.009	Q	LC031010.01	RBL10.226	E
HH020604.01	RBL04.01	E	LC031010.02	RBL10.227	V
HH020604.10	RBL04.010	E	LC031010.03	RBL10.228	E
HH020604.11	RBL04.011	E	LC031010.04	RBL10.229	E
HH020604.12	RBL04.012	E	LC031010.05	RBL10.230	E
HH020604.13	RBL04.013	E	LC031010.06	RBL10.231	E
HH020604.14	RBL04.014	E	LC031010.07	RBL10.232	E
HH020604.15	RBL04.015	K	LC031010.08	RBL10.233	E
HH031504.01	RBL04.016	E	LC031010.09	RBL10.234	E
HH031504.02	RBL04.017	E	LC031010.10	RBL10.235	B
HH031504.03	RBL04.018	B	LC031010.11	RBL10.236	E
HH031504.04	RBL04.019	E	LC031010.12	RBL10.237	P
HH031504.05	RBL04.020	E	LC031010.13	RBL10.238	E
HH031504.06	RBL04.021	R	LC031010.14	RBL10.239	E
HH031504.07	RBL04.022	E	LC031010.15	RBL10.240	E
HH031504.08	RBL04.023	E	LC031010.16	RBL10.241	E
HH031504.09	RBL04.024	E	LC031010.17	RBL10.242	E
HH031504.10	RBL04.025	E	LC031010.18	RBL10.243	E
HH031504.11	RBL04.026	E	LC031010.19	RBL10.244	E
HH031504.12	RBL04.027	E	LC031010.20	RBL10.245	E
HH031504.13	RBL04.028	A	LC031010.21	RBL10.246	E
HH031504.14	RBL04.029	E	LC031010.22	RBL10.247	E
HH031504.15	RBL04.030	Z	LC031010.23	RBL10.248	V
HH031504.16	RBL04.031	E	LC031010.24	RBL10.249	E
HH031504.17	RBL04.032	E	LC031010.25	RBL10.250	P

HH031504.18	RBL04.033	S	VC031610.01	RBL10.251	E
HH031504.19	RBL04.034	S	VC031610.02	RBL10.252	B
HH031504.20	RBL04.035	E	VC031610.03	RBL10.253	E
HH031504.21	RBL04.036	F	VC031610.04	RBL10.254	E
HH031504.22	RBL04.037	E	VC031610.05	RBL10.255	C
HH031504.23	RBL04.038	E	VC031610.06	RBL10.256	E
HH032904.01	RBL04.039	E	VC031610.07	RBL10.257	E
HH032904.02	RBL04.040	S	VC031610.08	RBL10.258	R
HH032904.03	RBL04.041	V	VC031610.09	RBL10.259	R
HH032904.04	RBL04.042	V	VC031610.10	RBL10.260	B
HH032904.05	RBL04.043	V	VC031610.11	RBL10.261	B
HH032904.06	RBL04.044	V	VC031610.12	RBL10.262	E
HH032904.07	RBL04.045	E	VC031610.13	RBL10.263	B
HH032904.08	RBL04.046	E	VC031610.14	RBL10.264	E
HH032904.09	RBL04.047	B	VC031610.15	RBL10.265	E
HH032904.10	RBL04.048	E	VC031610.16	RBL10.266	E
HH032904.11	RBL04.049	E	VC031610.17	RBL10.267	E
HH032904.12	RBL04.050	B	VC031610.18	RBL10.268	E
HH032904.13	RBL04.051	V	VC031610.19	RBL10.269	E
HH032904.14	RBL04.052	E	VC031610.20	RBL10.270	E
HH032904.15	RBL04.053	V	VC031610.21	RBL10.271	E
HH032904.16	RBL04.054	E	VC031610.22	RBL10.272	E
HH032904.17	RBL04.055	H	VC031610.23	RBL10.273	E
HH032904.18	RBL04.056	B	VC031610.24	RBL10.274	B
HH032904.19	RBL04.057	E	VC031610.25	RBL10.275	R
HH032904.20	RBL04.058	V	TC031610.01	RBL10.276	E
HH032904.21	RBL04.059	F	TC031610.02	RBL10.277	E
HH032904.22	RBL04.060	V	TC031610.03	RBL10.278	E
HH032904.23	RBL04.061	E	TC031610.04	RBL10.279	E
HH032904.24	RBL04.062	V	TC031610.05	RBL10.280	E
HH032904.25	RBL04.063	E	TC031610.06	RBL10.281	E
NM020304.01	RBL04.064	E	TC031610.07	RBL10.282	B
NM020304.02	RBL04.065	E	TC031610.08	RBL10.283	E
NM020304.03	RBL04.066	E	TC031610.09	RBL10.284	E
NM020304.04	RBL04.067	E	TC031610.10	RBL10.285	E
NM020304.05	RBL04.068	B	TC031610.11	RBL10.286	R
NM020304.06	RBL04.069	E	TC031610.12	RBL10.287	E
NM020304.07	RBL04.070	E	TC031610.13	RBL10.288	E
NM020304.08	RBL04.071	E	TC031610.15	RBL10.290	E
NM020304.09	RBL04.072	E	TC031610.16	RBL10.291	E
NM020304.10	RBL04.073	E	TC031610.17	RBL10.292	E

NM021704.01	RBL04.074	E	TC031610.18	RBL10.293	S
NM021704.02	RBL04.075	E	TC031610.19	RBL10.294	R
NM021704.03	RBL04.076	E	TC031610.20	RBL10.295	S
NM021704.04	RBL04.077	E	TC031610.21	RBL10.296	E
NM022004.01	RBL04.078	E	TC031610.22	RBL10.297	R
NM022004.02	RBL04.079	E	TC031610.24	RBL10.299	E
NM022004.03	RBL04.080	S	TC031610.25	RBL10.300	R
NM022004.04	RBL04.081	E	YC031610.01	RBL10.301	R
NM022004.05	RBL04.082	E	YC031610.02	RBL10.302	E
NM022004.06	RBL04.083	V	YC031610.03	RBL10.303	E
NM022004.07	RBL04.084	E	YC031610.04	RBL10.304	E
NM022004.08	RBL04.085	A	YC031610.05	RBL10.305	R
NM022004.09	RBL04.086	A	YC031610.06	RBL10.306	E
NM022004.10	RBL04.087	E	YC031610.07	RBL10.307	E
NM022004.11	RBL04.088	E	YC031610.08	RBL10.308	E
NM022004.12	RBL04.089	A	YC031610.09	RBL10.309	E
NM022004.13	RBL04.090	E	YC031610.10	RBL10.310	E
NM022004.14	RBL04.091	E	YC031610.11	RBL10.311	E
NM022004.15	RBL04.092	R	YC031610.12	RBL10.312	E
NM022004.16	RBL04.093	E	YC031610.13	RBL10.313	E
NM022004.17	RBL04.094	CC	YC031610.14	RBL10.314	A
NM022004.18	RBL04.095	E	YC031610.15	RBL10.315	E
NM022004.19	RBL04.096	E	YC031610.16	RBL10.316	E
NM022004.20	RBL04.097	P	YC031610.17	RBL10.317	E
NM022004.21	RBL04.098	R	YC031610.18	RBL10.318	F
NM022004.22	RBL04.099	S	YC031610.19	RBL10.319	R
NM022004.23	RBL04.100	E	YC031610.20	RBL10.320	B
NM022004.24	RBL04.101	S	YC031610.21	RBL10.321	E
NM022004.25	RBL04.102	E	YC031610.22	RBL10.322	J
NM030104.01	RBL04.103	E	YC031610.23	RBL10.323	E
NM030104.02	RBL04.104	A	YC031610.24	RBL10.324	E
NM030104.03	RBL04.105	G	YC031610.25	RBL10.325	G
NM030104.04	RBL04.106	G	JC032910.01	RBL10.326	E
NM030104.05	RBL04.107	E	JC032910.02	RBL10.327	E
NM030104.06	RBL04.108	A	JC032910.03	RBL10.328	S
NM030104.07	RBL04.109	E	JC032910.04	RBL10.329	C
NM030104.08	RBL04.110	E	JC032910.05	RBL10.330	E
NM030104.09	RBL04.111	E	JC032910.06	RBL10.331	R
NM030104.10	RBL04.112	E	JC032910.07	RBL10.332	E
NM030104.11	RBL04.113	E	JC032910.08	RBL10.333	E
NM030104.12	RBL04.114	E	JC032910.09	RBL10.334	E

NM030104.13	RBL04.115	E	JC032910.10	RBL10.335	E
NM030104.14	RBL04.116	E	JC032910.11	RBL10.336	E
NM030104.15	RBL04.117	E	JC032910.12	RBL10.337	E
NM030104.16	RBL04.118	S	JC032910.13	RBL10.338	C
NM030104.17	RBL04.119	F	JC032910.14	RBL10.339	E
NM030104.18	RBL04.120	E	JC032910.15	RBL10.340	BB
NM030104.19	RBL04.121	E	JC032910.16	RBL10.341	E
NM030104.20	RBL04.122	E	JC032910.17	RBL10.342	E
NM030104.21	RBL04.123	E	JC032910.18	RBL10.343	C
NM030104.22	RBL04.124	E	JC032910.19	RBL10.344	E
NM030104.23	RBL04.125	E	JC032910.20	RBL10.345	S
NM030104.24	RBL04.126	E	JC032910.21	RBL10.346	E
NM030104.25	RBL04.127	A	JC032910.22	RBL10.347	E
NM041304.01	RBL04.128	F	JC032910.23	RBL10.348	E
NM041304.02	RBL04.129	E	JC032910.24	RBL10.349	R
NM041304.03	RBL04.130	E	JC032910.25	RBL10.350	H
NM041304.04	RBL04.131	C	YC032910.01	RBL10.351	B
NM041304.05	RBL04.132	S	YC032910.02	RBL10.352	E
NM041304.06	RBL04.133	E	YC032910.03	RBL10.353	E
NM041304.07	RBL04.134	E	YC032910.04	RBL10.354	Q
NM041304.08	RBL04.135	V	YC032910.05	RBL10.355	E
NM041304.09	RBL04.136	F	YC032910.06	RBL10.356	Q
NM041304.10	RBL04.137	E	YC032910.07	RBL10.357	E
NM041304.11	RBL04.138	S	YC032910.08	RBL10.358	C
NM041304.12	RBL04.139	E	YC032910.09	RBL10.359	E
NM041304.13	RBL04.140	E	YC032910.10	RBL10.360	E
NM041304.14	RBL04.141	S	YC032910.11	RBL10.361	E
NM041304.15	RBL04.142	A	YC032910.12	RBL10.362	E
NM041304.16	RBL04.143	R	YC032910.13	RBL10.363	E
NM041304.17	RBL04.144	E	YC032910.14	RBL10.364	R
NM041304.18	RBL04.145	E	YC032910.15	RBL10.365	Q
NM041304.19	RBL04.146	E	YC032910.16	RBL10.366	C
NM041304.20	RBL04.147	G	YC032910.17	RBL10.367	G
NM041304.21	RBL04.148	E	YC032910.18	RBL10.368	E
TC020304.01	RBL04.149	E	YC032910.19	RBL10.369	HH
TC020304.02	RBL04.150	A	YC032910.20	RBL10.370	C
TC020304.03	RBL04.151	E	YC032910.21	RBL10.371	C
TC020304.04	RBL04.152	E	YC032910.22	RBL10.372	E
TC020304.05	RBL04.153	E	YC032910.23	RBL10.373	E
TC020304.06	RBL04.154	E	YC032910.24	RBL10.374	E
TC020304.07	RBL04.155	E	YC032910.25	RBL10.375	R

TC020304.08	RBL04.156	E	YC032910.26	RBL10.376	E
TC020304.09	RBL04.157	E	YC032910.27	RBL10.377	E
TC020304.10	RBL04.158	E	TC033010.01	RBL10.378	BB
TC020304.11	RBL04.159	E	TC033010.02	RBL10.379	R
TC020304.12	RBL04.160	A	TC033010.03	RBL10.380	B
TC020304.13	RBL04.161	A	TC033010.04	RBL10.381	E
TC020304.14	RBL04.162	E	TC033010.05	RBL10.382	R
TC020304.15	RBL04.163	E	TC033010.06	RBL10.383	E
TC020304.16	RBL04.164	I	TC033010.07	RBL10.384	R
TC020304.17	RBL04.165	E	TC033010.08	RBL10.385	E
TC020304.18	RBL04.166	E	TC033010.09	RBL10.386	R
TC020304.19	RBL04.167	E	TC033010.10	RBL10.387	E
TC020304.20	RBL04.168	E	TC033010.11	RBL10.388	E
TC020304.21	RBL04.169	E	TC033010.12	RBL10.389	A
TC020304.22	RBL04.170	E	TC033010.13	RBL10.390	E
TC020304.23	RBL04.171	E	TC033010.14	RBL10.391	S
TC020304.24	RBL04.172	E	TC033010.15	RBL10.392	E
TC020304.25	RBL04.173	E	TC033010.16	RBL10.393	H
TC020304.26	RBL04.174	E	TC033010.17	RBL10.394	R
TC020304.27	RBL04.175	E	TC033010.18	RBL10.395	R
TC021804.01	RBL04.176	S	TC033010.19	RBL10.396	E
TC021804.02	RBL04.177	A	TC033010.20	RBL10.397	R
TC021804.03	RBL04.178	A	TC033010.21	RBL10.398	A
TC021804.04	RBL04.179	E	TC033010.22	RBL10.399	E
TC021804.05	RBL04.180	E	TC033010.23	RBL10.400	E
TC021804.06	RBL04.181	E	TC033010.24	RBL10.401	E
TC021804.07	RBL04.182	E	TC033010.25	RBL10.402	E
TC021804.08	RBL04.183	E	HF033010.01	RBL10.403	G
TC021804.09	RBL04.184	S	HF033010.02	RBL10.404	R
TC021804.10	RBL04.185	S	HF033010.03	RBL10.405	E
TC021804.11	RBL04.186	E	HF033010.04	RBL10.406	E
TC021804.12	RBL04.187	E	HF033010.05	RBL10.407	B
TC021804.13	RBL04.188	S	HF033010.06	RBL10.408	E
TC021804.14	RBL04.189	E	HF033010.08	RBL10.410	A
TC021804.15	RBL04.190	S	HF033010.09	RBL10.411	A
TC021804.16	RBL04.191	S	HF033010.10	RBL10.412	E
TC021804.17	RBL04.192	A	HF033010.11	RBL10.413	E
TC021804.18	RBL04.193	A	HF033010.12	RBL10.414	H
TC021804.19	RBL04.194	E	HF033010.13	RBL10.415	E
TC021804.20	RBL04.195	A	HF033010.14	RBL10.416	E
TC021804.21	RBL04.196	B	HF033010.15	RBL10.417	E

TC021804.22	RBL04.197	E
TC021804.23	RBL04.198	S
TC021804.24	RBL04.199	E
TC021804.25	RBL04.200	E
TC030204.01	RBL04.201	E
TC030204.02	RBL04.202	E
TC030204.03	RBL04.203	B
TC030204.04	RBL04.204	E
TC030204.05	RBL04.205	R
TC030204.06	RBL04.206	E
TC030204.07	RBL04.207	E
TC030204.08	RBL04.208	E
TC030204.09	RBL04.209	E
TC030204.10	RBL04.210	E
TC030204.11	RBL04.211	B
TC030204.12	RBL04.212	E
TC030204.13	RBL04.213	B
TC030204.14	RBL04.214	B
TC030204.15	RBL04.215	E
TC030204.16	RBL04.216	E
TC030204.17	RBL04.217	E
TC030204.18	RBL04.218	E
TC030204.19	RBL04.219	E
TC030204.20	RBL04.220	E
TC030204.21	RBL04.221	E
TC030204.22	RBL04.222	R
TC030204.23	RBL04.223	E
TC030204.24	RBL04.224	E
TC030204.25	RBL04.225	B
TC031704.01	RBL04.226	E
TC031704.02	RBL04.227	E
TC031704.03	RBL04.228	E
TC031704.04	RBL04.229	E
TC031704.05	RBL04.230	E
TC031704.06	RBL04.231	E
TC031704.07	RBL04.232	E
TC031704.08	RBL04.233	E
TC031704.09	RBL04.234	E
TC031704.11	RBL04.236	E
TC031704.12	RBL04.237	E
TC031704.13	RBL04.238	E

HF033010.16	RBL10.418	S
HF033010.17	RBL10.419	S
HF033010.18	RBL10.420	R
HF033010.19	RBL10.421	E
HF033010.20	RBL10.422	E
HF033010.21	RBL10.423	E
HF033010.22	RBL10.424	E
HF033010.23	RBL10.425	E
HF033010.24	RBL10.426	E
HF033010.25	RBL10.427	E
TC041310.01	RBL10.428	F
TC041310.02	RBL10.429	E
TC041310.03	RBL10.430	E
TC041310.04	RBL10.431	E
TC041310.05	RBL10.432	S
TC041310.06	RBL10.433	E
TC041310.07	RBL10.434	GG
TC041310.08	RBL10.435	S
TC041310.09	RBL10.436	B
TC041310.10	RBL10.437	E
TC041310.11	RBL10.438	E
TC041310.12	RBL10.439	E
TC041310.13	RBL10.440	Q
TC041310.14	RBL10.441	E
TC041310.15	RBL10.442	C
TC041310.16	RBL10.443	E
TC041310.17	RBL10.444	E
TC041310.18	RBL10.445	BB
TC041310.19	RBL10.446	E
TC041310.20	RBL10.447	E
TC041310.21	RBL10.448	E
TC041310.22	RBL10.449	BB
TC041310.23	RBL10.450	CC
TC041310.24	RBL10.451	E
TC041310.25	RBL10.452	GG
JC041510.01	RBL10.453	A
JC041510.02	RBL10.454	E
JC041510.03	RBL10.455	E
JC041510.04	RBL10.456	E
JC041510.05	RBL10.457	E
JC041510.06	RBL10.458	S

TC031704.14	RBL04.239	E	JC041510.07	RBL10.459	S
TC031704.15	RBL04.240	E	JC041510.08	RBL10.460	E
TC031704.16	RBL04.241	S	JC041510.09	RBL10.461	S
TC031704.17	RBL04.242	E	JC041510.10	RBL10.462	E
TC031704.18	RBL04.243	E	JC041510.11	RBL10.463	E
TC031704.19	RBL04.244	E	JC041510.12	RBL10.464	J
TC031704.20	RBL04.245	E	JC041510.13	RBL10.465	J
TC031704.21	RBL04.246	E	JC041510.14	RBL10.466	E
TC031704.22	RBL04.247	E	JC041510.15	RBL10.467	E
TC031704.23	RBL04.248	E	JC041510.16	RBL10.468	B
TC031704.24	RBL04.249	E	JC041510.17	RBL10.469	J
TC031704.25	RBL04.250	E	JC041510.18	RBL10.470	B
TC031704.26	RBL04.251	E	JC041510.19	RBL10.471	E
TC031704.27	RBL04.252	E	JC041510.20	RBL10.472	S
TC031704.28	RBL04.253	R	JC041510.21	RBL10.473	S
TC031704.29	RBL04.254	E	JC041510.22	RBL10.474	E
TC031704.30	RBL04.255	E	JC041510.23	RBL10.475	E
TC033004.01	RBL04.256	R	JC041510.24	RBL10.476	E
TC033004.02	RBL04.257	E	JC041510.25	RBL10.477	E
TC033004.03	RBL04.258	E	JC041510.26	RBL10.478	F
TC033004.04	RBL04.259	C	JC041510.27	RBL10.479	E
TC033004.05	RBL04.260	E	JC041510.28	RBL10.480	S
TC033004.06	RBL04.261	E	JC041510.29	RBL10.481	E
TC033004.07	RBL04.262	R	HH.013111.01	RBL11.001	H
TC033004.08	RBL04.263	C	HH.013111.02	RBL11.002	E
TC033004.09	RBL04.264	R	HH.013111.03	RBL11.003	E
TC033004.10	RBL04.265	R	HH.013111.04	RBL11.004	S
TC033004.11	RBL04.266	E	HH.013111.05	RBL11.005	E
TC033004.12	RBL04.267	E	HH.013111.06	RBL11.006	E
TC033004.13	RBL04.268	R	HH.013111.07	RBL11.007	E
TC033004.14	RBL04.269	R	HH.013111.08	RBL11.008	I
TC033004.15	RBL04.270	S	HH.013111.09	RBL11.009	E
TC033004.16	RBL04.271	C	HH.013111.10	RBL11.010	E
TC033004.17	RBL04.272	CC	HH.013111.11	RBL11.011	S
TC033004.18	RBL04.273	R	HH.013111.12	RBL11.012	E
TC033004.19	RBL04.274	R	HH.013111.13	RBL11.013	E
TC033004.20	RBL04.275	E	HH.013111.14	RBL11.014	B
TC033004.21	RBL04.276	P	HH.013111.15	RBL11.015	B
TC033004.22	RBL04.277	E	HH.013111.16	RBL11.016	R
TC033004.23	RBL04.278	R	HH.013111.17	RBL11.017	S
TC033004.24	RBL04.279	S	HH.013111.18	RBL11.018	E

TC033004.25	RBL04.280	E	HH.013111.19	RBL11.019	E
TC041304.01	RBL04.281	E	HH.013111.20	RBL11.020	B
TC041304.02	RBL04.282	E	HH.013111.21	RBL11.021	S
TC041304.03	RBL04.283	E	HH.013111.22	RBL11.022	E
TC041304.04	RBL04.284	E	HH.013111.23	RBL11.023	S
TC041304.05	RBL04.285	E	HH.013111.24	RBL11.024	S
TC041304.06	RBL04.286	S	HH.013111.25	RBL11.025	E
TC041304.07	RBL04.287	B	GC.020311.01	RBL11.026	E
TC041304.08	RBL04.288	A	GC.020311.02	RBL11.027	E
TC041304.09	RBL04.289	E	GC.020311.03	RBL11.028	B
TC041304.10	RBL04.290	E	GC.020311.04	RBL11.029	E
TC041304.11	RBL04.291	E	GC.020311.05	RBL11.030	E
TC041304.12	RBL04.292	S	GC.020311.06	RBL11.031	E
TC041304.13	RBL04.293	E	GC.020311.07	RBL11.032	E
TC041304.14	RBL04.294	E	GC.020311.08	RBL11.033	E
TC041304.15	RBL04.295	E	GC.020311.09	RBL11.034	E
TC041304.16	RBL04.296	E	GC.020311.10	RBL11.035	E
TC041304.17	RBL04.297	E	GC.020311.11	RBL11.036	E
TC041304.18	RBL04.298	E	GC.020311.12	RBL11.037	E
TC041304.19	RBL04.299	S	GC.020311.13	RBL11.038	E
TC041304.20	RBL04.300	E	GC.020311.14	RBL11.039	E
TC041304.21	RBL04.301	S	GC.020311.15	RBL11.040	E
TC041304.22	RBL04.302	E	GC.020311.16	RBL11.041	E
TC041304.23	RBL04.303	B	GC.020311.17	RBL11.042	E
TC041304.24	RBL04.304	E	GC.020311.18	RBL11.043	E
TC041304.25	RBL04.305	E	GC.020311.19	RBL11.044	E
TC041304.26	RBL04.306	E	GC.020311.20	RBL11.045	V
TC041304.27	RBL04.307	E	GC.020311.21	RBL11.046	S
WC032904.01	RBL04.308	E	GC.020311.22	RBL11.047	R
WC032904.02	RBL04.309	E	GC.020311.23	RBL11.048	S
WC032904.03	RBL04.310	E	TC.020311.01	RBL11.049	E
WC032904.04	RBL04.311	E	TC.020311.02	RBL11.050	B
WC032904.05	RBL04.312	E	TC.020311.03	RBL11.051	E
WC032904.06	RBL04.313	C	TC.020311.04	RBL11.052	E
WC032904.07	RBL04.314	E	TC.020311.05	RBL11.053	E
WC032904.08	RBL04.315	E	TC.020311.06	RBL11.054	B
WC032904.09	RBL04.316	E	TC.020311.07	RBL11.055	B
WC032904.10	RBL04.317	E	TC.020311.08	RBL11.056	E
WC032904.11	RBL04.318	E	TC.020311.09	RBL11.057	E
WC032904.12	RBL04.319	E	TC.020311.10	RBL11.058	E
WC032904.13	RBL04.320	E	TC.020311.11	RBL11.059	E

WC032904.14	RBL04.321	E	TC.020311.12	RBL11.060	E
WC032904.15	RBL04.322	U	TC.020311.13	RBL11.061	B
WC032904.16	RBL04.323	F	TC.020311.14	RBL11.062	E
WC032904.17	RBL04.324	E	TC.020311.15	RBL11.063	B
WC032904.18	RBL04.325	R	TC.020311.16	RBL11.064	E
WC032904.19	RBL04.326	E	TC.020311.17	RBL11.065	A
WC032904.20	RBL04.327	B	TC.020311.18	RBL11.066	E
WC032904.21	RBL04.328	E	TC.020311.19	RBL11.067	E
WC032904.22	RBL04.329	A	TC.020311.20	RBL11.068	E
WC032904.23	RBL04.330	E	TC.020311.21	RBL11.069	A
WC032904.24	RBL04.331	E	TC.020311.22	RBL11.070	B
WC032904.25	RBL04.332	E	TC.020311.23	RBL11.071	B
WC041204.01	RBL04.333	R	TC.020311.24	RBL11.072	E
WC041204.02	RBL04.334	C	TC.020311.25	RBL11.073	A
WC041204.03	RBL04.335	R	TC.021511.01	RBL11.074	E
WC041204.04	RBL04.336	E	TC.021511.02	RBL11.075	E
WC041204.05	RBL04.337	R	TC.021511.03	RBL11.076	E
WC041204.06	RBL04.338	R	TC.021511.04	RBL11.077	S
WC041204.07	RBL04.339	R	TC.021511.05	RBL11.078	R
WC041204.08	RBL04.340	R	TC.021511.06	RBL11.079	E
WC041204.09	RBL04.341	R	TC.021511.07	RBL11.080	E
WC041204.10	RBL04.342	R	TC.021511.08	RBL11.081	E
WC041204.11	RBL04.343	R	TC.021511.09	RBL11.082	R
WC041204.12	RBL04.344	R	TC.021511.10	RBL11.083	E
WC041204.13	RBL04.345	R	TC.021511.11	RBL11.084	E
WC041204.14	RBL04.346	R	TC.021511.12	RBL11.085	E
WC041204.15	RBL04.347	C	TC.021511.13	RBL11.086	E
WC041204.16	RBL04.348	C	TC.021511.14	RBL11.087	Q
WC041204.17	RBL04.349	E	TC.021511.15	RBL11.088	E
WC041204.18	RBL04.350	R	TC.021511.16	RBL11.089	E
WC041204.19	RBL04.351	R	TC.021511.17	RBL11.090	E
WC041204.20	RBL04.352	E	TC.021511.18	RBL11.091	A
WC041204.21	RBL04.353	R	TC.021511.19	RBL11.092	E
WC041204.22	RBL04.354	C	TC.021511.20	RBL11.093	E
WC041204.23	RBL04.355	E	TC.021511.21	RBL11.094	E
WC041204.24	RBL04.356	C	TC.021511.22	RBL11.095	E
WC041204.25	RBL04.357	R	TC.021511.23	RBL11.096	A
WC050404.01	RBL04.358	A	TC.021511.24	RBL11.097	E
WC050404.02	RBL04.359	E	YC.021511.01	RBL11.098	E
WC050404.03	RBL04.360	B	YC.021511.02	RBL11.099	E
WC050404.04	RBL04.361	E	YC.021511.03	RBL11.100	F

WC050404.05	RBL04.362	E	YC.021511.04	RBL11.101	E
WC050404.06	RBL04.363	E	YC.021511.05	RBL11.102	K
WC050404.08	RBL04.365	E	YC.021511.06	RBL11.103	E
WC050404.09	RBL04.366	R	YC.021511.07	RBL11.104	E
WC050404.10	RBL04.367	E	YC.021511.08	RBL11.105	R
WC050404.11	RBL04.368	R	YC.021511.09	RBL11.106	E
WC050404.12	RBL04.369	B	YC.021511.10	RBL11.107	M
WC050404.13	RBL04.370	E	YC.021511.11	RBL11.108	E
WC050404.14	RBL04.371	A	YC.021511.12	RBL11.109	E
WC050404.15	RBL04.372	F	YC.021511.13	RBL11.110	E
WC050404.16	RBL04.373	E	YC.021511.14	RBL11.111	E
WC050404.17	RBL04.374	R	YC.021511.15	RBL11.112	E
WC050404.18	RBL04.375	E	YC.021511.16	RBL11.113	M
WC050404.19	RBL04.376	E	YC.021511.17	RBL11.114	E
WC050404.20	RBL04.377	E	YC.021511.18	RBL11.115	E
WC050404.21	RBL04.378	E	YC.021511.19	RBL11.116	B
WC050404.22	RBL04.379	E	YC.021511.20	RBL11.117	E
WC050404.23	RBL04.380	E	YC.021511.21	RBL11.118	C
WC050404.24	RBL04.381	A	YC.021511.22	RBL11.119	E
WC050404.25	RBL04.382	E	YC.021511.23	RBL11.120	M
YC012904.01	RBL04.383	E	YC.021511.24	RBL11.121	E
YC012904.02	RBL04.384	E	NM.021711.01	RBL11.122	G
YC012904.03	RBL04.385	R	NM.021711.02	RBL11.123	E
YC012904.04	RBL04.386	E	NM.021711.03	RBL11.124	S
YC012904.05	RBL04.387	B	NM.021711.04	RBL11.125	E
YC012904.06	RBL04.388	B	NM.021711.05	RBL11.126	F
YC012904.07	RBL04.389	E	NM.021711.06	RBL11.127	S
YC012904.08	RBL04.390	E	NM.022411.01	RBL11.128	E
YC012904.09	RBL04.391	E	NM.022411.02	RBL11.129	E
YC012904.10	RBL04.392	E	NM.022411.03	RBL11.130	C
YC012904.11	RBL04.393	E	NM.022411.04	RBL11.131	E
YC012904.12	RBL04.394	E	NM.022411.05	RBL11.132	E
YC012904.13	RBL04.395	S	NM.022411.06	RBL11.133	E
YC012904.14	RBL04.396	S	NM.022411.07	RBL11.134	E
YC012904.15	RBL04.397	R	NM.022411.08	RBL11.135	K
YC012904.16	RBL04.398	E	NM.022411.09	RBL11.136	E
YC012904.17	RBL04.399	E	NM.022411.10	RBL11.137	E
YC012904.18	RBL04.400	E	NM.022411.11	RBL11.138	E
YC012904.19	RBL04.401	R	NM.022411.12	RBL11.139	A
YC012904.20	RBL04.402	H	NM.022411.13	RBL11.140	
YC012904.21	RBL04.403	E	NM.022411.14	RBL11.141	E

YC012904.22	RBL04.404	S	NM.022411.15	RBL11.142	E
YC012904.23	RBL04.405	F	NM.022411.16	RBL11.143	E
YC012904.24	RBL04.406	E	NM.022411.17	RBL11.144	K
YC012904.25	RBL04.407	E	NM.022411.18	RBL11.145	S
YC012904.26	RBL04.408	E	NM.022411.19	RBL11.146	A
YC012904.27	RBL04.409	E	NM.022411.20	RBL11.147	E
YC012904.28	RBL04.410	B	NM.022411.21	RBL11.148	E
YC021704.01	RBL04.411	A	NM.022411.22	RBL11.149	E
YC021704.02	RBL04.412	E	NM.022411.23	RBL11.150	A
YC021704.03	RBL04.413	E	NM.022411.24	RBL11.151	E
YC021704.04	RBL04.414	E	NM.022411.25	RBL11.152	F
YC021704.05	RBL04.415	C	YC.022811.01	RBL11.153	E
YC021704.06	RBL04.416	E	YC.022811.02	RBL11.154	E
YC021704.07	RBL04.417	E	YC.022811.03	RBL11.155	B
YC021704.08	RBL04.418	R	YC.022811.04	RBL11.156	B
YC021704.09	RBL04.419	E	YC.022811.05	RBL11.157	A
YC021704.10	RBL04.420	E	YC.022811.06	RBL11.158	E
YC021704.11	RBL04.421	E	YC.022811.07	RBL11.159	R
YC021704.12	RBL04.422	E	YC.022811.08	RBL11.160	A
YC021704.13	RBL04.423	A	YC.022811.09	RBL11.161	E
YC021704.14	RBL04.424	K	YC.022811.10	RBL11.162	E
YC021704.15	RBL04.425	E	YC.022811.11	RBL11.163	E
YC021704.16	RBL04.426	E	YC.022811.12	RBL11.164	F
YC021704.17	RBL04.427	E	YC.022811.13	RBL11.165	B
YC021704.18	RBL04.428	E	YC.022811.14	RBL11.166	A
YC021704.19	RBL04.429	E	YC.022811.15	RBL11.167	E
YC021704.20	RBL04.430	E	YC.022811.16	RBL11.168	E
YC021704.21	RBL04.431	E	YC.022811.17	RBL11.169	B
YC021704.22	RBL04.432	E	YC.022811.18	RBL11.170	B
YC021704.23	RBL04.433	E	YC.022811.19	RBL11.171	A
YC021704.24	RBL04.434	C	YC.022811.20	RBL11.172	E
YC021704.25	RBL04.435	E	YC.022811.21	RBL11.173	C
YC030104.01	RBL04.436	E	YC.022811.22	RBL11.174	B
YC030104.02	RBL04.437	E	YC.022811.23	RBL11.175	U
YC030104.03	RBL04.438	R	YC.022811.24	RBL11.176	E
YC030104.04	RBL04.439	E	YC.022811.25	RBL11.177	V
YC030104.05	RBL04.440	J	YC.022811.26	RBL11.178	E
YC030104.06	RBL04.441	E	TC.030111.01	RBL11.179	E
YC030104.07	RBL04.442	E	TC.030111.02	RBL11.180	E
YC030104.08	RBL04.443	B	TC.030111.03	RBL11.181	S
YC030104.09	RBL04.444	B	TC.030111.04	RBL11.182	E

YC030104.10	RBL04.445	B	TC.030111.05	RBL11.183	E
YC030104.11	RBL04.446	S	TC.030111.06	RBL11.184	E
YC030104.12	RBL04.447	E	TC.030111.07	RBL11.185	E
YC030104.13	RBL04.448	E	TC.030111.08	RBL11.186	E
YC030104.14	RBL04.449	A	TC.030111.09	RBL11.187	J
YC030104.15	RBL04.450	S	TC.030111.10	RBL11.188	R
YC030104.16	RBL04.451	R	TC.030111.11	RBL11.189	E
YC030104.17	RBL04.452	E	TC.030111.12	RBL11.190	E
YC030104.18	RBL04.453	R	TC.030111.13	RBL11.191	U
YC030104.19	RBL04.454	E	TC.030111.14	RBL11.192	E
YC030104.20	RBL04.455	E	TC.030111.15	RBL11.193	E
YC030104.21	RBL04.456	B	TC.030111.16	RBL11.194	E
YC030104.22	RBL04.457	E	TC.030111.17	RBL11.195	E
YC030104.23	RBL04.458	S	TC.030111.18	RBL11.196	E
YC031504.01	RBL04.459	J	TC.030111.19	RBL11.197	E
YC031504.02	RBL04.460	E	TC.030111.20	RBL11.198	B
YC031504.03	RBL04.461	E	TC.030111.21	RBL11.199	E
YC031504.04	RBL04.462	E	TC.030111.22	RBL11.200	E
YC031504.05	RBL04.463	E	TC.030111.23	RBL11.201	E
YC031504.06	RBL04.464	E	TC.030111.24	RBL11.202	A
YC031504.07	RBL04.465	F	NM.030111.01	RBL11.206	E
YC031504.08	RBL04.466	E	NM.030111.02	RBL11.207	E
YC031504.09	RBL04.467	R	NM.030111.03	RBL11.208	R
YC031504.10	RBL04.468	B	NM.030111.04	RBL11.209	E
YC031504.11	RBL04.469	E	NM.030111.05	RBL11.210	E
YC031504.12	RBL04.470	E	NM.030111.06	RBL11.211	S
YC031504.13	RBL04.471	E	NM.030111.07	RBL11.212	E
YC031504.14	RBL04.472	S	NM.030111.08	RBL11.213	E
YC031504.15	RBL04.473	B	NM.030111.09	RBL11.214	S
YC031504.16	RBL04.474	E	NM.030111.10	RBL11.215	CC
YC031504.17	RBL04.475	E	NM.030111.11	RBL11.216	E
YC031504.18	RBL04.476	E	NM.030111.12	RBL11.217	E
YC031504.19	RBL04.477	E	NM.030111.13	RBL11.218	E
YC031504.20	RBL04.478	E	NM.030111.14	RBL11.219	E
YC031504.21	RBL04.479	E	NM.030111.15	RBL11.220	E
YC031504.22	RBL04.480	F	NM.030111.16	RBL11.221	E
YC031504.23	RBL04.481	E	NM.030111.17	RBL11.222	E
YC033104.01	RBL04.482	E	NM.030111.18	RBL11.223	E
YC033104.02	RBL04.483	E	NM.030111.19	RBL11.224	E
YC033104.03	RBL04.484	E	NM.030111.20	RBL11.225	E
YC033104.04	RBL04.485	E	NM.030111.21	RBL11.226	E

YC033104.05	RBL04.486	V	NM.030111.22	RBL11.227	E
YC033104.06	RBL04.487	B	NM.030111.23	RBL11.228	E
YC033104.07	RBL04.488	F	NM.030111.24	RBL11.229	C
YC033104.08	RBL04.489	S	NM.030111.25	RBL11.230	B
YC033104.09	RBL04.490	B	NM.030111.26	RBL11.231	K
YC033104.10	RBL04.491	A	NM.030111.27	RBL11.232	C
YC033104.11	RBL04.492	E	TC.031411.01	RBL11.233	S
YC033104.12	RBL04.493	E	TC.031411.02	RBL11.234	E
YC033104.13	RBL04.494	E	TC.031411.03	RBL11.235	E
YC033104.14	RBL04.495	E	TC.031411.04	RBL11.236	BB
YC033104.15	RBL04.496	E	TC.031411.05	RBL11.237	C
YC033104.16	RBL04.497	E	TC.031411.06	RBL11.238	E
YC033104.17	RBL04.498	V	TC.031411.07	RBL11.239	E
YC033104.18	RBL04.499	E	TC.031411.08	RBL11.240	E
YC033104.19	RBL04.500	B	TC.031411.09	RBL11.241	E
YC033104.20	RBL04.501	S	TC.031411.10	RBL11.242	E
YC033104.21	RBL04.502	E	TC.031411.11	RBL11.243	G
YC033104.22	RBL04.503	E	TC.031411.12	RBL11.244	E
YC033104.23	RBL04.504	E	TC.031411.13	RBL11.245	E
YC033104.24	RBL04.505	V	TC.031411.14	RBL11.246	E
YC033104.25	RBL04.506	E	TC.031411.15	RBL11.247	E
YC041204.01	RBL04.507	E	TC.031411.16	RBL11.248	E
YC041204.02	RBL04.508	A	TC.031411.17	RBL11.249	P
YC041204.03	RBL04.509	E	TC.031411.18	RBL11.250	E
YC041204.04	RBL04.510	E	TC.031411.19	RBL11.251	E
YC041204.05	RBL04.511	E	TC.031411.20	RBL11.252	R
YC041204.06	RBL04.512	R	TC.031411.21	RBL11.253	P
YC041204.07	RBL04.513	E	TC.031411.22	RBL11.254	C
YC041204.08	RBL04.514	E	TC.031411.23	RBL11.255	P
YC041204.09	RBL04.515	A	TC.031411.24	RBL11.256	P
YC041204.10	RBL04.516	R	NM.031411.01	RBL11.257	CC
YC041204.11	RBL04.517	A	NM.031411.02	RBL11.258	E
YC041204.12	RBL04.518	E	NM.031411.03	RBL11.259	E
YC041204.13	RBL04.519	E	NM.031411.04	RBL11.260	E
YC041204.14	RBL04.520	E	NM.031411.05	RBL11.261	E
YC041204.15	RBL04.521	E	NM.031411.06	RBL11.262	E
YC041204.16	RBL04.522	B	NM.031411.07	RBL11.263	E
YC041204.17	RBL04.523	E	NM.031411.08	RBL11.264	B
YC041204.18	RBL04.524	E	NM.031411.09	RBL11.265	J
YC041204.19	RBL04.525	E	NM.031411.10	RBL11.266	R
YC041204.20	RBL04.526	B	NM.031411.11	RBL11.267	E

YC041204.21	RBL04.527	E	NM.031411.12	RBL11.268	E
YC041204.22	RBL04.528	A	NM.031411.13	RBL11.269	E
YC041204.23	RBL04.529	E	NM.031411.14	RBL11.270	E
YC041204.24	RBL04.530	R	NM.031411.15	RBL11.271	E
YC041204.25	RBL04.531	E	NM.031411.16	RBL11.272	E
YC041204.26	RBL04.532	R	NM.031411.17	RBL11.273	E
YC041204.27	RBL04.533	E	NM.031411.18	RBL11.274	B
YC041204.28	RBL04.534	A	NM.031411.19	RBL11.275	E
YC041204.29	RBL04.535	A	NM.031411.20	RBL11.276	J
YC050404.01	RBL04.536	E	NM.031411.21	RBL11.277	E
YC050404.02	RBL04.537	E	NM.031411.22	RBL11.278	B
YC050404.03	RBL04.538	E	NM.031411.23	RBL11.279	S
YC050404.04	RBL04.539	E	NM.031411.24	RBL11.280	E
YC050404.05	RBL04.540	E	NM.031411.25	RBL11.281	E
YC050404.06	RBL04.541	E	YC.031511.01	RBL11.282	A
YC050404.07	RBL04.542	E	YC.031511.02	RBL11.283	E
YC050404.08	RBL04.543	E	YC.031511.03	RBL11.284	E
YC050404.09	RBL04.544	E	YC.031511.04	RBL11.285	R
YC050404.10	RBL04.545	E	YC.031511.05	RBL11.286	E
YC050404.11	RBL04.546	E	YC.031511.06	RBL11.287	E
YC050404.12	RBL04.547	E	YC.031511.07	RBL11.288	E
YC050404.13	RBL04.548	E	YC.031511.08	RBL11.289	E
YC050404.14	RBL04.549	E	YC.031511.09	RBL11.290	R
YC050404.15	RBL04.550	E	YC.031511.10	RBL11.291	B
YC050404.16	RBL04.551	E	YC.031511.11	RBL11.292	E
YC050404.17	RBL04.552	E	YC.031511.12	RBL11.293	E
YC050404.18	RBL04.553	E	YC.031511.13	RBL11.294	E
YC050404.19	RBL04.554	E	YC.031511.14	RBL11.295	P
YC050404.20	RBL04.555	E	YC.031511.15	RBL11.296	R
YC050404.21	RBL04.556	E	YC.031511.16	RBL11.297	E
YC050404.22	RBL04.557	E	YC.031511.17	RBL11.298	E
YC050404.23	RBL04.558	E	YC.031511.18	RBL11.299	P
YC050404.24	RBL04.559	E	YC.031511.19	RBL11.300	E
YC050404.25	RBL04.560	E	YC.031511.20	RBL11.301	E
YC050404.26	RBL04.561	E	YC.031511.21	RBL11.302	A
NM12605.01	RBL05.001	E	YC.031511.22	RBL11.303	E
NM12605.02	RBL05.002	E	YC.031511.23	RBL11.304	V
NM12605.03	RBL05.003	E	YC.032811.01	RBL11.305	E
NM12605.04	RBL05.004	I	YC.032811.02	RBL11.306	E
NM12605.05	RBL05.005	S	YC.032811.03	RBL11.307	F
NM12605.06	RBL05.006	E	YC.032811.04	RBL11.308	E

NM12605.07	RBL05.007	E	YC.032811.05	RBL11.309	C
NM12605.08	RBL05.008	S	YC.032811.06	RBL11.310	E
NM12605.09	RBL05.009	E	YC.032811.07	RBL11.311	E
NM12605.10	RBL05.010	E	YC.032811.08	RBL11.312	E
NM12605.11	RBL05.011	E	YC.032811.09	RBL11.313	P
NM12605.12	RBL05.012	E	YC.032811.10	RBL11.314	E
NM12605.13	RBL05.013	E	YC.032811.11	RBL11.315	E
NM12605.14	RBL05.014	E	YC.032811.12	RBL11.316	E
NM12605.15	RBL05.015	E	YC.032811.13	RBL11.317	E
NM12605.16	RBL05.016	E	YC.032811.14	RBL11.318	B
NM12605.17	RBL05.017	I	YC.032811.15	RBL11.319	E
NM12605.18	RBL05.018	E	YC.032811.16	RBL11.320	R
NM12605.19	RBL05.019	E	YC.032811.17	RBL11.321	R
NM12605.20	RBL05.020	E	YC.032811.18	RBL11.322	E
NM12605.21	RBL05.021	E	YC.032811.19	RBL11.323	E
NM12605.22	RBL05.022	E	YC.032811.20	RBL11.324	G
NM12605.23	RBL05.023	E	YC.032811.21	RBL11.325	G
NM12605.24	RBL05.024	E	YC.032811.22	RBL11.326	E
NM12605.25	RBL05.025	E	YC.032811.23	RBL11.327	P
NM12605.26	RBL05.026	F	YC.032811.24	RBL11.328	E
NM2905.01	RBL05.027	E	YC.032811.25	RBL11.329	S
NM2905.02	RBL05.028	H	TC.032911.01	RBL11.330	E
NM2905.03	RBL05.029	K	TC.032911.02	RBL11.331	E
NM2905.04	RBL05.030	B	TC.032911.03	RBL11.332	R
NM2905.05	RBL05.031	K	TC.032911.04	RBL11.333	E
NM2905.06	RBL05.032	E	TC.032911.05	RBL11.334	E
NM2905.07	RBL05.033	E	TC.032911.06	RBL11.335	I
NM2905.08	RBL05.034	E	TC.032911.07	RBL11.336	I
NM2905.09	RBL05.035	E	TC.032911.08	RBL11.337	E
NM2905.10	RBL05.036	E	TC.032911.09	RBL11.338	E
NM2905.11	RBL05.037	E	TC.032911.10	RBL11.339	S
NM2905.12	RBL05.038	C	TC.032911.11	RBL11.340	E
NM2905.13	RBL05.039	E	TC.032911.12	RBL11.341	E
NM2905.14	RBL05.040	E	TC.032911.13	RBL11.342	I
NM2905.15	RBL05.041	E	TC.032911.14	RBL11.343	I
NM2905.16	RBL05.042	E	TC.032911.15	RBL11.344	E
NM2905.17	RBL05.043	E	TC.032911.16	RBL11.345	E
NM2905.18	RBL05.044	E	TC.032911.17	RBL11.346	E
NM2905.19	RBL05.045	E	TC.032911.18	RBL11.347	E
NM2905.20	RBL05.046	E	TC.032911.19	RBL11.348	E
NM2905.21	RBL05.047	B	TC.032911.20	RBL11.349	E

NM2905.22	RBL05.048	E	TC.032911.21	RBL11.350	BB
NM2905.23	RBL05.049	A	TC.032911.22	RBL11.351	S
NM2905.24	RBL05.050	H	TC.032911.23	RBL11.352	R
NM2905.25	RBL05.051	G	TC.032911.24	RBL11.353	I
NM2905.26	RBL05.052	S	TC.032911.25	RBL11.354	E
NM2905.27	RBL05.053	E	WC.032911.01	RBL11.355	M
NM22505.01	RBL05.054	E	WC.032911.02	RBL11.356	E
NM22505.02	RBL05.055	E	WC.032911.03	RBL11.357	E
NM22505.03	RBL05.056	E	WC.032911.04	RBL11.358	G
NM22505.04	RBL05.057	BB	WC.032911.05	RBL11.359	E
NM22505.05	RBL05.058	D	WC.032911.06	RBL11.360	R
NM22505.06	RBL05.059	E	WC.032911.07	RBL11.361	E
NM22505.07	RBL05.060	E	WC.032911.08	RBL11.362	E
NM22505.08	RBL05.061	E	WC.032911.09	RBL11.363	E
NM22505.09	RBL05.062	A	WC.032911.10	RBL11.364	E
NM22505.10	RBL05.063	F	WC.032911.11	RBL11.365	E
NM22505.11	RBL05.064	E	WC.032911.12	RBL11.366	E
NM22505.12	RBL05.065	E	WC.032911.13	RBL11.367	F
NM22505.13	RBL05.066	A	WC.032911.14	RBL11.368	U
NM22505.14	RBL05.067	E	WC.032911.15	RBL11.369	R
NM22505.15	RBL05.068	E	WC.032911.16	RBL11.370	M
NM22505.16	RBL05.069	E	WC.032911.17	RBL11.371	A
NM22505.17	RBL05.070	E	WC.032911.18	RBL11.372	E
NM22505.18	RBL05.071	E	WC.032911.19	RBL11.373	E
NM22505.19	RBL05.072	E	WC.032911.20	RBL11.374	S
NM22505.20	RBL05.073	F	WC.032911.21	RBL11.375	E
NM22505.21	RBL05.074	E	WC.032911.22	RBL11.376	E
NM22505.22	RBL05.075	E	WC.032911.23	RBL11.377	E
NM22505.23	RBL05.076	E	WC.032911.24	RBL11.378	E
NM22505.24	RBL05.077	E	WC.032911.25	RBL11.379	R
NM22505.25	RBL05.078	E	UL..033011.01	RBL11.380	B
NM3705.01	RBL05.079	E	UL..033011.02	RBL11.381	E
NM3705.02	RBL05.080	E	UL..033011.03	RBL11.382	E
NM3705.03	RBL05.081	E	UL..033011.04	RBL11.383	E
NM3705.04	RBL05.082	S	UL..033011.05	RBL11.384	E
NM3705.05	RBL05.083	S	UL..033011.06	RBL11.385	E
NM3705.06	RBL05.084	Z	UL..033011.07	RBL11.386	E
NM3705.07	RBL05.085	S	UL..033011.08	RBL11.387	E
NM3705.08	RBL05.086	E	UL..033011.09	RBL11.388	E
NM3705.09	RBL05.087	A	UL..033011.10	RBL11.389	E
NM3705.10	RBL05.088	E	UL..033011.11	RBL11.390	B

NM3705.11	RBL05.089	E	UL..033011.12	RBL11.391	E
NM3705.12	RBL05.090	E	UL..033011.13	RBL11.392	P
NM3705.13	RBL05.091	E	UL..033011.14	RBL11.393	E
NM3705.14	RBL05.092	E	UL..033011.15	RBL11.394	E
NM3705.15	RBL05.093	E	UL..033011.16	RBL11.395	E
NM3705.16	RBL05.094	K	UL..033011.17	RBL11.396	E
NM3705.17	RBL05.095	H	UL..033011.18	RBL11.397	E
NM3705.18	RBL05.096	E	UL..033011.19	RBL11.398	E
NM3705.19	RBL05.097	E	UL..033011.20	RBL11.399	E
NM3705.20	RBL05.098	E	UL..033011.21	RBL11.400	E
NM3705.21	RBL05.099	E	UL..033011.22	RBL11.401	E
NM3705.22	RBL05.100	E	UL..033011.23	RBL11.402	E
NM3705.23	RBL05.101	E	UL..033011.24	RBL11.403	E
NM3705.24	RBL05.102	E	UL..033011.25	RBL11.404	E
NM3705.25	RBL05.103	E	NM.033111.01	RBL11.405	E
NM3705.26	RBL05.104	R	NM.033111.02	RBL11.406	E
NM3705.27	RBL05.105	E	NM.033111.03	RBL11.407	HH
NM3705.28	RBL05.106	R	NM.033111.04	RBL11.408	E
NM32105.01	RBL05.107	S	NM.033111.05	RBL11.409	A
NM32105.02	RBL05.108	E	NM.033111.06	RBL11.410	E
NM32105.03	RBL05.109	E	NM.033111.07	RBL11.411	P
NM32105.04	RBL05.110	R	NM.033111.08	RBL11.412	R
NM32105.05	RBL05.111	E	NM.033111.09	RBL11.413	R
NM32105.06	RBL05.112	E	NM.033111.10	RBL11.414	E
NM32105.07	RBL05.113	V	NM.033111.11	RBL11.415	R
NM32105.08	RBL05.114	E	NM.033111.12	RBL11.416	R
NM32105.09	RBL05.115	E	NM.033111.13	RBL11.417	E
NM32105.10	RBL05.116	B	NM.033111.14	RBL11.418	A
NM32105.11	RBL05.117	E	NM.033111.15	RBL11.419	S
NM32105.12	RBL05.118	E	NM.033111.16	RBL11.420	C
NM32105.13	RBL05.119	E	NM.033111.17	RBL11.421	S
NM32105.14	RBL05.120	E	NM.033111.18	RBL11.422	B
NM32105.15	RBL05.121	V	NM.033111.19	RBL11.423	E
NM32105.16	RBL05.122	E	NM.033111.20	RBL11.424	E
NM32105.17	RBL05.123	R	NM.033111.21	RBL11.425	S
NM32105.18	RBL05.124	E	NM.033111.22	RBL11.426	E
NM32105.19	RBL05.125	S	NM.033111.23	RBL11.427	E
NM32105.20	RBL05.126	E	NM.033111.24	RBL11.428	E
NM32105.21	RBL05.127	S	NM.033111.25	RBL11.429	C
NM32105.22	RBL05.128	E	LL.040511.01	RBL11.430	E
NM32105.23	RBL05.129	R	LL.040511.02	RBL11.431	U

NM32105.24	RBL05.130	B	LL.040511.03	RBL11.432	R
NM32105.25	RBL05.131	A	LL.040511.04	RBL11.433	E
NM32105.26	RBL05.132	E	LL.040511.05	RBL11.434	E
NM32105.27	RBL05.133	B	LL.040511.06	RBL11.435	G
NM32105.28	RBL05.134	C	LL.040511.07	RBL11.436	G
NM4505.01	RBL05.135	B	LL.040511.08	RBL11.437	BB
NM4505.02	RBL05.136	V	LL.040511.09	RBL11.438	E
NM4505.03	RBL05.137	R	LL.040511.10	RBL11.439	E
NM4505.04	RBL05.138	B	LL.040511.11	RBL11.440	S
NM4505.05	RBL05.139	B	LL.040511.12	RBL11.441	E
NM4505.06	RBL05.140	R	LL.040511.13	RBL11.442	E
NM4505.07	RBL05.141	E	LL.040511.14	RBL11.443	S
NM4505.08	RBL05.142	E	LL.040511.15	RBL11.444	R
NM4505.09	RBL05.143	S	LL.040511.16	RBL11.445	E
NM4505.10	RBL05.144	E	LL.040511.17	RBL11.446	A
NM4505.11	RBL05.145	E	LL.040511.18	RBL11.447	E
NM4505.12	RBL05.146	E	LL.040511.19	RBL11.448	E
NM4505.13	RBL05.147	E	LL.040511.20	RBL11.449	A
NM4505.14	RBL05.148	R	LL.040511.21	RBL11.450	E
NM4505.15	RBL05.149	P	LL.040511.22	RBL11.451	E
NM4505.16	RBL05.150	E	LL.040511.23	RBL11.452	J
NM4505.17	RBL05.151	E	LL.040511.24	RBL11.453	J
NM4505.18	RBL05.152	R	LL.040511.25	RBL11.454	P
NM4505.19	RBL05.153	A	WC.041411.01	RBL11.455	S
NM4505.20	RBL05.154	R	WC.041411.02	RBL11.456	E
NM4505.21	RBL05.155	F	WC.041411.03	RBL11.457	P
NM4505.22	RBL05.156	P	WC.041411.04	RBL11.458	E
NM4505.23	RBL05.157	G	WC.041411.05	RBL11.459	E
NM4505.24	RBL05.158	E	WC.041411.06	RBL11.460	E
NM4505.25	RBL05.159	E	WC.041411.07	RBL11.461	E
TC21005.01	RBL05.160	A	WC.041411.08	RBL11.462	P
TC21005.02	RBL05.161	A	WC.041411.09	RBL11.463	E
TC21005.03	RBL05.162	E	WC.041411.10	RBL11.464	E
TC21005.04	RBL05.163	E	WC.041411.11	RBL11.465	E
TC21005.05	RBL05.164	S	WC.041411.12	RBL11.466	F
TC21005.06	RBL05.165	S	WC.041411.13	RBL11.467	E
TC21005.07	RBL05.166	A	WC.041411.14	RBL11.468	II
TC21005.08	RBL05.167	E	WC.041411.15	RBL11.469	E
TC21005.09	RBL05.168	A	WC.041411.16	RBL11.470	P
TC21005.10	RBL05.169	B	WC.041411.17	RBL11.471	G
TC21005.11	RBL05.170	E	WC.041411.18	RBL11.472	Q

TC21005.12	RBL05.171	A	WC.041411.19	RBL11.473	S
TC21005.13	RBL05.172	A	WC.041411.20	RBL11.474	E
TC21005.14	RBL05.173	E	WC.041411.21	RBL11.475	A
TC21005.15	RBL05.174	E	WC.041411.22	RBL11.476	E
TC21005.16	RBL05.175	E	WC.041411.23	RBL11.477	E
TC21005.17	RBL05.176	S	WC.041411.24	RBL11.478	R
TC21005.18	RBL05.177	A	WC.041411.25	RBL11.479	A
TC21005.19	RBL05.178	E	WC.042611.01	RBL11.480	E
TC21005.20	RBL05.179	A	WC.042611.02	RBL11.481	B
TC21005.21	RBL05.180	A	WC.042611.03	RBL11.482	C
TC21005.22	RBL05.181	A	WC.042611.04	RBL11.483	J
TC21005.23	RBL05.182	E	WC.042611.05	RBL11.484	E
TC21005.24	RBL05.183	E	WC.042611.06	RBL11.485	F
TC21005.25	RBL05.184	E	WC.042611.07	RBL11.486	E
TC22305.01	RBL05.185	B	WC.042611.08	RBL11.487	E
TC22305.02	RBL05.186	R	WC.042611.09	RBL11.488	E
TC22305.03	RBL05.187	S	WC.042611.10	RBL11.489	P
TC22305.04	RBL05.188	E	WC.042611.11	RBL11.490	E
TC22305.05	RBL05.189	E	WC.042611.12	RBL11.491	B
TC22305.06	RBL05.190	S	WC.042611.13	RBL11.492	E
TC22305.07	RBL05.191	B	WC.042611.14	RBL11.493	E
TC22305.08	RBL05.192	E	WC.042611.15	RBL11.494	E
TC22305.09	RBL05.193	E	WC.042611.16	RBL11.495	E
TC22305.11	RBL05.195	R	WC.042611.17	RBL11.496	E
TC22305.12	RBL05.196	E	WC.042611.18	RBL11.497	C
TC22305.13	RBL05.197	E	WC.042611.19	RBL11.498	B
TC22305.14	RBL05.198	B	WC.042611.20	RBL11.499	S
TC22305.15	RBL05.199	E	WC.042611.21	RBL11.500	C
TC22305.16	RBL05.200	A	WC.042611.22	RBL11.501	E
TC22305.17	RBL05.201	S	WC.042611.23	RBL11.502	E
TC22305.18	RBL05.202	B	WC.042611.24	RBL11.503	E
TC22305.19	RBL05.203	R	WC.042611.25	RBL11.504	E
TC22305.20	RBL05.204	B	YC.042711.01	RBL11.505	E
TC22305.21	RBL05.205	E	YC.042711.02	RBL11.506	B
TC22305.22	RBL05.206	B	YC.042711.03	RBL11.507	E
TC22305.23	RBL05.207	R	YC.042711.04	RBL11.508	E
TC22305.24	RBL05.208	E	YC.042711.05	RBL11.509	E
TC22305.25	RBL05.209	B	YC.042711.06	RBL11.510	E
TC3705.01	RBL05.210	E	YC.042711.07	RBL11.511	R
TC3705.02	RBL05.211	B	YC.042711.08	RBL11.512	E
TC3705.03	RBL05.212	E	YC.042711.09	RBL11.513	E

TC3705.04	RBL05.213	E
TC3705.05	RBL05.214	S
TC3705.06	RBL05.215	E
TC3705.07	RBL05.216	S
TC3705.08	RBL05.217	E
TC3705.09	RBL05.218	E
TC3705.10	RBL05.219	E
TC3705.11	RBL05.220	E
TC3705.12	RBL05.221	E
TC3705.13	RBL05.222	E
TC3705.14	RBL05.223	E
TC3705.15	RBL05.224	E
TC3705.16	RBL05.225	S
TC3705.17	RBL05.226	E
TC3705.18	RBL05.227	E
TC3705.19	RBL05.228	E
TC3705.20	RBL05.229	E

YC.042711.10	RBL11.514	E
YC.042711.11	RBL11.515	R
YC.042711.12	RBL11.516	E
YC.042711.13	RBL11.517	E
YC.042711.14	RBL11.518	B
YC.042711.15	RBL11.519	R
YC.042711.16	RBL11.520	E
YC.042711.17	RBL11.521	E
YC.042711.18	RBL11.522	R
YC.042711.19	RBL11.523	E
YC.042711.20	RBL11.524	R
YC.042711.21	RBL11.525	E
YC.042711.22	RBL11.526	E
YC.042711.23	RBL11.527	B
YC.042711.24	RBL11.528	E
YC.042711.25	RBL11.529	E

Supplemental Table 3. Population genetic statistics for mtDNA haplotypes from each sample collected from 2004-2011 for razorback sucker in Lake Mohave, Arizona and Nevada; see Fig. 1 for Area locations. N is the sample size, N_h is the number of haplotypes observed, and A_R is the number of haplotypes corrected for sample size by rarefaction (Kalinowski 2005). "Sig" indicates significance ($P < 0.05$) as determined by resampling. "na" indicates statistic not calculated due to small sample size, while "-" and "ns" indicated significant and not significant, respectively. Statistics for the samples collected from 1997-2003 are reported in Dowling *et al.* (2005).

Year	Area	Date	N	N_h	H_R	sig	gene diversity	sig
2004	Nine Mile area	3-Feb	10	2	na	na	0.20+/-0.15	na
2004	Nine Mile area	6-Feb	15	8	na	na	0.73+/-0.12	na
2004	Nine Mile area	17-Feb	4	1	na	na	0	na
2004	Nine Mile area	20-Feb	25	7	6.36	ns	0.67+/-0.10	ns
2004	Nine Mile area	1-Mar	25	5	4.56	ns	0.48+/-0.12	ns
2004	Nine Mile area	15-Mar	23	7	6.34	ns	0.52+/-0.12	ns
2004	Nine Mile area	29-Mar	25	6	5.40	ns	0.72+/-0.06	ns
2004	Nine Mile area	13-Apr	21	8	7.76	ns	0.72+/-0.10	ns
2004	Tequila Cove	3-Feb	27	3	2.73	-	0.27+/-0.11	-
2004	Tequila Cove	18-Feb	25	4	3.80	-	0.68+/-0.06	ns
2004	Tequila Cove	2-Mar	25	3	2.97	-	0.45+/-0.10	ns
2004	Tequila Cove	17-Mar	29	3	2.38	-	0.14+/-0.09	-
2004	Tequila Cove	30-Mar	25	6	5.56	ns	0.75+/-0.06	ns
2004	Tequila Cove	13-Apr	27	4	3.68	-	0.44+/-0.11	-
2004	Wrong Cove	29-Mar	25	7	5.80	ns	0.43+/-0.12	ns
2004	Wrong Cove	12-Apr	25	3	3.00	-	0.55+/-0.09	ns
2004	Wrong Cove	4-May	24	5	4.81	ns	0.59+/-0.11	ns

Supplemental Table 3. Continued

2004	Yuma Cove	29-Jan	28	7	5.38	ns	0.64+/-0.09	ns
2004	Yuma Cove	17-Feb	25	5	4.53	ns	0.42+/- 0.12	ns
2004	Yuma Cove	1-Mar	23	6	5.74	ns	0.74+/-0.08	ns
2004	Yuma Cove	15-Mar	23	6	5.59	ns	0.52+/-0.12	ns
2004	Yuma Cove	31-Mar	25	6	5.56	ns	0.63+/-0.10	ns
2004	Yuma Cove	12-Apr	29	4	3.91	-	0.61+/-0.08	ns
2004	Yuma Cove	4-May	26	1	1.00	-	0	-
2005	Nine Mile area	26-Jan	26	4	3.68	-	0.35+/-0.12	-
2005	Nine Mile area	9-Feb	27	8	6.78	ns	0.60+/-0.11	ns
2005	Nine Mile area	25-Feb	25	5	4.53	ns	0.42+/-0.12	-
2005	Nine Mile area	7-Mar	28	7	5.77	ns	0.54+/-0.11	ns
2005	Nine Mile area	21-Mar	28	7	6.30	ns	0.70+/-0.09	ns
2005	Nine Mile area	5-Apr	25	9	7.96	ns	0.80+/-0.06	ns
2005	Tequila Cove	10-Feb	25	4	3.80	-	0.66+/-0.06	ns
2005	Tequila Cove	23-Feb	24	5	4.83	ns	0.76+/-0.05	ns
2005	Tequila Cove	7-Mar	28	3	2.70	-	0.26+/-0.10	-
2005	Tequila Cove	21-Mar	26	7	6.07	ns	0.65+/-0.10	ns
2005	Tequila Cove	5-Apr	24	3	2.83	-	0.36+/-0.11	-
2005	Yuma Cove	25-Jan	26	7	6.03	ns	0.67+/-0.08	ns
2005	Yuma Cove	8-Feb	26	4	3.76	-	0.60+/-0.08	ns
2005	Yuma Cove	23-Feb	25	7	6.16	ns	0.59+/-0.11	ns
2005	Yuma Cove	8-Mar	25	5	4.76	ns	0.65+/-0.09	ns
2005	Yuma Cove	22-Mar	25	6	5.56	ns	0.66+/-0.09	ns
2005	Yuma Cove	5-Apr	24	6	5.33	ns	0.58+/-0.10	ns

Supplemental Table 3. Continued

2006	Nine Mile area	7-Feb	25	9	7.99	ns	0.81+/-0.06	ns
2006	Nine Mile area	23-Feb	25	8	6.97	ns	0.73+/-0.08	ns
2006	Nine Mile area	7-Mar	25	5	4.56	ns	0.48+/-0.12	ns
2006	Nine Mile area	15-Mar	26	3	2.99	-	0.45+/-0.11	ns
2006	Nine Mile area	21-Mar	25	6	5.17	ns	0.43+/-0.12	-
2006	Nine Mile area	6-Apr	25	5	4.59	ns	0.53+/-0.11	ns
2006	Tequila Cove	8-Feb	25	4	3.97	-	0.64+/-0.08	ns
2006	Tequila Cove	21-Feb	25	4	3.97	-	0.67+/-0.07	ns
2006	Tequila Cove	7-Mar	24	6	5.64	ns	0.76+/-0.06	ns
2006	Tequila Cove	15-Mar	23	6	5.60	ns	0.57+/-0.11	ns
2006	Tequila Cove	22-Mar	25	5	4.53	ns	0.42+/-0.12	-
2006	Tequila Cove	5-Apr	24	4	3.93	ns	0.44+/-0.12	ns
2006	Tequila Cove	27-Apr	24	4	3.81	ns	0.56+/-0.09	ns
2006	Wrong Cove	22-Mar	24	6	5.48	ns	0.59+/-0.11	ns
2006	Wrong Cove	4-Apr	22	7	6.81	ns	0.80+/-0.07	ns
2006	Wrong Cove	27-Apr	25	3	2.60	-	0.16+/-0.10	-
2006	Yuma Cove	7-Feb	26	6	4.85	ns	0.35+/-0.12	-
2006	Yuma Cove	23-Feb	25	7	6.33	ns	0.63+/-0.10	ns
2006	Yuma Cove	8-Mar	25	8	7.13	ns	0.71+/-0.09	ns
2006	Yuma Cove	14-Mar	26	4	3.72	-	0.50+/-0.10	ns
2006	Yuma Cove	22-Mar	25	5	4.59	ns	0.53+/-0.11	ns
2006	Yuma Cove	5-Apr	24	6	5.60	ns	0.55+/-0.12	ns
2006	Yuma Cove	25-Apr	28	3	2.98	-	0.61+/-0.05	ns
2007	Nine Mile area	6-Feb	25	7	6.36	ns	0.75+/-0.07	ns
2007	Nine Mile area	23-Feb	5	3	na	na	0.70+/-0.22	na
2007	Nine Mile area	27-Feb	20	6	6.00	ns	0.80+/-0.05	ns
2007	Nine Mile area	15-Mar	25	5	4.53	ns	0.42+/-0.12	-

Supplemental Table 3. Continued

2007	Tequila Cove	7-Feb	25	4	3.96	-	0.60+/-0.09	ns
2007	Tequila Cove	20-Feb	23	7	6.73	ns	0.81+/-0.06	ns
2007	Tequila Cove	14-Mar	25	5	4.40	ns	0.47+/-0.11	ns
2007	Wrong Cove	5-Apr	25	7	5.97	ns	0.49+/-0.12	ns
2007	Wrong Cove	17-Apr	25	7	6.00	ns	0.62+/-0.10	ns
2007	Yuma Cove	5-Feb	25	6	5.37	ns	0.61+/-0.10	ns
2007	Yuma Cove	20-Feb	25	9	7.93	ns	0.72+/-0.09	ns
2007	Yuma Cove	12-Mar	35	7	5.91	ns	0.72+/-0.07	ns
2007	Yuma Cove	14-Mar	25	8	6.60	ns	0.49+/-0.12	ns
2008	Nine Mile area	5-Feb	25	9	8.07	ns	0.73+/-0.09	ns
2008	Nine Mile area	26-Feb	25	6	5.53	ns	0.58+/-0.11	ns
2008	Nine Mile area	3-Mar	25	7	6.00	ns	0.53+/-0.12	ns
2008	Nine Mile area	18-Mar	25	7	6.30	ns	0.59+/-0.11	ns
2008	Nine Mile area	31-Mar	25	6	5.37	ns	0.58+/-0.11	ns
2008	Nine Mile area	29-Apr	25	3	2.77	-	0.23+/-0.11	-
2008	Tequila Cove	5-Feb	25	5	4.59	ns	0.53+/-0.11	ns
2008	Tequila Cove	22-Feb	25	5	4.59	ns	0.53+/-0.11	ns
2008	Tequila Cove	4-Mar	25	7	6.36	ns	0.67+/-0.10	ns
2008	Tequila Cove	18-Mar	25	4	3.60	-	0.53+/-0.09	ns
2008	Tequila Cove	31-Mar	25	6	5.36	ns	0.53+/-0.11	ns
2008	Tequila Cove	15-Apr	25	2	2.00	-	0.52+/-0.03	ns
2008	Tequila Cove	14-May	25	6	5.79	ns	0.81+/-0.05	ns
2008	Wrong Cove	3-Apr	25	9	8.13	ns	0.81+/-0.06	ns
2008	Wrong Cove	14-Apr	25	4	3.97	-	0.64+/-0.08	ns
2008	Wrong Cove	30-Apr	25	6	5.56	ns	0.69+/-0.08	ns
2008	Wrong Cove	15-May	8	2	na	na	0.25+/-0.18	na

Supplemental Table 3. Continued

2008	Yuma Cove	6-Feb	25	6	5.53	ns	0.58+/-0.11	ns
2008	Yuma Cove	19-Feb	21	8	7.81	ns	0.72+/-0.10	ns
2008	Yuma Cove	4-Mar	25	3	2.93	-	0.29+/-0.11	-
2008	Yuma Cove	18-Mar	25	7	5.80	ns	0.43+/-0.12	ns
2008	Yuma Cove	1-Apr	25	2	1.80	-	0.08+/-0.07	-
2008	Yuma Cove	14-Apr	22	5	4.82	ns	0.75+/-0.05	ns
2008	Yuma Cove	30-Apr	25	5	4.76	ns	0.70+/-0.07	ns
2009	Nine Mile area	6-Feb	25	7	6.36	ns	0.71+/-0.09	ns
2009	Nine Mile area	17-Feb	25	7	6.73	ns	0.82+/-0.05	ns
2009	Nine Mile area	2-Mar	25	5	4.57	ns	0.60+/-0.09	ns
2009	Nine Mile area	18-Mar	25	5	4.73	ns	0.53+/-0.11	ns
2009	Nine Mile area	31-Mar	24	4	3.66	ns	0.37+/-0.18	-
2009	Tequila Cove	6-Feb	25	3	3.00	-	0.61+/-0.07	ns
2009	Tequila Cove	17-Feb	25	6	5.56	ns	0.74+/-0.06	ns
2009	Tequila Cove	2-Mar	25	5	4.70	ns	0.48+/-0.12	ns
2009	Tequila Cove	16-Mar	25	4	3.80	-	0.59+/-0.09	ns
2009	Tequila Cove	31-Mar	25	2	2.00	-	0.38+/-0.09	-
2009	Tequila Cove	13-Apr	25	4	4.00	-	0.74+/-0.05	ns
2009	Wrong Cove	17-Mar	25	6	5.57	ns	0.70+/-0.08	ns
2009	Wrong Cove	31-Mar	24	7	6.33	ns	0.74+/-0.07	ns
2009	Wrong Cove	13-Apr	25	7	6.33	ns	0.63+/-0.10	ns
2009	Yuma Cove	6-Feb	21	5	4.86	ns	0.42+/-0.13	ns
2009	Yuma Cove	17-Feb	25	5	4.56	ns	0.48+/-0.12	ns
2009	Yuma Cove	2-Mar	23	7	6.40	ns	0.82+/-0.04	ns
2009	Yuma Cove	18-Mar	25	7	6.16	ns	0.59+/-0.11	ns
2009	Yuma Cove	20-Mar	23	9	8.20	ns	0.72+/-0.10	ns
2009	Yuma Cove	31-Mar	25	6	5.57	ns	0.76+/-0.05	ns
2009	Yuma Cove	17-Apr	25	4	4.00	-	0.75+/-0.04	ns

Supplemental Table 3. Continued

2010	Nine Mile area	3-Feb	25	7	6.53	ns	0.74+/-0.08	ns
2010	Nine Mile area	16-Feb	25	7	6.36	ns	0.75+/-0.07	ns
2010	Nine Mile area	3-Mar	25	5	4.76	ns	0.58+/-0.11	ns
2010	Nine Mile area	16-Mar	25	4	3.80	-	0.56+/-0.10	ns
2010	Nine Mile area	30-Mar	24	7	6.43	ns	0.61+/-0.12	ns
2010	Tequila Cove	1-Feb	25	3	2.80	-	0.55+/-0.05	ns
2010	Tequila Cove	16-Feb	25	1	1.00	-	0	-
2010	Tequila Cove	2-Mar	25	6	5.17	ns	0.43+/-0.12	ns
2010	Tequila Cove	16-Mar	23	4	3.86	ns	0.50+/-0.11	ns
2010	Tequila Cove	30-Mar	25	7	6.17	ns	0.71+/-0.07	ns
2010	Tequila Cove	13-Apr	25	9	7.90	ns	0.69+/-0.10	ns
2010	Wrong Cove	10-Mar	25	4	3.73	-	0.36+/-0.12	-
2010	Wrong Cove	29-Mar	25	6	5.53	ns	0.58+/-0.11	ns
2010	Wrong Cove	15-Apr	29	6	5.27	ns	0.66+/-0.08	ns
2010	Yuma Cove	1-Feb	25	5	4.40	ns	0.42+/-0.12	-
2010	Yuma Cove	18-Feb	25	6	5.50	ns	0.54+/-0.12	ns
2010	Yuma Cove	1-Mar	25	5	6.96	ns	0.48+/-0.12	ns
2010	Yuma Cove	16-Mar	25	7	6.00	ns	0.54+/-0.12	ns
2010	Yuma Cove	29-Mar	27	7	6.15	ns	0.67+/-0.09	ns
2011	Nine Mile area	31-Jan	25	6	5.40	ns	0.68+/-0.08	ns
2011	Nine Mile area	24-Feb	24	6	5.48	ns	0.55+/-0.11	ns
2011	Nine Mile area	1-Mar	27	7	5.84	ns	0.51+/-0.12	ns
2011	Nine Mile area	14-Mar	25	6	5.36	ns	0.53+/-0.11	ns
2011	Nine Mile area	31-Mar	25	8	7.33	ns	0.78+/-0.07	ns

Supplemental Table 3. Continued

2011	Tequila Cove	3-Feb	25	3	3.00	-	0.57+/-0.08	ns
2011	Tequila Cove	15-Feb	24	5	4.62	ns	0.44+/-0.12	ns
2011	Tequila Cove	1-Mar	24	7	6.00	ns	0.45+/-0.13	ns
2011	Tequila Cove	14-Mar	24	7	6.31	ns	0.65+/-0.10	ns
2011	Tequila Cove	29-Mar	25	5	4.73	ns	0.61+/-0.10	ns
2011	Wrong Cove	29-Mar	25	8	6.96	ns	0.64+/-0.11	ns
2011	Wrong Cove	14-Apr	25	9	7.93	ns	0.72+/-0.09	ns
2011	Wrong Cove	26-Apr	25	7	6.19	ns	0.63+/-0.10	ns
2011	Yuma Cove	3-Feb	23	5	4.60	ns	0.39+/-0.13	-
2011	Yuma Cove	15-Feb	24	7	6.16	ns	0.55+/-0.12	ns
2011	Yuma Cove	28-Feb	26	8	6.85	ns	0.77+/-0.06	ns
2011	Yuma Cove	15-Mar	23	6	5.71	ns	0.62+/-0.11	ns
2011	Yuma Cove	28-Mar	25	8	7.10	ns	0.64+/-0.11	ns
2011	Yuma Cove	27-Apr	25	3	3.00	-	0.50+/-0.10	ns

Supplemental Table 4. Microsatellite genotypes for individuals examined in this study. Sample ID provides the lab assigned identification number for each individual. 0 indicates missing data.

Sample ID	Xte1	Xte1	Xte2	Xte2	Xte7	Xte7	Xte8	Xte8	Xte10	Xte10	Xte11	Xte11	Xte12	Xte12	Xte16	Xte16
97rbl.002	135	137	76	76	140	156	224	224	249	253	298	298	160	194	216	248
97rbl.008	135	137	76	76	144	152	224	230	239	255	298	298	202	202	222	224
97rbl.009	135	135	76	76	144	144	224	238	247	251	292	298	196	198	220	226
97rbl.010	135	137	76	76	144	174	224	230	239	255	300	300	202	202	236	240
97rbl.011	137	137	76	76	144	144	226	230	239	255	322	322	202	202	214	220
97rbl.018	137	137	76	76	144	156	222	260	239	251	298	302	160	200	228	234
97rbl.021	137	137	76	78	144	144	222	224	239	247	322	322	200	202	214	234
97rbl.029	137	137	76	76	144	188	230	240	239	245	296	300	202	202	216	252
97rbl.031	137	137	76	76	144	158	224	230	239	247	298	300	160	200	222	234
97rbl.032	137	137	76	76	144	144	232	240	239	251	296	302	160	200	232	236
97rbl.034	135	137	76	76	144	144	224	230	235	239	294	298	160	196	212	244
97rbl.040	137	137	76	76	144	144	228	248	239	247	294	296	200	202	234	238
97rbl.041	137	137	76	76	144	144	232	248	239	241	292	294	160	202	232	234
97rbl.043	137	137	76	76	156	156	224	226	247	247	296	300	198	202	212	250
97rbl.045	135	137	76	76	144	144	224	232	253	253	288	296	202	202	220	222
97rbl.048	135	137	76	78	144	152	232	236	247	249	304	304	200	200	190	238
97rbl.051	137	137	72	76	156	156	230	236	239	247	294	296	200	202	234	236
97rbl.055	137	137	76	76	140	144	220	230	239	247	286	296	196	200	234	238
97rbl.062	137	137	76	76	162	180	226	230	239	251	296	298	202	204	232	242
97rbl.068	137	137	76	76	144	156	220	246	239	251	294	302	202	210	236	240
97rbl.070	137	137	74	76	144	156	252	252	239	251	292	302	202	204	220	238
97rbl.072	135	137	76	78	144	144	222	224	241	247	292	300	160	202	226	248
97rbl.076	137	137	76	76	144	144	224	240	245	247	292	304	160	200	214	236
97rbl.077	135	137	76	76	160	160	220	222	251	251	294	300	196	202	228	236
97rbl.080	135	137	76	76	144	156	220	230	247	251	294	298	196	200	234	234
97rbl.082	137	137	76	76	144	156	226	234	239	239	302	306	194	200	218	236
97rbl.084	137	137	76	76	144	160	222	224	241	249	296	300	200	204	212	218
97rbl.085	137	137	76	76	156	188	238	256	239	249	300	300	160	196	220	238
97rbl.095	137	137	76	76	156	160	230	240	245	247	296	298	198	198	226	240
97rbl.102	135	137	76	78	144	156	222	240	247	247	296	300	196	200	220	240
97rbl.105	135	137	76	78	144	158	224	224	245	247	294	302	196	200	232	234
97rbl.106	135	137	76	76	144	158	234	238	239	245	296	298	200	204	190	190
97rbl.107	135	135	76	76	144	158	212	230	245	245	300	308	196	200	238	240
97rbl.108	137	137	76	76	154	164	222	232	245	251	300	300	196	196	222	222

97rbl.110	137	137	76	76	144	156	224	228	239	241	294	316	160	200	222	226
97rbl.111	137	137	76	78	144	156	230	248	245	247	296	298	198	202	222	248
97rbl.112	137	137	76	76	152	156	226	230	237	249	296	296	200	202	220	220
97rbl.113	137	137	76	76	144	158	222	222	247	247	294	302	194	206	216	238
97rbl.117	135	137	76	76	160	170	220	248	247	249	296	302	200	206	238	240
97rbl.120	137	137	76	76	144	156	232	248	247	247	292	296	194	202	216	238
97rbl.122	137	137	76	76	144	144	224	230	239	247	294	308	196	200	212	238
97rbl.125	135	137	76	76	144	144	220	224	247	247	302	308	200	200	218	238
97rbl.130	135	137	76	76	144	144	226	234	241	245	298	298	196	202	212	220
97rbl.132	137	137	76	78	144	156	232	236	241	251	302	332	200	206	234	238
97rbl.133	135	137	76	76	144	156	230	234	239	241	294	302	160	160	222	226
97rbl.138	135	137	76	76	144	158	224	230	245	251	298	302	200	202	220	238
97rbl.141	135	137	76	76	144	158	242	248	247	247	294	298	194	202	212	222
97rbl.143	137	137	76	76	156	160	224	230	245	245	302	302	196	206	218	236
97rbl.144	137	137	76	76	162	188	224	240	245	249	300	306	200	202	198	234
97rbl.145	137	137	76	78	144	156	214	224	239	239	300	300	200	200	228	240
97rbl.146	137	137	76	76	144	156	224	232	237	245	302	308	192	202	232	238
97rbl.150	137	137	76	76	156	164	226	238	245	249	302	308	200	202	212	232
97rbl.160	137	137	76	76	156	158	226	232	249	253	294	298	200	204	192	194
97rbl.163	137	137	76	76	140	156	232	240	241	249	296	296	200	204	192	238
97rbl.166	137	137	72	76	144	152	224	248	245	245	300	302	198	200	222	250
97rbl.168	137	137	76	78	144	158	224	238	245	249	294	300	198	198	210	224
97rbl.169	137	137	76	76	144	158	230	260	239	241	294	296	160	206	222	252
97rbl.170	135	137	76	76	144	144	224	232	247	249	298	300	196	200	234	244
97rbl.174	135	135	74	78	144	160	226	240	241	245	292	302	202	202	212	242
97rbl.175	137	137	76	76	156	156	230	248	239	249	296	298	200	210	232	236
97rbl.177	135	137	76	76	144	160	224	238	245	245	292	302	200	202	238	242
97rbl.185	135	137	76	76	144	158	230	248	245	247	300	302	196	200	242	258
97rbl.187	135	137	76	78	144	180	240	264	239	251	292	308	198	200	232	240
97rbl.193	135	137	76	76	144	144	222	248	245	251	292	296	200	200	232	240
97rbl.194	137	137	76	76	164	176	212	230	249	251	304	306	198	204	220	226
97rbl.195	137	137	76	76	144	144	222	248	239	241	304	304	202	204	222	250
97rbl.196	137	137	76	76	144	180	230	264	241	253	290	296	198	200	236	238
97rbl.199	137	137	76	76	158	162	224	248	247	255	294	302	202	204	238	246
97rbl.200	137	137	76	76	144	144	222	242	251	251	296	304	196	200	234	256
97rbl.208	137	137	76	76	144	162	222	230	235	239	292	296	196	200	222	230
97rbl.209	137	137	76	76	158	160	224	224	239	247	300	302	202	202	194	194

97rbl.211	137	137	76	76	144	180	222	232	239	251	302	304	200	204	226	260
97rbl.213	135	137	76	78	166	180	224	232	245	247	302	304	202	202	240	252
97rbl.215	137	137	76	78	144	156	224	248	247	255	288	294	200	202	226	234
97rbl.216	137	137	76	76	156	156	212	238	241	253	292	296	196	202	218	232
97rbl.221	137	137	76	76	144	156	240	252	245	249	298	300	196	200	232	234
97rbl.224	137	137	76	76	144	146	220	248	249	249	300	302	194	200	192	234
97rbl.226	135	137	76	78	144	158	222	238	239	249	304	304	196	202	240	252
97rbl.228	135	137	76	76	144	156	232	240	239	247	312	312	160	196	228	242
97rbl.232	135	137	76	76	144	158	222	224	241	241	302	306	196	206	230	250
97rbl.235	137	137	76	76	150	156	222	238	235	249	300	306	202	210	240	252
97rbl.237	137	137	76	76	144	144	222	240	245	247	294	296	198	200	226	242
97rbl.238	135	135	76	76	144	152	224	230	241	245	300	308	202	202	234	238
97rbl.239	137	137	76	76	144	154	230	240	245	247	292	300	198	206	192	238
97rbl.241	135	137	76	76	144	144	212	240	237	251	298	304	160	198	242	242
97rbl.243	137	137	76	76	162	162	224	236	251	251	294	294	200	202	222	240
97rbl.246	137	137	76	76	144	144	214	224	239	249	300	300	202	202	216	238
97rbl.251	137	137	76	76	144	144	226	242	235	239	294	302	198	202	222	240
97rbl.252	135	137	76	76	144	156	222	232	239	255	296	300	200	200	214	242
97rbl.255	137	137	76	76	144	156	224	230	247	247	298	300	196	196	238	238
97rbl.260	135	135	76	76	144	156	212	248	253	253	302	304	160	200	220	232
97rbl.269	137	137	76	76	144	156	230	240	249	253	294	296	200	200	238	266
97rbl.272	137	137	76	76	144	156	220	222	249	249	294	302	202	202	226	226
97rbl.280	137	137	72	76	144	144	222	232	235	247	294	298	196	204	234	236
97rbl.283	137	137	76	76	152	156	230	230	245	247	292	294	196	200	220	242
97rbl.289	135	137	76	76	144	152	222	224	249	249	288	292	196	198	238	270
97rbl.294	137	137	76	76	144	156	230	234	245	253	308	318	200	202	234	234
97rbl.295	137	137	76	76	156	164	212	228	251	251	298	300	200	200	232	240
97rbl.297	137	137	76	76	158	158	224	230	235	235	296	300	200	204	220	234
97rbl.303	137	137	76	76	156	166	230	238	247	247	296	298	200	200	226	238
97rbl.305	137	137	76	76	152	156	224	230	247	247	292	300	200	206	220	236
97rbl.308	137	137	76	76	152	158	222	236	245	251	296	300	196	202	192	224
97rbl.312	137	137	74	76	144	164	212	222	239	249	300	300	204	204	216	242
97rbl.318	137	137	76	76	158	158	226	230	239	251	294	302	202	214	212	220
97rbl.324	135	137	76	76	144	144	226	230	245	251	270	300	200	202	220	234
97rbl.328	135	137	76	76	144	156	224	228	239	247	294	306	180	204	220	220
97rbl.331	137	137	76	76	144	162	234	238	239	255	296	298	196	202	192	226
97rbl.333	135	137	76	76	144	190	232	232	235	251	298	300	196	204	220	222

97rbl.335	137	137	76	78	152	156	222	230	239	245	302	302	196	204	218	232
97rbl.336	137	137	76	76	144	156	230	236	237	245	300	302	198	202	218	238
97rbl.339	135	137	76	76	158	184	234	252	235	239	296	298	196	202	214	220
97rbl.347	135	137	76	76	144	168	220	236	247	255	296	296	196	212	232	236
97rbl.349	137	137	76	76	144	144	224	230	239	249	294	300	202	204	192	236
97rbl.351	137	137	76	76	144	170	224	236	239	247	298	300	200	204	218	226
97rbl.357	137	137	76	76	144	144	226	240	239	249	304	308	196	200	192	234
97rbl.359	135	137	76	76	152	162	224	238	235	247	294	302	202	202	212	236
97rbl.364	137	137	76	76	144	152	222	230	247	247	300	318	200	200	236	240
97rbl.365	135	137	76	76	144	162	224	232	239	249	288	296	160	200	220	222
97rbl.368	137	137	74	76	156	156	220	222	239	245	296	302	196	200	222	236
97rbl.370	137	137	76	76	144	144	230	232	249	249	296	304	200	200	230	254
98rbl.001	135	137	76	76	152	176	224	230	247	249	298	308	198	202	224	236
98rbl.002	137	137	76	76	144	158	228	230	239	247	298	304	160	204	238	242
98rbl.007	137	137	72	76	144	168	230	246	237	253	296	300	200	200	240	246
98rbl.008	137	137	74	78	144	152	220	224	235	251	302	304	198	200	220	236
98rbl.010	137	137	76	76	156	156	222	234	241	249	296	296	196	200	220	244
98rbl.020	137	137	76	76	156	156	224	228	241	247	296	298	200	202	240	240
98rbl.021	137	137	76	76	152	156	220	230	237	253	300	302	196	200	192	242
98rbl.026	137	137	76	76	144	156	214	224	241	241	302	302	198	202	220	238
98rbl.031	137	137	76	76	144	156	234	236	245	247	298	300	180	202	220	248
98rbl.034	137	137	74	76	144	156	222	230	241	247	294	298	200	202	238	250
98rbl.041	135	137	76	76	144	166	224	236	239	247	298	302	202	202	222	224
98rbl.042	137	137	74	76	144	156	224	244	249	249	294	294	160	202	248	250
98rbl.045	137	137	76	76	156	156	230	236	249	251	298	302	180	200	220	238
98rbl.048	137	137	76	76	156	156	224	236	245	249	298	300	202	204	220	232
98rbl.049	137	137	76	76	144	158	222	224	235	255	300	302	200	202	234	238
98rbl.066	137	137	76	76	144	144	222	230	245	249	298	300	160	196	232	252
98rbl.072	135	137	76	76	144	156	222	238	239	239	294	302	160	200	192	234
98rbl.073	137	137	76	76	144	158	224	240	251	255	300	306	196	202	218	242
98rbl.082	137	137	76	76	144	144	224	226	241	249	302	304	200	200	214	218
98rbl.086	137	137	76	76	156	160	224	242	245	245	296	302	200	204	220	238
98rbl.088	137	137	76	76	144	144	224	244	239	247	296	306	196	202	226	234
98rbl.090	137	137	76	76	144	144	224	236	247	247	300	302	200	202	220	240
98rbl.098	137	137	76	76	144	164	224	226	239	247	302	304	180	200	222	240
98rbl.100	137	137	76	76	144	144	224	224	235	251	292	300	202	202	240	242
98rbl.102	135	137	76	76	156	164	212	230	247	247	300	300	160	204	232	234

98rbl.104	137	137	76	76	156	166	220	224	235	245	296	298	160	200	234	236
98rbl.114	137	137	76	76	144	144	212	230	247	251	298	300	160	200	220	240
98rbl.120	137	137	76	76	144	162	224	230	239	249	298	304	202	202	220	238
98rbl.122	137	137	76	76	144	156	224	224	235	247	298	302	196	202	238	240
98rbl.127	137	137	76	76	156	180	224	228	245	249	298	302	200	200	226	236
98rbl.128	137	137	76	76	158	180	224	230	239	253	296	298	200	202	226	236
98rbl.131	137	137	76	76	144	144	224	224	247	247	296	302	202	206	238	240
98rbl.137	137	137	76	76	144	144	240	242	241	249	296	300	196	202	236	238
98rbl.140	137	137	76	76	144	144	220	264	247	255	298	300	202	206	234	234
98rbl.150	137	137	76	76	144	144	226	234	245	247	296	296	196	198	222	238
98rbl.151	135	135	76	78	144	152	212	224	237	249	302	302	180	200	220	234
98rbl.153	137	137	76	76	144	156	232	242	247	249	294	298	198	200	218	240
98rbl.156	137	137	76	76	144	156	230	260	239	239	292	304	200	202	192	234
98rbl.158	137	137	72	76	144	156	222	230	245	245	292	302	192	206	236	238
98rbl.160	137	137	76	76	144	156	230	260	239	239	294	304	200	202	220	234
98rbl.164	137	137	76	76	144	156	228	260	247	251	294	298	198	200	234	234
98rbl.165	135	137	76	76	152	158	212	228	237	247	296	302	200	204	222	242
98rbl.169	135	137	76	76	144	144	228	240	245	247	292	300	200	200	236	256
98rbl.173	135	137	76	76	144	144	224	238	247	247	296	300	200	200	234	242
98rbl.175	135	137	76	76	144	144	224	230	245	249	294	304	200	210	230	240
98rbl.183	137	137	76	76	144	152	234	242	247	249	302	310	196	202	240	240
98rbl.188	137	137	76	76	156	156	224	224	235	245	296	296	206	208	230	252
98rbl.190	137	137	76	76	156	188	226	232	245	247	300	314	196	202	194	232
98rbl.194	135	137	74	76	156	184	228	230	247	247	298	302	196	198	218	220
98rbl.196	137	137	76	76	144	188	232	242	247	247	296	300	200	202	232	236
98rbl.197	137	137	76	76	156	162	230	248	239	251	300	308	198	202	234	242
98rbl.205	137	137	76	76	144	184	230	232	247	249	302	302	198	202	218	220
98rbl.207	135	137	76	76	144	156	236	244	235	239	298	306	200	200	234	254
98rbl.215	137	137	76	76	144	144	220	224	247	247	292	300	198	202	222	236
98rbl.218	137	137	76	78	144	152	230	240	237	245	296	302	200	202	240	242
98rbl.219	135	137	76	76	144	174	222	248	247	251	270	298	208	208	232	254
98rbl.221	137	137	76	76	150	158	230	242	251	251	296	306	196	200	224	236
98rbl.224	135	137	76	76	144	174	228	256	239	239	294	310	160	196	236	236
98rbl.228	137	137	76	78	156	156	224	224	239	239	294	300	200	202	234	240
98rbl.233	135	137	76	76	158	162	222	226	249	255	298	302	200	202	236	240
98rbl.235	137	137	76	76	156	158	230	236	253	253	296	310	202	204	236	236
98rbl.244	137	137	76	76	144	144	232	264	247	255	300	304	198	198	218	220

98rbl.245	137	137	76	76	144	144	224	226	239	251	296	298	196	202	220	240
98rbl.249	137	137	76	76	144	144	232	238	241	247	296	300	196	202	220	254
98rbl.252	135	137	76	76	144	156	222	230	235	235	294	302	196	200	234	236
98rbl.254	137	137	76	78	144	144	230	230	239	249	300	324	200	202	238	252
98rbl.261	137	137	76	76	156	156	230	230	245	251	294	300	200	200	234	236
98rbl.267	137	137	76	76	150	158	224	230	239	247	300	302	202	202	220	254
98rbl.271	137	137	76	76	152	152	228	248	251	251	298	310	180	200	238	238
98rbl.274	137	137	76	76	144	144	224	232	239	239	298	308	198	200	212	220
98rbl.276	137	137	72	76	144	144	224	226	237	241	300	308	200	200	214	214
98rbl.282	135	137	76	76	158	158	214	224	249	249	302	308	200	200	224	224
98rbl.286	135	137	76	76	144	144	230	232	239	253	298	302	160	210	224	236
98rbl.289	137	137	76	78	158	176	228	238	245	249	300	310	204	206	220	254
98rbl.293	137	137	76	78	156	176	230	230	235	235	304	310	200	206	220	242
98rbl.298	137	137	76	76	152	176	244	248	247	247	300	302	202	202	194	234
98rbl.302	137	137	76	76	144	144	224	230	0	0	298	310	180	200	214	234
98rbl.309	137	137	76	76	144	182	224	228	241	245	298	308	198	200	216	238
98rbl.310	137	137	76	76	144	146	228	230	241	251	302	304	200	200	234	238
98rbl.311	137	137	76	76	144	144	230	230	249	249	302	310	200	200	214	234
98rbl.323	137	137	76	76	156	156	232	232	247	251	298	310	160	198	212	242
98rbl.326	137	137	76	78	144	144	234	238	247	251	296	302	196	196	218	234
98rbl.327	137	137	76	76	156	188	222	230	235	237	294	300	202	202	220	226
98rbl.335	137	137	76	76	144	144	224	230	247	247	292	304	200	202	238	262
98rbl.342	135	137	76	76	144	156	222	240	241	247	294	296	200	202	192	226
98rbl.346	137	137	76	76	144	150	222	242	245	249	298	298	200	202	220	228
98rbl.357	137	137	76	76	144	168	242	248	241	247	298	302	200	200	238	268
98rbl.361	137	137	76	76	144	144	230	248	247	247	292	300	200	200	218	250
98rbl.363	137	137	76	76	144	144	220	230	245	249	298	302	202	202	212	220
98rbl.369	137	137	76	76	144	188	230	240	245	247	296	302	196	200	220	238
98rbl.372	137	137	76	76	144	162	222	242	247	249	298	300	198	202	236	236
98rbl.374	137	137	76	76	144	166	220	242	247	251	296	302	200	200	238	268
98rbl.375	137	137	72	76	162	162	222	230	235	235	298	300	160	204	232	234
98rbl.380	137	137	76	76	144	156	222	224	237	247	296	304	196	204	214	230
98rbl.388	137	137	76	76	144	158	226	240	247	249	286	300	196	200	236	236
98rbl.391	135	135	76	76	156	192	220	220	251	255	294	310	196	200	222	228
98rbl.392	137	137	76	76	152	152	220	228	245	247	298	300	202	202	232	254
98rbl.393	137	137	76	76	144	158	212	222	239	249	286	302	196	196	232	242
98rbl.395	137	137	76	76	144	144	224	240	235	251	302	304	194	202	220	238

98rbl.396	137	137	76	76	144	176	222	248	235	247	296	302	196	206	212	220
98rbl.397	137	137	76	76	156	198	230	232	249	251	298	302	200	200	250	250
98rbl.400	137	137	76	76	154	156	238	264	235	251	302	304	194	202	238	244
98rbl.402	137	137	76	78	144	144	234	244	245	245	298	302	160	206	220	242
98rbl.403	137	137	76	76	152	152	214	238	239	247	302	306	196	200	218	232
98rbl.408	137	137	76	76	144	156	238	248	235	245	294	300	196	202	220	226
98rbl.410	135	137	76	76	156	156	222	228	237	245	294	298	196	202	216	240
98rbl.417	137	137	76	76	144	144	220	228	247	247	302	326	200	202	230	252
98rbl.420	137	137	76	76	158	160	230	242	247	251	300	308	196	208	232	236
98rbl.428	137	137	76	76	156	180	234	248	235	239	300	308	200	202	220	244
98rbl.431	137	137	74	76	144	152	220	248	249	249	292	302	200	202	244	244
98rbl.434	137	137	76	76	144	144	222	234	247	249	300	300	196	202	234	250
98rbl.437	135	137	76	76	144	156	222	230	247	247	294	296	202	204	212	244
98rbl.439	137	137	76	76	144	170	230	260	237	255	298	306	200	210	192	238
98rbl.440	135	137	76	76	144	144	222	238	235	247	300	306	200	200	226	242
98rbl.456	137	137	76	76	144	156	228	230	235	249	296	300	160	202	228	236
98rbl.458	135	137	76	76	144	156	230	230	247	247	306	306	202	210	212	222
98rbl.464	135	137	76	78	158	162	230	248	239	255	296	302	200	202	192	240
98rbl.474	135	137	76	76	144	144	224	224	247	249	298	306	200	202	222	242
98rbl.477	135	135	76	76	156	158	224	230	235	245	300	302	160	196	244	244
98rbl.488	135	137	76	76	144	170	232	242	237	251	288	296	200	210	236	240
99rbl.005	137	137	76	78	152	156	220	230	237	247	300	302	196	206	218	222
99rbl.007	137	137	76	76	144	156	222	224	247	251	292	298	196	202	234	236
99rbl.008	137	137	76	76	144	156	224	234	247	247	296	300	160	200	218	230
99rbl.012	137	137	76	76	144	170	222	230	245	249	298	318	196	198	220	244
99rbl.019	137	137	76	76	144	144	236	240	247	255	292	294	196	200	192	222
99rbl.023	137	137	76	76	144	194	220	240	245	249	298	298	200	200	192	236
99rbl.025	137	137	76	76	140	156	222	224	237	249	296	296	178	202	192	228
99rbl.026	135	137	76	76	144	188	222	230	237	245	294	302	200	202	234	234
99rbl.027	137	137	76	78	144	172	220	248	241	241	296	302	202	204	212	218
99rbl.029	135	137	76	76	144	144	242	248	237	239	296	304	202	204	216	252
99rbl.031	135	137	76	76	144	160	228	230	235	241	294	304	196	202	220	236
99rbl.034	137	137	76	76	144	180	224	230	245	245	296	302	200	204	224	232
99rbl.036	137	137	76	78	144	144	222	238	249	251	290	302	200	202	218	232
99rbl.037	137	137	76	76	144	144	234	264	245	245	304	306	200	200	232	248
99rbl.041	137	137	76	76	156	162	230	240	245	249	292	302	200	202	236	240
99rbl.044	137	137	76	76	144	156	224	230	249	249	292	296	200	202	220	236

99rbl.046	137	137	76	78	152	156	222	222	245	249	298	302	200	204	220	232
99rbl.050	135	137	76	76	144	156	222	242	235	247	296	302	196	200	222	226
99rbl.051	137	137	76	76	144	156	222	238	245	249	294	296	200	204	198	254
99rbl.053	137	137	76	76	144	152	238	264	245	245	290	300	200	202	220	248
99rbl.054	137	137	76	78	144	156	222	234	249	249	298	302	200	204	218	220
99rbl.058	135	137	76	76	184	184	222	228	241	255	296	304	196	196	218	244
99rbl.060	135	137	76	76	152	156	224	236	245	245	300	306	196	204	218	240
99rbl.063	135	137	76	76	144	156	228	246	241	245	298	334	204	206	230	250
99rbl.066	135	137	76	76	156	164	230	246	247	253	302	320	202	204	234	248
99rbl.067	137	137	76	76	156	166	224	238	245	249	300	334	200	206	226	230
99rbl.068	137	137	76	76	144	158	224	226	237	247	294	310	160	202	226	234
99rbl.069	137	137	76	76	144	156	230	242	247	249	298	304	202	210	234	236
99rbl.072	135	137	76	76	144	156	222	238	239	249	294	298	202	202	226	230
99rbl.075	137	137	76	76	144	156	222	224	237	239	292	300	196	196	230	236
99rbl.076	137	137	76	76	144	188	222	230	235	251	298	302	198	214	234	252
99rbl.077	135	137	76	76	144	180	230	230	235	247	296	300	200	200	220	234
99rbl.078	135	137	76	76	144	180	226	230	245	247	292	306	196	202	234	254
99rbl.079	137	137	76	76	144	144	224	234	245	247	300	318	202	202	236	252
99rbl.083	135	137	76	76	144	158	230	240	237	249	300	302	160	160	220	236
99rbl.084	137	137	76	76	144	160	232	238	249	249	294	308	200	200	220	250
99rbl.088	135	137	76	76	144	158	226	230	239	245	296	298	202	208	220	228
99rbl.089	135	137	76	76	144	158	212	240	249	249	298	302	198	206	194	236
99rbl.090	135	137	76	76	144	144	222	224	239	251	296	302	198	200	218	242
99rbl.092	137	137	76	76	156	156	224	252	235	241	296	310	202	208	212	236
99rbl.098	137	137	76	76	144	164	220	248	239	245	296	308	196	208	228	236
99rbl.099	135	137	76	78	144	180	222	240	237	239	302	302	202	206	194	258
99rbl.104	135	137	76	76	144	144	224	230	239	249	300	300	160	202	192	220
99rbl.111	137	137	76	76	144	156	232	238	239	249	304	304	198	202	232	250
99rbl.112	137	137	76	76	156	156	222	232	239	249	298	306	200	202	220	220
99rbl.115	135	135	76	76	152	156	230	240	237	245	300	304	160	200	220	238
99rbl.119	135	137	76	76	144	144	222	224	241	241	296	296	196	200	220	228
99rbl.120	137	137	76	76	144	152	230	240	239	251	300	304	200	206	220	236
99rbl.123	137	137	76	76	144	144	224	224	239	251	300	308	202	202	214	236
99rbl.126	137	137	76	76	144	152	228	240	247	251	294	296	198	200	212	230
99rbl.127	137	137	76	76	144	152	222	224	245	249	290	302	202	202	198	220
99rbl.128	137	137	76	76	144	144	222	246	245	255	298	300	196	202	230	240
99rbl.129	137	137	76	76	144	144	224	224	237	245	304	304	196	196	220	226

99rbl.130	137	137	76	76	144	152	222	228	245	249	300	304	202	206	198	220
99rbl.131	135	137	76	76	144	144	224	226	249	249	296	296	202	202	232	236
99rbl.133	137	137	72	76	152	158	224	232	247	251	296	298	202	202	220	226
99rbl.135	137	137	76	76	144	182	222	238	245	249	298	300	204	212	230	244
99rbl.136	137	137	76	76	144	144	226	232	241	247	302	326	202	206	232	244
99rbl.137	135	137	76	76	144	156	214	230	251	251	300	310	198	202	236	242
99rbl.138	137	137	76	76	144	156	220	222	251	251	298	304	194	204	238	242
99rbl.140	137	137	76	78	144	158	222	224	249	255	300	304	202	204	220	236
99rbl.147	135	137	76	76	144	156	222	232	239	239	298	304	204	206	232	248
99rbl.149	137	137	76	76	156	156	230	244	237	241	300	300	202	206	230	238
99rbl.150	137	137	76	76	144	144	230	236	235	247	306	306	202	202	234	238
99rbl.156	135	137	76	76	144	144	226	230	245	245	298	302	180	202	226	238
99rbl.157	137	137	76	78	144	188	230	242	235	249	300	308	196	200	218	234
99rbl.158	135	137	76	78	144	176	224	240	241	245	294	298	200	200	236	238
99rbl.164	135	137	76	76	144	168	220	222	239	245	300	302	202	212	220	230
99rbl.166	135	137	76	76	144	150	222	230	247	249	296	302	202	206	220	234
99rbl.167	137	137	76	76	144	156	222	230	247	249	298	300	202	204	212	244
99rbl.168	137	137	76	78	156	180	222	242	245	247	302	312	202	204	222	238
99rbl.170	137	137	76	76	144	156	232	232	245	249	294	308	202	204	234	236
99rbl.172	137	137	74	76	156	156	230	230	245	251	298	300	202	206	216	234
99rbl.174	137	137	76	76	152	156	232	246	239	241	296	298	196	204	236	236
99rbl.177	137	137	76	76	144	156	226	226	245	247	304	308	204	204	230	236
99rbl.180	137	137	76	76	144	156	222	222	247	247	294	302	160	202	234	240
99rbl.182	137	137	76	76	156	156	222	244	237	241	300	304	200	204	234	236
99rbl.190	135	137	76	76	144	158	226	234	249	251	294	310	200	202	220	252
99rbl.191	137	137	76	76	144	144	226	232	239	253	284	302	196	202	238	248
99rbl.194	135	137	76	76	156	158	232	236	245	245	298	302	200	202	218	236
99rbl.195	135	137	76	76	144	156	222	224	239	239	292	302	196	202	192	222
99rbl.196	137	137	76	76	144	144	230	230	247	251	294	296	200	206	220	226
99rbl.207	135	137	76	76	144	158	222	224	247	249	292	302	198	198	220	236
99rbl.208	137	137	76	76	144	156	222	222	235	239	302	302	202	202	236	238
99rbl.209	137	137	76	76	144	144	226	238	237	249	294	302	160	200	194	226
99rbl.215	135	137	76	76	144	156	222	226	249	249	300	302	196	198	234	236
99rbl.219	137	137	76	76	144	144	224	234	239	239	292	298	200	200	226	226
99rbl.220	135	137	76	76	144	204	230	248	235	237	302	302	200	202	248	252
99rbl.222	135	135	76	76	144	158	224	230	247	249	298	298	196	200	244	250
99rbl.223	135	137	76	76	158	184	224	230	247	247	292	294	196	196	236	236

99rbl.224	135	137	76	76	144	152	222	230	249	251	296	302	196	206	212	236
99rbl.225	137	137	76	76	144	144	222	230	241	247	296	300	198	200	238	238
99rbl.227	137	137	76	76	144	152	224	230	237	245	294	300	198	202	214	238
99rbl.229	137	137	76	76	144	188	220	230	247	247	296	296	196	200	250	250
99rbl.230	135	135	76	76	144	156	214	226	239	251	294	300	200	210	192	238
99rbl.231	137	137	76	78	156	156	224	240	239	241	298	304	202	202	236	238
99rbl.233	137	137	76	76	156	156	230	238	235	245	298	302	196	204	222	230
99rbl.234	137	137	76	76	144	182	228	230	247	255	294	300	196	200	226	236
99rbl.239	137	137	76	76	152	152	222	230	247	251	296	300	202	204	218	220
99rbl.242	137	137	76	76	156	162	224	230	239	249	296	300	202	210	250	252
99rbl.243	137	137	76	76	144	156	230	230	247	251	298	302	198	204	212	220
99rbl.246	137	137	76	76	144	144	224	226	245	253	298	302	160	202	250	252
99rbl.248	135	137	76	76	144	144	224	236	247	247	304	306	202	208	238	238
99rbl.249	135	137	76	76	144	162	222	222	237	245	296	302	202	208	192	232
99rbl.256	137	137	76	76	144	156	224	230	235	245	298	302	202	202	230	236
99rbl.258	135	137	76	76	144	176	222	232	245	245	292	302	196	202	220	238
99rbl.259	137	137	76	76	144	180	224	230	235	239	300	306	202	202	220	236
99rbl.263	137	137	76	76	152	156	222	230	245	249	296	312	180	204	220	220
99rbl.267	137	137	76	76	144	156	228	230	239	245	296	300	196	200	220	228
99rbl.268	135	135	76	76	152	180	222	230	239	245	302	304	196	196	216	228
99rbl.274	137	137	76	76	144	144	230	236	245	249	294	298	198	200	234	236
99rbl.278	137	137	76	76	156	158	226	228	241	249	300	306	202	202	212	234
99rbl.281	137	137	76	76	144	144	226	228	249	249	302	302	202	206	236	236
99rbl.287	135	137	76	76	144	144	224	234	239	249	300	300	202	204	192	234
99rbl.289	135	137	76	76	144	162	212	212	235	245	302	302	198	200	192	220
99rbl.290	137	137	76	76	144	144	232	244	245	247	300	302	160	204	236	240
99rbl.292	137	137	76	76	144	162	220	232	245	247	294	310	200	206	212	260
99rbl.294	135	137	76	76	144	144	220	242	241	247	300	302	196	202	220	234
99rbl.295	137	137	76	76	144	144	238	248	249	251	298	304	202	204	234	236
99rbl.298	135	137	76	76	144	168	222	230	239	251	300	300	202	202	192	220
00rbl.001	135	137	76	76	144	158	224	224	251	251	298	300	160	196	226	238
00rbl.002	137	137	76	76	144	158	220	248	243	249	298	300	202	202	214	238
00rbl.005	137	137	76	76	144	160	222	230	245	249	304	306	200	200	238	248
00rbl.006	137	137	76	76	144	152	224	232	247	249	300	306	160	198	228	242
00rbl.007	137	137	76	78	144	144	230	230	245	249	300	306	202	210	234	236
00rbl.009	137	137	76	78	144	156	222	224	237	245	296	306	202	210	220	236
00rbl.010	137	137	76	76	144	144	224	232	247	247	302	302	204	204	214	244

00rbl.011	137	137	76	76	144	144	224	224	253	253	296	300	200	204	230	238
00rbl.012	137	137	76	76	140	144	222	244	237	251	302	306	202	210	192	224
00rbl.016	137	137	76	76	144	144	224	232	239	251	270	298	196	204	214	234
00rbl.022	137	137	76	76	144	144	226	232	243	245	296	300	202	202	214	234
00rbl.023	137	137	76	76	154	156	224	226	237	247	270	296	198	198	234	238
00rbl.025	137	137	76	76	144	158	226	226	249	249	296	300	198	198	222	252
00rbl.028	135	137	76	76	144	144	226	240	249	249	296	308	202	206	228	240
00rbl.040	137	137	76	76	144	144	224	240	239	247	302	304	200	202	218	240
00rbl.043	137	137	76	76	144	144	226	226	235	245	296	302	196	202	222	242
00rbl.048	135	137	76	76	176	188	230	234	235	245	298	304	202	202	220	234
00rbl.064	137	137	76	76	156	158	220	230	237	247	304	306	202	210	218	250
00rbl.065	137	137	76	76	144	156	222	232	239	245	296	296	160	200	218	232
00rbl.066	137	137	76	76	154	156	220	228	251	251	298	304	160	200	234	270
00rbl.069	137	137	76	76	144	144	224	230	237	239	270	302	202	202	202	232
00rbl.070	137	137	76	76	144	156	222	230	245	247	300	302	160	202	234	236
00rbl.073	137	137	76	76	144	144	230	230	247	257	296	296	200	202	236	236
00rbl.075	137	137	76	76	156	156	224	230	239	251	292	296	200	202	220	220
00rbl.077	137	137	76	76	144	152	222	228	247	247	298	302	200	202	218	234
00rbl.082	137	137	76	76	144	156	230	252	235	251	298	300	198	202	226	236
00rbl.085	137	137	76	76	144	158	224	230	241	247	294	300	202	202	222	230
00rbl.089	137	137	76	76	144	144	230	230	237	245	296	298	196	202	236	238
00rbl.096	137	137	76	78	144	156	224	236	241	253	298	298	196	202	216	234
00rbl.097	137	137	76	76	144	144	230	236	239	245	298	300	196	202	236	240
00rbl.099	137	137	76	76	144	156	228	248	251	251	296	302	200	208	216	218
00rbl.104	137	137	76	76	152	156	220	224	237	245	298	302	200	202	220	236
00rbl.107	137	137	76	76	144	156	224	244	249	249	298	300	200	200	234	246
00rbl.108	137	137	76	76	144	146	224	236	247	249	302	302	196	202	230	238
00rbl.111	137	137	76	76	144	144	226	230	239	239	304	306	198	200	226	248
00rbl.118	137	137	74	76	144	144	224	232	247	249	294	298	198	198	226	238
00rbl.120	137	137	76	78	144	144	222	226	235	247	296	304	202	202	220	236
00rbl.122	137	137	76	76	144	154	232	240	239	251	298	304	196	200	192	248
00rbl.123	137	137	76	76	144	144	224	234	245	245	294	294	198	202	220	240
00rbl.124	137	137	76	76	144	144	262	264	235	247	298	298	200	202	226	238
00rbl.126	135	137	76	78	140	152	222	222	253	253	298	302	202	206	216	236
00rbl.135	137	137	76	76	152	164	232	232	243	249	296	302	196	208	220	226
00rbl.137	135	137	76	76	156	160	232	240	247	253	298	318	200	206	222	246
00rbl.154	135	137	76	76	162	162	226	232	235	247	296	296	202	204	218	220

00rbl.155	135	137	76	76	144	152	230	248	245	249	302	304	196	204	226	252
00rbl.158	137	137	76	78	144	144	232	236	247	251	304	304	202	204	232	234
00rbl.160	137	137	76	76	144	156	228	232	249	249	284	298	196	200	228	238
00rbl.163	137	137	76	76	144	156	232	238	249	257	294	310	200	202	240	254
00rbl.164	137	137	76	78	144	156	220	230	239	249	300	302	200	202	222	226
00rbl.169	137	137	76	78	144	156	222	226	245	245	296	306	200	200	234	236
00rbl.176	135	137	76	78	144	156	222	236	239	241	296	296	200	202	234	234
00rbl.178	135	137	76	76	144	188	222	238	239	255	288	296	200	206	228	248
00rbl.179	137	137	76	76	144	144	222	222	247	249	294	304	196	202	234	234
00rbl.180	137	137	76	76	144	144	230	256	245	247	298	300	160	202	232	254
00rbl.183	135	137	76	76	144	144	228	248	239	247	298	302	200	202	218	220
00rbl.186	137	137	76	76	144	188	232	236	239	247	302	306	198	200	210	236
00rbl.191	137	137	76	76	144	156	234	240	247	247	306	308	198	202	220	226
00rbl.194	137	137	76	76	178	190	234	242	247	247	296	298	200	200	224	236
00rbl.196	135	137	76	76	156	156	232	240	251	251	290	300	200	200	212	232
00rbl.198	135	135	76	76	144	144	240	240	241	241	296	300	202	204	238	244
00rbl.202	137	137	76	76	144	156	232	234	237	239	302	302	196	212	232	236
00rbl.203	137	137	76	76	144	144	238	240	247	247	296	300	202	202	222	234
00rbl.204	137	137	76	76	144	158	230	230	247	247	302	306	202	202	234	234
00rbl.206	135	137	76	76	156	162	224	232	247	247	300	300	198	200	242	244
00rbl.212	137	137	76	76	144	144	232	242	237	251	300	328	202	210	236	236
00rbl.217	135	137	76	76	144	144	230	230	243	249	298	304	200	200	214	236
00rbl.223	137	137	74	76	144	156	214	226	247	247	298	298	196	202	228	244
00rbl.225	137	137	76	76	144	144	224	230	247	247	302	304	202	202	232	234
00rbl.227	137	137	76	76	156	156	236	248	247	247	296	312	180	200	220	220
00rbl.228	137	137	76	76	144	162	232	248	239	239	300	300	200	202	242	250
00rbl.232	137	137	76	78	144	156	232	240	245	245	298	304	160	202	212	212
00rbl.236	137	137	76	76	144	144	230	242	247	247	294	302	202	202	256	270
00rbl.239	137	137	76	76	144	144	232	240	237	251	294	304	202	202	232	252
00rbl.240	137	137	76	78	144	152	228	266	241	245	296	296	204	206	220	240
00rbl.242	137	137	76	76	144	144	228	252	241	247	294	296	200	202	232	234
00rbl.244	137	137	76	76	156	156	236	266	243	245	294	296	200	206	242	252
00rbl.245	137	137	76	76	144	158	228	230	251	251	294	294	200	200	236	236
00rbl.249	137	137	76	76	144	144	224	240	249	249	294	302	200	200	234	270
00rbl.257	137	137	76	76	144	144	224	242	247	249	294	296	200	202	232	232
00rbl.258	137	137	76	76	144	144	222	226	249	249	0	0	200	202	238	254
00rbl.263	137	137	76	76	144	144	222	236	237	245	292	304	202	202	226	228

00rbl.267	137	137	76	78	144	144	224	230	237	251	298	300	200	202	192	234
00rbl.268	137	137	76	76	144	144	224	252	249	249	296	300	200	200	220	268
00rbl.271	137	137	76	76	144	144	220	230	247	251	296	302	196	200	234	236
00rbl.273	135	137	74	76	144	144	238	238	243	253	298	302	200	202	194	238
00rbl.277	137	137	76	76	164	188	230	238	239	245	300	302	200	200	216	236
00rbl.278	137	137	76	78	144	156	232	244	235	247	300	308	198	202	238	240
00rbl.282	135	137	76	76	144	144	224	238	239	245	300	302	196	200	220	236
00rbl.284	137	137	76	76	156	164	226	226	247	249	300	306	200	202	234	234
00rbl.288	137	137	76	76	140	164	220	256	239	247	300	304	200	200	234	250
00rbl.290	137	137	76	76	144	152	228	242	245	249	296	296	196	196	220	220
00rbl.294	137	137	76	76	144	158	226	230	241	251	296	300	200	202	194	238
00rbl.295	137	137	76	76	144	144	234	236	245	249	298	300	200	202	220	238
00rbl.299	137	137	76	76	144	164	220	222	239	251	296	302	196	202	234	238
00rbl.301	137	137	76	76	144	144	224	236	253	253	296	304	200	202	202	226
00rbl.302	137	137	76	76	144	144	230	232	239	247	294	302	202	202	236	236
00rbl.304	137	137	76	76	144	162	222	224	241	249	296	304	200	202	192	224
00rbl.306	137	137	76	76	144	144	228	230	247	247	296	308	202	202	216	234
00rbl.307	137	137	76	76	144	144	226	230	247	247	298	310	200	202	214	234
00rbl.309	137	137	76	76	144	156	222	240	249	249	296	302	204	204	230	242
00rbl.312	137	137	76	76	144	144	224	230	239	247	300	302	200	202	216	234
00rbl.320	137	137	76	76	144	144	224	230	245	249	298	302	200	202	220	220
00rbl.321	137	137	76	78	144	156	232	234	237	239	298	300	160	212	220	224
00rbl.324	137	137	76	76	144	144	234	234	239	239	302	314	200	200	214	234
00rbl.325	137	137	76	76	144	144	230	232	239	249	300	300	200	202	0	0
00rbl.326	137	137	76	76	144	188	224	224	245	249	294	298	196	200	218	220
00rbl.327	137	137	76	76	144	144	232	250	245	247	294	294	200	202	234	248
00rbl.328	137	137	76	76	144	158	226	230	235	239	296	298	196	196	222	234
00rbl.335	137	137	76	78	144	156	220	222	247	247	298	298	200	202	250	252
00rbl.336	137	137	76	76	144	182	232	250	247	249	294	294	202	204	234	248
00rbl.337	137	137	76	78	156	158	222	232	247	249	296	302	196	200	220	260
00rbl.344	137	137	76	76	144	144	240	258	241	247	298	300	160	200	218	238
00rbl.347	137	137	76	76	144	144	224	228	253	253	296	300	202	204	228	248
00rbl.351	137	137	76	76	144	156	222	230	245	247	304	306	200	202	228	236
00rbl.354	137	137	76	76	144	144	222	226	239	247	296	298	200	202	234	236
00rbl.355	137	137	76	76	144	144	222	230	239	245	296	302	202	204	228	234
00rbl.357	137	137	76	76	144	144	236	238	243	249	300	300	202	204	232	236
00rbl.359	137	137	76	76	144	144	222	226	245	251	294	304	202	202	244	260

00rbl.364	137	137	76	76	144	144	228	232	249	249	294	294	200	202	0	0
00rbl.372	137	137	76	76	144	144	228	232	239	247	296	300	200	202	0	0
01rbl.002	137	137	76	76	144	144	242	244	245	245	302	308	200	202	234	264
01rbl.003	137	137	76	76	154	158	240	240	241	245	298	304	196	204	222	236
01rbl.004	137	137	76	76	144	156	226	244	247	249	300	330	196	202	222	254
01rbl.005	137	137	76	76	172	188	226	242	245	255	296	304	202	204	234	236
01rbl.009	137	137	76	78	154	156	228	244	241	245	298	304	160	204	220	222
01rbl.010	137	137	76	76	144	144	232	242	247	247	298	304	204	204	236	256
01rbl.013	137	137	76	78	144	144	228	244	241	245	298	298	160	204	192	222
01rbl.015	137	137	76	76	144	154	240	244	247	247	298	304	204	204	236	238
01rbl.017	137	137	76	76	158	164	234	244	247	247	308	332	202	204	236	240
01rbl.021	137	137	76	76	144	158	224	232	241	251	296	308	198	202	220	242
01rbl.022	137	137	76	76	144	144	232	244	247	247	298	304	204	204	222	256
01rbl.023	135	137	76	76	154	162	234	238	249	249	304	306	202	204	222	252
01rbl.024	137	137	76	78	144	166	234	244	247	247	296	302	200	202	236	240
01rbl.025	137	137	76	76	144	144	242	250	247	247	298	332	204	204	220	222
01rbl.026	137	137	76	76	158	162	214	248	247	247	296	304	200	202	236	250
01rbl.027	137	137	76	76	144	144	224	224	251	251	294	300	202	204	204	250
01rbl.030	137	137	76	76	144	156	230	240	251	253	296	298	198	204	234	236
01rbl.031	137	137	76	76	144	144	224	234	241	245	294	298	200	200	222	228
01rbl.032	137	137	76	76	144	144	224	230	249	251	298	300	194	206	214	238
01rbl.035	137	137	76	76	144	144	224	238	241	247	298	298	200	202	0	0
01rbl.037	137	137	76	76	144	144	224	230	247	247	300	302	202	202	0	0
01rbl.038	137	137	76	76	144	144	230	230	237	253	304	306	196	202	234	240
01rbl.042	137	137	76	76	144	144	222	224	247	247	298	302	198	202	214	234
01rbl.044	137	137	76	76	144	144	230	248	247	247	302	304	206	208	226	234
01rbl.046	137	137	76	76	144	144	238	266	247	247	296	302	202	202	238	238
01rbl.049	137	137	76	76	144	158	222	226	251	253	298	302	200	202	220	220
01rbl.050	137	137	76	76	144	144	228	238	245	255	300	318	196	200	218	220
01rbl.051	137	137	76	76	144	158	222	224	247	249	298	306	200	202	192	212
01rbl.053	137	137	76	76	144	168	230	234	249	253	296	300	200	206	240	240
01rbl.054	135	137	76	76	156	156	224	240	249	251	294	296	200	200	226	250
01rbl.057	137	137	74	76	144	156	224	230	249	251	298	300	200	200	220	240
01rbl.060	137	137	76	76	144	162	224	224	249	249	296	300	202	204	238	240
01rbl.061	135	137	76	76	144	156	226	230	239	247	296	306	198	210	214	238
01rbl.063	137	137	76	76	144	144	230	238	241	249	302	302	192	202	224	234
01rbl.067	137	137	76	76	144	144	214	240	241	249	300	302	196	204	250	252

01rbl.069	137	137	72	76	144	156	222	232	239	247	294	298	198	200	220	238
01rbl.073	137	137	76	76	144	156	224	230	239	247	296	330	196	198	218	238
01rbl.074	137	137	76	76	144	160	236	242	241	241	300	308	196	206	218	238
01rbl.078	137	137	76	76	154	158	232	238	247	247	294	296	200	202	218	234
01rbl.079	137	137	76	76	144	158	232	234	235	239	298	304	200	202	228	236
01rbl.083	137	137	76	76	144	144	226	248	237	249	290	300	160	208	242	254
01rbl.086	137	137	76	76	144	144	222	224	247	247	292	296	160	200	0	0
01rbl.087	137	137	76	76	144	158	230	240	241	247	296	296	200	202	238	238
01rbl.088	137	137	76	76	144	158	222	224	247	247	304	312	200	204	218	220
01rbl.090	137	137	76	76	144	144	222	238	247	247	300	310	202	202	192	220
01rbl.091	137	137	76	78	144	156	222	224	247	247	298	306	202	206	220	232
01rbl.093	137	137	76	76	144	144	238	240	247	247	300	320	204	204	238	246
01rbl.094	137	137	76	76	144	156	224	234	241	249	296	312	202	202	230	240
01rbl.096	137	137	76	76	156	172	226	232	249	251	298	304	204	204	224	238
01rbl.099	137	137	76	76	144	144	222	240	247	247	290	306	198	204	192	240
01rbl.104	135	137	76	76	144	188	232	236	239	247	298	298	200	200	192	240
01rbl.105	137	137	76	76	144	176	240	240	241	251	296	300	200	208	212	220
01rbl.107	137	137	76	76	144	176	230	230	241	247	298	302	202	204	232	234
01rbl.108	137	137	76	76	144	144	224	240	241	247	304	306	202	204	234	238
01rbl.109	137	137	76	76	144	154	238	244	245	247	296	330	202	204	220	234
01rbl.110	137	137	76	76	150	158	234	238	245	251	298	300	196	208	212	242
01rbl.113	137	137	76	76	144	156	224	260	245	247	292	300	200	202	238	248
01rbl.114	135	137	76	76	152	158	230	232	239	241	304	310	196	196	226	240
01rbl.118	137	137	76	76	156	160	228	248	241	245	302	302	202	208	238	250
01rbl.119	135	137	76	76	144	158	226	240	247	247	306	320	196	204	226	238
01rbl.121	137	137	76	76	144	144	226	228	249	255	292	310	196	204	220	240
01rbl.122	135	137	76	76	156	168	224	234	239	245	300	302	202	204	222	236
01rbl.124	135	137	76	78	144	160	234	244	239	245	294	296	196	208	232	270
01rbl.125	135	137	76	76	144	144	240	264	247	251	298	304	200	202	192	200
01rbl.126	137	137	76	76	144	184	224	224	249	251	294	298	202	204	234	252
01rbl.128	135	137	76	76	144	156	224	230	239	241	298	298	200	200	212	220
01rbl.133	135	137	76	76	144	144	224	230	235	251	296	300	206	208	220	234
01rbl.135	137	137	76	76	144	182	246	248	239	241	300	300	198	204	216	218
01rbl.139	137	137	76	76	152	158	230	230	235	237	296	302	200	204	224	224
01rbl.140	137	137	76	78	176	182	222	248	245	247	298	304	160	202	212	240
01rbl.142	137	137	76	76	144	144	224	224	245	247	300	302	200	202	218	250
01rbl.143	137	137	76	76	144	144	224	224	239	247	300	318	180	212	220	236

01rbl.144	137	137	76	76	182	184	246	248	245	245	298	304	160	200	216	252
01rbl.149	137	137	76	76	144	162	224	238	245	245	302	304	204	206	216	252
01rbl.150	137	137	76	78	144	144	220	236	241	245	298	298	202	204	238	240
01rbl.153	137	137	76	78	156	176	224	224	239	243	298	302	202	204	236	240
01rbl.158	137	137	76	76	144	156	224	248	237	251	296	304	200	202	220	224
01rbl.159	135	137	76	76	168	190	244	256	245	249	298	300	202	206	218	242
01rbl.160	137	137	76	76	154	164	222	226	247	247	296	302	196	202	230	236
01rbl.161	137	137	76	76	144	164	224	242	245	249	296	304	200	208	240	254
01rbl.162	137	137	76	76	144	156	222	238	249	249	298	300	200	204	226	234
01rbl.163	137	137	76	76	144	156	222	242	255	255	300	304	160	200	220	252
01rbl.164	137	137	76	76	144	156	222	228	239	249	302	302	202	204	222	242
01rbl.165	137	137	76	76	144	144	220	224	239	241	296	308	160	200	212	230
01rbl.167	135	137	76	76	156	162	222	246	245	247	298	306	200	206	238	238
01rbl.168	137	137	76	76	144	164	224	242	247	249	296	304	206	206	190	240
01rbl.169	135	137	76	76	152	152	230	238	239	247	294	302	160	198	220	240
01rbl.170	137	137	76	76	144	156	246	250	239	249	298	302	200	202	222	222
01rbl.171	135	137	76	76	158	180	222	238	239	241	302	302	198	200	220	236
01rbl.172	135	137	76	76	156	180	212	238	241	245	300	302	198	200	220	222
01rbl.174	137	137	76	76	144	166	224	242	241	249	296	296	160	204	212	242
01rbl.175	137	137	76	76	156	156	230	238	245	247	294	298	160	202	222	238
01rbl.179	137	137	76	76	144	144	222	242	249	249	296	304	200	202	238	250
01rbl.180	137	137	76	76	144	152	230	230	239	255	298	302	196	198	220	260
01rbl.185	135	137	72	76	144	160	224	238	245	251	296	300	200	202	238	240
01rbl.188	135	137	72	76	156	156	222	226	239	241	300	302	196	202	216	232
01rbl.189	137	137	76	78	144	156	234	254	237	239	300	302	204	204	222	236
01rbl.191	137	137	76	76	144	166	222	240	235	249	308	312	204	208	214	236
01rbl.193	135	137	76	76	156	168	224	230	239	241	300	300	200	208	220	236
01rbl.194	135	137	76	76	156	158	222	234	245	249	294	304	196	204	240	242
01rbl.195	137	137	76	76	144	152	226	232	247	247	298	298	202	208	230	244
01rbl.196	135	137	76	76	144	144	230	230	235	245	292	330	204	204	236	238
01rbl.197	137	137	76	76	156	180	224	232	247	249	296	334	180	200	212	238
01rbl.198	137	137	76	76	158	184	228	230	239	243	300	302	198	204	212	226
01rbl.207	137	137	76	76	170	170	230	238	241	251	296	302	204	206	222	238
01rbl.209	137	137	76	76	144	156	226	230	239	249	298	298	202	210	220	250
01rbl.211	137	137	76	76	144	144	238	248	249	251	298	298	180	204	220	222
01rbl.212	135	137	76	76	144	152	222	222	237	249	298	304	196	206	220	226
01rbl.213	137	137	76	76	156	156	224	234	245	251	292	302	200	202	236	238

01rbl.214	135	137	76	76	144	156	222	230	249	249	298	300	204	204	212	254
01rbl.215	137	137	76	76	144	152	222	224	245	249	286	296	200	202	236	238
01rbl.216	137	137	76	76	152	160	222	230	247	247	298	298	198	202	238	238
01rbl.217	137	137	76	76	144	174	236	248	245	247	296	308	160	202	192	222
01rbl.218	135	137	76	76	156	156	224	232	245	247	296	298	200	206	234	242
01rbl.220	137	137	76	76	156	164	232	242	247	251	298	302	202	204	212	254
01rbl.221	135	137	76	76	144	156	248	248	239	249	298	304	202	204	212	222
01rbl.223	137	137	76	76	144	144	222	232	247	249	294	304	200	202	220	220
01rbl.224	137	137	76	76	144	154	224	224	239	247	302	304	198	198	228	232
01rbl.225	137	137	74	76	152	158	212	244	237	237	304	306	160	204	216	228
01rbl.232	135	135	76	76	144	156	226	242	247	251	304	304	160	204	228	248
02rbl.002	137	137	76	76	144	158	222	244	239	245	294	300	196	202	234	236
02rbl.003	137	137	76	76	144	144	224	236	245	245	294	298	200	202	220	220
02rbl.004	137	137	76	76	144	158	222	226	245	251	292	306	200	202	226	248
02rbl.008	137	137	76	76	162	162	230	234	237	251	300	302	198	202	242	250
02rbl.011	137	137	76	76	144	152	228	240	237	245	294	302	196	198	220	230
02rbl.012	137	137	76	76	144	144	222	232	247	249	298	300	196	198	220	226
02rbl.014	137	137	76	76	156	156	238	248	251	251	294	314	200	200	216	236
02rbl.016	137	137	76	76	156	172	232	240	239	247	298	300	160	202	228	236
02rbl.018	137	137	76	76	144	156	238	242	239	249	294	296	196	208	218	232
02rbl.019	137	137	76	76	144	144	222	232	247	247	298	320	196	198	220	236
02rbl.020	137	137	76	76	144	154	222	224	239	247	270	298	198	200	222	236
02rbl.024	137	137	76	76	144	162	214	232	247	251	294	300	160	200	234	250
02rbl.025	135	137	76	76	144	154	228	234	245	245	292	302	196	198	216	220
02rbl.030	135	137	76	76	144	174	230	242	247	247	292	296	200	202	226	226
02rbl.032	135	137	76	78	144	158	224	224	235	247	300	302	196	200	228	236
02rbl.033	137	137	76	76	144	164	228	230	239	251	298	300	196	200	218	244
02rbl.038	137	137	76	78	144	144	222	236	245	245	296	304	200	210	228	228
02rbl.039	137	137	76	76	144	156	230	248	239	245	296	302	202	204	242	250
02rbl.043	137	137	76	76	144	156	228	230	239	239	306	318	198	200	234	238
02rbl.048	135	137	76	76	144	158	238	238	247	247	302	302	160	204	234	238
02rbl.053	137	137	76	76	144	172	230	236	245	249	300	306	196	210	228	234
02rbl.061	137	137	76	76	144	156	222	242	239	249	302	302	200	200	224	242
02rbl.062	137	137	76	76	144	164	220	224	241	247	298	308	200	204	220	236
02rbl.067	135	137	76	76	144	176	236	242	237	247	292	302	196	196	214	234
02rbl.068	137	137	76	76	144	166	222	226	245	245	294	300	196	204	218	234
02rbl.071	137	137	76	78	144	156	230	240	249	251	296	300	198	202	192	234

02rbl.072	135	137	76	76	144	144	224	242	245	245	290	300	196	204	218	234
02rbl.078	137	137	76	76	144	160	220	230	247	247	302	302	198	198	240	252
02rbl.083	137	137	76	76	158	170	220	224	235	245	302	306	192	200	218	236
02rbl.086	137	137	74	76	158	174	228	240	247	247	298	306	194	202	216	220
02rbl.088	137	137	76	76	144	144	222	222	235	239	300	312	198	204	222	234
02rbl.093	135	135	76	78	144	144	226	230	239	247	292	306	196	210	214	238
02rbl.095	137	137	76	76	144	144	228	230	247	253	294	304	198	200	194	218
02rbl.100	137	137	76	78	144	144	224	228	247	249	294	302	198	200	214	236
02rbl.102	137	137	76	76	144	144	222	222	249	251	300	300	196	202	234	236
02rbl.107	135	137	76	76	144	144	224	236	235	247	294	294	200	202	192	248
02rbl.108	137	137	76	76	144	164	226	232	245	249	300	304	200	202	224	244
02rbl.114	137	137	76	76	144	144	224	224	245	249	294	298	198	202	222	222
02rbl.117	137	137	76	76	144	156	228	230	235	251	300	302	196	200	238	240
02rbl.121	135	137	76	76	156	164	232	232	249	251	294	300	196	202	218	236
02rbl.126	137	137	76	76	144	154	220	230	239	253	296	300	202	202	192	236
02rbl.128	137	137	76	76	144	144	230	238	245	249	302	302	200	204	230	240
02rbl.132	137	137	76	76	144	158	222	230	245	249	292	298	196	202	234	252
02rbl.133	135	137	76	76	144	144	224	244	247	249	296	306	194	200	220	226
02rbl.137	137	137	76	76	160	174	238	238	247	247	296	302	194	204	220	234
02rbl.140	137	137	76	76	144	144	222	230	249	251	292	310	196	200	234	254
02rbl.142	137	137	76	76	156	176	240	244	245	249	298	302	200	200	220	236
02rbl.146	137	137	76	76	156	172	222	230	247	251	296	298	196	202	234	238
02rbl.149	137	137	76	76	144	154	222	240	239	251	292	304	200	202	228	236
02rbl.152	137	137	74	76	140	144	224	242	239	245	288	306	200	202	232	236
02rbl.153	137	137	76	76	144	156	224	248	247	249	296	304	198	200	238	250
02rbl.157	137	137	72	76	144	152	222	226	245	249	300	302	196	198	220	250
02rbl.159	137	137	76	78	144	158	224	230	251	253	298	302	160	202	230	234
02rbl.161	135	137	76	76	144	156	222	226	239	247	294	298	200	200	214	234
02rbl.163	137	137	76	76	144	144	222	230	239	245	306	330	200	202	230	236
02rbl.164	137	137	76	78	154	176	232	238	245	249	292	298	196	200	236	238
02rbl.165	137	137	76	76	144	180	226	240	239	247	294	296	198	202	192	212
02rbl.172	137	137	76	76	156	158	232	234	245	249	292	302	196	196	226	234
02rbl.173	137	137	76	76	144	158	222	230	245	247	298	300	200	200	230	234
02rbl.180	137	137	76	76	188	188	230	230	245	245	288	298	200	206	218	238
02rbl.184	135	137	76	76	144	156	230	230	247	249	296	300	200	202	212	220
02rbl.185	137	137	76	76	144	156	226	260	247	249	292	292	196	198	234	238
02rbl.186	135	137	76	76	152	156	224	230	237	247	294	302	202	204	220	238

02rbl.188	137	137	76	76	144	144	224	238	239	247	294	296	202	206	224	236
02rbl.192	135	137	76	76	156	158	212	248	239	239	300	306	196	202	220	238
02rbl.197	137	137	76	76	144	156	226	236	247	249	298	298	200	202	220	234
02rbl.201	137	137	76	78	144	156	230	244	239	245	296	304	202	210	230	242
02rbl.210	137	137	76	76	144	164	240	242	239	247	302	302	160	200	226	236
02rbl.212	135	137	76	76	144	164	240	248	247	251	314	330	200	206	238	254
02rbl.214	137	137	76	76	144	156	220	262	239	247	292	304	202	202	246	250
02rbl.215	137	137	76	76	156	156	236	248	247	251	302	310	200	200	240	242
02rbl.216	137	137	76	76	144	144	226	228	237	245	300	302	196	196	220	230
02rbl.217	137	137	74	76	152	156	214	240	239	245	298	300	198	202	234	240
02rbl.223	137	137	76	76	144	156	224	240	245	249	294	312	200	208	232	254
02rbl.226	137	137	76	76	144	188	226	232	241	251	296	306	200	202	228	240
02rbl.229	137	137	76	76	144	144	234	234	235	243	300	302	196	200	192	234
02rbl.230	135	137	74	76	144	156	232	246	247	251	306	310	202	202	234	234
02rbl.235	135	137	76	76	144	156	224	236	249	249	300	302	196	200	238	254
02rbl.236	135	137	74	76	144	158	220	242	241	249	300	302	202	202	220	240
02rbl.237	135	137	74	76	144	184	230	230	237	239	300	302	202	204	230	234
02rbl.242	137	137	76	76	144	162	214	232	247	247	298	302	160	198	212	258
02rbl.243	137	137	76	76	156	158	214	220	241	247	302	302	160	200	212	216
02rbl.246	137	137	76	76	144	156	224	238	247	249	292	298	200	210	232	236
02rbl.252	135	137	76	76	144	152	234	234	239	255	298	300	196	204	234	260
02rbl.253	135	137	76	76	152	162	212	232	241	249	284	296	200	202	228	254
02rbl.254	137	137	76	76	144	158	214	224	247	247	294	298	196	204	212	220
02rbl.255	135	137	76	76	156	158	224	232	239	253	296	298	202	206	240	250
02rbl.260	135	137	74	76	144	144	222	224	239	239	296	302	202	204	230	234
02rbl.261	135	135	76	78	144	164	240	240	245	247	300	314	194	202	192	254
02rbl.264	137	137	76	76	144	146	224	224	237	245	296	306	196	196	218	240
02rbl.265	137	137	72	76	144	156	228	238	241	241	300	302	200	202	234	238
02rbl.267	137	137	76	76	144	156	224	238	237	245	294	304	200	202	230	238
02rbl.268	137	137	76	76	144	152	224	248	239	247	286	296	200	200	218	240
02rbl.271	137	137	76	76	144	144	238	240	241	245	298	302	200	204	226	238
02rbl.274	137	137	76	76	154	162	222	230	247	249	298	302	198	204	232	238
02rbl.277	137	137	76	76	144	144	230	230	241	245	302	318	200	202	216	246
02rbl.278	137	137	76	76	144	144	230	230	239	251	296	300	160	204	226	232
02rbl.279	137	137	76	76	156	162	230	248	239	247	300	302	200	206	192	236
02rbl.291	137	137	76	76	144	162	224	246	239	245	298	302	202	206	234	238
02rbl.295	137	137	76	76	144	144	224	230	235	251	296	300	206	208	194	234

02rbl.300	137	137	76	76	156	156	230	230	239	245	300	302	196	206	220	234
02rbl.304	135	137	76	76	144	164	224	236	245	247	300	302	202	204	240	252
02rbl.306	137	137	76	76	144	156	224	230	235	239	300	304	204	204	218	226
02rbl.307	135	137	76	76	152	156	224	240	239	245	296	306	202	206	216	220
02rbl.311	135	137	76	76	156	162	234	246	245	247	292	296	200	202	216	238
02rbl.319	137	137	72	76	144	174	222	222	239	245	296	304	196	208	192	236
02rbl.323	137	137	76	76	144	188	230	238	247	251	292	302	160	198	238	240
02rbl.327	135	137	72	78	156	156	224	240	245	245	296	300	200	202	232	234
02rbl.328	137	137	76	76	144	156	224	224	239	245	270	296	198	200	192	236
02rbl.330	135	137	76	78	144	156	230	238	235	245	302	302	200	202	238	266
02rbl.335	137	137	76	76	144	144	224	232	239	247	298	302	196	200	194	238
02rbl.337	137	137	76	76	144	144	230	238	237	239	298	306	202	206	238	248
02rbl.338	137	137	76	76	144	156	222	224	239	245	296	296	200	202	232	232
02rbl.339	137	137	76	76	144	156	230	240	237	239	294	300	202	206	220	234
02rbl.340	135	137	76	76	164	176	230	230	235	239	294	300	200	202	192	218
02rbl.341	135	137	76	76	144	144	228	230	245	249	294	300	200	200	234	250
02rbl.345	137	137	76	76	144	156	220	240	249	255	296	302	160	200	230	254
02rbl.350	137	137	76	76	156	162	234	240	249	249	302	310	200	200	214	220
02rbl.351	135	135	76	76	144	144	238	242	239	241	298	302	196	200	220	236
02rbl.353	137	137	76	76	144	156	222	230	239	247	292	294	202	204	212	220
03rbl.001	137	137	76	76	144	162	214	224	249	249	294	298	200	200	236	236
03rbl.005	137	137	76	76	144	144	214	226	249	251	304	304	202	202	230	230
03rbl.006	137	137	76	76	154	176	220	224	247	249	298	304	200	202	222	226
03rbl.015	137	137	76	76	144	156	226	230	249	251	294	296	198	204	240	246
03rbl.016	137	137	76	76	144	184	224	240	239	247	300	302	200	202	236	242
03rbl.019	135	137	76	76	156	156	226	230	249	251	300	300	198	202	234	240
03rbl.023	135	137	76	78	144	144	224	234	247	249	298	304	200	208	220	238
03rbl.026	137	137	76	78	144	144	226	230	245	249	300	304	202	204	224	238
03rbl.027	137	137	76	76	144	156	222	222	245	249	304	308	204	204	238	252
03rbl.029	137	137	76	76	144	162	224	238	249	249	298	298	204	204	190	232
03rbl.030	137	137	76	76	144	144	224	224	237	249	302	304	202	202	234	252
03rbl.031	137	137	76	76	144	144	224	230	239	247	300	304	192	202	220	234
03rbl.032	137	137	76	76	156	158	222	222	239	245	296	308	200	202	220	220
03rbl.033	137	137	76	78	144	144	214	238	245	253	302	304	202	206	220	226
03rbl.034	137	137	76	76	152	158	222	230	241	251	300	304	200	202	236	250
03rbl.035	135	137	76	76	144	144	220	236	239	257	298	308	206	212	216	222
03rbl.038	137	137	76	76	158	158	224	230	239	243	296	304	196	202	228	240

03rbl.039	137	137	76	76	144	158	224	224	245	249	302	302	204	204	220	240
03rbl.041	137	137	76	76	152	158	228	228	243	245	296	302	196	202	216	224
03rbl.042	137	137	76	76	144	144	230	264	249	249	296	298	196	206	234	238
03rbl.043	135	137	76	76	144	184	222	230	239	239	296	298	202	202	212	232
03rbl.044	135	137	76	76	144	176	226	246	245	249	298	302	198	204	220	242
03rbl.046	135	137	76	76	162	180	230	230	239	245	300	308	196	200	236	270
03rbl.049	137	137	76	76	156	156	224	226	245	245	296	302	198	200	218	220
03rbl.050	137	137	76	76	156	156	232	232	245	249	294	306	202	202	222	238
03rbl.053	137	137	76	76	144	154	224	230	239	247	294	296	202	204	212	236
03rbl.054	137	137	76	78	144	174	214	238	237	249	298	302	194	198	220	234
03rbl.057	137	137	76	76	144	144	234	236	235	251	274	308	160	198	236	238
03rbl.058	137	137	76	76	144	144	230	238	245	247	298	302	202	206	250	252
03rbl.059	137	137	76	76	144	144	222	234	239	251	302	306	200	200	230	250
03rbl.063	137	137	76	76	144	156	230	234	241	255	296	302	200	202	192	236
03rbl.069	137	137	76	76	150	156	224	224	249	251	302	304	196	200	198	234
03rbl.070	137	137	76	78	144	156	222	224	239	241	298	300	198	204	226	254
03rbl.071	137	137	76	76	164	176	230	236	235	245	292	296	196	210	234	234
03rbl.072	137	137	76	78	144	198	224	236	249	249	290	298	196	206	222	234
03rbl.076	137	137	76	76	144	144	224	230	241	245	296	302	160	202	212	234
03rbl.082	135	137	76	76	156	164	224	230	247	249	298	312	194	200	236	256
03rbl.086	137	137	76	76	144	168	218	238	249	251	294	296	202	204	222	254
03rbl.089	137	137	76	76	156	160	222	238	249	251	298	300	160	202	222	242
03rbl.091	137	137	76	78	144	156	230	230	247	247	296	302	200	202	236	236
03rbl.093	135	137	76	76	144	156	224	246	239	247	302	302	202	204	222	238
03rbl.105	137	137	76	76	156	180	232	248	247	251	296	306	202	210	238	242
03rbl.106	137	137	76	76	158	158	222	248	241	245	296	304	198	202	240	242
03rbl.108	137	137	76	76	144	158	224	232	245	255	296	310	200	204	222	236
03rbl.112	137	137	76	76	144	144	232	248	249	251	296	300	202	212	224	236
03rbl.114	137	137	76	76	144	156	228	230	241	253	296	308	202	206	220	238
03rbl.115	137	137	76	76	156	158	232	238	249	255	296	300	202	206	220	238
03rbl.116	137	137	76	76	180	180	224	232	245	249	296	300	196	202	236	240
03rbl.122	135	137	76	76	152	180	224	232	245	255	296	306	196	202	238	246
03rbl.124	137	137	74	76	154	180	232	232	245	255	300	302	160	202	192	238
03rbl.137	135	137	76	76	156	176	226	236	245	249	296	296	198	198	212	218
03rbl.138	135	137	76	76	144	144	224	244	247	249	298	302	204	204	216	242
03rbl.140	137	137	76	76	144	158	222	250	237	249	294	306	204	206	216	240
03rbl.142	137	137	76	76	144	156	234	250	247	249	296	304	200	206	216	220

03rbl.145	137	137	76	76	144	170	224	234	247	253	300	302	200	202	234	242
03rbl.146	137	137	76	76	144	144	216	242	247	249	298	300	204	212	192	236
03rbl.149	137	137	76	76	156	156	230	242	249	251	300	306	200	204	220	236
03rbl.150	135	137	74	76	144	156	216	228	241	249	304	308	204	206	234	258
03rbl.156	135	137	76	76	144	168	228	238	241	249	288	300	196	202	240	254
03rbl.157	137	137	76	76	156	156	240	244	249	251	300	300	200	210	236	238
03rbl.159	137	137	74	76	144	156	230	230	245	247	292	306	196	202	252	258
03rbl.160	137	137	76	76	144	156	224	224	245	249	298	306	202	206	220	222
03rbl.165	137	137	76	76	144	158	226	238	247	249	298	298	200	204	236	236
03rbl.167	135	137	76	76	144	160	234	238	239	247	292	298	204	206	192	220
03rbl.171	135	137	76	76	158	160	222	236	251	253	298	298	200	204	220	238
03rbl.173	135	137	76	76	144	158	222	230	239	255	298	302	192	200	240	248
03rbl.174	135	137	76	76	144	176	224	230	245	249	302	308	202	204	244	260
03rbl.176	135	135	76	76	144	144	222	230	237	247	298	308	206	208	228	236
03rbl.178	137	137	76	76	158	164	230	240	239	245	302	306	204	204	236	236
03rbl.179	137	137	76	78	152	164	224	230	241	251	304	304	204	206	220	220
03rbl.181	137	137	76	76	156	156	228	232	249	251	294	296	200	200	230	236
03rbl.185	137	137	76	78	144	144	234	240	249	251	296	298	160	200	218	220
03rbl.190	135	137	76	76	154	158	222	230	241	245	300	302	180	204	240	250
03rbl.192	137	137	76	76	144	156	238	240	241	249	302	306	206	208	238	252
03rbl.194	135	137	76	76	158	158	222	256	245	249	298	304	160	206	220	234
03rbl.196	135	137	76	76	144	144	230	240	241	251	300	304	204	206	228	250
03rbl.199	137	137	76	76	144	156	222	240	247	249	292	306	160	204	220	236
03rbl.209	137	137	76	76	156	176	214	222	241	249	300	304	200	206	220	220
03rbl.218	137	137	76	76	156	158	230	230	235	245	300	302	180	196	240	244
03rbl.220	135	137	76	76	144	144	224	232	235	247	298	302	200	202	220	220
03rbl.222	135	137	76	76	144	166	234	234	247	249	298	302	196	200	212	246
03rbl.224	135	137	76	78	144	144	214	234	235	249	296	302	196	202	220	232
03rbl.231	137	137	76	76	144	160	224	234	239	239	296	302	200	202	220	236
03rbl.232	137	137	76	78	164	166	230	232	247	249	296	298	196	200	212	246
03rbl.235	135	137	76	76	144	156	230	240	249	249	296	296	200	202	190	252
03rbl.236	137	137	76	76	144	144	232	238	247	249	296	298	196	200	198	220
03rbl.244	137	137	76	78	156	156	222	240	239	245	292	294	200	204	238	238
03rbl.250	137	137	76	76	144	144	240	242	247	249	296	300	202	204	220	254
03rbl.258	135	137	74	76	144	144	222	222	245	247	300	302	200	202	220	226
03rbl.259	137	137	76	76	144	156	226	236	245	249	294	302	160	202	218	230
03rbl.262	135	137	76	78	144	144	224	240	235	247	298	304	202	204	238	240

03rbl.264	135	137	76	76	144	156	230	238	237	249	296	300	160	202	240	240
03rbl.272	135	137	76	76	144	152	224	238	239	249	302	304	196	202	220	238
03rbl.273	137	137	76	78	152	156	220	224	245	249	296	300	200	214	192	254
03rbl.283	137	137	76	78	144	180	224	238	251	251	298	302	200	202	228	236
03rbl.284	137	137	76	76	156	156	232	232	249	255	302	302	200	210	234	234
03rbl.285	137	137	76	76	144	152	224	224	235	251	292	296	198	202	212	254
03rbl.294	135	135	74	76	152	162	212	230	247	247	298	312	200	200	226	238
03rbl.297	137	137	72	76	156	168	218	240	237	247	296	300	196	206	222	270
03rbl.298	137	137	76	76	144	156	222	234	243	249	296	302	196	200	224	240
03rbl.299	137	137	76	76	144	144	228	230	237	251	296	298	202	202	220	260
03rbl.305	135	137	76	76	152	156	220	238	249	249	296	300	160	200	228	254
03rbl.306	135	137	76	76	144	162	224	248	247	249	298	304	196	200	238	240
03rbl.307	137	137	76	76	156	160	224	234	241	251	300	302	202	202	212	238
03rbl.312	137	137	76	76	158	174	224	236	245	253	296	302	204	206	192	220
03rbl.316	137	137	76	76	156	162	220	224	239	247	294	298	198	202	192	220
03rbl.322	135	137	76	76	144	184	222	238	239	247	296	298	202	206	220	232
03rbl.325	135	137	76	76	144	158	226	232	247	249	292	292	160	202	214	256
03rbl.326	137	137	76	76	144	162	232	238	239	247	286	302	160	206	222	244
03rbl.327	137	137	76	78	144	174	224	240	251	253	296	302	180	206	234	242
03rbl.333	137	137	76	76	144	144	212	230	247	253	300	300	198	202	220	222
03rbl.334	135	137	76	76	144	188	230	248	245	251	296	300	200	206	192	242
03rbl.335	135	137	76	76	144	152	228	230	239	249	296	298	180	196	220	234
03rbl.337	137	137	76	76	154	156	224	234	235	245	294	302	160	198	220	240
03rbl.338	135	137	76	76	144	152	222	248	249	251	292	294	196	202	194	216
03rbl.340	135	137	76	76	156	156	230	238	239	255	296	300	202	206	230	232
03rbl.343	137	137	76	76	168	188	220	240	235	245	298	308	204	208	214	242
03rbl.349	137	137	76	76	144	156	224	226	247	249	300	306	200	204	230	236
03rbl.359	137	137	76	76	144	156	234	234	239	249	298	302	204	204	222	236
03rbl.372	137	137	76	76	144	174	222	240	247	249	302	308	198	204	234	236
04rbl.003	137	137	76	78	140	144	230	240	245	253	296	304	200	202	232	238
04rbl.005	137	137	76	76	144	144	240	240	235	245	292	302	202	204	220	238
04rbl.012	137	137	72	76	144	144	224	240	245	247	296	304	200	210	236	238
04rbl.017	137	137	76	76	144	144	222	230	245	255	296	302	202	202	220	230
04rbl.019	137	137	76	76	144	158	224	230	245	255	296	302	196	200	224	230
04rbl.028	137	137	76	76	144	154	230	240	235	245	292	296	200	206	220	252
04rbl.029	137	137	76	76	144	176	222	230	235	247	292	294	200	202	234	236
04rbl.031	137	137	76	76	144	144	222	230	245	249	296	302	198	202	234	236

04rbl.042	137	137	76	76	144	156	230	230	245	249	292	298	198	202	234	236
04rbl.047	137	137	76	78	156	156	214	224	235	239	292	296	194	200	234	244
04rbl.054	135	137	76	76	158	182	214	248	245	249	298	300	198	202	234	248
04rbl.060	137	137	76	76	144	156	232	252	239	241	292	302	160	206	234	236
04rbl.061	137	137	76	76	144	144	222	224	245	249	298	302	196	202	234	252
04rbl.064	137	137	76	76	144	156	226	230	247	251	296	306	180	202	218	238
04rbl.065	135	137	76	76	156	176	220	222	235	249	296	312	180	202	216	238
04rbl.066	137	137	76	76	144	144	214	238	241	249	300	306	196	198	234	236
04rbl.068	137	137	76	76	144	174	224	230	247	251	296	304	204	206	240	240
04rbl.081	137	137	76	76	144	162	220	224	245	247	296	300	204	204	236	242
04rbl.086	135	137	76	76	144	158	224	230	241	251	306	310	180	202	192	226
04rbl.088	137	137	76	76	144	152	240	240	245	245	294	298	196	208	214	214
04rbl.091	137	137	76	76	144	144	228	236	245	249	296	304	198	210	222	236
04rbl.094	137	137	76	76	144	164	230	238	239	245	310	316	196	204	210	238
04rbl.097	137	137	76	76	144	144	224	232	243	249	296	302	198	202	216	236
04rbl.100	137	137	76	76	156	160	224	232	245	247	296	298	160	202	220	242
04rbl.107	137	137	76	76	144	156	226	230	239	245	298	300	180	202	220	236
04rbl.118	137	137	76	78	144	156	230	246	241	251	296	296	196	196	212	238
04rbl.121	137	137	76	76	144	144	224	230	239	249	302	302	202	202	220	224
04rbl.126	135	137	74	76	144	152	224	230	247	247	296	300	198	208	222	238
04rbl.132	137	137	76	76	144	156	222	230	235	239	296	296	198	200	226	236
04rbl.137	137	137	76	76	144	164	226	230	235	251	298	306	202	204	234	238
04rbl.151	137	137	76	76	144	162	222	222	249	251	296	306	200	204	238	240
04rbl.152	137	137	72	76	144	144	240	248	249	249	296	298	200	202	220	236
04rbl.154	137	137	72	76	144	144	236	238	249	251	296	304	198	200	220	236
04rbl.160	137	137	76	76	144	162	238	242	247	257	294	302	196	206	220	240
04rbl.161	137	137	76	76	144	144	214	242	247	253	294	298	202	204	226	250
04rbl.164	137	137	76	76	144	144	226	228	239	245	300	302	180	200	222	244
04rbl.176	137	137	76	76	144	158	224	224	241	251	298	300	200	202	238	238
04rbl.177	137	137	76	76	144	144	232	236	249	255	296	302	202	204	236	238
04rbl.186	137	137	76	76	144	144	226	228	245	245	296	304	204	204	192	222
04rbl.190	137	137	76	76	144	144	224	230	239	247	298	298	200	202	232	234
04rbl.195	137	137	76	76	144	144	222	238	247	249	298	304	196	206	220	234
04rbl.201	135	137	76	76	150	156	228	240	249	251	300	302	206	208	238	246
04rbl.202	137	137	76	76	144	144	232	248	241	251	292	302	202	204	220	238
04rbl.205	137	137	76	76	144	160	224	242	247	249	302	306	200	204	236	242
04rbl.209	135	137	76	76	144	144	224	234	241	251	296	298	202	202	214	236

04rbl.210	135	137	76	76	144	152	222	248	239	239	270	300	200	204	222	236
04rbl.216	137	137	76	76	144	158	234	248	239	245	292	296	204	204	238	238
04rbl.220	137	137	76	76	144	158	226	240	249	249	304	308	202	202	230	234
04rbl.221	137	137	76	76	144	158	224	230	237	241	296	302	202	206	218	234
04rbl.224	137	137	76	76	144	144	222	224	239	247	296	302	198	202	218	234
04rbl.226	137	137	76	76	144	144	222	230	245	249	298	300	202	202	218	234
04rbl.228	137	137	76	76	144	156	230	244	239	249	296	310	196	200	192	252
04rbl.233	137	137	76	76	152	158	230	238	243	253	296	308	160	204	218	228
04rbl.237	137	137	76	78	144	158	222	240	243	249	296	306	160	200	236	242
04rbl.246	137	137	76	76	144	144	226	248	239	243	296	298	160	200	218	222
04rbl.249	137	137	76	76	144	154	230	234	247	249	300	302	196	204	220	236
04rbl.255	137	137	76	76	144	158	226	230	237	249	296	298	202	204	220	226
04rbl.266	135	137	76	76	144	164	222	224	241	251	296	302	196	200	238	252
04rbl.267	135	137	76	76	156	164	224	236	235	245	296	302	196	206	216	222
04rbl.272	135	137	76	76	144	156	228	236	235	245	300	310	198	200	236	236
04rbl.277	137	137	76	76	144	164	214	222	241	249	294	302	200	204	220	236
04rbl.279	135	137	76	76	144	162	232	240	245	249	294	300	204	204	222	234
04rbl.294	137	137	76	78	144	158	240	262	245	249	302	302	202	202	192	220
04rbl.297	135	137	76	76	144	144	220	242	247	249	292	302	198	200	218	222
04rbl.298	137	137	76	78	144	144	232	242	249	251	292	302	200	206	220	248
04rbl.300	137	137	76	76	144	190	214	230	235	245	298	302	198	204	222	234
04rbl.303	137	137	76	76	144	144	230	240	239	239	294	296	204	204	220	234
04rbl.306	137	137	76	76	144	144	224	230	247	249	298	302	198	200	218	222
04rbl.307	137	137	76	76	144	144	220	220	235	249	298	298	204	208	220	250
04rbl.312	137	137	76	76	144	144	228	230	247	249	298	302	200	200	216	220
04rbl.313	137	137	76	76	144	158	212	230	239	241	298	300	204	206	226	236
04rbl.316	137	137	76	76	144	154	226	234	239	241	298	300	202	204	192	228
04rbl.317	137	137	76	76	158	182	238	248	245	249	298	300	202	202	236	250
04rbl.330	137	137	76	76	144	158	222	246	235	245	298	300	204	208	234	234
04rbl.339	137	137	76	76	144	144	230	238	249	251	292	294	160	202	214	236
04rbl.345	137	137	76	76	144	160	230	238	249	249	298	302	196	202	228	230
04rbl.346	137	137	76	76	144	144	220	238	239	249	302	302	200	200	234	238
04rbl.348	137	137	76	76	144	158	224	230	245	249	294	302	202	204	238	240
04rbl.355	137	137	76	78	144	144	222	230	251	251	294	302	202	206	212	250
04rbl.358	135	135	76	76	144	162	222	234	245	249	294	302	200	206	212	236
04rbl.368	137	137	76	76	144	144	230	230	245	249	300	302	196	202	236	254
04rbl.371	137	137	76	76	144	144	228	230	245	249	298	302	160	196	220	244

04rbl.383	137	137	76	76	162	174	224	228	239	245	298	302	200	202	240	248
04rbl.384	137	137	76	76	144	144	226	246	239	245	274	306	202	202	236	246
04rbl.388	137	137	76	76	144	144	224	246	235	249	302	304	200	200	192	238
04rbl.390	137	137	76	76	156	176	218	234	245	247	298	306	200	202	246	254
04rbl.400	137	137	72	76	144	144	230	246	239	247	292	294	160	200	220	230
04rbl.404	135	137	76	76	144	144	220	234	241	243	298	300	178	202	230	236
04rbl.409	137	137	76	76	144	156	222	240	245	247	294	296	200	202	220	222
04rbl.425	137	137	76	76	144	144	230	264	245	245	292	296	202	202	220	236
04rbl.433	137	137	76	76	144	144	240	242	245	247	298	302	160	204	230	238
04rbl.441	137	137	76	76	144	154	224	228	239	255	296	296	200	202	230	232
04rbl.443	137	137	76	76	144	144	228	228	247	249	304	308	198	200	222	238
04rbl.446	137	137	76	76	144	156	220	238	249	251	296	298	196	196	218	230
04rbl.463	137	137	76	76	144	144	226	228	245	249	298	304	200	202	218	248
04rbl.464	135	137	76	76	144	144	222	238	249	249	0	0	200	204	220	254
04rbl.471	137	137	74	76	144	144	224	246	247	251	294	296	200	204	234	248
04rbl.472	137	137	76	76	144	144	234	246	247	251	294	302	160	204	220	222
04rbl.473	135	137	76	76	156	156	228	246	245	249	296	304	198	204	220	242
04rbl.474	137	137	76	76	144	144	224	234	237	251	296	300	202	204	232	238
04rbl.477	137	137	76	76	144	154	222	246	247	249	296	300	196	200	192	234
04rbl.480	137	137	76	76	144	162	230	238	249	249	306	314	202	204	234	244
04rbl.483	137	137	76	76	144	154	246	248	237	241	300	302	204	204	212	220
04rbl.484	137	137	76	76	144	152	224	230	245	251	302	302	196	204	220	244
04rbl.485	137	137	76	76	156	158	224	238	239	247	294	306	202	202	220	228
04rbl.491	137	137	76	76	144	156	222	242	245	245	300	302	202	206	192	230
04rbl.492	137	137	76	76	144	156	230	230	239	247	298	302	202	208	192	220
04rbl.493	137	137	76	76	144	144	224	242	235	239	298	298	202	208	232	236
04rbl.494	137	137	76	76	154	186	230	246	237	251	298	300	202	208	212	232
04rbl.495	137	137	76	76	144	156	226	230	245	247	296	300	200	206	220	248
04rbl.499	137	137	76	78	158	158	230	242	245	245	304	304	160	206	212	220
04rbl.502	135	137	76	76	144	156	238	248	247	247	296	304	202	204	234	236
04rbl.506	137	137	76	76	144	144	224	224	247	249	300	302	200	204	236	238
04rbl.519	137	137	76	76	144	156	230	234	245	247	300	302	198	204	224	252
04rbl.539	137	137	76	76	144	156	242	242	239	245	292	318	200	204	218	234
04rbl.545	135	137	76	76	144	144	224	230	239	239	296	330	204	210	228	234
04rbl.546	137	137	76	76	144	144	238	242	239	245	296	298	204	206	220	234
04rbl.550	137	137	76	76	144	144	212	230	239	249	296	304	196	202	234	236
04rbl.551	137	137	76	76	144	158	224	244	235	239	300	330	204	204	222	234

04rbl.556	137	137	76	76	144	144	240	242	239	241	292	330	202	210	228	236
05rbl.005	137	137	76	76	144	144	230	238	241	245	300	312	200	200	222	240
05rbl.009	135	137	76	76	156	170	222	224	237	239	298	300	200	210	234	234
05rbl.014	137	137	76	76	144	156	230	230	239	241	296	298	200	200	236	252
05rbl.022	137	137	76	78	144	156	222	230	239	245	300	308	200	206	236	238
05rbl.030	137	137	76	76	144	184	224	230	239	249	298	302	202	204	214	226
05rbl.033	137	137	76	76	144	144	212	224	245	245	296	302	200	202	212	238
05rbl.043	137	137	76	78	144	158	224	234	241	251	298	304	202	202	192	194
05rbl.044	137	137	76	76	144	144	228	248	247	257	296	304	200	206	226	240
05rbl.045	137	137	76	78	144	158	222	222	247	257	288	310	202	208	236	238
05rbl.053	135	137	76	76	144	164	212	224	245	245	296	302	200	202	212	230
05rbl.056	135	135	76	76	144	144	226	248	237	245	310	312	204	204	212	236
05rbl.058	135	137	76	76	144	144	220	230	237	247	298	302	202	204	218	242
05rbl.059	137	137	74	76	156	174	234	238	239	239	296	296	200	208	218	242
05rbl.061	137	137	76	78	144	158	224	224	247	249	298	302	196	200	234	238
05rbl.063	137	137	76	76	158	164	236	240	247	249	298	300	196	200	214	236
05rbl.065	137	137	76	76	144	170	212	234	247	249	298	312	202	204	234	236
05rbl.073	135	137	76	76	144	158	222	230	245	251	300	302	202	204	240	252
05rbl.078	137	137	76	76	144	180	230	238	245	249	302	302	160	200	240	242
05rbl.080	137	137	76	76	144	156	222	230	241	245	292	294	160	196	220	220
05rbl.081	137	137	76	78	152	156	224	226	245	249	296	302	202	202	216	224
05rbl.082	135	137	76	76	144	164	224	238	247	251	302	302	198	202	220	232
05rbl.083	137	137	76	76	144	156	214	224	237	247	294	302	196	202	218	220
05rbl.086	137	137	76	76	144	156	224	246	247	249	298	304	200	202	220	236
05rbl.090	137	137	76	76	144	156	228	242	239	245	302	302	202	202	234	236
05rbl.096	137	137	76	76	144	156	224	232	247	247	296	296	200	202	232	254
05rbl.099	135	135	76	76	144	156	222	226	237	249	294	296	198	206	192	246
05rbl.102	135	137	76	78	144	144	220	234	239	241	298	300	200	202	230	234
05rbl.108	137	137	76	76	144	156	236	238	239	249	296	304	196	196	230	232
05rbl.109	137	137	74	76	144	144	214	228	245	247	296	298	200	202	214	216
05rbl.117	137	137	76	76	156	156	230	234	239	239	298	298	196	200	192	234
05rbl.120	135	137	76	76	144	152	232	246	241	255	298	302	196	202	240	260
05rbl.121	137	137	76	76	144	144	248	248	249	249	296	302	198	206	212	236
05rbl.122	137	137	76	76	144	156	224	226	245	255	298	302	180	200	220	234
05rbl.123	137	137	76	76	144	176	222	242	245	249	296	296	196	206	212	246
05rbl.128	135	137	76	76	144	174	230	240	239	245	302	304	160	202	212	248
05rbl.134	137	137	76	76	144	158	222	232	239	245	296	306	200	206	234	234

05rbl.140	137	137	76	76	144	158	234	238	235	247	300	306	202	208	214	234
05rbl.144	137	137	76	76	144	144	224	238	239	245	294	298	198	202	220	222
05rbl.146	137	137	76	76	144	154	222	222	245	247	294	298	196	200	220	250
05rbl.147	137	137	76	76	144	144	248	250	247	247	296	308	160	200	226	234
05rbl.148	137	137	76	76	144	156	232	246	239	245	298	298	180	206	220	232
05rbl.149	137	137	76	76	144	144	238	250	247	247	292	298	200	210	230	246
05rbl.152	137	137	76	76	156	164	214	246	239	245	298	302	202	202	220	234
05rbl.154	137	137	76	76	158	172	222	246	241	249	296	304	198	200	238	248
05rbl.155	137	137	76	76	144	144	240	246	237	245	298	302	206	206	232	234
05rbl.156	137	137	76	78	144	144	226	242	239	239	302	308	180	200	222	264
05rbl.163	137	137	76	76	144	156	222	236	247	249	296	298	198	200	220	236
05rbl.164	137	137	76	76	144	156	212	222	245	245	296	300	202	204	236	240
05rbl.175	137	137	76	76	144	144	230	238	241	253	296	334	200	200	220	226
05rbl.180	135	137	76	76	144	144	220	230	239	245	302	304	200	206	236	238
05rbl.181	137	137	76	76	144	144	230	236	245	245	298	298	196	206	236	246
05rbl.183	137	137	76	76	144	144	222	222	253	253	296	296	196	200	222	240
05rbl.187	137	137	76	76	144	144	224	236	249	251	294	300	198	202	212	232
05rbl.190	137	137	76	76	144	176	220	224	251	253	292	296	196	202	222	238
05rbl.192	137	137	76	76	144	158	222	230	249	249	286	296	200	206	216	252
05rbl.198	137	137	76	76	144	180	232	238	239	245	298	304	202	212	218	238
05rbl.200	135	137	76	76	144	168	222	224	239	249	300	300	196	200	232	254
05rbl.201	137	137	76	78	144	180	224	238	239	251	294	300	198	200	212	232
05rbl.202	137	137	76	76	144	156	232	248	245	253	296	300	198	202	234	240
05rbl.206	135	137	76	76	144	144	222	248	247	253	296	300	196	196	240	254
05rbl.210	137	137	76	76	144	156	224	236	235	247	300	304	202	208	192	236
05rbl.211	137	137	76	76	144	156	228	248	237	249	294	302	196	200	222	246
05rbl.212	137	137	76	76	144	164	226	228	237	249	296	296	202	202	228	252
05rbl.215	137	137	76	76	144	144	238	240	247	249	304	304	160	204	230	236
05rbl.216	137	137	76	76	144	144	212	220	239	241	302	308	160	200	220	248
05rbl.220	137	137	76	76	144	144	224	252	245	255	292	300	160	202	194	212
05rbl.222	137	137	76	76	144	144	228	228	247	249	296	298	196	202	234	264
05rbl.223	137	137	76	76	144	144	222	232	241	253	296	300	200	202	220	254
05rbl.226	137	137	74	76	144	158	226	232	241	251	294	296	196	200	192	236
05rbl.233	137	137	76	76	144	144	224	230	241	245	300	300	198	200	212	250
05rbl.241	137	137	76	76	144	144	222	238	235	247	292	304	200	202	224	238
05rbl.242	137	137	76	78	156	176	230	230	247	249	300	302	200	200	222	236
05rbl.268	137	137	76	76	144	144	230	230	249	249	294	298	198	198	222	238

05rbl.270	137	137	76	76	144	144	222	246	245	245	296	304	202	202	238	240
05rbl.272	137	137	76	76	144	152	224	240	245	245	294	296	200	202	220	242
05rbl.273	137	137	76	76	144	144	224	230	245	245	292	302	196	202	220	234
05rbl.275	135	137	76	76	144	176	212	222	245	245	294	296	200	210	220	234
05rbl.284	137	137	76	78	144	144	214	230	245	249	292	296	198	200	222	252
05rbl.297	137	137	76	76	144	144	222	234	235	245	304	320	196	196	240	244
05rbl.298	137	137	76	76	144	144	230	230	239	247	294	296	196	200	242	256
05rbl.301	137	137	76	78	140	144	222	224	239	241	294	302	198	198	230	232
05rbl.302	135	137	76	76	144	164	230	230	239	245	286	294	202	204	220	244
05rbl.305	137	137	76	76	144	144	232	238	235	245	294	302	198	200	190	192
05rbl.306	137	137	74	76	144	144	226	240	239	249	298	300	196	204	226	240
05rbl.313	137	137	76	76	144	156	218	224	247	249	296	302	202	202	236	240
05rbl.314	137	137	76	76	144	176	228	228	245	251	298	304	160	202	226	238
05rbl.316	137	137	76	78	156	156	222	236	245	249	294	296	202	206	220	232
05rbl.320	137	137	76	76	144	144	224	226	251	251	302	304	196	206	236	236
05rbl.322	137	137	76	76	144	156	222	238	245	247	298	300	202	204	240	244
05rbl.324	137	137	76	76	144	144	222	224	235	253	296	298	196	202	230	242
05rbl.329	137	137	76	78	144	144	230	230	235	251	294	300	200	200	220	234
05rbl.331	137	137	76	76	144	158	224	230	245	253	300	302	208	210	234	236
05rbl.334	137	137	76	76	144	144	230	230	249	249	300	308	198	200	234	256
05rbl.343	137	137	76	76	144	156	224	232	241	243	298	302	204	204	232	246
05rbl.345	137	137	72	76	144	158	224	240	247	247	302	304	198	200	220	256
05rbl.349	137	137	76	76	156	156	230	240	253	253	300	302	160	204	236	236
05rbl.350	137	137	76	76	144	152	228	248	241	247	298	298	196	200	236	240
05rbl.351	137	137	76	76	144	152	214	230	239	247	296	304	202	204	192	240
05rbl.357	137	137	76	76	144	156	238	238	239	241	298	318	196	196	212	238
05rbl.362	137	137	76	76	144	162	222	232	235	249	302	302	200	204	220	234
05rbl.366	137	137	76	76	144	158	224	238	247	247	296	306	198	200	222	246
05rbl.367	137	137	76	76	144	150	220	250	241	253	294	296	202	210	220	240
05rbl.369	137	137	76	78	144	158	230	234	249	249	292	298	196	202	220	240
05rbl.370	137	137	72	76	144	158	232	242	243	249	300	302	200	202	236	252
05rbl.373	137	137	76	76	144	144	222	242	247	249	298	302	200	202	220	222
05rbl.377	137	137	76	76	144	144	224	230	247	255	294	302	202	208	212	234
05rbl.387	137	137	76	78	144	154	232	238	237	249	302	302	202	204	234	240
05rbl.390	137	137	76	76	144	160	220	224	239	249	292	304	200	202	220	250
05rbl.395	137	137	74	76	144	158	226	234	247	249	300	308	180	202	220	244
05rbl.398	137	137	76	76	156	168	220	234	241	249	296	300	160	200	212	222

05rbl.407	137	137	76	76	144	152	230	230	249	251	296	302	200	200	228	236
05rbl.408	135	137	76	76	172	174	234	242	245	249	296	296	196	200	238	238
05rbl.409	137	137	76	76	144	144	224	230	239	251	296	302	180	198	218	238
05rbl.414	137	137	76	76	158	160	224	230	239	245	298	300	198	202	236	236
05rbl.415	137	137	76	76	144	144	224	230	241	247	302	302	200	202	220	236
05rbl.416	135	137	76	78	144	156	230	230	245	247	296	302	200	206	192	238
05rbl.419	137	137	76	76	144	156	224	230	239	239	294	296	196	196	220	236
05rbl.421	137	137	76	76	144	158	220	224	241	249	300	302	200	204	192	252
05rbl.427	137	137	76	76	144	152	230	236	251	251	296	300	196	200	212	238
05rbl.429	137	137	76	76	144	166	222	242	239	245	300	328	196	200	234	234
06rbl.037	137	137	76	76	144	144	238	240	245	245	302	302	202	202	236	238
06rbl.043	137	137	76	76	144	144	214	230	239	241	302	316	198	200	238	240
06rbl.056	137	137	76	76	144	158	236	236	241	251	296	298	200	210	234	234
06rbl.057	137	137	76	76	144	176	212	230	247	251	292	302	160	204	234	252
06rbl.059	137	137	76	76	144	164	230	248	237	249	300	300	196	200	222	224
06rbl.061	137	137	76	76	144	190	222	236	239	251	296	316	200	202	242	256
06rbl.064	137	137	76	76	144	160	220	224	239	241	302	302	160	200	228	240
06rbl.075	137	137	76	76	144	162	238	240	247	251	294	298	208	208	222	232
06rbl.083	135	137	76	76	158	158	230	232	245	245	294	298	202	208	192	234
06rbl.087	137	137	76	76	158	166	222	230	247	249	296	296	206	206	220	240
06rbl.089	137	137	72	76	144	162	224	230	241	249	296	302	206	208	232	240
06rbl.090	137	137	76	76	144	144	222	242	249	249	300	318	196	202	192	220
06rbl.094	137	137	76	76	144	176	222	230	249	249	296	320	198	200	238	242
06rbl.095	137	137	76	78	144	144	222	230	247	247	292	304	198	204	230	236
06rbl.098	137	137	76	76	144	174	222	230	241	241	292	298	200	202	212	236
06rbl.103	137	137	76	76	144	156	228	236	247	251	298	302	200	202	238	250
06rbl.107	137	137	74	76	144	156	222	222	247	251	300	306	200	202	238	260
06rbl.109	137	137	76	76	144	180	228	228	241	243	302	302	200	200	192	238
06rbl.110	135	137	76	76	156	190	222	232	239	245	296	302	196	200	224	236
06rbl.119	137	137	76	76	152	180	230	230	241	249	300	300	204	204	226	226
06rbl.122	137	137	76	76	152	164	224	232	247	247	294	302	196	202	224	252
06rbl.127	137	137	76	78	156	156	228	264	235	245	296	304	200	202	212	222
06rbl.130	137	137	76	76	144	152	220	224	235	245	294	302	198	200	220	252
06rbl.133	137	137	76	78	144	152	230	246	239	251	292	300	198	202	222	238
06rbl.141	137	137	76	76	144	154	228	238	245	249	294	298	196	204	232	250
06rbl.144	137	137	76	76	144	158	230	246	241	249	294	306	160	196	232	238
06rbl.147	137	137	76	76	144	158	226	242	241	247	296	298	196	204	220	238

06rbl.158	137	137	76	76	144	158	222	248	235	239	294	298	200	202	220	220
06rbl.159	137	137	76	76	144	164	224	236	247	251	298	306	198	202	220	224
06rbl.161	135	137	76	76	144	156	224	246	247	251	296	300	200	206	216	222
06rbl.162	135	137	76	76	156	156	220	224	245	249	292	294	196	204	220	220
06rbl.163	137	137	76	76	144	176	224	248	237	239	294	300	200	200	220	250
06rbl.167	137	137	76	76	144	158	224	232	239	247	296	302	196	202	220	232
06rbl.168	137	137	76	76	158	162	222	224	239	245	296	302	160	196	220	238
06rbl.180	137	137	76	76	144	162	226	232	245	249	302	306	160	202	228	228
06rbl.187	137	137	76	78	156	158	222	224	247	251	294	304	202	208	220	220
06rbl.191	137	137	76	76	144	164	248	248	247	247	300	302	180	200	212	234
06rbl.201	135	137	76	76	152	156	226	240	239	247	298	302	200	200	236	242
06rbl.203	137	137	72	76	144	144	222	222	241	245	298	302	202	212	236	250
06rbl.206	137	137	76	76	144	158	222	232	245	249	296	308	160	200	212	216
06rbl.211	137	137	76	76	144	156	224	230	235	249	298	308	200	200	218	220
06rbl.231	137	137	76	78	144	144	230	230	247	251	296	298	204	204	234	246
06rbl.245	137	137	76	76	140	144	214	224	237	249	298	298	200	206	220	236
06rbl.247	137	137	72	76	144	158	220	230	241	245	300	302	200	206	236	236
06rbl.251	137	137	76	76	144	156	224	224	245	249	296	296	206	208	238	238
06rbl.252	137	137	76	76	144	144	230	230	245	251	294	310	202	204	220	236
06rbl.257	137	137	76	76	144	144	230	236	239	249	296	298	204	206	218	234
06rbl.258	137	137	76	76	144	154	224	232	245	245	298	302	204	206	220	238
06rbl.271	137	137	76	76	144	156	224	236	241	241	298	302	202	210	234	238
06rbl.273	137	137	76	76	144	144	230	230	245	249	296	310	198	202	236	242
06rbl.278	137	137	76	76	144	158	220	220	245	251	296	300	196	198	238	252
06rbl.283	137	137	76	76	144	158	230	238	249	257	308	308	196	204	218	258
06rbl.286	137	137	76	76	144	144	224	240	239	247	300	306	196	204	220	220
06rbl.288	137	137	76	76	144	158	240	242	239	249	296	296	198	200	222	240
06rbl.295	137	137	76	76	144	158	220	236	249	249	300	304	200	200	212	236
06rbl.301	137	137	74	76	144	150	224	224	245	249	304	312	200	206	222	252
06rbl.315	137	137	72	76	144	144	222	238	239	251	296	296	194	200	228	238
06rbl.317	137	137	76	76	144	144	230	248	243	249	298	302	198	200	212	236
06rbl.319	137	137	76	76	158	188	224	234	239	253	296	300	204	204	212	248
06rbl.320	137	137	76	78	144	152	230	246	245	249	300	304	196	204	222	238
06rbl.321	137	137	76	76	144	144	224	230	249	251	298	312	196	204	240	252
06rbl.322	137	137	76	76	144	144	230	230	251	251	294	302	204	206	212	234
06rbl.329	135	135	76	76	172	198	224	264	241	247	300	302	198	202	220	222
06rbl.336	137	137	76	76	144	144	224	242	243	243	296	302	196	208	218	220

06rbl.339	137	137	76	76	144	144	228	262	239	241	296	310	200	200	234	234
06rbl.341	137	137	76	76	144	144	222	236	241	253	298	302	196	202	236	238
06rbl.349	137	137	76	76	164	164	230	248	247	253	298	298	200	202	234	240
06rbl.355	137	137	76	76	144	144	230	238	247	247	294	296	196	198	222	238
06rbl.356	137	137	76	76	144	154	222	230	241	243	296	300	196	202	228	240
06rbl.372	137	137	76	76	144	144	220	230	241	249	296	298	198	202	222	240
06rbl.375	137	137	76	76	144	166	224	224	247	249	294	296	180	200	234	242
06rbl.382	137	137	76	76	144	158	242	266	241	247	296	298	204	206	222	242
06rbl.383	135	137	76	76	150	156	230	238	241	249	288	298	160	200	238	246
06rbl.386	137	137	76	76	144	144	238	266	241	251	298	298	202	204	220	246
06rbl.388	137	137	76	76	144	144	220	230	247	251	300	306	206	212	236	238
06rbl.389	137	137	76	76	144	144	224	224	237	249	304	304	196	204	222	250
06rbl.390	137	137	76	76	156	156	220	240	251	253	302	302	202	208	238	240
06rbl.391	137	137	76	78	144	144	222	230	251	253	296	300	204	204	222	222
06rbl.395	137	137	76	76	144	166	224	230	241	247	294	298	204	206	242	246
06rbl.397	137	137	76	76	150	156	232	232	241	241	288	298	202	204	220	238
06rbl.420	135	137	76	76	144	144	232	260	241	249	294	300	198	204	238	238
06rbl.421	137	137	76	76	144	158	224	248	241	251	294	296	196	198	192	234
06rbl.424	137	137	76	76	144	158	222	232	241	251	302	302	202	202	214	238
06rbl.452	137	137	76	76	144	158	220	220	245	247	298	306	202	202	220	254
06rbl.460	137	137	76	76	144	150	222	224	239	245	296	302	196	208	220	236
06rbl.469	137	137	76	76	144	144	222	238	235	247	296	306	196	196	224	232
06rbl.471	137	137	76	76	144	156	220	232	245	245	300	300	202	204	192	232
06rbl.475	137	137	74	76	144	144	226	230	239	245	294	306	202	204	218	248
06rbl.478	137	137	76	76	144	144	224	238	245	249	300	320	202	204	238	246
06rbl.481	137	137	76	76	144	144	220	230	245	245	296	312	204	206	192	248
06rbl.488	137	137	76	76	144	144	222	232	245	245	298	302	196	200	212	236
06rbl.495	137	137	76	78	144	156	224	246	245	245	298	302	200	202	236	238
06rbl.501	137	137	76	78	144	144	230	230	245	245	296	298	202	204	212	220
06rbl.508	137	137	76	78	144	144	224	234	241	245	300	302	200	200	212	240
06rbl.509	137	137	76	76	158	160	230	238	251	255	298	302	206	210	222	232
06rbl.515	137	137	76	78	144	158	232	238	235	245	298	304	196	198	222	224
06rbl.522	137	137	76	78	144	144	222	230	247	249	296	306	200	204	236	238
06rbl.526	137	137	76	76	144	144	226	232	241	249	296	306	196	202	230	240
06rbl.534	135	137	76	76	152	152	230	256	241	253	296	298	196	202	222	236
06rbl.536	137	137	76	76	144	144	224	238	241	249	304	304	198	208	236	252
06rbl.545	137	137	76	76	144	144	212	238	241	251	300	302	198	198	218	236

06rbl.548	137	137	76	76	144	144	240	240	247	249	300	304	198	202	212	244
06rbl.551	137	137	76	76	144	158	212	224	241	247	300	302	198	204	220	242
06rbl.552	137	137	76	76	144	156	220	244	241	249	298	302	198	206	218	234
06rbl.553	137	137	76	78	144	144	230	230	249	249	296	302	202	204	234	236
06rbl.565	137	137	76	76	156	156	236	248	241	243	300	300	198	206	220	238
06rbl.568	137	137	76	76	144	162	228	238	241	249	300	306	202	202	218	232
06rbl.577	137	137	76	76	156	158	224	234	237	247	300	302	204	208	224	232
06rbl.579	137	137	76	76	144	144	242	244	239	249	298	298	202	206	192	220
06rbl.580	137	137	76	76	144	144	224	236	235	249	300	306	198	202	234	236
06rbl.581	137	137	76	78	176	176	214	226	251	251	302	304	202	212	226	252
06rbl.583	137	137	76	76	164	164	222	234	239	239	298	302	202	206	242	250
06rbl.584	137	137	76	76	144	160	222	238	239	253	300	304	204	206	238	260
06rbl.588	137	137	76	76	144	144	222	234	249	251	302	302	200	204	228	240
06rbl.590	137	137	76	76	158	158	224	230	247	249	296	306	198	200	236	250
06rbl.598	137	137	76	78	144	158	230	230	237	247	300	302	196	202	192	234
06rbl.600	137	137	76	76	144	144	220	224	239	247	302	304	204	212	228	230
06rbl.611	137	137	76	76	144	144	224	224	239	243	304	312	160	196	220	236
06rbl.623	137	137	76	76	144	156	220	224	241	245	298	306	206	208	222	238
06rbl.628	137	137	76	76	144	144	230	230	247	249	302	308	202	202	242	250
07rbl.001	135	137	76	76	144	144	222	228	245	247	302	304	198	202	212	222
07rbl.003	137	137	76	76	144	158	230	242	245	249	296	302	200	202	192	220
07rbl.007	137	137	76	76	156	156	222	248	247	249	292	300	202	206	224	238
07rbl.008	137	137	76	76	144	144	238	238	247	249	296	318	200	206	230	252
07rbl.009	137	137	76	76	144	144	230	242	237	247	296	298	202	206	192	220
07rbl.010	137	137	76	76	144	164	226	230	245	249	296	302	200	200	192	222
07rbl.014	137	137	76	76	144	156	224	224	237	249	292	298	196	198	238	238
07rbl.015	137	137	76	76	152	162	220	242	247	255	296	302	202	202	192	236
07rbl.016	137	137	76	76	152	156	224	230	247	255	296	298	200	202	222	240
07rbl.019	137	137	76	78	144	156	222	230	235	249	298	298	202	202	236	238
07rbl.021	137	137	76	76	152	156	220	224	241	245	294	298	198	200	222	234
07rbl.023	135	135	76	76	144	152	230	230	245	247	294	294	160	202	234	250
07rbl.024	137	137	76	76	144	144	230	234	245	245	286	294	202	206	220	238
07rbl.028	135	137	76	76	144	144	222	238	237	247	274	292	160	200	226	242
07rbl.029	137	137	76	76	152	156	222	242	239	245	300	300	200	202	192	216
07rbl.033	137	137	76	76	144	144	228	230	249	249	296	302	198	206	198	234
07rbl.045	135	137	76	76	144	144	230	252	249	249	294	300	196	198	212	220
07rbl.051	135	137	76	76	156	156	224	230	241	251	296	302	196	206	218	232

07rbl.054	137	137	76	76	144	160	212	238	243	247	294	298	200	200	220	232
07rbl.055	137	137	76	76	144	144	224	230	235	247	296	306	196	208	224	234
07rbl.057	137	137	76	78	144	156	230	238	245	255	300	310	196	206	214	234
07rbl.058	137	137	76	76	144	152	224	248	239	247	302	304	202	202	222	226
07rbl.060	135	137	76	76	144	158	228	230	237	249	298	304	196	202	236	258
07rbl.063	135	137	76	76	144	160	230	236	245	251	296	302	200	202	232	234
07rbl.067	137	137	76	76	144	144	220	238	235	239	294	308	198	200	236	242
07rbl.069	137	137	76	76	156	164	224	238	241	247	298	304	198	202	192	232
07rbl.070	135	137	76	76	144	160	222	224	245	255	298	302	196	204	240	240
07rbl.072	137	137	76	76	144	156	230	230	241	245	292	296	196	206	220	226
07rbl.073	137	137	76	76	144	152	224	230	245	249	296	304	200	202	222	224
07rbl.076	137	137	76	76	144	158	224	242	247	249	302	306	196	206	220	232
07rbl.078	137	137	76	76	144	156	226	226	249	251	296	302	160	200	234	238
07rbl.080	135	137	76	76	144	144	244	248	239	247	298	300	196	200	192	194
07rbl.082	135	137	76	76	144	144	222	232	241	245	296	302	200	200	218	236
07rbl.083	137	137	72	76	158	162	224	224	239	247	290	302	200	200	192	234
07rbl.085	137	137	74	76	144	154	224	240	249	253	300	300	196	200	220	228
07rbl.089	137	137	76	76	156	160	224	240	245	249	298	304	202	208	220	236
07rbl.091	137	137	76	76	144	144	222	228	245	245	296	300	160	198	216	236
07rbl.094	137	137	76	78	144	158	222	228	239	249	294	298	202	202	220	252
07rbl.095	137	137	76	76	144	164	226	240	239	251	288	300	202	204	216	236
07rbl.097	137	137	76	76	144	144	224	226	245	251	302	304	200	200	232	234
07rbl.099	135	137	76	76	144	156	222	238	239	245	300	302	200	200	222	248
07rbl.106	135	137	76	78	144	144	224	230	245	255	304	306	200	202	232	236
07rbl.110	135	137	76	78	144	156	222	230	241	249	298	306	200	210	238	238
07rbl.111	137	137	76	76	144	180	222	230	239	245	300	302	202	206	220	234
07rbl.113	137	137	76	76	144	156	222	222	247	251	296	296	200	200	228	236
07rbl.118	137	137	76	76	144	156	226	242	239	247	294	298	200	206	212	236
07rbl.122	137	137	76	78	144	156	224	230	235	241	300	300	196	200	220	240
07rbl.123	137	137	76	76	144	166	224	248	237	251	292	296	202	202	226	226
07rbl.124	137	137	76	76	144	164	240	248	247	247	296	300	206	210	216	220
07rbl.125	137	137	76	76	144	156	224	248	245	247	292	298	204	206	222	244
07rbl.130	137	137	76	76	144	156	242	242	237	247	298	298	196	200	244	246
07rbl.132	135	137	76	76	144	144	230	234	239	251	298	298	206	208	214	242
07rbl.136	137	137	76	76	154	156	222	230	247	249	300	302	160	200	214	224
07rbl.139	137	137	76	78	144	156	230	230	235	247	298	298	202	206	212	220
07rbl.144	137	137	76	76	144	144	222	230	239	249	300	302	200	202	236	238

07rbl.152	137	137	76	78	144	156	226	242	235	245	296	304	202	206	236	236
07rbl.158	135	137	76	76	144	164	226	230	237	251	292	296	196	196	214	222
07rbl.159	137	137	76	76	144	144	226	232	245	253	296	308	194	204	222	238
07rbl.164	137	137	76	76	144	144	222	232	237	239	296	298	200	200	212	236
07rbl.167	137	137	76	76	144	144	236	244	245	249	274	296	198	202	236	236
07rbl.168	137	137	76	76	144	144	226	238	245	251	294	306	194	198	236	238
07rbl.171	137	137	76	76	156	172	214	242	241	249	296	300	196	196	236	236
07rbl.179	137	137	76	76	144	152	240	244	245	245	296	300	200	202	238	254
07rbl.180	137	137	76	76	144	174	224	236	247	249	296	296	198	200	238	248
07rbl.182	137	137	76	78	144	156	230	242	239	245	298	302	198	210	220	250
07rbl.183	137	137	76	78	144	156	224	238	239	245	294	296	196	200	220	248
07rbl.186	137	137	76	76	144	144	222	244	241	249	302	302	204	204	202	220
07rbl.187	137	137	72	76	144	144	222	238	245	247	296	296	200	206	192	240
07rbl.188	135	137	76	76	144	174	222	224	237	239	292	300	196	204	220	252
07rbl.189	135	137	76	76	144	156	222	232	245	249	292	304	160	206	220	234
07rbl.192	137	137	76	76	156	174	232	234	237	247	294	300	196	204	242	244
07rbl.195	137	137	76	76	144	144	222	238	239	239	294	296	202	202	218	234
07rbl.196	137	137	76	76	144	156	226	228	237	237	304	306	200	202	232	236
07rbl.203	137	137	72	76	144	144	224	232	247	251	296	298	200	202	220	258
07rbl.205	137	137	76	76	144	144	240	240	247	249	296	296	198	200	220	234
07rbl.210	137	137	76	76	152	160	240	240	241	245	290	298	206	206	220	226
07rbl.211	137	137	76	76	156	160	232	240	245	247	292	302	196	196	224	252
07rbl.212	137	137	76	76	158	180	222	228	239	249	296	302	202	202	216	230
07rbl.213	137	137	76	76	144	156	232	248	247	249	298	300	196	202	234	270
07rbl.214	137	137	76	76	144	168	224	232	247	253	300	302	196	200	242	254
07rbl.215	135	137	76	76	144	162	224	236	239	245	300	302	196	196	238	252
07rbl.217	137	137	76	76	144	158	224	238	247	251	296	300	204	204	222	238
07rbl.219	137	137	76	76	156	164	230	232	245	245	296	298	202	202	212	238
07rbl.220	137	137	76	78	144	156	224	230	245	251	298	300	198	202	220	234
07rbl.221	137	137	76	76	144	144	238	242	245	249	298	300	200	202	226	240
07rbl.225	137	137	74	76	156	166	224	230	239	241	294	298	200	202	218	252
07rbl.229	137	137	76	76	144	176	224	240	247	249	294	298	198	202	212	218
07rbl.233	137	137	76	76	144	144	220	230	245	249	300	300	200	204	220	236
07rbl.235	135	137	76	76	144	164	222	230	245	249	290	300	160	210	220	254
07rbl.236	135	137	76	76	144	158	230	230	245	247	302	302	200	210	192	220
07rbl.238	137	137	76	76	144	156	222	238	239	239	296	296	202	202	220	250
07rbl.240	137	137	76	78	154	160	230	238	237	237	300	302	196	200	192	218

07rbl.242	137	137	76	76	144	144	240	240	247	247	298	300	160	206	218	220
07rbl.243	137	137	76	76	144	144	228	252	249	249	298	302	200	200	224	238
07rbl.245	137	137	76	76	144	156	234	240	253	253	300	304	204	204	220	220
07rbl.251	137	137	76	78	144	156	222	238	239	245	300	300	200	200	226	234
07rbl.255	135	137	72	76	144	162	230	230	245	247	298	298	180	202	212	250
07rbl.258	137	137	76	76	144	144	220	226	245	247	300	308	160	206	228	234
07rbl.259	137	137	76	78	144	144	222	230	239	251	300	304	200	202	240	252
07rbl.262	137	137	76	76	144	156	230	248	235	245	300	300	196	202	220	252
07rbl.264	137	137	72	76	144	156	222	230	241	249	304	306	210	210	232	238
07rbl.266	137	137	76	76	152	156	236	238	241	249	300	302	204	204	220	252
07rbl.269	137	137	72	76	144	144	226	232	241	249	302	302	160	202	218	238
07rbl.271	137	137	76	76	144	156	230	240	235	249	300	306	198	202	220	226
07rbl.272	135	137	76	76	144	158	222	222	237	245	296	298	200	202	190	226
07rbl.275	137	137	76	76	144	156	222	222	247	251	300	306	196	206	222	240
07rbl.278	137	137	76	76	144	156	224	248	247	249	302	304	200	202	216	238
07rbl.279	137	137	76	78	144	156	230	248	247	249	294	302	200	202	222	238
07rbl.281	137	137	76	76	144	156	224	226	237	249	292	292	202	204	238	250
07rbl.284	137	137	76	76	144	158	224	234	245	249	292	300	204	206	238	238
07rbl.286	137	137	76	78	144	152	222	230	247	249	296	300	200	200	234	260
07rbl.290	137	137	76	76	144	156	224	228	239	251	298	302	202	202	220	236
07rbl.295	137	137	76	76	144	158	230	236	237	249	292	302	194	200	220	234
07rbl.296	137	137	76	76	144	156	218	224	245	245	304	308	196	200	230	240
07rbl.300	137	137	76	76	144	176	222	232	237	247	298	300	202	204	234	234
07rbl.301	137	137	76	76	144	156	214	242	239	251	290	300	200	200	220	226
07rbl.302	137	137	76	76	144	152	222	224	245	249	292	304	202	210	234	238
07rbl.303	137	137	76	78	144	156	230	246	247	251	296	302	200	202	234	240
07rbl.304	135	137	76	76	144	156	220	230	241	251	296	304	196	202	212	254
07rbl.309	135	137	76	76	144	156	226	238	237	245	300	310	160	200	220	234
08rbl.011	135	137	76	76	144	152	226	230	235	247	298	300	160	196	220	244
08rbl.015	135	137	76	76	144	144	220	230	245	247	300	304	198	200	234	234
08rbl.020	135	137	76	76	144	144	222	238	235	249	298	300	202	208	220	240
08rbl.021	137	137	76	76	160	160	224	234	245	245	294	296	204	204	192	234
08rbl.023	135	137	76	76	144	176	230	236	247	249	300	304	198	200	220	232
08rbl.025	137	137	76	76	144	164	222	230	235	245	300	302	160	200	194	220
08rbl.027	137	137	76	76	144	144	222	230	241	251	300	300	202	206	232	240
08rbl.033	137	137	76	76	156	156	224	224	235	245	298	302	196	198	224	236
08rbl.034	137	137	76	76	144	154	220	238	245	249	292	294	160	202	220	222

08rbl.041	135	137	72	76	144	144	224	244	241	251	298	302	200	210	212	238
08rbl.045	137	137	76	76	144	156	230	240	245	245	296	306	196	196	220	238
08rbl.054	135	137	76	76	144	144	220	230	249	249	294	298	200	210	216	240
08rbl.055	137	137	76	76	144	156	228	242	239	243	296	304	202	208	220	242
08rbl.059	137	137	76	76	144	144	234	240	239	247	294	298	200	206	236	256
08rbl.061	135	137	72	76	150	154	214	234	241	251	294	298	200	202	238	238
08rbl.064	135	137	76	76	144	156	226	236	237	239	300	332	196	200	222	240
08rbl.070	137	137	76	76	156	164	222	248	247	249	306	308	196	200	216	256
08rbl.074	137	137	76	76	144	144	236	236	235	241	302	304	204	204	226	228
08rbl.079	137	137	76	76	144	144	232	242	249	251	294	302	202	204	238	252
08rbl.081	137	137	76	76	160	164	222	230	241	245	298	302	200	202	212	232
08rbl.085	137	137	76	76	144	152	222	240	237	245	302	304	198	200	212	222
08rbl.124	137	137	76	76	144	144	236	238	239	255	296	306	194	198	238	252
08rbl.129	137	137	76	76	144	156	230	230	239	239	286	294	200	202	238	252
08rbl.144	135	137	76	78	144	156	224	230	239	247	292	300	204	206	192	222
08rbl.153	135	137	76	76	144	152	224	252	237	247	294	300	200	202	214	218
08rbl.155	135	137	76	76	144	174	224	228	243	251	304	310	200	210	234	238
08rbl.156	137	137	76	78	144	144	230	230	243	245	298	304	200	202	212	236
08rbl.159	135	135	76	76	156	174	222	222	237	245	294	302	196	200	230	236
08rbl.162	137	137	76	78	156	156	222	232	239	243	296	298	196	204	238	252
08rbl.165	137	137	76	76	144	164	230	232	247	251	292	296	200	202	220	222
08rbl.167	137	137	76	78	144	180	232	232	241	245	298	308	200	204	214	220
08rbl.170	137	137	76	78	156	158	224	228	239	245	298	318	196	204	230	248
08rbl.174	137	137	76	76	144	156	222	236	239	239	296	302	202	204	238	252
08rbl.175	137	137	76	76	156	156	230	240	241	249	296	296	196	204	234	238
08rbl.176	137	137	76	76	156	176	224	226	247	247	298	298	196	202	222	238
08rbl.177	137	137	76	76	156	156	222	224	251	251	298	300	180	200	234	236
08rbl.182	137	137	76	76	144	144	224	226	235	245	298	300	196	200	250	252
08rbl.189	137	137	76	76	156	156	232	232	241	245	296	296	188	200	234	236
08rbl.196	135	137	76	76	144	156	226	230	249	249	296	302	198	202	224	234
08rbl.210	137	137	74	76	144	158	224	224	247	255	294	302	196	200	238	252
08rbl.211	135	137	76	76	144	144	224	248	247	249	298	302	200	202	230	240
08rbl.215	135	137	76	76	144	156	230	252	243	247	298	298	208	212	232	236
08rbl.225	137	137	76	76	144	156	224	232	245	249	302	302	160	200	230	236
08rbl.231	137	137	76	76	144	164	230	230	237	241	300	300	194	208	218	236
08rbl.242	137	137	76	76	144	154	230	230	239	249	298	302	196	204	216	248
08rbl.244	137	137	76	76	144	144	222	242	245	249	294	302	200	202	222	238

08rbl.245	137	137	76	76	144	156	222	236	239	247	292	300	202	202	236	236
08rbl.248	137	137	76	76	144	158	234	234	245	249	298	302	198	200	192	242
08rbl.260	135	137	76	76	144	144	242	242	249	251	304	308	198	202	218	236
08rbl.277	135	137	76	76	144	176	230	238	245	247	296	296	200	202	232	238
08rbl.284	137	137	76	76	144	156	222	250	237	245	294	302	202	202	232	236
08rbl.289	137	137	76	76	144	156	224	260	235	245	298	304	202	202	224	236
08rbl.295	137	137	76	76	156	160	230	240	245	247	306	310	196	206	216	220
08rbl.302	135	137	76	76	144	144	222	230	239	241	304	330	196	202	218	236
08rbl.303	137	137	74	76	144	156	228	230	237	241	302	302	200	206	230	240
08rbl.308	137	137	76	76	144	176	222	230	245	251	288	308	202	204	236	238
08rbl.313	137	137	76	76	176	188	222	224	247	251	288	296	200	202	242	244
08rbl.315	137	137	76	76	144	156	224	230	235	245	292	294	160	196	234	252
08rbl.318	135	137	76	76	144	144	240	248	251	253	304	306	204	208	220	238
08rbl.325	137	137	76	76	144	144	226	228	239	253	296	302	200	202	234	234
08rbl.327	135	137	76	76	144	156	232	232	237	245	300	306	198	202	218	234
08rbl.337	137	137	76	76	144	160	226	244	245	249	292	300	204	210	226	230
08rbl.347	137	137	76	76	144	154	230	256	235	249	302	302	160	204	192	230
08rbl.356	137	137	76	76	144	170	224	230	245	245	300	302	200	202	220	250
08rbl.360	137	137	72	76	144	158	232	258	239	245	302	304	160	200	230	238
08rbl.363	137	137	76	76	158	166	220	256	249	251	296	306	202	204	216	234
08rbl.368	135	137	76	76	144	158	230	238	237	239	296	302	196	200	234	240
08rbl.371	137	137	72	76	144	156	226	238	239	247	296	302	160	196	218	234
08rbl.374	137	137	76	76	144	156	222	224	241	247	294	296	198	202	220	232
08rbl.380	137	137	76	76	144	156	230	230	239	245	300	302	196	204	226	234
08rbl.384	137	137	76	76	144	144	232	232	247	249	304	306	194	200	234	236
08rbl.397	137	137	76	76	156	158	222	230	241	241	294	298	208	210	234	236
08rbl.406	137	137	76	78	144	144	232	232	239	249	298	300	208	212	234	254
08rbl.412	137	137	76	76	152	156	224	230	249	255	296	302	206	206	234	246
08rbl.415	137	137	76	76	144	144	232	240	249	249	294	302	196	202	236	236
08rbl.418	137	137	76	76	144	156	232	232	247	257	300	304	206	206	236	254
08rbl.425	137	137	76	76	144	144	222	222	239	247	296	300	202	202	220	242
08rbl.430	137	137	76	76	144	152	224	232	247	247	292	304	200	204	220	252
08rbl.442	137	137	72	76	144	144	232	234	241	249	300	302	202	204	238	254
08rbl.446	137	137	76	76	144	144	224	230	241	243	304	304	196	202	222	234
08rbl.447	137	137	76	76	144	156	240	242	249	253	304	304	200	202	246	256
08rbl.449	137	137	76	78	144	162	240	242	237	239	298	304	204	204	194	246
08rbl.450	137	137	76	76	144	144	240	242	239	241	294	300	204	204	196	222

08rbl.459	137	137	76	76	144	156	224	224	235	247	294	298	160	196	234	252
08rbl.466	137	137	76	78	144	144	222	242	239	249	298	300	196	204	196	222
08rbl.467	137	137	76	76	158	158	222	228	239	243	296	308	196	196	244	252
08rbl.470	137	137	76	76	144	144	226	240	247	249	296	304	198	212	220	246
08rbl.475	137	137	76	76	144	144	230	242	241	241	292	292	206	206	226	240
08rbl.482	137	137	76	76	144	154	224	242	245	249	296	296	204	206	240	248
08rbl.487	137	137	76	76	144	180	224	240	237	241	294	298	196	206	220	220
08rbl.493	137	137	76	78	144	144	212	240	239	247	294	298	196	206	220	238
08rbl.497	137	137	76	76	156	164	232	232	235	245	298	306	180	202	230	252
08rbl.502	137	137	76	78	144	156	222	226	247	249	296	298	160	200	192	250
08rbl.508	137	137	76	76	144	152	226	232	239	247	300	300	196	208	218	236
08rbl.509	137	137	76	76	144	144	224	238	241	245	300	302	180	200	238	242
08rbl.511	137	137	76	78	144	144	244	248	247	247	306	306	202	208	232	238
08rbl.513	137	137	76	78	144	144	224	230	247	257	294	296	204	204	230	236
08rbl.516	135	137	76	76	144	152	248	248	247	249	292	300	202	208	220	250
08rbl.523	135	137	76	78	144	144	230	252	247	249	304	310	204	204	192	232
08rbl.530	137	137	76	76	144	144	230	238	237	245	296	304	180	204	216	236
08rbl.531	137	137	76	76	144	144	222	226	239	249	270	310	202	206	220	226
08rbl.532	135	137	76	76	152	156	248	260	245	247	294	296	200	200	234	244
08rbl.534	137	137	76	76	144	144	224	230	247	249	296	300	198	204	220	236
08rbl.542	137	137	76	76	144	144	226	232	253	253	306	308	202	204	230	240
08rbl.553	135	137	76	76	144	156	224	262	245	249	296	334	202	204	240	240
08rbl.554	135	137	76	76	144	156	226	240	237	239	300	302	202	204	226	238
08rbl.565	135	137	76	76	156	184	226	242	239	245	304	308	200	202	220	232
08rbl.569	137	137	76	76	144	144	248	248	241	249	294	298	200	200	238	240
08rbl.573	137	137	76	76	144	144	222	224	239	245	296	302	208	208	214	236
08rbl.574	137	137	76	76	144	144	232	240	239	251	300	300	196	196	218	238
08rbl.608	137	137	76	76	144	144	224	240	243	245	304	318	160	198	228	232
08rbl.613	137	137	76	76	144	144	224	230	237	245	298	300	160	206	218	236
08rbl.614	137	137	76	76	144	166	224	226	237	237	296	298	200	202	220	222
08rbl.619	135	137	76	76	144	156	220	244	239	247	292	306	180	200	192	270
08rbl.620	135	137	76	76	144	144	240	240	243	245	292	292	204	204	222	238
08rbl.621	137	137	76	76	144	156	230	234	239	247	294	298	196	208	220	236
08rbl.624	137	137	76	76	144	152	232	232	245	247	294	298	196	204	226	232
08rbl.625	137	137	76	76	144	144	230	248	239	245	274	292	200	202	214	232
08rbl.628	135	137	76	76	156	158	222	230	241	245	292	296	196	200	236	240
08rbl.629	137	137	76	76	144	144	230	260	239	245	292	298	196	200	192	218

09rbl.001	137	137	76	76	144	156	220	224	243	245	298	300	196	200	222	238
09rbl.004	137	137	76	76	144	144	220	226	243	251	298	298	200	204	236	236
09rbl.005	137	137	72	76	144	144	212	230	247	251	284	298	196	200	192	244
09rbl.007	135	137	76	76	144	162	228	248	245	245	298	302	200	202	218	258
09rbl.009	135	137	76	78	156	156	224	236	249	253	296	304	198	200	238	238
09rbl.013	137	137	76	76	144	156	228	228	245	245	298	300	202	204	234	234
09rbl.014	135	137	76	76	144	144	240	260	241	251	292	294	196	202	218	234
09rbl.018	137	137	76	76	144	158	214	228	249	251	296	300	200	202	192	220
09rbl.026	137	137	74	78	144	172	214	230	251	255	296	298	202	202	240	260
09rbl.030	135	137	76	76	144	144	222	232	249	249	294	298	200	206	220	238
09rbl.031	137	137	76	78	144	156	230	240	245	249	292	298	206	208	220	252
09rbl.032	137	137	76	76	156	180	230	238	247	249	294	300	196	200	220	252
09rbl.033	137	137	76	76	144	188	222	238	251	253	296	302	198	202	192	234
09rbl.036	137	137	76	76	144	156	222	230	249	249	292	296	160	196	216	234
09rbl.040	135	137	76	76	144	156	234	244	249	251	302	302	196	200	234	240
09rbl.043	137	137	76	76	160	162	222	240	249	249	302	306	202	206	192	226
09rbl.045	137	137	76	76	156	164	230	240	249	253	292	296	196	200	234	234
09rbl.047	137	137	76	76	144	160	236	238	249	253	298	298	198	208	220	238
09rbl.048	137	137	76	76	144	144	224	238	249	251	302	306	206	208	226	238
09rbl.082	135	137	76	76	144	144	226	242	243	243	302	308	200	202	222	234
09rbl.086	135	137	76	76	144	144	224	234	241	243	294	296	200	206	212	226
09rbl.092	137	137	76	76	156	158	224	238	243	243	300	308	200	202	228	238
09rbl.105	135	135	76	76	144	156	234	240	249	251	296	298	198	206	222	240
09rbl.109	137	137	76	76	144	152	222	236	243	255	298	306	202	214	238	238
09rbl.111	137	137	76	78	144	158	224	230	249	249	292	302	200	204	246	250
09rbl.112	137	137	76	76	156	158	240	242	251	255	292	306	198	202	236	236
09rbl.113	137	137	76	76	144	156	224	230	249	249	294	298	198	198	222	238
09rbl.117	137	137	76	78	144	144	224	240	249	253	290	292	200	212	218	232
09rbl.118	137	137	76	76	144	174	228	230	249	255	300	306	198	198	236	238
09rbl.121	137	137	76	76	144	156	222	230	245	255	298	306	200	202	240	252
09rbl.123	137	137	76	76	144	144	236	238	251	255	294	320	210	214	218	252
09rbl.125	137	137	76	76	144	144	228	234	241	251	298	302	180	196	250	270
09rbl.133	135	137	76	76	156	160	214	248	239	251	296	296	202	204	222	238
09rbl.134	135	137	76	76	144	176	222	232	243	243	300	308	200	200	218	220
09rbl.137	137	137	76	76	154	158	226	248	249	249	294	300	198	206	236	242
09rbl.139	137	137	76	76	144	158	224	228	241	249	300	304	200	202	218	238
09rbl.153	135	137	76	76	144	176	228	242	249	253	300	300	198	202	220	238

09rbl.157	137	137	76	76	144	156	222	222	251	253	300	302	202	204	222	228
09rbl.159	137	137	76	78	156	164	222	230	249	251	300	302	200	200	216	240
09rbl.167	137	137	76	76	144	144	224	240	251	253	300	300	198	200	238	250
09rbl.168	137	137	76	76	144	156	232	232	251	251	296	296	202	204	238	240
09rbl.170	137	137	76	78	144	162	222	230	243	253	296	300	200	202	222	222
09rbl.171	137	137	76	76	156	168	224	230	249	249	298	298	200	212	238	240
09rbl.173	135	137	76	76	144	144	224	238	243	255	302	310	198	202	218	220
09rbl.186	137	137	76	78	144	144	224	234	239	241	296	308	200	202	224	230
09rbl.190	137	137	76	78	156	176	224	240	243	251	296	302	200	208	236	254
09rbl.203	135	137	76	76	156	158	232	240	253	253	294	294	200	202	214	246
09rbl.207	135	137	76	76	144	144	224	236	251	251	306	310	200	200	240	250
09rbl.209	135	137	74	76	144	162	230	238	249	251	294	296	200	202	222	222
09rbl.211	135	137	76	78	144	164	228	230	243	251	296	300	196	206	224	236
09rbl.214	137	137	76	76	144	158	222	222	241	243	298	310	198	200	236	236
09rbl.219	135	137	76	76	144	156	222	230	249	251	300	334	202	202	222	234
09rbl.222	137	137	76	76	144	156	224	232	251	251	274	294	160	160	240	242
09rbl.228	137	137	76	76	144	158	214	234	241	255	294	296	204	204	228	252
09rbl.232	135	137	76	76	144	188	222	262	241	245	298	298	200	200	220	220
09rbl.233	137	137	76	76	156	156	226	240	243	243	302	304	196	202	218	256
09rbl.234	137	137	76	76	156	164	220	230	251	253	300	306	198	202	220	234
09rbl.235	137	137	76	76	144	156	220	228	245	251	298	298	198	202	232	238
09rbl.244	137	137	76	78	144	144	220	234	251	255	298	324	200	202	234	234
09rbl.245	137	137	76	76	144	158	230	230	243	251	300	310	202	204	216	234
09rbl.248	135	137	76	76	144	156	220	224	249	251	298	302	196	202	234	234
09rbl.253	137	137	76	76	144	156	230	238	243	243	300	302	198	200	232	236
09rbl.254	137	137	76	78	144	156	224	228	243	251	298	300	196	200	222	240
09rbl.256	137	137	76	78	156	156	214	230	243	243	294	300	196	198	220	232
09rbl.266	137	137	76	76	152	158	232	250	251	255	304	304	196	204	192	240
09rbl.269	135	137	76	76	174	180	222	224	247	247	296	298	200	202	220	230
09rbl.270	135	137	76	76	144	156	238	242	245	253	298	298	198	200	220	224
09rbl.276	135	137	76	76	144	156	224	226	239	243	300	308	198	200	234	244
09rbl.284	137	137	76	76	156	184	224	232	249	251	300	302	204	206	192	244
09rbl.289	135	137	76	76	144	188	226	238	243	251	300	302	198	200	230	252
09rbl.295	135	137	76	76	144	152	226	234	237	249	302	302	198	200	228	234
09rbl.297	137	137	76	76	144	152	228	232	253	253	296	302	200	204	218	236
09rbl.299	137	137	76	78	154	164	232	236	247	249	296	296	160	206	192	228
09rbl.309	137	137	76	76	156	180	226	234	245	245	296	296	202	202	238	242

09rbl.314	135	137	76	78	144	144	234	236	243	243	300	300	200	200	212	218
09rbl.316	137	137	76	76	144	144	232	232	249	249	296	298	196	196	230	234
09rbl.319	135	137	76	76	160	188	224	228	243	253	296	302	198	200	220	232
09rbl.320	137	137	76	76	156	156	224	224	245	251	294	294	200	202	234	238
09rbl.321	135	135	74	76	144	180	228	252	245	249	300	300	198	198	220	238
09rbl.323	137	137	76	78	158	158	228	234	251	253	298	302	200	202	232	238
09rbl.327	137	137	76	76	144	156	226	232	251	255	292	298	200	200	218	220
09rbl.331	137	137	76	76	144	158	230	250	249	255	292	310	194	200	212	212
09rbl.333	137	137	76	76	144	154	224	224	251	251	298	304	200	202	224	250
09rbl.350	137	137	76	76	144	162	222	232	243	255	296	302	202	204	220	248
09rbl.364	135	137	72	76	156	158	222	224	241	253	298	302	196	196	218	252
09rbl.371	137	137	76	76	156	156	222	234	249	249	296	308	198	204	220	234
09rbl.372	137	137	76	76	144	156	230	238	249	255	294	306	204	208	230	238
09rbl.380	137	137	76	76	144	180	224	224	249	255	298	302	202	204	238	250
09rbl.385	135	135	76	76	144	158	230	236	253	253	296	302	198	200	234	238
09rbl.391	137	137	76	78	144	144	222	240	241	247	292	296	0	0	240	250
09rbl.394	135	135	76	78	144	144	226	228	239	243	296	298	202	204	220	220
09rbl.401	137	137	76	76	144	156	222	230	253	253	298	302	200	206	236	236
09rbl.406	137	137	76	76	144	152	230	240	251	255	294	302	200	200	192	236
09rbl.413	137	137	76	76	144	156	234	254	249	251	302	302	192	196	220	254
09rbl.415	137	137	76	76	144	152	214	230	243	255	300	304	204	206	192	238
09rbl.418	137	137	76	76	144	144	228	250	243	249	298	298	198	208	222	238
09rbl.420	137	137	76	76	144	156	228	230	249	251	296	300	200	202	234	236
09rbl.425	135	137	76	76	152	188	224	224	253	255	296	302	200	202	238	240
09rbl.434	137	137	76	76	160	180	220	234	249	251	298	302	198	206	214	214
09rbl.435	135	137	76	78	144	172	230	248	251	251	298	302	204	204	220	232
09rbl.440	137	137	76	76	144	144	230	236	249	251	298	304	198	204	222	222
09rbl.442	135	137	76	76	144	158	222	230	243	251	298	302	202	204	230	230
09rbl.447	135	135	76	76	144	160	230	238	251	255	296	312	196	196	218	222
09rbl.453	137	137	76	76	156	176	220	224	249	253	300	306	200	204	192	194
09rbl.455	137	137	76	76	144	156	230	238	245	253	302	304	202	206	234	240
09rbl.460	137	137	76	76	164	176	236	238	243	251	298	298	202	204	214	216
09rbl.462	135	137	76	76	144	144	220	260	251	251	302	302	202	202	220	236
09rbl.463	137	137	74	76	144	164	230	238	245	251	300	308	200	202	236	240
09rbl.466	135	135	76	76	144	146	224	240	243	253	296	296	204	206	240	250
09rbl.468	137	137	76	76	158	158	230	238	243	251	296	298	202	204	228	236
09rbl.476	135	137	76	76	144	144	230	248	247	253	296	306	198	206	212	232

09rbl.490	137	137	76	76	144	158	222	242	255	255	300	302	200	202	240	240
09rbl.494	137	137	76	76	156	158	212	220	243	253	302	302	200	200	242	256
09rbl.501	135	137	76	76	144	174	222	246	249	253	292	304	200	206	212	222
09rbl.503	137	137	76	76	150	180	230	238	243	249	302	304	202	206	228	236
09rbl.510	135	137	76	76	144	146	222	228	243	255	294	300	202	202	222	228
09rbl.511	135	137	76	76	144	158	222	224	243	251	294	306	200	202	234	238
09rbl.514	135	137	76	76	144	144	224	246	243	243	298	306	200	204	240	244
09rbl.516	135	137	76	76	154	158	228	230	249	251	294	310	200	206	224	240
09rbl.518	135	137	76	76	144	158	222	238	241	253	300	308	202	206	216	228
10rbl.001	137	137	76	76	156	160	230	242	249	255	296	296	196	206	222	226
10rbl.004	137	137	76	78	152	152	214	238	245	255	290	302	202	204	212	240
10rbl.006	137	137	76	78	144	144	214	236	253	255	300	300	198	210	236	240
10rbl.007	137	137	76	78	144	162	214	222	251	255	296	300	198	204	238	240
10rbl.008	137	137	76	78	144	144	214	214	249	255	296	302	198	200	220	238
10rbl.016	137	137	76	78	144	144	230	238	249	255	292	298	202	204	212	220
10rbl.017	137	137	76	78	162	162	214	222	251	253	296	308	202	202	192	238
10rbl.018	137	137	76	78	144	144	224	230	253	253	298	300	198	198	220	236
10rbl.019	137	137	76	76	156	156	230	236	243	253	296	300	196	202	224	232
10rbl.021	137	137	76	76	156	162	222	230	255	259	296	296	196	202	224	234
10rbl.028	137	137	72	78	144	156	232	240	251	251	298	304	202	202	230	234
10rbl.031	137	137	76	76	160	180	228	230	245	249	286	290	196	202	212	244
10rbl.035	137	137	72	76	168	188	246	246	249	249	294	310	198	202	218	234
10rbl.038	137	137	76	76	160	160	230	232	239	251	296	302	200	200	222	232
10rbl.049	137	137	72	76	156	168	224	230	241	253	300	300	202	206	236	244
10rbl.056	137	137	76	78	152	164	222	238	255	255	300	304	196	200	220	240
10rbl.058	135	137	76	76	144	156	222	222	245	249	304	304	200	200	214	238
10rbl.060	137	137	76	76	144	144	224	242	249	253	302	302	202	210	220	238
10rbl.064	137	137	76	76	144	144	230	236	243	243	298	302	200	204	212	240
10rbl.065	137	137	76	76	144	158	224	230	249	249	294	296	196	198	220	238
10rbl.066	137	137	76	76	144	144	224	234	241	249	294	302	196	204	220	236
10rbl.067	137	137	76	76	144	156	222	230	251	251	296	304	196	196	234	250
10rbl.081	137	137	76	76	156	160	222	242	249	249	296	306	196	202	230	236
10rbl.082	137	137	76	76	144	194	214	224	243	249	296	296	198	200	212	212
10rbl.083	137	137	76	76	158	182	222	238	241	251	302	316	160	196	226	236
10rbl.084	137	137	76	76	144	156	224	238	245	249	300	300	198	200	238	242
10rbl.088	137	137	76	76	144	158	224	224	251	259	296	302	160	202	214	238
10rbl.091	137	137	76	76	152	156	224	230	251	255	296	300	202	202	232	240

10rbl.095	137	137	76	76	144	144	220	222	251	251	298	298	202	202	236	238
10rbl.099	137	137	76	76	144	144	220	222	249	253	300	300	196	200	238	248
10rbl.112	137	137	76	76	164	164	220	230	245	249	302	302	198	200	194	236
10rbl.115	135	137	76	76	156	156	222	234	239	249	300	300	196	202	220	240
10rbl.117	137	137	76	78	144	144	230	256	249	253	296	302	200	202	212	236
10rbl.122	137	137	76	76	156	156	224	238	249	253	302	302	200	206	236	240
10rbl.131	137	137	76	76	144	152	220	222	243	255	298	302	160	202	234	234
10rbl.133	137	137	76	78	144	156	222	230	243	253	296	296	200	202	234	240
10rbl.139	137	137	76	76	156	184	224	224	243	251	298	302	200	202	222	236
10rbl.141	137	137	76	76	138	144	230	238	245	249	300	300	202	202	228	234
10rbl.144	137	137	72	76	156	156	238	240	249	253	296	302	200	200	212	234
10rbl.150	135	137	76	78	144	156	222	238	251	251	296	304	196	196	238	252
10rbl.151	135	137	76	76	144	144	220	224	239	249	294	300	200	200	192	234
10rbl.155	135	137	76	76	156	156	222	232	247	247	294	296	202	204	234	270
10rbl.156	137	137	76	76	144	162	230	250	249	249	292	310	200	202	222	226
10rbl.159	137	137	76	76	144	152	222	224	243	249	296	296	202	202	240	240
10rbl.161	137	137	76	76	144	176	224	224	243	253	300	300	180	200	218	250
10rbl.162	137	137	76	76	144	144	230	240	247	247	292	298	160	198	232	252
10rbl.166	137	137	76	78	144	170	220	230	249	251	296	296	200	202	226	226
10rbl.168	135	137	76	76	156	156	228	228	239	253	298	302	200	202	224	240
10rbl.171	137	137	76	76	144	158	230	236	243	249	298	310	202	202	224	234
10rbl.172	135	137	76	76	144	156	240	264	251	259	298	304	160	196	238	242
10rbl.179	135	135	76	76	144	144	222	238	249	249	302	302	202	210	218	252
10rbl.182	137	137	76	76	156	194	214	226	243	255	298	298	206	210	230	248
10rbl.192	137	137	76	76	144	156	212	230	251	253	286	300	200	206	250	250
10rbl.200	137	137	76	78	144	152	222	238	243	249	300	300	196	200	216	220
10rbl.203	135	135	76	78	144	144	234	238	253	253	298	298	200	202	232	244
10rbl.208	137	137	76	76	144	164	224	230	241	249	300	300	200	210	220	230
10rbl.214	137	137	76	78	144	156	222	230	253	253	300	320	196	204	226	250
10rbl.215	137	137	76	76	150	188	228	238	249	253	296	302	194	206	222	264
10rbl.216	137	137	76	76	144	144	222	230	249	249	298	302	200	204	212	220
10rbl.220	135	137	76	76	156	162	222	224	243	249	296	298	196	198	228	238
10rbl.221	137	137	76	76	144	144	236	236	249	249	294	294	196	210	222	238
10rbl.224	137	137	76	76	144	158	222	222	243	249	304	308	198	202	192	220
10rbl.225	137	137	76	76	152	156	224	224	253	253	298	308	196	200	218	238
10rbl.227	137	137	76	76	144	144	242	260	243	255	292	300	200	202	234	234
10rbl.232	137	137	76	76	144	156	230	238	243	243	300	302	202	204	234	238

10rbl.233	135	137	72	76	156	158	224	226	243	243	300	302	196	200	236	238
10rbl.238	135	137	74	76	144	158	226	230	243	249	296	298	200	200	238	248
10rbl.240	135	137	76	76	144	146	214	224	239	249	292	302	196	200	222	238
10rbl.241	135	137	76	76	144	162	230	238	249	249	300	310	180	200	234	234
10rbl.242	135	137	76	76	146	180	226	230	247	247	298	302	200	202	220	248
10rbl.244	135	137	76	76	144	180	228	230	249	249	298	300	198	202	220	234
10rbl.246	137	137	76	76	144	176	236	240	241	255	294	318	196	196	218	222
10rbl.253	137	137	76	78	144	188	232	240	245	249	292	294	198	204	238	240
10rbl.257	135	137	76	76	158	162	222	222	253	255	296	296	198	198	222	224
10rbl.258	137	137	76	76	144	156	224	230	243	253	298	298	204	206	232	236
10rbl.265	135	135	76	76	144	156	230	230	249	249	298	298	196	202	238	242
10rbl.267	137	137	76	76	144	144	224	230	243	243	296	302	202	202	232	238
10rbl.276	135	137	76	76	158	158	222	224	239	245	294	294	200	200	226	226
10rbl.278	135	137	76	76	156	162	230	230	243	251	300	302	200	202	220	242
10rbl.282	137	137	76	76	144	158	230	230	245	251	294	294	202	202	224	244
10rbl.283	137	137	76	76	144	144	224	240	251	251	296	302	200	202	218	218
10rbl.284	137	137	76	76	0	0	234	238	245	245	298	298	198	206	220	236
10rbl.288	137	137	76	76	144	144	224	230	251	259	302	302	202	202	220	236
10rbl.297	137	137	76	76	144	144	224	242	239	253	302	328	202	202	220	236
10rbl.311	137	137	76	76	146	188	222	228	239	249	292	292	202	204	234	234
10rbl.316	137	137	74	76	144	144	220	258	243	243	296	306	200	202	192	230
10rbl.321	137	137	76	78	162	188	220	228	249	249	292	300	198	202	234	252
10rbl.324	137	137	76	76	144	144	226	238	239	243	296	296	202	202	218	234
10rbl.325	137	137	72	76	144	156	230	230	245	253	300	300	196	202	198	234
10rbl.326	137	137	76	76	144	156	224	230	251	251	294	302	196	200	216	230
10rbl.327	137	137	76	76	144	144	222	230	251	257	292	300	160	200	220	254
10rbl.330	137	137	76	76	144	144	232	234	251	251	296	304	160	160	216	220
10rbl.339	137	137	76	76	144	156	224	250	251	255	298	302	196	198	234	234
10rbl.340	135	137	76	78	144	156	224	230	243	251	302	302	200	204	222	238
10rbl.348	137	137	76	76	144	156	230	240	243	249	296	296	200	200	240	248
10rbl.350	137	137	76	78	144	144	224	230	249	249	292	298	202	202	218	222
10rbl.352	137	137	76	76	144	152	224	246	251	251	296	304	196	202	194	250
10rbl.358	137	137	76	76	156	168	222	230	241	241	296	296	202	202	192	244
10rbl.360	135	135	76	76	144	144	222	236	249	249	288	302	200	202	232	238
10rbl.361	135	137	76	76	138	138	228	236	251	255	294	302	200	202	220	240
10rbl.363	137	137	76	76	144	158	212	246	241	253	302	302	200	202	236	238
10rbl.365	135	137	76	78	144	184	230	240	251	255	298	298	200	206	218	232

10rbl.366	135	137	76	76	144	156	230	234	243	243	296	296	204	206	234	234
10rbl.367	135	137	76	76	144	144	230	238	249	251	302	306	198	206	228	238
10rbl.376	135	135	76	76	144	156	224	230	239	251	292	300	160	206	240	248
10rbl.382	135	137	76	76	156	156	230	242	251	255	302	302	200	204	212	234
10rbl.387	137	137	76	76	144	156	214	224	239	253	296	298	200	202	234	234
10rbl.393	137	137	76	76	158	158	224	246	243	249	292	294	200	200	234	248
10rbl.398	137	137	76	76	158	158	224	230	239	255	296	300	200	200	212	250
10rbl.424	137	137	76	76	144	156	222	230	243	255	294	300	204	206	214	238
10rbl.425	137	137	76	76	144	156	214	224	243	253	292	292	196	202	236	248
10rbl.427	135	137	76	76	144	160	222	226	239	253	298	298	160	202	220	242
10rbl.438	135	137	76	78	144	164	222	238	241	249	296	296	202	202	214	238
10rbl.442	135	135	76	76	144	156	224	232	249	249	292	302	196	196	222	238
10rbl.444	135	135	76	76	156	158	228	238	249	249	296	298	202	202	234	236
10rbl.450	137	137	76	76	144	156	222	230	251	251	302	302	196	202	232	236
10rbl.453	137	137	76	76	144	182	226	240	241	249	296	296	200	204	234	236
10rbl.456	137	137	76	76	152	164	228	250	243	243	298	306	200	202	236	250
10rbl.477	135	137	76	76	158	158	226	240	243	243	298	298	196	200	226	236
10rbl.478	137	137	76	76	144	188	222	236	249	255	294	296	160	202	240	250
11rbl.002	137	137	76	76	144	144	226	226	241	243	294	298	196	204	218	226
11rbl.004	137	137	76	76	156	188	224	224	249	251	0	0	0	0	212	230
11rbl.011	137	137	76	76	144	158	222	264	239	243	294	318	200	202	228	238
11rbl.021	137	137	76	76	144	156	230	240	239	239	296	300	200	202	192	238
11rbl.022	135	137	76	76	144	144	226	252	249	253	294	308	196	202	214	248
11rbl.030	137	137	76	76	156	160	222	228	243	253	296	296	196	196	192	216
11rbl.033	137	137	76	76	144	184	212	224	243	251	296	306	198	200	222	238
11rbl.034	137	137	76	76	156	156	222	230	251	253	296	296	202	202	212	240
11rbl.035	135	137	72	76	144	156	230	246	251	251	300	306	202	202	246	260
11rbl.036	137	137	76	76	144	144	230	236	243	255	294	300	160	180	240	244
11rbl.040	137	137	76	76	144	144	222	242	249	255	298	302	200	202	228	238
11rbl.041	137	137	76	76	156	160	230	230	245	249	296	310	200	200	240	240
11rbl.045	137	137	76	76	144	156	222	230	249	251	298	302	204	204	238	260
11rbl.046	137	137	76	76	156	160	222	230	245	253	296	306	196	204	248	256
11rbl.049	135	137	76	76	152	162	222	222	241	249	296	296	198	202	224	232
11rbl.055	137	137	76	76	144	156	230	230	243	249	292	300	196	210	246	254
11rbl.060	137	137	76	76	152	164	214	224	249	253	302	302	204	206	212	236
11rbl.064	135	137	76	76	144	156	222	236	249	249	294	300	196	204	220	220
11rbl.067	137	137	76	76	144	174	230	238	239	249	298	302	194	202	226	250

11rbl.076	137	137	76	76	144	156	220	240	251	259	296	302	200	206	220	250
11rbl.077	137	137	76	78	144	156	224	230	241	243	292	296	196	200	222	240
11rbl.086	137	137	76	76	144	156	224	230	251	255	296	300	200	200	240	240
11rbl.087	137	137	76	76	144	156	222	238	241	243	298	308	200	206	234	252
11rbl.094	137	137	76	76	144	144	230	240	251	253	298	298	196	208	238	252
11rbl.101	137	137	76	76	156	156	226	230	251	251	288	294	196	202	230	234
11rbl.105	135	137	76	76	144	144	230	246	241	251	302	310	200	202	216	240
11rbl.115	135	137	76	76	144	158	228	228	249	253	292	296	202	202	238	262
11rbl.119	135	137	76	76	144	156	222	224	251	251	294	294	200	206	200	238
11rbl.138	137	137	76	76	144	144	238	238	253	259	302	302	196	202	224	240
11rbl.147	137	137	76	78	144	144	222	230	245	249	300	304	196	200	212	238
11rbl.151	137	137	76	76	144	144	224	236	249	249	300	302	196	202	222	230
11rbl.158	137	137	74	76	150	156	212	224	249	251	296	296	202	206	240	240
11rbl.159	137	137	76	76	144	188	238	240	249	255	300	300	200	202	240	254
11rbl.163	135	137	76	76	144	144	236	238	243	259	310	318	200	204	214	250
11rbl.164	137	137	76	76	144	156	222	230	243	253	296	310	200	202	230	254
11rbl.171	137	137	76	76	144	156	230	230	249	253	296	300	200	204	220	234
11rbl.176	137	137	76	78	144	174	228	236	251	255	302	302	200	206	216	238
11rbl.178	135	137	76	76	160	160	230	240	243	259	292	302	160	202	218	234
11rbl.182	137	137	76	76	144	156	222	248	251	255	302	304	196	202	234	234
11rbl.185	135	137	76	76	158	158	222	230	245	249	296	302	202	210	236	240
11rbl.187	135	137	76	76	144	152	226	244	243	251	296	302	200	202	234	238
11rbl.189	135	135	76	78	156	156	230	240	239	255	294	298	200	206	194	236
11rbl.194	137	137	76	76	144	174	224	224	245	253	300	304	198	200	226	240
11rbl.195	137	137	76	76	144	164	212	222	243	251	294	304	200	200	218	240
11rbl.211	137	137	76	76	144	156	224	240	239	255	296	300	196	198	238	244
11rbl.212	137	137	76	76	144	156	222	240	241	249	294	294	198	200	236	238
11rbl.213	137	137	76	76	156	162	226	234	251	255	294	302	160	204	196	220
11rbl.215	135	137	76	76	156	158	224	238	253	259	294	302	196	200	220	232
11rbl.216	137	137	76	76	152	198	224	240	249	251	296	296	198	204	234	234
11rbl.217	137	137	76	76	156	164	222	238	239	249	296	302	160	198	220	230
11rbl.223	137	137	76	76	144	156	214	230	253	253	296	300	194	210	236	242
11rbl.225	137	137	76	76	156	164	222	230	241	243	296	302	200	202	214	224
11rbl.230	135	137	76	76	144	156	224	232	243	255	296	310	202	202	212	232
11rbl.233	135	137	76	76	144	184	226	240	239	253	300	300	202	206	220	236
11rbl.238	135	137	76	76	144	198	230	230	249	249	296	302	200	202	214	218
11rbl.245	135	135	76	76	144	182	226	246	249	251	296	302	180	198	222	222

11rbl.247	137	137	76	76	144	164	230	248	251	253	298	298	160	196	220	232
11rbl.252	135	137	76	76	144	156	222	246	249	255	296	304	196	202	220	242
11rbl.261	135	137	76	76	144	144	230	230	249	259	296	300	196	214	216	228
11rbl.263	135	137	76	78	144	176	230	260	243	243	300	300	200	210	220	234
11rbl.265	137	137	76	94	144	144	222	244	251	253	296	302	196	202	238	238
11rbl.271	135	137	76	76	144	156	238	240	239	249	296	300	180	200	220	234
11rbl.282	135	137	76	76	144	144	230	246	249	251	302	310	200	202	246	258
11rbl.287	137	137	76	76	172	192	222	222	249	253	300	300	160	196	240	248
11rbl.290	137	137	76	76	144	144	228	232	249	251	304	304	198	198	220	240
11rbl.293	135	137	76	76	144	156	226	260	243	243	292	302	202	206	234	234
11rbl.299	135	137	76	76	156	164	230	238	251	257	298	298	204	210	232	246
11rbl.302	135	137	76	76	144	160	232	252	249	251	298	300	196	200	226	252
11rbl.303	135	137	76	76	144	156	222	250	243	251	286	298	200	202	220	236
11rbl.309	137	137	76	76	156	176	224	224	251	253	296	300	200	204	212	240
11rbl.310	137	137	76	76	144	144	214	238	251	253	292	302	200	202	192	236
11rbl.312	135	137	76	76	144	144	230	244	243	253	292	298	196	202	220	236
11rbl.313	137	137	76	78	156	162	224	236	245	257	296	298	196	210	222	254
11rbl.317	137	137	76	76	144	144	230	234	243	251	292	298	202	202	228	234
11rbl.318	137	137	76	78	144	146	214	224	243	255	292	296	202	206	220	236
11rbl.320	137	137	76	76	144	156	230	248	245	249	294	300	202	206	192	234
11rbl.321	137	137	76	78	144	156	214	222	243	251	274	300	200	200	234	240
11rbl.331	137	137	76	76	156	176	230	242	253	255	306	310	196	202	220	236
11rbl.338	135	137	76	76	144	188	226	248	251	261	298	298	196	196	234	234
11rbl.341	137	137	76	76	144	180	234	238	243	249	292	306	202	202	234	250
11rbl.345	137	137	76	76	144	144	224	224	239	241	296	302	196	200	218	238
11rbl.346	135	137	76	76	146	152	220	228	253	253	294	302	202	202	218	238
11rbl.352	137	137	76	76	156	156	236	246	249	251	302	302	202	202	220	234
11rbl.354	137	137	76	76	144	162	222	224	251	253	296	300	196	200	234	258
11rbl.356	137	137	76	76	152	184	228	230	245	249	300	300	200	202	200	236
11rbl.360	137	137	76	78	144	176	230	232	249	251	296	302	196	196	220	250
11rbl.361	137	137	76	76	144	156	224	230	243	249	298	302	180	196	220	262
11rbl.367	137	137	76	76	144	144	224	238	259	259	294	310	196	200	220	252
11rbl.368	135	137	76	76	144	144	224	230	255	259	300	300	196	200	226	242
11rbl.375	135	137	76	76	144	144	224	230	249	251	298	304	200	202	222	228
11rbl.376	135	135	76	76	144	146	212	246	249	251	302	302	160	204	216	240
11rbl.390	137	137	76	78	144	156	228	246	245	249	302	302	200	206	212	230
11rbl.400	137	137	72	76	150	156	212	220	245	251	298	298	200	200	192	212

11rbl.404	135	137	76	76	144	174	224	254	249	249	310	310	200	200	222	234
11rbl.406	135	137	76	76	158	158	228	240	251	253	296	296	192	200	236	240
11rbl.411	137	137	76	76	144	144	224	226	249	255	298	298	198	202	228	230
11rbl.415	137	137	76	76	156	156	222	238	249	253	300	300	196	196	218	238
11rbl.421	135	137	76	76	158	180	222	260	249	249	302	302	200	202	216	234
11rbl.422	135	137	76	76	144	156	222	230	249	259	292	298	192	204	240	252
11rbl.429	137	137	72	76	144	144	224	240	249	255	300	304	160	206	220	234
11rbl.436	135	137	76	76	156	176	220	230	243	255	296	306	196	200	222	236
11rbl.441	137	137	76	78	144	158	222	226	243	259	302	302	196	204	220	236
11rbl.442	137	137	76	76	158	184	224	228	243	249	302	302	202	202	234	236
11rbl.448	137	137	76	94	156	156	230	238	251	253	300	300	196	206	226	238
11rbl.451	137	137	76	76	146	156	222	238	249	251	294	300	196	206	220	226
11rbl.452	137	137	76	76	144	194	224	230	249	253	302	306	200	206	240	240
11rbl.454	137	137	76	76	144	152	230	246	249	249	300	306	180	200	192	236
11rbl.463	137	137	76	76	0	0	224	240	243	249	304	308	178	200	238	238
11rbl.466	135	137	76	76	156	164	222	238	241	253	308	308	200	204	218	220
11rbl.469	135	137	76	76	150	166	220	230	243	259	300	302	200	200	222	250
11rbl.482	137	137	76	76	144	158	224	230	241	253	302	318	202	204	212	270
11rbl.491	135	137	76	76	144	158	224	226	243	251	296	304	198	202	234	238
11rbl.501	137	137	76	78	156	156	224	230	243	249	294	304	200	202	224	238
11rbl.502	137	137	76	78	144	144	234	238	249	251	302	302	202	204	212	232
11rbl.507	137	137	74	76	144	144	238	238	243	255	294	304	196	200	214	240
11rbl.510	137	137	76	76	144	156	212	230	255	255	300	302	200	200	192	232
11rbl.516	137	137	76	76	144	152	224	246	243	251	298	304	196	202	234	252
11rbl.521	137	137	76	76	144	144	214	220	243	245	298	300	196	200	212	254
11rbl.522	135	137	76	76	144	176	222	222	241	241	294	300	196	206	220	224
11rbl.529	137	137	72	76	144	144	222	230	243	243	302	302	202	206	234	238

Sample ID	Xte17	Xte17	Xte18	Xte18	Xte19	Xte19	Xte20	Xte20	Xte22	Xte22	Xte24	Xte24	Xte25	Xte25
97rbl.002	214	232	282	290	210	220	274	274	174	204	272	284	110	110
97rbl.008	216	224	290	290	202	218	298	308	160	160	266	270	110	110
97rbl.009	226	226	282	282	218	222	298	298	188	196	286	298	110	110
97rbl.010	224	224	282	286	202	220	280	280	150	168	274	274	110	112
97rbl.011	212	224	282	288	214	218	280	298	174	186	274	288	110	110
97rbl.018	214	254	282	290	212	214	284	298	174	174	266	288	110	112
97rbl.021	214	222	292	292	214	214	268	298	174	174	268	286	110	112
97rbl.029	214	218	282	292	214	216	282	296	170	170	262	302	110	110
97rbl.031	224	226	290	290	216	222	284	284	192	212	262	284	110	110
97rbl.032	214	214	286	290	214	218	278	298	188	202	276	298	110	110
97rbl.034	224	234	290	290	202	218	270	294	194	200	272	306	110	110
97rbl.040	224	230	284	286	206	216	280	308	166	204	276	282	110	110
97rbl.041	222	230	282	282	212	214	254	294	174	192	282	288	110	112
97rbl.043	222	226	288	294	218	218	306	308	170	202	282	292	110	110
97rbl.045	216	216	286	290	214	214	256	272	166	168	288	298	110	112
97rbl.048	228	250	286	286	212	214	220	270	206	208	276	288	110	110
97rbl.051	224	226	288	292	214	232	272	300	200	200	284	294	110	112
97rbl.055	212	224	282	282	214	214	294	296	168	212	284	288	110	112
97rbl.062	224	226	286	290	214	216	280	304	168	168	284	300	110	110
97rbl.068	224	238	282	286	212	216	292	292	182	182	288	294	110	112
97rbl.070	224	234	0	0	216	218	268	270	150	166	292	296	110	110
97rbl.072	212	224	282	290	210	214	252	288	170	170	270	286	110	110
97rbl.076	212	224	282	286	214	218	270	300	150	170	288	290	110	110
97rbl.077	222	226	282	284	214	214	268	282	182	182	276	288	110	110
97rbl.080	214	248	282	284	210	216	272	272	210	210	264	270	110	110
97rbl.082	212	218	286	290	202	214	288	296	198	214	278	296	110	110
97rbl.084	214	222	282	286	214	214	254	298	180	180	294	298	110	110
97rbl.085	238	244	288	292	204	228	276	300	170	172	264	270	110	110
97rbl.095	218	224	282	284	214	230	280	280	150	150	268	292	110	110
97rbl.102	214	222	282	284	214	214	280	296	188	188	268	274	110	110
97rbl.105	226	226	282	286	214	214	272	276	170	170	286	292	114	114
97rbl.106	214	244	282	282	208	218	270	294	194	194	264	282	110	110
97rbl.107	214	214	280	284	204	204	288	288	168	168	286	298	110	112
97rbl.108	226	226	288	290	216	224	258	282	204	206	280	294	110	110
97rbl.110	214	224	282	290	210	218	264	294	168	168	286	298	110	114
97rbl.111	214	224	286	290	212	236	268	274	196	196	290	300	110	112

97rbl.112	212	224	290	290	202	208	286	298	192	210	280	296	112	112
97rbl.113	224	226	292	292	214	222	304	322	200	202	284	302	110	110
97rbl.117	220	222	282	292	212	222	290	290	202	204	284	292	110	110
97rbl.120	214	218	286	286	216	222	256	288	168	194	278	294	110	112
97rbl.122	224	224	284	290	214	218	282	292	204	218	282	294	110	110
97rbl.125	244	252	284	286	214	218	296	298	204	208	292	294	110	110
97rbl.130	226	228	292	294	214	216	252	296	178	178	278	300	110	110
97rbl.132	226	246	284	284	214	222	282	298	172	190	288	294	110	112
97rbl.133	224	224	290	290	212	218	264	304	194	194	282	284	110	114
97rbl.138	226	226	282	284	210	230	296	300	210	210	274	284	110	114
97rbl.141	214	224	282	282	208	218	284	298	180	208	266	284	106	110
97rbl.143	220	226	282	286	210	210	270	276	150	166	262	290	110	110
97rbl.144	220	246	282	286	218	226	272	298	174	216	300	300	110	110
97rbl.145	220	226	282	282	218	220	298	304	166	194	280	298	110	112
97rbl.146	224	244	284	284	212	222	254	284	150	150	286	300	110	110
97rbl.150	218	222	282	286	216	218	284	292	194	216	284	298	110	110
97rbl.160	226	232	286	292	218	218	252	284	178	198	286	290	110	110
97rbl.163	224	228	284	284	202	218	292	294	150	150	278	290	110	110
97rbl.166	214	224	288	290	214	218	258	286	150	150	284	286	110	112
97rbl.168	222	226	286	290	214	218	290	304	150	156	284	292	110	114
97rbl.169	216	220	282	286	214	218	266	272	166	188	292	296	110	110
97rbl.170	226	230	290	290	208	210	286	286	168	208	284	296	110	110
97rbl.174	216	228	290	290	214	222	292	304	224	224	268	288	110	110
97rbl.175	212	232	290	290	210	210	270	272	170	216	290	296	110	110
97rbl.177	226	228	282	282	214	222	298	304	170	228	268	276	110	110
97rbl.185	224	228	286	290	214	222	286	290	168	188	284	284	110	110
97rbl.187	212	226	286	290	202	220	272	298	210	210	278	296	110	110
97rbl.193	212	224	286	286	208	214	288	294	170	210	276	294	110	110
97rbl.194	224	226	282	288	214	220	252	270	150	204	268	300	110	114
97rbl.195	222	226	282	288	212	214	284	306	150	190	282	282	110	112
97rbl.196	226	228	282	282	208	214	274	302	150	216	288	296	110	110
97rbl.199	222	226	284	290	210	212	292	292	174	226	268	302	110	110
97rbl.200	212	224	282	290	210	214	272	304	218	218	284	290	110	112
97rbl.208	214	224	290	290	218	222	282	300	168	170	286	292	110	110
97rbl.209	216	222	290	290	202	232	286	300	172	172	274	278	110	112
97rbl.211	214	224	282	286	212	232	272	294	150	172	274	276	110	112
97rbl.213	216	224	292	292	212	232	272	292	198	214	270	270	110	112

97rbl.215	224	226	286	290	214	232	292	292	172	172	288	300	110	110
97rbl.216	222	224	280	290	202	208	280	284	168	194	274	274	110	110
97rbl.221	222	224	280	290	202	208	280	284	168	214	276	276	110	110
97rbl.224	228	256	280	290	212	218	282	282	186	202	274	278	110	110
97rbl.226	214	224	284	286	222	232	290	300	170	188	270	290	112	112
97rbl.228	224	228	282	290	212	222	292	300	150	182	288	290	110	112
97rbl.232	224	224	284	292	216	220	306	306	160	184	278	284	110	110
97rbl.235	218	224	282	290	210	218	292	296	202	204	284	288	110	110
97rbl.237	212	224	280	290	216	218	286	286	190	202	284	284	110	110
97rbl.238	228	246	282	286	218	234	286	286	184	190	282	282	110	112
97rbl.239	218	232	282	284	212	216	294	296	170	204	278	278	110	110
97rbl.241	212	242	282	284	218	220	286	286	188	190	286	286	110	112
97rbl.243	212	226	286	286	206	226	220	292	198	198	298	306	110	112
97rbl.246	214	224	286	292	212	218	292	296	150	150	274	280	110	110
97rbl.251	224	224	286	288	210	216	288	290	178	182	278	278	110	110
97rbl.252	214	228	288	290	220	220	286	290	150	170	278	278	110	110
97rbl.255	224	228	290	290	208	212	280	308	186	188	296	298	110	110
97rbl.260	214	222	288	290	210	214	288	294	166	182	286	286	110	110
97rbl.269	214	214	282	290	210	214	264	264	172	172	280	282	110	110
97rbl.272	222	228	284	290	212	212	272	274	190	190	280	296	110	114
97rbl.280	224	226	282	282	202	208	272	288	174	174	274	284	112	112
97rbl.283	232	232	284	284	210	218	302	306	168	182	282	288	110	110
97rbl.289	214	224	286	290	208	232	290	298	170	192	290	290	110	110
97rbl.294	224	224	282	290	214	214	298	308	150	176	266	288	110	110
97rbl.295	212	232	282	288	216	220	272	308	202	222	276	298	110	110
97rbl.297	222	232	282	292	202	218	268	304	150	172	284	284	110	110
97rbl.303	214	224	282	282	202	214	254	306	196	196	284	284	110	110
97rbl.305	228	242	280	284	202	218	288	298	198	210	294	298	110	110
97rbl.308	224	224	282	286	212	214	268	268	150	176	268	300	110	112
97rbl.312	224	224	282	292	210	212	272	286	168	200	262	294	110	110
97rbl.318	226	226	282	286	214	214	270	282	206	218	284	294	110	110
97rbl.324	224	226	282	286	214	216	270	296	176	184	290	300	110	110
97rbl.328	218	224	282	290	214	218	284	310	194	204	292	298	110	112
97rbl.331	212	224	282	282	212	222	292	302	176	200	280	302	110	110
97rbl.333	218	226	284	290	214	214	288	308	168	170	262	286	110	112
97rbl.335	214	228	282	286	202	218	272	274	172	202	282	296	110	112
97rbl.336	214	246	290	298	208	214	280	286	202	202	280	284	110	112

97rbl.339	224	236	284	286	210	218	270	292	170	192	286	290	112	112
97rbl.347	224	224	282	286	216	218	272	298	180	216	276	284	110	114
97rbl.349	220	224	282	290	202	214	278	290	0	0	300	300	110	112
97rbl.351	218	218	286	290	210	214	290	302	168	170	280	292	110	112
97rbl.357	224	226	282	282	212	214	270	290	166	174	280	292	110	112
97rbl.359	226	250	282	290	210	222	280	282	150	162	268	280	110	110
97rbl.364	226	228	282	286	214	214	284	288	168	198	274	274	110	112
97rbl.365	212	218	288	290	210	222	284	290	206	206	270	286	110	112
97rbl.368	214	224	282	290	214	218	258	298	150	150	284	288	110	110
97rbl.370	212	214	286	286	208	222	292	292	170	196	282	288	110	116
98rbl.001	224	228	282	290	202	216	276	300	208	208	276	280	110	110
98rbl.002	224	226	286	286	210	214	272	294	192	200	264	286	110	110
98rbl.007	228	228	282	290	210	212	274	292	174	200	276	306	110	110
98rbl.008	214	222	282	286	208	212	298	306	192	196	274	274	110	110
98rbl.010	214	238	282	282	212	218	278	292	168	218	284	288	110	110
98rbl.020	232	252	282	282	214	214	276	280	188	192	280	280	112	114
98rbl.021	230	232	286	290	210	216	280	312	190	192	288	300	110	112
98rbl.026	224	250	284	288	214	232	284	290	174	178	292	292	110	110
98rbl.031	224	232	282	284	212	214	252	294	204	204	284	290	110	110
98rbl.034	212	220	286	286	208	222	284	292	202	202	278	284	110	112
98rbl.041	212	216	284	290	216	216	268	286	150	176	292	302	110	110
98rbl.042	220	230	282	282	214	220	270	300	150	176	274	296	110	112
98rbl.045	230	230	284	288	212	214	274	312	166	202	292	294	110	110
98rbl.048	216	224	282	294	212	214	284	294	150	150	290	298	110	110
98rbl.049	222	232	284	292	208	228	286	292	150	202	284	296	110	110
98rbl.066	218	222	282	282	212	212	244	246	190	216	288	298	110	112
98rbl.072	224	254	284	286	212	214	286	294	190	196	278	284	112	114
98rbl.073	214	216	282	292	210	222	308	310	150	216	288	288	110	112
98rbl.082	222	222	282	288	212	214	270	288	192	192	266	286	110	110
98rbl.086	214	222	282	286	212	236	254	254	150	194	284	296	110	110
98rbl.088	220	220	282	282	214	216	266	298	172	192	286	300	110	110
98rbl.090	224	238	282	288	204	210	264	278	170	192	284	290	110	110
98rbl.098	222	244	280	284	212	214	272	288	168	188	286	290	110	110
98rbl.100	226	244	282	292	210	214	256	278	166	176	288	300	110	110
98rbl.102	214	224	284	290	212	214	286	290	168	170	272	288	110	110
98rbl.104	224	232	282	292	214	218	272	286	168	168	282	286	110	110
98rbl.114	214	224	280	282	212	214	286	290	168	188	268	268	110	110

98rbl.120	222	224	284	286	210	222	272	272	214	226	286	290	110	110
98rbl.122	232	252	282	290	208	214	252	274	168	168	278	286	110	112
98rbl.127	216	224	282	290	218	222	272	272	150	170	296	298	110	112
98rbl.128	220	226	286	288	208	230	290	300	192	202	282	286	112	114
98rbl.131	224	232	282	282	208	222	254	286	168	192	280	284	110	110
98rbl.137	212	224	282	288	214	222	280	290	190	208	300	304	110	114
98rbl.140	226	254	284	284	214	214	274	282	168	192	280	290	110	112
98rbl.150	216	226	286	294	210	218	252	308	208	218	262	270	110	114
98rbl.151	214	222	282	284	202	214	272	304	196	202	282	294	110	110
98rbl.153	226	230	284	288	210	212	286	304	192	196	276	294	110	112
98rbl.156	220	220	284	286	214	214	270	290	204	204	290	294	110	112
98rbl.158	216	216	286	290	214	216	286	286	170	226	296	298	110	112
98rbl.160	216	220	282	290	214	214	272	290	178	178	270	290	110	112
98rbl.164	214	218	282	284	214	214	270	296	176	176	270	296	110	112
98rbl.165	214	226	284	290	202	218	286	312	196	200	276	282	112	114
98rbl.169	218	226	282	290	216	218	252	290	166	200	262	282	110	110
98rbl.173	218	226	282	286	214	216	252	290	166	182	262	284	110	110
98rbl.175	216	228	282	282	214	214	262	280	200	202	278	288	110	110
98rbl.183	212	216	288	292	210	214	250	290	182	198	280	302	110	110
98rbl.188	228	228	284	292	222	232	266	308	192	220	292	296	110	110
98rbl.190	226	226	284	300	208	218	276	292	224	224	266	288	110	110
98rbl.194	214	218	290	290	208	214	286	294	172	208	280	288	110	112
98rbl.196	216	224	292	292	208	214	284	298	170	176	268	288	110	110
98rbl.197	222	230	280	288	214	222	256	308	150	176	284	284	110	110
98rbl.205	224	224	282	288	208	214	266	290	198	208	280	290	110	110
98rbl.207	226	232	282	294	212	222	282	290	168	188	272	290	110	110
98rbl.215	222	224	282	292	202	208	292	302	192	202	262	284	110	114
98rbl.218	212	214	282	292	208	214	290	294	202	202	284	284	110	112
98rbl.219	224	252	288	288	216	222	272	274	168	208	288	288	110	110
98rbl.221	222	226	282	288	210	210	286	302	170	202	280	286	110	110
98rbl.224	214	216	290	290	214	214	272	288	162	204	274	302	110	110
98rbl.228	224	226	282	288	210	214	274	292	172	188	288	290	110	112
98rbl.233	222	226	282	290	218	222	274	286	150	166	274	286	110	110
98rbl.235	222	222	282	288	204	208	300	308	174	204	286	298	110	110
98rbl.244	214	218	290	290	204	214	290	312	166	224	284	290	110	110
98rbl.245	222	224	282	290	214	216	260	272	198	202	286	288	110	110
98rbl.249	214	228	286	290	218	220	300	310	204	204	268	296	110	110

98rbl.252	222	226	282	286	212	218	266	298	216	222	286	296	110	112
98rbl.254	214	220	282	290	218	226	286	286	210	210	266	278	112	112
98rbl.261	212	252	286	290	212	214	286	294	170	202	288	292	110	110
98rbl.267	218	224	282	282	206	208	292	298	170	176	286	286	110	110
98rbl.271	214	226	286	290	208	218	274	290	0	0	280	290	110	114
98rbl.274	222	228	282	286	210	216	282	290	170	178	288	290	110	112
98rbl.276	214	224	286	290	210	212	282	290	194	208	288	288	110	112
98rbl.282	222	224	282	282	206	214	300	304	180	190	280	284	110	112
98rbl.286	222	224	282	282	204	230	290	304	150	192	270	282	110	112
98rbl.289	214	214	290	290	214	216	294	304	168	206	266	290	110	110
98rbl.293	214	224	290	290	214	214	300	304	166	206	266	280	110	110
98rbl.298	226	234	284	284	204	208	274	284	202	214	284	288	110	110
98rbl.302	222	232	282	282	208	218	272	280	150	174	280	290	110	110
98rbl.309	214	214	286	288	210	216	290	308	176	222	288	288	110	110
98rbl.310	214	226	284	286	206	214	276	282	162	198	282	290	110	110
98rbl.311	214	230	284	286	206	214	0	0	170	170	280	284	110	110
98rbl.323	214	224	290	290	210	216	280	312	150	150	276	278	110	116
98rbl.326	224	228	288	290	214	216	282	284	190	204	286	302	110	112
98rbl.327	222	226	284	286	212	222	270	276	176	196	284	298	110	112
98rbl.335	226	236	282	290	210	218	274	290	190	222	296	296	110	112
98rbl.342	228	236	286	288	212	214	284	290	168	192	286	286	110	112
98rbl.346	214	218	286	288	218	222	298	310	0	0	268	296	110	110
98rbl.357	224	228	282	290	212	218	264	282	172	174	284	290	110	110
98rbl.361	212	220	290	292	214	218	270	294	192	194	272	290	110	110
98rbl.363	224	246	286	286	214	214	264	268	172	196	268	290	110	112
98rbl.369	224	248	282	290	214	214	272	298	198	198	288	292	110	110
98rbl.372	212	226	282	290	204	216	272	280	170	174	272	300	110	112
98rbl.374	222	236	282	290	210	230	264	282	170	172	288	288	110	110
98rbl.375	212	214	282	288	214	214	286	288	190	214	290	292	110	112
98rbl.380	222	224	282	290	204	218	294	296	170	214	262	286	110	110
98rbl.388	222	224	282	290	214	214	272	286	190	190	288	298	110	112
98rbl.391	214	224	284	288	204	214	280	302	172	182	272	290	110	114
98rbl.392	222	250	282	286	210	212	282	292	168	202	280	296	110	112
98rbl.393	224	248	286	290	210	214	272	272	200	210	280	298	110	110
98rbl.395	232	232	282	282	210	214	286	296	192	200	266	292	110	112
98rbl.396	224	226	282	286	210	218	262	298	166	182	284	286	110	112
98rbl.397	226	232	282	290	216	220	296	298	188	188	284	294	110	110

98rbl.400	214	218	282	286	204	214	286	296	182	200	284	292	110	112
98rbl.402	222	236	282	290	218	218	300	300	222	222	284	288	110	110
98rbl.403	214	222	282	290	214	214	272	298	168	212	274	282	110	110
98rbl.408	224	226	282	282	210	214	258	292	212	212	272	278	110	110
98rbl.410	222	224	282	290	216	218	284	292	190	216	276	284	110	110
98rbl.417	218	224	284	290	212	214	270	290	170	174	292	304	110	110
98rbl.420	214	214	282	292	210	210	268	282	150	188	276	282	110	110
98rbl.428	212	218	284	286	212	214	292	292	170	188	282	300	110	110
98rbl.431	224	226	282	282	218	222	286	296	184	210	282	282	110	112
98rbl.434	226	226	284	290	208	218	274	284	216	218	276	296	110	110
98rbl.437	218	226	282	286	212	212	290	300	172	208	274	304	110	112
98rbl.439	224	224	282	286	214	214	272	298	210	210	278	296	110	110
98rbl.440	224	230	284	290	204	212	274	280	178	194	280	298	110	110
98rbl.456	222	224	282	286	204	214	264	266	150	196	286	290	110	110
98rbl.458	214	232	282	286	208	214	272	272	206	206	286	286	110	110
98rbl.464	224	224	282	284	208	218	280	282	166	170	284	294	110	110
98rbl.474	222	224	284	286	206	210	272	288	170	170	288	288	110	112
98rbl.477	224	224	286	290	210	212	292	300	210	212	268	280	110	110
98rbl.488	224	228	282	290	214	214	260	268	192	216	288	290	110	110
99rbl.005	212	232	282	286	210	218	266	300	170	170	262	288	110	110
99rbl.007	228	228	282	290	204	212	248	292	194	194	280	284	110	110
99rbl.008	226	226	282	288	210	218	280	288	170	202	274	288	110	110
99rbl.012	214	232	282	284	216	236	256	270	170	204	286	294	110	110
99rbl.019	212	224	286	290	212	222	302	302	200	204	288	298	110	110
99rbl.023	214	222	286	286	214	236	278	292	196	196	274	294	110	110
99rbl.025	226	226	284	290	216	220	268	286	174	174	282	298	110	110
99rbl.026	218	224	282	286	216	218	254	292	196	208	290	294	110	110
99rbl.027	218	244	290	290	218	218	252	300	208	230	280	288	110	110
99rbl.029	222	232	284	286	210	212	270	280	170	198	274	284	110	110
99rbl.031	218	224	284	286	218	222	280	312	166	172	272	296	110	110
99rbl.034	232	238	286	286	204	212	296	302	188	192	276	288	110	112
99rbl.036	214	224	290	290	208	218	256	292	192	216	270	276	110	110
99rbl.037	228	232	282	290	214	218	300	304	170	196	292	304	110	110
99rbl.041	222	226	282	286	208	236	272	306	176	196	294	294	110	110
99rbl.044	222	226	284	284	208	236	272	292	170	176	288	296	110	110
99rbl.046	214	224	286	290	208	236	252	256	192	196	276	288	110	110
99rbl.050	212	254	282	290	204	218	264	286	196	200	278	280	110	110

99rbl.051	216	226	284	290	214	218	290	296	174	176	266	290	110	112
99rbl.053	212	224	282	290	214	218	266	292	196	196	270	270	110	110
99rbl.054	214	222	286	290	208	236	252	256	192	216	270	276	110	110
99rbl.058	220	228	288	288	216	218	286	298	192	206	284	286	110	110
99rbl.060	224	224	286	290	216	216	286	296	176	190	262	274	110	110
99rbl.063	214	218	282	288	214	214	270	296	190	190	266	266	110	114
99rbl.066	222	222	282	286	208	226	254	286	150	150	272	288	112	114
99rbl.067	226	230	282	282	210	214	270	298	190	190	284	302	110	114
99rbl.068	222	248	286	286	214	216	292	318	170	170	272	284	110	112
99rbl.069	212	224	284	286	214	222	290	292	208	208	282	288	110	112
99rbl.072	212	220	282	282	212	216	292	296	170	214	286	298	110	110
99rbl.075	224	224	282	286	214	218	284	284	188	192	294	298	110	110
99rbl.076	214	214	282	290	214	214	282	282	194	202	282	282	110	110
99rbl.077	222	224	286	292	204	214	254	306	172	192	284	300	110	110
99rbl.078	222	236	280	286	208	212	260	276	170	202	284	288	110	110
99rbl.079	218	224	284	288	214	220	272	294	172	178	274	288	110	110
99rbl.083	224	228	282	282	208	218	290	290	168	168	288	288	112	112
99rbl.084	224	228	290	292	210	214	272	296	200	222	262	292	110	112
99rbl.088	224	226	282	290	212	222	294	310	150	184	274	288	112	114
99rbl.089	214	238	282	282	202	208	252	294	168	198	280	300	110	110
99rbl.090	214	232	286	290	222	232	272	296	170	192	274	292	110	110
99rbl.092	214	224	282	290	212	222	296	298	196	196	288	288	110	110
99rbl.098	214	228	286	286	222	222	268	294	150	168	288	288	110	112
99rbl.099	214	228	282	290	208	214	290	298	168	170	268	280	112	112
99rbl.104	224	236	284	290	212	214	262	268	168	168	288	296	110	112
99rbl.111	222	224	288	288	214	218	264	286	190	196	286	294	110	110
99rbl.112	232	248	282	286	202	214	246	270	170	170	270	298	110	110
99rbl.115	212	226	290	290	216	232	286	296	166	192	280	286	110	110
99rbl.119	226	248	282	284	210	222	286	304	188	206	270	282	112	114
99rbl.120	212	222	290	290	216	216	254	286	166	172	268	288	110	112
99rbl.123	224	232	284	302	214	216	298	304	168	170	284	284	110	110
99rbl.126	224	244	282	290	216	218	282	284	166	200	290	300	112	112
99rbl.127	218	224	290	290	210	218	284	292	150	166	270	286	110	110
99rbl.128	218	248	286	292	214	216	288	304	172	178	268	302	110	112
99rbl.129	214	222	286	286	216	236	292	304	192	196	284	298	110	110
99rbl.130	214	222	290	290	214	236	252	292	150	196	272	286	110	110
99rbl.131	254	254	282	288	214	214	270	296	212	216	266	278	110	110

99rbl.133	224	248	282	282	216	216	286	286	168	172	268	286	110	110
99rbl.135	212	212	282	290	216	216	290	292	174	178	274	274	110	110
99rbl.136	224	232	284	284	212	214	286	290	174	182	276	276	110	112
99rbl.137	212	212	280	286	212	214	274	300	216	216	284	286	110	110
99rbl.138	226	236	282	290	216	216	290	292	192	200	268	296	110	114
99rbl.140	214	226	282	288	212	218	264	290	168	170	294	294	110	112
99rbl.147	214	230	292	292	210	218	270	278	172	216	272	288	110	110
99rbl.149	214	232	282	288	204	218	290	292	172	172	292	300	110	112
99rbl.150	214	250	282	290	214	214	254	272	168	214	278	284	110	110
99rbl.156	224	226	282	282	210	214	266	272	150	174	300	306	110	110
99rbl.157	226	226	282	292	212	216	270	280	194	208	284	300	110	112
99rbl.158	228	250	290	292	214	214	284	294	170	170	270	270	110	110
99rbl.164	214	214	282	288	218	218	268	292	168	206	284	290	110	110
99rbl.166	224	232	282	290	204	210	252	282	150	182	274	296	110	112
99rbl.167	224	232	284	292	216	218	270	308	170	192	276	288	110	110
99rbl.168	224	224	286	290	222	224	288	292	150	170	278	292	110	110
99rbl.170	218	232	288	292	216	232	286	286	168	168	288	288	110	110
99rbl.172	224	230	282	294	216	220	280	290	178	192	286	302	110	110
99rbl.174	216	228	282	282	220	230	282	282	192	192	282	288	110	110
99rbl.177	226	228	282	282	210	214	256	286	166	168	268	284	110	114
99rbl.180	224	224	280	282	216	216	296	300	168	202	286	302	110	110
99rbl.182	212	226	286	288	208	218	302	310	170	194	266	290	110	110
99rbl.190	220	220	286	292	210	218	280	304	166	200	274	274	110	110
99rbl.191	226	234	288	292	202	216	274	290	162	176	290	296	110	110
99rbl.194	224	224	286	292	210	212	256	282	168	204	276	302	110	110
99rbl.195	218	232	282	286	212	216	290	294	176	204	278	296	110	110
99rbl.196	220	224	286	292	214	236	272	308	182	222	274	282	110	112
99rbl.207	238	238	282	286	202	214	254	284	196	204	276	278	110	110
99rbl.208	218	218	280	288	216	222	268	288	170	190	274	280	110	110
99rbl.209	214	224	282	290	214	230	290	298	192	202	288	296	110	112
99rbl.215	224	226	282	282	218	222	284	290	168	192	284	288	110	110
99rbl.219	218	228	286	288	210	214	286	308	192	194	278	280	110	112
99rbl.220	224	224	282	288	208	210	290	290	170	192	274	286	110	110
99rbl.222	212	228	282	286	216	220	286	308	170	196	276	294	110	110
99rbl.223	222	232	284	290	212	214	272	298	172	188	294	296	110	112
99rbl.224	212	226	286	286	214	234	276	304	170	170	274	284	110	114
99rbl.225	214	224	282	292	210	210	286	302	178	186	274	274	110	114

99rbl.227	224	232	292	292	214	218	274	274	168	170	272	276	110	110
99rbl.229	214	224	282	282	214	230	258	280	168	168	264	278	110	110
99rbl.230	224	228	284	286	204	218	270	304	178	184	282	292	110	112
99rbl.231	226	252	282	286	212	218	278	282	186	202	272	282	110	110
99rbl.233	214	226	286	288	216	222	270	270	172	186	284	298	112	114
99rbl.234	232	232	282	282	208	212	278	284	170	182	284	292	110	110
99rbl.239	224	232	290	292	214	214	272	292	150	168	266	298	110	110
99rbl.242	214	254	286	290	214	218	250	288	168	194	274	292	110	112
99rbl.243	222	224	286	292	208	216	278	308	202	210	294	310	110	112
99rbl.246	212	228	282	290	210	218	286	288	166	170	294	298	110	110
99rbl.248	212	214	282	282	208	214	268	268	170	172	274	282	110	110
99rbl.249	224	224	282	290	208	222	282	290	202	202	288	290	110	110
99rbl.256	212	214	282	282	208	216	256	290	168	168	282	300	110	110
99rbl.258	218	222	290	292	204	236	284	290	196	216	286	290	110	110
99rbl.259	224	226	282	282	222	230	266	272	214	216	280	284	110	110
99rbl.263	214	248	282	284	214	218	284	300	172	172	262	298	110	110
99rbl.267	218	222	282	290	214	218	296	298	168	172	288	298	110	110
99rbl.268	218	224	282	290	212	218	286	304	170	174	288	288	110	110
99rbl.274	222	222	282	282	216	216	260	284	172	216	288	288	110	112
99rbl.278	214	216	282	282	214	218	292	298	150	150	282	298	110	112
99rbl.281	224	240	284	292	214	222	300	322	188	192	280	284	112	112
99rbl.287	224	238	286	290	220	220	290	298	188	204	0	0	110	112
99rbl.289	214	230	280	288	216	218	282	306	170	170	280	290	110	112
99rbl.290	224	224	282	286	218	222	264	286	168	184	290	292	110	112
99rbl.292	224	238	286	290	218	236	290	310	206	216	272	278	110	110
99rbl.294	214	224	282	290	202	214	282	288	166	174	278	288	110	110
99rbl.295	214	214	282	284	214	214	254	254	170	170	278	284	110	112
99rbl.298	232	232	282	290	216	218	292	310	150	168	284	284	110	110
00rbl.001	224	228	290	292	214	222	284	298	194	194	274	288	110	112
00rbl.002	216	232	282	292	216	218	272	288	192	192	284	290	110	110
00rbl.005	224	232	282	288	214	218	270	270	192	210	286	292	110	114
00rbl.006	226	226	282	286	214	218	280	288	184	220	288	292	110	110
00rbl.007	222	228	284	286	218	218	264	268	204	210	274	284	110	110
00rbl.009	224	232	282	286	208	210	282	282	200	210	274	286	110	110
00rbl.010	218	218	286	294	214	222	286	286	178	192	274	280	110	112
00rbl.011	224	224	286	294	214	214	278	298	170	222	288	292	110	110
00rbl.012	226	228	284	290	214	218	268	292	178	196	282	290	110	110

00rbl.016	224	254	290	292	216	218	274	274	170	194	276	302	110	112
00rbl.022	228	254	284	290	214	214	272	290	150	170	266	272	110	110
00rbl.023	212	254	282	290	202	214	284	284	168	168	284	302	110	112
00rbl.025	226	230	284	284	228	228	284	298	170	170	278	284	110	110
00rbl.028	230	232	284	288	214	214	298	298	168	170	266	288	110	110
00rbl.040	228	232	284	292	214	218	0	0	150	168	266	286	110	110
00rbl.043	228	238	284	292	214	218	256	266	192	192	270	294	110	110
00rbl.048	224	228	290	290	212	216	256	302	198	218	270	294	110	110
00rbl.064	224	224	282	286	208	214	262	292	172	192	280	286	112	112
00rbl.065	222	250	286	286	214	218	294	296	190	198	270	288	110	112
00rbl.066	224	224	280	282	212	212	250	296	168	186	270	274	110	110
00rbl.069	218	222	280	282	218	224	292	302	170	170	284	292	110	110
00rbl.070	224	224	282	286	214	214	286	294	178	190	292	304	110	110
00rbl.073	224	224	282	288	214	218	262	280	194	216	276	280	110	114
00rbl.075	212	218	290	290	212	214	296	296	162	196	266	282	110	110
00rbl.077	218	218	282	282	212	222	272	284	174	186	282	304	110	110
00rbl.082	224	230	280	282	212	212	302	302	170	176	286	288	110	110
00rbl.085	214	222	282	282	210	218	264	290	190	204	274	274	110	110
00rbl.089	226	226	284	284	212	220	284	308	170	200	266	284	110	112
00rbl.096	218	224	286	290	202	202	250	252	196	196	290	302	110	110
00rbl.097	224	226	284	288	214	218	288	296	166	204	280	280	110	110
00rbl.099	214	226	286	290	202	218	256	288	168	182	286	294	110	110
00rbl.104	226	232	284	286	214	218	280	308	196	202	268	296	110	110
00rbl.107	222	232	290	290	214	222	214	274	198	198	282	304	110	112
00rbl.108	216	224	282	290	216	220	264	312	160	170	266	298	110	112
00rbl.111	240	240	282	290	214	218	270	274	150	170	290	290	110	110
00rbl.118	216	232	282	290	214	218	250	272	198	202	276	280	110	110
00rbl.120	220	228	288	290	214	218	284	284	192	206	282	302	110	110
00rbl.122	224	228	282	282	222	222	220	300	194	226	268	276	110	110
00rbl.123	218	258	282	290	212	212	284	310	168	170	292	294	112	116
00rbl.124	218	232	282	290	214	214	214	274	198	198	272	280	110	112
00rbl.126	222	224	290	290	204	236	270	292	166	216	272	282	110	114
00rbl.135	224	252	288	294	214	214	286	300	182	182	274	286	110	110
00rbl.137	218	228	284	290	214	220	298	306	150	200	290	296	110	110
00rbl.154	216	258	284	286	214	228	286	292	172	224	298	304	110	110
00rbl.155	218	244	288	292	214	224	286	294	208	208	276	284	110	110
00rbl.158	226	226	284	292	214	222	254	294	170	182	274	284	110	110

00rbl.160	226	230	288	292	216	220	266	302	162	220	278	292	110	110
00rbl.163	226	226	286	292	214	218	256	302	180	180	276	308	110	110
00rbl.164	232	232	282	290	210	214	282	290	150	196	280	284	110	116
00rbl.169	214	226	282	288	202	208	258	286	150	150	282	296	110	110
00rbl.176	220	226	292	292	212	214	252	308	150	194	276	292	110	110
00rbl.178	226	232	282	290	208	216	272	304	168	212	294	298	110	110
00rbl.179	222	222	286	286	214	218	270	308	170	170	286	298	110	110
00rbl.180	224	224	282	288	208	214	292	308	168	202	274	284	110	112
00rbl.183	212	232	282	282	222	232	272	284	150	170	284	288	110	110
00rbl.186	216	224	292	292	214	214	278	286	194	194	276	286	110	110
00rbl.191	220	220	286	292	216	218	292	300	178	178	266	282	110	110
00rbl.194	218	238	290	292	212	218	264	286	168	196	270	288	110	110
00rbl.196	228	234	286	286	218	230	266	312	198	202	276	282	110	110
00rbl.198	224	228	282	290	210	220	272	290	174	196	288	294	110	110
00rbl.202	224	226	284	290	202	214	220	220	162	170	270	286	110	110
00rbl.203	226	228	286	286	212	214	276	300	166	166	266	266	110	110
00rbl.204	228	228	282	282	212	216	252	270	176	200	284	284	110	110
00rbl.206	220	232	286	292	212	218	288	300	188	210	302	310	110	112
00rbl.212	214	214	282	282	212	224	292	298	176	208	284	298	110	110
00rbl.217	224	226	282	284	214	218	290	290	166	170	262	290	110	110
00rbl.223	220	226	286	292	214	214	264	286	174	180	276	282	110	110
00rbl.225	0	0	288	290	214	218	268	276	170	202	274	280	110	110
00rbl.227	234	234	288	294	212	218	272	300	150	192	284	284	110	110
00rbl.228	214	232	282	288	212	214	284	300	184	216	266	276	110	110
00rbl.232	226	234	284	294	212	214	272	288	170	186	286	290	110	114
00rbl.236	222	224	288	294	212	222	300	310	182	198	286	300	110	110
00rbl.239	212	226	284	292	212	212	282	292	178	202	284	288	110	110
00rbl.240	224	252	290	292	212	218	258	312	150	170	274	286	110	114
00rbl.242	212	224	286	290	214	226	258	308	170	204	290	296	110	110
00rbl.244	214	228	286	292	212	212	294	310	170	174	276	278	110	114
00rbl.245	218	228	282	286	204	214	258	272	168	168	292	292	110	110
00rbl.249	222	232	292	294	212	222	300	308	180	198	294	302	110	110
00rbl.257	218	224	284	288	218	226	274	274	170	170	282	282	110	112
00rbl.258	224	224	282	284	216	220	300	300	198	202	276	292	110	112
00rbl.263	228	228	284	288	216	220	284	300	168	198	278	288	110	110
00rbl.267	230	232	282	286	218	222	256	282	150	208	268	270	110	110
00rbl.268	226	232	282	284	218	220	304	312	168	168	286	298	110	110

00rbl.271	216	218	286	290	214	214	272	290	172	214	276	286	110	110
00rbl.273	224	226	282	288	208	236	256	312	168	170	286	288	110	112
00rbl.277	232	232	282	286	214	222	270	288	172	200	274	282	110	110
00rbl.278	228	228	288	292	210	212	256	284	172	178	278	278	110	110
00rbl.282	228	228	284	290	214	218	254	272	150	204	286	294	110	112
00rbl.284	218	218	286	286	210	218	280	290	200	208	298	298	110	112
00rbl.288	222	244	282	284	214	218	264	298	150	150	286	290	110	110
00rbl.290	224	228	284	286	214	218	300	310	170	172	274	274	110	110
00rbl.294	222	222	282	290	214	218	282	304	202	206	276	290	110	112
00rbl.295	226	228	282	288	218	218	286	304	166	170	280	290	110	112
00rbl.299	214	228	282	286	222	232	266	298	208	208	276	284	110	114
00rbl.301	232	232	284	292	216	220	286	286	172	198	276	288	110	112
00rbl.302	224	256	286	286	204	212	290	300	196	204	284	302	110	110
00rbl.304	216	228	288	292	214	218	304	304	168	174	284	302	110	112
00rbl.306	224	230	284	290	204	212	0	0	168	174	268	276	110	110
00rbl.307	224	230	284	292	214	218	238	312	150	164	264	282	110	110
00rbl.309	226	228	288	292	212	218	278	278	150	216	276	290	110	110
00rbl.312	224	224	284	292	214	218	270	300	0	0	0	0	110	110
00rbl.320	216	224	284	292	210	218	280	280	150	150	268	282	110	112
00rbl.321	224	226	286	290	202	218	286	304	196	206	282	292	110	110
00rbl.324	232	238	288	292	216	218	246	290	172	182	270	294	110	110
00rbl.325	226	228	282	292	214	222	286	294	168	168	264	276	110	112
00rbl.326	224	228	282	290	212	218	284	298	168	168	262	290	110	110
00rbl.327	226	228	284	286	202	214	292	310	176	192	288	304	110	110
00rbl.328	226	228	284	292	214	222	286	294	150	168	264	278	112	112
00rbl.335	230	232	282	290	210	210	300	300	170	170	270	284	110	110
00rbl.336	226	228	284	286	204	212	290	310	150	192	264	290	110	110
00rbl.337	216	218	286	288	214	214	272	286	190	214	268	274	110	112
00rbl.344	218	238	284	290	220	224	274	294	168	194	266	290	110	110
00rbl.347	216	226	288	288	216	220	224	274	190	214	282	282	110	112
00rbl.351	216	224	290	292	214	218	290	296	150	166	288	288	110	110
00rbl.354	228	228	284	290	214	218	288	294	214	214	266	296	110	110
00rbl.355	224	226	288	290	214	218	300	300	200	200	274	286	110	110
00rbl.357	224	230	286	290	214	218	278	280	150	170	278	282	110	114
00rbl.359	222	224	290	292	214	214	280	300	168	192	272	298	110	110
00rbl.364	216	254	286	292	214	214	274	274	150	172	0	0	110	110
00rbl.372	224	226	282	290	214	222	268	298	178	200	276	284	110	110

01rbl.002	224	250	280	290	214	222	282	288	168	168	272	288	110	110
01rbl.003	212	216	282	284	220	222	282	304	192	216	272	280	110	114
01rbl.004	226	250	286	292	216	222	282	294	178	194	274	284	110	110
01rbl.005	226	254	282	290	216	216	274	298	210	210	286	288	110	110
01rbl.009	250	254	286	286	212	222	260	282	218	218	274	290	110	110
01rbl.010	218	258	282	284	204	222	258	278	150	218	292	304	110	114
01rbl.013	218	254	282	282	224	232	260	260	218	218	274	290	110	110
01rbl.015	214	218	284	286	220	222	284	284	194	218	272	274	110	110
01rbl.017	218	252	282	292	214	224	282	290	170	218	272	288	110	110
01rbl.021	216	224	284	292	216	222	260	300	168	176	288	310	112	112
01rbl.022	226	250	284	286	224	228	278	282	150	196	274	304	110	114
01rbl.023	228	230	288	292	212	216	260	298	200	202	292	294	110	114
01rbl.024	250	252	282	286	214	222	282	308	196	224	290	290	110	110
01rbl.025	218	218	286	286	204	222	260	292	174	218	290	292	110	110
01rbl.026	224	230	284	290	212	218	252	292	168	178	288	288	110	112
01rbl.027	226	234	286	294	204	210	274	290	168	224	290	300	110	110
01rbl.030	224	224	290	290	214	216	280	298	158	220	274	298	110	112
01rbl.031	222	222	282	282	216	218	282	296	176	206	290	290	110	112
01rbl.032	226	226	290	290	214	224	264	298	150	150	268	294	110	110
01rbl.035	224	224	282	288	208	218	260	276	150	166	282	300	110	110
01rbl.037	216	222	288	288	212	218	298	300	176	188	280	298	110	110
01rbl.038	230	230	284	284	212	222	250	270	162	162	294	304	110	110
01rbl.042	224	232	292	292	214	222	272	274	170	170	268	272	110	112
01rbl.044	224	224	288	290	216	220	214	214	174	208	276	286	110	112
01rbl.046	214	244	284	286	208	214	268	286	178	178	274	292	110	110
01rbl.049	224	230	288	292	218	222	272	274	194	194	266	288	110	110
01rbl.050	214	250	288	288	216	220	268	292	196	206	286	296	110	110
01rbl.051	226	226	282	286	214	218	256	276	170	206	276	288	110	110
01rbl.053	214	230	284	284	214	214	282	298	150	150	274	294	110	112
01rbl.054	230	238	284	290	214	218	280	282	150	168	280	294	110	110
01rbl.057	224	230	284	286	214	214	282	284	196	210	284	294	110	110
01rbl.060	232	238	290	290	206	216	252	312	198	208	280	298	110	110
01rbl.061	226	230	282	290	228	232	268	278	200	212	270	278	110	110
01rbl.063	214	214	282	286	214	216	266	270	198	202	284	298	110	110
01rbl.067	216	216	282	292	214	214	280	290	196	220	266	280	110	110
01rbl.069	212	216	290	290	218	218	252	292	170	198	280	290	110	112
01rbl.073	224	232	282	290	218	228	286	294	198	214	266	270	110	110

01rbl.074	216	224	290	290	212	232	280	288	170	170	292	302	110	110
01rbl.078	216	254	282	292	214	218	214	292	196	196	284	288	110	110
01rbl.079	226	226	282	286	210	218	300	304	194	194	288	288	110	112
01rbl.083	226	230	290	290	212	222	272	308	174	192	268	284	110	112
01rbl.086	226	228	280	290	212	220	288	288	150	174	274	284	110	110
01rbl.087	218	224	284	284	216	230	270	286	178	208	282	294	110	110
01rbl.088	216	224	284	290	208	214	284	292	172	172	284	294	110	110
01rbl.090	216	222	284	284	204	214	288	288	168	190	282	286	110	110
01rbl.091	220	226	280	292	216	218	280	288	162	188	282	284	110	110
01rbl.093	214	226	284	286	214	236	256	294	162	204	292	294	110	110
01rbl.094	222	224	290	290	214	214	286	292	178	186	278	284	110	110
01rbl.096	224	242	282	290	210	214	220	288	150	150	270	292	110	110
01rbl.099	226	228	288	290	204	220	220	288	150	196	294	298	110	112
01rbl.104	226	228	282	286	218	218	266	288	196	222	278	302	110	110
01rbl.105	228	228	284	288	220	230	260	312	198	216	276	290	110	112
01rbl.107	214	224	284	292	216	216	298	298	168	190	272	300	110	110
01rbl.108	230	254	284	288	214	222	254	300	176	192	278	286	110	110
01rbl.109	218	228	286	288	214	222	284	292	198	218	274	288	110	110
01rbl.110	226	248	282	290	204	218	214	284	192	192	268	292	112	114
01rbl.113	216	234	282	282	214	214	290	300	184	198	294	304	110	112
01rbl.114	224	254	286	288	216	218	276	300	198	198	276	290	110	110
01rbl.118	244	254	284	286	214	214	308	312	178	186	276	290	110	110
01rbl.119	216	226	288	288	222	236	256	306	192	196	284	294	110	110
01rbl.121	220	224	284	290	212	214	286	298	150	196	280	286	110	112
01rbl.122	232	234	286	286	212	236	276	298	200	216	264	288	110	110
01rbl.124	218	222	288	292	212	214	280	288	200	202	288	296	110	114
01rbl.125	224	224	286	290	214	214	270	286	166	214	280	288	110	110
01rbl.126	214	224	282	284	218	220	264	264	178	190	276	294	110	110
01rbl.128	226	226	282	286	214	218	256	296	192	194	280	294	110	110
01rbl.133	214	222	282	290	212	214	252	284	174	184	288	292	110	112
01rbl.135	222	226	282	288	214	220	280	312	150	200	276	288	110	110
01rbl.139	224	224	284	290	216	220	290	290	192	214	282	292	110	110
01rbl.140	214	254	284	292	212	216	270	286	168	168	280	286	110	110
01rbl.142	226	228	282	288	214	214	292	292	150	170	290	296	110	110
01rbl.143	218	252	284	286	214	216	296	296	180	180	288	290	110	110
01rbl.144	214	218	282	290	214	220	280	290	200	206	276	286	110	114
01rbl.149	214	214	282	290	214	220	290	312	168	192	276	286	110	114

01rbl.150	218	224	282	282	216	232	256	306	166	174	276	288	110	110
01rbl.153	226	228	284	290	216	216	270	278	150	168	286	288	110	110
01rbl.158	212	214	286	292	218	220	284	298	202	222	284	298	110	112
01rbl.159	214	250	282	290	210	216	288	306	150	192	278	290	110	110
01rbl.160	230	232	282	290	212	214	282	290	168	174	274	280	110	112
01rbl.161	218	224	290	290	214	216	294	306	168	168	268	276	110	110
01rbl.162	224	248	282	286	208	214	272	290	168	170	286	298	110	110
01rbl.163	228	232	282	286	210	222	272	288	200	210	280	280	110	112
01rbl.164	218	224	286	292	214	216	260	272	168	196	266	296	110	112
01rbl.165	218	224	286	292	202	218	306	308	192	192	284	290	110	112
01rbl.167	224	226	282	284	214	230	266	288	196	210	282	284	110	112
01rbl.168	232	248	290	290	216	218	292	292	150	168	276	288	110	114
01rbl.169	214	226	288	290	212	214	290	298	186	214	280	286	110	110
01rbl.170	222	232	282	290	210	212	294	298	170	200	274	278	110	110
01rbl.171	212	214	286	290	212	214	292	302	170	178	276	290	110	110
01rbl.172	214	234	282	290	214	214	290	302	178	200	290	298	110	110
01rbl.174	224	248	290	290	216	230	304	308	150	202	276	290	110	110
01rbl.175	212	222	282	284	212	232	252	284	170	198	266	288	110	110
01rbl.179	224	228	282	286	218	226	272	288	174	210	278	280	110	112
01rbl.180	212	228	282	290	214	222	282	302	224	224	268	282	112	112
01rbl.185	226	232	290	290	204	214	250	308	188	218	284	292	110	110
01rbl.188	226	228	286	290	214	222	290	290	166	200	276	290	110	112
01rbl.189	214	214	282	290	212	218	272	298	168	214	268	304	110	110
01rbl.191	222	224	284	290	212	214	270	288	192	198	288	300	110	110
01rbl.193	212	230	282	282	214	218	288	304	182	202	270	284	110	110
01rbl.194	212	214	282	286	210	222	264	272	204	204	288	296	110	112
01rbl.195	226	232	282	292	216	218	284	294	192	202	284	288	110	110
01rbl.196	218	254	282	290	212	220	278	278	150	170	280	286	110	112
01rbl.197	214	224	282	288	214	216	288	288	150	150	274	286	110	110
01rbl.198	214	214	290	292	204	236	280	308	170	172	278	288	110	110
01rbl.207	220	226	286	288	214	216	284	304	182	182	292	292	110	110
01rbl.209	226	238	282	290	204	218	272	284	170	178	276	282	110	110
01rbl.211	238	244	282	284	216	216	272	282	150	214	282	292	110	110
01rbl.212	224	244	286	292	214	214	272	298	150	168	264	288	110	110
01rbl.213	216	228	282	282	214	218	252	306	170	170	294	306	110	114
01rbl.214	214	224	280	292	214	218	286	306	168	206	272	278	110	112
01rbl.215	224	232	282	290	214	214	298	298	166	194	282	288	110	110

01rbl.216	214	248	282	290	204	218	286	286	196	208	282	284	110	112
01rbl.217	222	224	288	290	212	218	292	296	174	202	286	288	110	112
01rbl.218	224	244	282	286	210	214	252	270	178	178	282	292	110	110
01rbl.220	224	226	290	290	222	222	274	292	194	210	284	288	110	112
01rbl.221	218	218	284	290	220	224	272	272	196	214	292	300	110	110
01rbl.223	222	224	284	286	214	222	260	270	204	204	280	298	110	110
01rbl.224	222	232	286	290	214	216	286	298	168	168	268	280	110	110
01rbl.225	214	228	282	284	206	220	282	308	170	170	284	300	110	112
01rbl.232	222	224	282	290	214	226	290	312	180	182	284	292	110	110
02rbl.002	214	232	284	286	218	222	284	294	190	208	270	276	110	110
02rbl.003	224	226	286	290	208	210	270	274	168	176	290	294	112	112
02rbl.004	222	228	282	282	208	210	266	272	182	184	276	294	110	112
02rbl.008	226	230	286	292	214	218	268	312	186	204	284	310	110	110
02rbl.011	218	222	282	292	212	214	254	270	170	176	274	294	110	112
02rbl.012	216	226	284	288	214	218	272	286	174	208	262	288	110	112
02rbl.014	228	228	286	294	212	218	272	280	160	208	276	286	110	110
02rbl.016	226	230	284	290	208	218	264	312	170	202	270	274	110	110
02rbl.018	224	226	284	294	214	214	290	290	170	192	270	296	110	112
02rbl.019	226	226	288	292	216	218	278	286	150	172	270	288	110	110
02rbl.020	216	254	288	288	208	218	252	286	170	174	274	288	110	110
02rbl.024	218	232	282	286	212	214	272	282	150	216	286	288	110	112
02rbl.025	224	224	282	282	212	214	270	304	174	184	274	288	110	112
02rbl.030	230	238	282	282	218	218	278	312	200	202	274	288	110	110
02rbl.032	218	234	282	290	212	216	254	270	206	226	270	284	110	110
02rbl.033	222	254	282	290	218	222	284	288	192	198	286	286	110	110
02rbl.038	228	228	286	292	212	218	268	270	210	210	294	294	110	110
02rbl.039	232	238	282	282	214	214	272	304	178	218	276	288	110	110
02rbl.043	226	252	286	290	210	216	308	308	172	202	262	296	110	110
02rbl.048	214	232	282	284	208	214	254	300	168	192	280	294	110	110
02rbl.053	230	248	282	292	212	222	270	290	190	192	274	300	110	110
02rbl.061	222	224	282	286	214	236	258	288	150	166	282	290	110	112
02rbl.062	214	238	286	292	214	222	256	304	166	192	266	278	112	112
02rbl.067	214	224	284	292	214	218	290	300	150	186	296	298	110	110
02rbl.068	214	224	290	292	210	214	264	290	192	192	290	292	110	110
02rbl.071	212	224	284	292	204	208	270	294	194	200	274	290	110	110
02rbl.072	216	224	284	290	214	214	264	290	192	192	284	296	110	110
02rbl.078	212	214	282	282	204	204	254	288	174	202	284	290	110	110

02rbl.083	214	226	282	288	218	222	298	304	196	204	274	288	110	110
02rbl.086	216	220	286	290	214	222	270	288	174	192	282	288	110	112
02rbl.088	224	224	286	290	216	222	264	290	202	222	278	288	110	112
02rbl.093	224	224	284	290	210	232	278	290	192	196	290	296	110	110
02rbl.095	224	226	288	290	208	214	266	312	170	198	282	284	110	110
02rbl.100	226	244	284	292	214	218	298	304	170	196	282	290	110	110
02rbl.102	218	224	282	286	220	222	266	294	206	208	278	288	110	110
02rbl.107	224	232	282	286	214	230	290	300	192	216	282	284	110	110
02rbl.108	224	228	284	284	212	214	274	292	192	192	286	288	110	110
02rbl.114	222	236	288	290	214	224	266	292	150	170	278	282	114	114
02rbl.117	216	218	284	288	214	222	214	312	162	226	286	300	110	110
02rbl.121	228	254	286	286	220	222	254	284	192	198	284	286	110	110
02rbl.126	214	214	282	290	212	214	272	274	162	200	274	282	110	112
02rbl.128	224	256	284	290	214	218	270	290	170	176	294	298	110	112
02rbl.132	228	244	284	290	214	214	262	314	170	202	284	296	110	114
02rbl.133	222	222	290	290	214	220	214	296	196	200	270	286	110	112
02rbl.137	214	214	288	288	216	216	292	298	192	194	286	294	110	112
02rbl.140	224	226	284	288	208	216	286	296	202	222	280	298	110	110
02rbl.142	228	228	282	286	214	236	304	306	196	196	268	294	110	110
02rbl.146	224	224	282	292	214	214	286	304	150	168	282	294	112	112
02rbl.149	230	232	282	290	218	230	296	304	170	174	272	300	110	114
02rbl.152	212	244	282	282	218	230	294	298	168	192	282	284	110	110
02rbl.153	226	232	286	288	218	228	264	268	174	174	270	276	110	110
02rbl.157	226	232	282	286	214	214	260	294	188	196	276	284	110	110
02rbl.159	216	244	284	286	222	232	272	278	210	210	274	288	110	112
02rbl.161	0	0	280	286	218	218	288	298	198	202	286	300	110	110
02rbl.163	224	228	286	294	218	222	220	270	208	210	284	294	110	110
02rbl.164	214	222	286	292	214	218	278	298	186	206	280	284	110	110
02rbl.165	212	228	286	292	216	236	258	260	182	196	274	300	110	110
02rbl.172	216	230	284	286	214	220	252	314	202	202	276	278	112	114
02rbl.173	222	222	286	290	208	216	272	280	190	200	276	284	110	110
02rbl.180	214	226	282	282	210	216	272	286	206	212	298	306	110	112
02rbl.184	214	224	282	286	210	214	268	282	166	196	284	286	110	110
02rbl.185	222	244	282	292	214	214	272	272	150	190	284	296	110	110
02rbl.186	216	246	286	288	208	214	278	308	150	170	276	284	110	110
02rbl.188	222	232	282	284	204	208	254	272	168	168	282	296	110	110
02rbl.192	228	232	286	286	212	222	272	290	192	192	284	284	110	110

02rbl.197	224	248	286	290	212	214	286	298	166	202	284	300	110	110
02rbl.201	214	214	284	284	212	212	222	292	190	220	274	288	110	110
02rbl.210	226	230	284	284	204	218	268	292	188	216	274	292	110	110
02rbl.212	234	248	284	288	212	212	282	290	174	200	270	286	110	112
02rbl.214	212	226	284	292	214	218	298	298	150	212	270	288	110	110
02rbl.215	224	226	284	292	204	216	260	284	196	196	270	292	110	110
02rbl.216	212	228	288	288	218	220	264	276	168	196	288	288	110	110
02rbl.217	214	226	284	284	204	222	292	296	168	174	272	296	110	110
02rbl.223	216	224	290	292	202	214	284	286	178	196	286	286	110	110
02rbl.226	218	220	284	286	214	214	278	288	168	190	278	288	110	110
02rbl.229	224	250	286	292	210	216	260	290	172	172	266	288	112	112
02rbl.230	250	250	292	294	212	214	270	274	170	192	284	286	110	110
02rbl.235	216	218	286	288	212	214	252	292	168	188	262	274	110	110
02rbl.236	218	218	286	286	208	214	286	294	172	210	266	288	110	112
02rbl.237	222	224	284	286	210	214	288	288	202	216	266	274	110	110
02rbl.242	254	258	282	292	214	218	256	276	170	200	270	274	110	110
02rbl.243	218	218	282	282	208	218	272	288	206	222	270	282	110	110
02rbl.246	228	228	282	292	202	214	256	312	190	192	270	284	110	110
02rbl.252	212	224	288	292	210	214	288	302	168	196	272	288	110	110
02rbl.253	222	222	282	282	216	218	286	290	202	208	284	286	110	112
02rbl.254	214	224	286	290	214	218	256	302	190	200	282	296	110	112
02rbl.255	222	248	286	290	214	218	284	288	190	190	290	294	110	110
02rbl.260	224	238	282	290	204	212	264	264	190	202	284	300	110	110
02rbl.261	226	248	284	290	212	222	278	282	168	200	266	304	110	110
02rbl.264	212	224	288	290	214	222	250	312	168	200	284	304	110	110
02rbl.265	224	230	282	284	204	212	270	294	196	214	292	294	110	110
02rbl.267	214	224	290	290	214	218	280	290	180	182	282	284	110	110
02rbl.268	222	224	282	286	212	222	272	282	172	208	282	284	110	112
02rbl.271	214	228	284	286	214	236	278	308	170	170	274	284	110	110
02rbl.274	224	230	284	286	214	218	250	254	186	190	270	282	110	110
02rbl.277	226	226	282	286	210	218	256	292	170	170	276	284	110	110
02rbl.278	254	254	286	292	208	214	274	288	178	184	286	286	110	110
02rbl.279	218	230	286	290	214	218	220	298	194	202	274	286	110	110
02rbl.291	218	224	286	290	214	218	308	308	194	202	274	284	110	112
02rbl.295	214	224	286	290	204	214	288	308	150	178	274	274	110	112
02rbl.300	218	224	282	290	202	214	220	308	150	210	278	286	110	112
02rbl.304	214	224	282	292	212	214	254	278	198	202	262	290	110	110

02rbl.306	220	228	282	282	222	236	220	252	202	208	270	274	110	110
02rbl.307	214	226	290	290	204	236	256	282	170	178	284	294	110	110
02rbl.311	218	218	282	288	214	214	282	294	202	204	270	274	110	112
02rbl.319	224	226	286	286	214	216	270	272	166	168	288	288	110	112
02rbl.323	222	222	288	290	204	212	256	312	170	202	290	298	110	110
02rbl.327	238	246	286	290	208	210	282	286	190	196	282	286	110	110
02rbl.328	212	216	282	282	202	216	280	292	150	202	286	288	110	110
02rbl.330	222	232	282	286	202	214	290	298	162	190	278	288	112	114
02rbl.335	218	224	282	282	216	232	288	288	170	170	274	290	110	110
02rbl.337	212	214	284	290	212	218	214	268	202	202	288	288	110	110
02rbl.338	216	226	282	290	208	210	278	284	168	174	286	288	110	110
02rbl.339	214	226	288	290	216	218	264	272	208	208	286	288	110	114
02rbl.340	224	224	286	290	208	210	260	272	170	190	264	288	110	112
02rbl.341	224	224	282	286	214	222	274	300	162	192	276	308	110	114
02rbl.345	224	252	282	282	210	218	270	270	176	176	276	292	110	110
02rbl.350	222	224	282	282	204	216	304	304	166	168	286	298	110	110
02rbl.351	214	224	290	290	216	218	272	272	170	208	280	284	110	110
02rbl.353	212	214	284	290	210	216	272	272	196	196	282	294	110	110
03rbl.001	228	232	290	290	212	222	274	274	150	150	276	288	112	112
03rbl.005	218	224	284	292	216	220	254	290	150	150	272	298	110	110
03rbl.006	222	224	280	290	214	218	290	298	174	174	266	292	110	112
03rbl.015	214	226	284	286	214	218	252	292	170	170	274	284	112	112
03rbl.016	214	232	280	290	208	222	254	312	150	208	268	286	110	110
03rbl.019	214	216	284	288	216	218	256	292	208	208	276	276	110	112
03rbl.023	228	248	282	284	210	222	292	306	170	170	286	296	110	110
03rbl.026	214	216	282	284	214	222	272	276	166	200	282	298	110	110
03rbl.027	226	228	282	286	224	232	294	312	198	208	272	288	110	110
03rbl.029	232	252	288	290	220	228	254	254	174	178	288	290	110	112
03rbl.030	214	214	290	290	220	222	264	278	168	194	292	294	110	110
03rbl.031	214	226	282	288	210	214	278	278	168	228	286	288	110	110
03rbl.032	214	244	282	290	214	220	282	282	150	166	284	300	110	110
03rbl.033	226	250	286	288	218	220	286	294	168	206	278	284	110	112
03rbl.034	212	224	282	290	214	214	286	286	162	196	288	298	110	110
03rbl.035	214	248	290	290	216	218	276	280	170	178	268	286	110	110
03rbl.038	218	232	282	290	214	218	290	298	170	200	280	280	110	110
03rbl.039	218	218	284	290	214	214	280	288	168	206	286	288	110	110
03rbl.041	214	228	284	290	208	208	296	296	172	172	282	298	110	112

03rbl.042	214	222	282	284	218	222	272	280	184	196	276	282	110	110
03rbl.043	222	224	282	284	212	218	248	274	168	176	288	288	110	110
03rbl.044	214	244	282	286	214	218	272	306	166	172	284	288	110	110
03rbl.046	218	238	282	290	214	218	288	296	170	170	266	268	110	110
03rbl.049	228	232	282	286	228	234	268	292	170	198	270	294	110	110
03rbl.050	224	224	286	288	204	216	256	304	194	214	270	280	110	110
03rbl.053	214	230	290	290	208	218	214	254	168	192	264	292	110	110
03rbl.054	226	248	286	290	218	230	272	286	168	194	276	292	110	110
03rbl.057	222	224	282	282	214	214	268	300	170	176	282	300	110	110
03rbl.058	214	218	284	286	212	214	264	284	170	174	284	292	110	110
03rbl.059	222	232	284	290	210	214	272	272	196	196	280	288	112	112
03rbl.063	222	224	282	286	212	218	294	294	188	206	264	280	110	112
03rbl.069	214	222	282	282	212	214	272	286	168	182	282	284	110	110
03rbl.070	214	222	284	286	220	222	256	312	162	168	266	288	110	110
03rbl.071	228	228	282	284	212	218	278	284	186	202	278	292	110	110
03rbl.072	214	222	282	284	210	218	278	282	186	186	278	292	110	110
03rbl.076	224	224	280	290	202	218	284	284	194	202	284	288	110	110
03rbl.082	214	254	286	286	214	218	282	298	170	170	266	284	110	112
03rbl.086	224	224	284	290	204	214	270	284	150	190	288	304	110	110
03rbl.089	214	226	282	284	210	214	258	266	190	206	264	280	110	110
03rbl.091	226	228	284	290	208	214	264	310	180	204	280	288	110	110
03rbl.093	218	244	282	292	212	222	264	272	166	192	270	282	110	110
03rbl.105	222	224	282	286	214	216	282	286	166	204	276	288	110	112
03rbl.106	214	216	284	286	216	220	272	284	170	186	284	284	110	110
03rbl.108	224	224	286	290	204	216	286	292	194	204	286	286	110	112
03rbl.112	226	228	282	286	204	214	282	292	166	196	278	286	110	110
03rbl.114	224	250	282	286	204	210	270	290	170	170	276	288	110	110
03rbl.115	222	226	282	286	202	214	270	286	180	204	288	290	110	112
03rbl.116	226	228	282	284	204	220	274	288	170	196	284	286	110	112
03rbl.122	224	226	286	286	202	212	272	290	186	204	276	284	110	110
03rbl.124	224	252	286	288	202	210	288	288	150	204	276	284	112	112
03rbl.137	224	232	282	282	216	228	284	296	210	210	270	270	110	110
03rbl.138	212	224	282	290	214	218	266	282	202	204	270	288	110	110
03rbl.140	212	238	282	290	216	218	308	308	176	220	286	290	110	110
03rbl.142	222	226	284	292	210	216	268	284	174	190	298	302	110	112
03rbl.145	224	224	282	286	208	208	298	308	208	208	270	276	110	112
03rbl.146	228	232	290	290	218	218	292	312	194	208	268	272	110	110

03rbl.149	224	232	290	292	218	218	220	292	170	194	268	290	110	110
03rbl.150	218	218	282	290	212	214	262	278	168	170	286	292	110	110
03rbl.156	228	244	282	286	218	222	284	300	182	208	266	290	110	110
03rbl.157	222	224	292	292	202	208	282	282	190	202	272	276	110	110
03rbl.159	228	244	284	290	214	216	270	294	150	170	278	284	110	112
03rbl.160	222	224	282	290	218	234	254	290	168	174	280	284	110	110
03rbl.165	214	222	286	286	218	218	284	290	150	192	284	284	110	112
03rbl.167	214	214	286	286	210	214	250	260	170	202	276	288	110	112
03rbl.171	214	226	282	288	210	210	272	308	166	194	284	284	110	114
03rbl.173	222	226	282	282	212	214	274	274	150	198	276	286	110	114
03rbl.174	214	244	282	290	216	218	272	298	170	198	286	302	110	110
03rbl.176	224	248	282	286	210	212	280	284	182	186	290	296	110	110
03rbl.178	218	228	288	290	212	214	268	286	150	150	278	282	112	112
03rbl.179	224	226	290	290	212	216	292	306	196	204	296	300	110	110
03rbl.181	226	228	282	284	208	218	288	288	174	226	268	286	110	112
03rbl.185	214	214	286	290	216	222	288	290	150	168	288	294	110	110
03rbl.190	224	224	282	286	210	220	272	274	178	196	288	296	110	110
03rbl.192	222	224	282	290	212	216	272	294	168	202	268	302	110	110
03rbl.194	218	222	290	292	204	222	290	312	178	178	286	302	110	110
03rbl.196	224	248	282	282	212	212	260	288	184	216	276	296	110	110
03rbl.199	222	232	282	282	212	228	264	300	192	210	276	280	110	112
03rbl.209	214	224	290	292	212	216	288	300	170	178	282	288	110	110
03rbl.218	224	228	282	290	210	214	290	292	196	228	272	282	110	110
03rbl.220	214	214	282	286	210	218	272	300	222	222	296	296	110	112
03rbl.222	226	226	290	292	214	214	288	298	168	192	288	298	110	116
03rbl.224	218	226	290	292	214	214	286	302	186	216	282	290	110	112
03rbl.231	214	226	290	290	214	236	250	304	196	204	282	288	110	110
03rbl.232	214	226	282	290	210	214	286	298	168	190	290	298	110	116
03rbl.235	224	228	290	292	218	222	254	294	174	178	286	288	110	110
03rbl.236	226	226	282	288	212	220	290	298	196	208	262	294	110	110
03rbl.244	214	230	284	290	218	222	272	272	196	220	270	286	110	110
03rbl.250	214	214	282	290	212	220	256	278	196	216	294	306	110	110
03rbl.258	222	226	286	286	208	218	272	312	172	190	278	284	110	112
03rbl.259	214	222	282	290	214	218	286	292	190	208	276	286	110	110
03rbl.262	218	228	282	286	204	210	256	284	182	208	268	284	110	112
03rbl.264	214	224	290	290	214	222	252	272	190	192	272	278	110	110
03rbl.272	214	228	288	290	214	236	264	304	150	190	286	296	110	110

03rbl.273	226	226	282	288	202	222	250	274	186	200	270	274	110	112
03rbl.283	230	232	280	282	214	214	274	274	194	210	272	286	110	110
03rbl.284	224	224	288	290	202	210	252	292	170	186	286	298	110	114
03rbl.285	224	254	282	290	202	210	270	290	150	196	282	288	110	110
03rbl.294	224	232	282	284	214	214	254	270	172	204	282	282	110	110
03rbl.297	212	214	284	290	218	222	282	284	194	214	276	284	110	114
03rbl.298	232	240	286	290	214	216	272	286	0	0	278	282	110	110
03rbl.299	214	224	282	290	210	218	292	298	166	168	286	300	110	110
03rbl.305	230	238	282	284	214	220	274	290	194	216	274	286	110	112
03rbl.306	218	218	284	286	222	232	252	256	192	194	288	298	110	112
03rbl.307	214	214	282	286	218	222	254	258	188	198	280	288	110	110
03rbl.312	214	222	282	282	214	214	288	306	170	182	288	310	110	110
03rbl.316	212	222	286	290	222	236	280	292	178	180	272	288	110	110
03rbl.322	214	224	282	290	222	230	268	294	166	168	288	298	110	112
03rbl.325	214	214	282	290	220	222	252	288	158	208	276	298	110	110
03rbl.326	212	226	290	290	212	222	286	288	222	224	288	298	110	110
03rbl.327	214	224	282	288	204	218	272	288	196	208	268	282	112	114
03rbl.333	248	254	284	288	216	218	292	306	202	202	272	288	110	110
03rbl.334	222	230	282	288	210	218	272	272	166	166	288	294	110	112
03rbl.335	214	224	288	288	202	216	270	290	208	224	282	298	110	110
03rbl.337	224	226	282	292	214	218	302	312	168	202	288	300	110	110
03rbl.338	226	248	286	288	208	222	252	284	196	196	286	288	110	110
03rbl.340	218	226	286	290	216	222	258	282	194	200	278	284	110	110
03rbl.343	224	228	282	282	212	212	272	286	182	182	268	296	110	110
03rbl.349	224	226	284	286	218	230	272	282	192	204	270	278	110	112
03rbl.359	224	228	288	290	214	214	264	302	204	208	288	288	110	110
03rbl.372	218	222	282	290	214	214	288	290	168	168	290	290	110	110
04rbl.003	226	242	282	290	230	232	294	294	168	194	262	276	110	112
04rbl.005	214	228	290	292	212	214	272	286	202	220	270	292	110	110
04rbl.012	224	252	290	290	202	214	222	288	194	196	286	288	110	110
04rbl.017	214	224	286	290	212	218	286	304	170	218	264	286	110	110
04rbl.019	218	224	282	286	208	212	300	308	176	192	266	288	110	114
04rbl.028	226	242	282	286	214	232	256	274	172	204	282	286	110	112
04rbl.029	224	256	286	288	202	216	270	286	168	202	270	284	110	112
04rbl.031	224	250	286	286	214	218	280	286	170	208	284	286	110	110
04rbl.042	224	224	282	290	216	236	274	282	170	200	266	278	110	112
04rbl.047	224	224	290	290	214	218	260	294	168	170	272	294	110	110

04rbl.054	218	224	282	292	216	218	296	308	216	216	266	274	110	110
04rbl.060	224	226	288	288	218	236	286	304	168	202	278	296	110	110
04rbl.061	214	226	284	284	210	214	278	286	204	204	288	292	110	112
04rbl.064	214	224	288	288	214	218	264	292	172	174	276	288	112	112
04rbl.065	212	224	286	292	218	232	264	272	174	220	276	280	110	112
04rbl.066	218	222	290	290	210	212	274	276	168	170	284	288	110	110
04rbl.068	212	226	286	292	218	222	288	312	198	202	290	292	110	112
04rbl.081	218	228	282	292	212	216	264	288	166	184	270	280	110	110
04rbl.086	212	226	290	290	202	214	272	286	178	208	266	278	110	110
04rbl.088	214	224	282	284	210	214	266	290	170	192	274	296	110	110
04rbl.091	222	224	282	292	216	218	274	312	190	190	272	276	110	110
04rbl.094	222	222	282	286	214	220	252	288	150	194	290	292	110	112
04rbl.097	222	224	280	282	212	218	286	292	150	174	286	288	110	110
04rbl.100	224	226	282	286	210	216	284	288	224	226	264	286	112	114
04rbl.107	218	226	282	282	212	216	264	294	170	170	276	294	110	110
04rbl.118	224	230	286	290	218	218	290	310	194	210	284	288	110	110
04rbl.121	224	224	290	290	214	218	264	284	166	176	270	278	110	110
04rbl.126	212	212	280	280	210	214	296	302	170	218	266	270	110	110
04rbl.132	224	234	286	290	202	210	280	282	170	200	288	290	110	110
04rbl.137	222	228	282	290	212	214	298	300	168	212	274	282	110	110
04rbl.151	226	230	284	286	218	232	270	288	196	196	284	286	110	112
04rbl.152	224	254	284	290	210	214	260	286	168	212	282	286	110	110
04rbl.154	224	230	284	292	218	230	222	288	190	214	270	282	110	110
04rbl.160	214	224	282	292	222	222	252	262	176	206	288	290	110	112
04rbl.161	214	224	286	290	214	214	260	314	170	172	276	296	110	112
04rbl.164	222	224	282	282	210	214	276	296	174	176	284	290	110	110
04rbl.176	226	244	284	284	218	218	258	288	150	174	284	284	110	110
04rbl.177	214	226	286	288	216	218	274	292	196	206	284	284	110	112
04rbl.186	224	226	280	280	218	218	280	292	190	198	270	286	110	110
04rbl.190	244	244	282	290	212	214	256	264	170	192	284	294	110	110
04rbl.195	214	214	288	288	222	222	292	292	176	210	286	300	110	112
04rbl.201	226	228	282	286	218	228	252	290	150	150	284	296	110	110
04rbl.202	224	224	282	288	214	222	254	304	170	204	262	278	110	110
04rbl.205	214	222	286	288	218	218	296	302	170	224	288	296	110	112
04rbl.209	214	224	282	286	214	222	254	284	170	204	286	296	110	110
04rbl.210	228	244	290	292	208	222	274	276	178	196	282	282	110	112
04rbl.216	222	224	292	292	214	222	254	304	204	210	280	288	110	110

04rbl.220	224	228	282	282	212	218	290	300	216	218	264	276	110	112
04rbl.221	214	226	282	284	212	214	286	286	170	190	282	288	110	110
04rbl.224	212	228	284	286	222	228	268	286	200	204	270	270	110	110
04rbl.226	222	232	286	286	218	222	266	290	150	172	278	286	110	110
04rbl.228	220	224	282	288	210	214	272	298	192	202	272	288	110	110
04rbl.233	222	246	286	288	210	214	274	300	170	172	262	288	110	112
04rbl.237	224	230	282	286	212	214	290	304	202	202	268	270	110	110
04rbl.246	214	222	286	290	202	218	280	288	174	180	290	302	110	114
04rbl.249	212	254	282	290	212	214	280	282	192	210	300	302	110	110
04rbl.255	214	224	286	292	208	218	298	314	176	204	286	288	110	114
04rbl.266	236	242	286	286	222	232	276	300	178	184	278	284	110	114
04rbl.267	212	222	288	290	214	214	288	312	170	222	290	300	110	112
04rbl.272	228	228	282	286	212	222	274	292	150	192	300	304	110	112
04rbl.277	216	224	286	290	204	222	274	292	176	206	286	294	110	110
04rbl.279	216	224	290	290	212	232	292	298	194	194	276	298	110	112
04rbl.294	224	232	282	282	210	230	262	300	168	206	276	284	110	110
04rbl.297	224	226	282	282	214	214	298	308	196	214	296	300	110	114
04rbl.298	224	224	282	284	214	214	260	274	196	216	264	302	110	110
04rbl.300	224	230	280	288	214	230	296	300	150	150	276	294	110	112
04rbl.303	214	226	290	290	214	222	292	308	150	214	282	286	110	110
04rbl.306	224	226	282	284	214	214	260	308	174	214	264	290	110	114
04rbl.307	224	224	284	290	214	218	258	260	194	216	262	274	110	110
04rbl.312	224	232	290	290	208	218	284	294	168	170	280	298	110	112
04rbl.313	214	224	282	284	204	214	272	286	168	204	280	286	110	110
04rbl.316	212	224	282	290	214	222	290	304	150	222	272	274	110	114
04rbl.317	218	224	280	288	212	214	288	290	168	168	274	292	112	114
04rbl.330	224	226	280	284	212	218	284	294	162	172	280	288	110	110
04rbl.339	218	224	280	280	210	212	252	254	190	202	288	290	110	110
04rbl.345	214	224	282	286	210	218	252	280	172	212	282	292	110	110
04rbl.346	212	224	282	282	210	212	250	274	192	212	290	294	110	112
04rbl.348	224	226	282	292	208	214	264	288	204	204	280	288	110	110
04rbl.355	214	226	282	282	202	214	276	286	204	208	288	292	110	110
04rbl.358	214	224	286	290	210	214	284	290	198	224	284	286	110	110
04rbl.368	224	226	288	288	214	220	252	254	210	210	282	296	110	110
04rbl.371	226	254	282	284	216	220	260	276	184	206	266	292	110	112
04rbl.383	214	226	280	280	202	208	256	274	150	176	288	292	110	110
04rbl.384	224	226	280	288	214	222	292	300	170	214	266	276	110	110

04rbl.388	224	236	282	284	208	214	274	308	170	210	284	286	110	112
04rbl.390	220	252	280	286	212	214	272	276	170	194	266	288	110	112
04rbl.400	222	226	282	286	214	236	272	296	150	208	288	300	110	110
04rbl.404	242	258	284	284	212	214	272	286	162	198	276	298	110	112
04rbl.409	236	236	286	288	212	222	254	292	150	188	272	280	110	114
04rbl.425	212	232	286	288	202	204	278	310	196	224	274	274	110	110
04rbl.433	214	242	280	280	216	216	292	296	170	202	266	284	110	112
04rbl.441	224	250	280	288	216	222	274	288	170	172	286	288	110	112
04rbl.443	224	236	288	288	214	218	286	296	192	216	276	282	110	110
04rbl.446	214	222	280	286	214	216	272	272	170	208	268	286	110	110
04rbl.463	214	214	280	288	214	220	274	282	196	196	288	294	110	110
04rbl.464	218	224	290	290	208	214	214	214	200	200	284	300	112	112
04rbl.471	212	216	282	282	210	216	272	276	170	204	300	300	112	114
04rbl.472	214	222	282	288	214	214	272	272	172	226	268	276	110	110
04rbl.473	224	226	282	286	212	214	288	300	178	178	286	308	110	110
04rbl.474	214	224	286	290	212	222	288	302	206	216	274	288	110	110
04rbl.477	214	224	282	282	208	214	216	292	162	214	278	284	110	110
04rbl.480	214	224	290	290	208	210	270	298	168	196	284	294	110	112
04rbl.483	214	222	290	290	210	214	296	298	192	202	280	290	110	112
04rbl.484	226	226	284	288	220	222	256	284	166	170	284	298	110	110
04rbl.485	212	228	290	290	208	214	264	274	168	192	280	292	110	110
04rbl.491	214	246	282	286	212	220	286	286	150	186	286	296	110	112
04rbl.492	214	224	282	282	214	216	274	276	184	194	276	290	110	110
04rbl.493	214	232	282	290	222	236	270	282	188	192	282	284	110	110
04rbl.494	222	226	280	280	204	210	298	300	168	192	266	282	110	112
04rbl.495	224	228	288	290	212	214	256	288	170	204	292	296	110	112
04rbl.499	214	232	282	292	214	214	260	274	170	176	286	286	110	110
04rbl.502	224	224	280	280	218	226	286	292	178	178	282	290	110	110
04rbl.506	224	244	286	286	216	218	284	288	202	202	300	300	110	110
04rbl.519	218	226	290	290	214	218	286	288	196	216	274	284	110	110
04rbl.539	218	228	282	286	214	218	252	274	202	204	270	278	110	112
04rbl.545	212	224	282	282	214	222	280	294	196	208	286	290	110	110
04rbl.546	226	246	282	286	218	236	294	310	206	210	288	296	110	110
04rbl.550	224	228	282	286	210	218	290	296	170	202	270	288	110	110
04rbl.551	228	248	286	286	218	218	268	268	204	210	272	304	110	110
04rbl.556	214	246	282	282	214	218	286	296	198	204	284	288	110	110
05rbl.005	224	254	290	290	214	230	250	296	150	170	276	298	110	110

05rbl.009	214	224	290	290	214	218	258	280	168	176	270	294	112	112
05rbl.014	218	222	286	288	212	216	274	294	190	202	280	290	110	112
05rbl.022	218	224	282	290	212	218	254	278	196	196	274	292	110	110
05rbl.030	222	226	282	282	208	214	250	278	170	216	276	288	110	110
05rbl.033	230	232	282	284	218	222	288	304	150	202	298	304	110	114
05rbl.043	212	224	288	290	212	214	282	300	204	204	274	298	110	110
05rbl.044	214	224	280	292	204	214	290	290	150	216	280	284	110	110
05rbl.045	226	232	284	286	202	222	298	300	204	212	276	296	110	114
05rbl.053	230	232	282	282	208	218	288	304	150	202	296	302	110	114
05rbl.056	212	246	284	292	208	210	274	310	188	202	282	306	110	110
05rbl.058	224	254	280	286	214	214	264	284	170	178	272	280	110	110
05rbl.059	220	226	284	290	210	212	286	312	190	200	284	288	110	110
05rbl.061	214	232	284	292	212	214	252	296	174	174	280	294	110	114
05rbl.063	214	232	282	286	214	216	220	304	170	200	286	292	110	110
05rbl.065	228	228	290	292	216	218	272	282	172	200	286	294	110	110
05rbl.073	224	224	282	286	216	218	272	294	216	218	298	302	110	110
05rbl.078	222	224	280	286	202	214	266	274	194	204	288	294	110	110
05rbl.080	222	222	282	284	212	214	264	284	194	194	292	304	110	110
05rbl.081	222	252	282	288	218	218	262	290	188	212	276	286	110	110
05rbl.082	224	236	282	286	212	214	270	286	150	196	282	286	110	110
05rbl.083	224	224	286	286	210	212	272	274	150	202	280	280	110	110
05rbl.086	226	252	282	284	214	218	286	304	204	204	268	292	110	110
05rbl.090	224	244	284	290	214	214	282	294	168	216	268	288	110	110
05rbl.096	224	226	288	288	208	214	286	292	170	192	266	282	110	112
05rbl.099	224	228	282	284	214	236	286	296	170	196	280	282	110	110
05rbl.102	222	244	284	290	214	222	248	272	162	194	276	286	112	112
05rbl.108	214	226	284	292	212	218	260	288	208	208	298	304	110	110
05rbl.109	226	232	280	282	208	218	286	300	208	224	286	292	110	110
05rbl.117	212	218	286	290	202	214	294	294	150	162	290	296	110	110
05rbl.120	212	248	284	290	210	214	274	304	170	170	270	276	110	110
05rbl.121	218	224	290	290	216	226	268	298	200	208	274	288	110	110
05rbl.122	212	236	282	284	202	216	286	298	150	196	282	282	110	110
05rbl.123	212	214	282	284	212	216	272	300	170	170	288	290	110	110
05rbl.128	224	248	282	284	216	222	280	288	168	204	278	294	110	112
05rbl.134	214	248	286	290	214	216	300	300	150	198	266	286	110	110
05rbl.140	222	224	282	284	212	222	252	288	198	224	266	266	110	110
05rbl.144	214	258	282	290	212	214	274	292	214	214	282	296	110	110

05rbl.146	226	248	282	290	210	214	246	260	192	206	288	296	110	110
05rbl.147	214	224	286	286	214	218	266	294	208	220	284	286	110	112
05rbl.148	214	224	280	288	210	212	284	292	168	192	294	300	110	110
05rbl.149	214	224	282	290	214	214	272	298	194	196	288	290	110	110
05rbl.152	224	232	280	282	218	236	256	258	216	216	278	298	110	110
05rbl.154	216	226	282	290	210	212	298	304	190	202	266	278	110	110
05rbl.155	218	222	282	292	204	212	264	300	200	200	296	298	110	110
05rbl.156	212	220	290	292	212	214	264	300	180	208	284	300	110	110
05rbl.163	224	230	284	290	218	232	268	312	194	212	284	290	110	110
05rbl.164	252	258	290	292	218	222	256	304	208	212	284	286	110	110
05rbl.175	214	230	284	288	214	218	286	290	196	202	288	288	110	112
05rbl.180	212	224	282	286	214	214	280	298	182	196	272	272	110	110
05rbl.181	216	216	290	290	236	236	292	310	194	204	284	284	110	112
05rbl.183	226	254	282	288	210	232	270	282	212	212	284	288	110	110
05rbl.187	226	226	282	290	218	230	256	314	184	192	274	284	110	110
05rbl.190	224	244	286	286	210	214	256	282	172	216	284	286	110	110
05rbl.192	224	226	286	302	208	222	274	284	180	196	272	284	110	110
05rbl.198	218	224	284	292	208	218	268	314	194	204	266	270	110	110
05rbl.200	224	246	282	286	214	218	256	300	192	192	268	294	110	110
05rbl.201	226	226	284	286	216	218	310	312	192	216	274	284	110	110
05rbl.202	218	222	290	290	214	216	288	296	192	196	294	296	110	110
05rbl.206	224	228	284	292	214	214	296	300	184	196	290	296	110	110
05rbl.210	214	222	282	282	204	218	286	304	170	202	266	292	110	112
05rbl.211	218	248	284	286	214	222	258	292	150	214	288	290	110	114
05rbl.212	222	224	290	290	212	214	254	304	174	178	266	288	110	114
05rbl.215	222	232	282	282	210	218	254	274	170	198	268	276	110	112
05rbl.216	214	252	282	290	214	222	288	298	166	204	282	286	110	110
05rbl.220	212	218	288	290	212	218	266	286	170	196	290	294	110	112
05rbl.222	214	224	288	288	222	230	288	290	202	204	270	280	110	110
05rbl.223	214	224	282	286	202	218	286	286	190	202	268	280	110	112
05rbl.226	238	238	288	290	214	214	286	298	170	170	274	290	110	114
05rbl.233	222	224	280	280	222	228	266	278	198	214	286	286	110	112
05rbl.241	216	218	282	282	204	204	272	286	192	202	284	284	110	112
05rbl.242	214	224	286	290	210	214	300	310	180	204	280	292	110	110
05rbl.268	224	228	284	290	214	214	286	298	216	216	294	300	110	110
05rbl.270	224	230	282	286	212	214	286	288	170	192	286	308	110	110
05rbl.272	214	232	288	288	214	222	270	292	178	196	270	274	110	110

05rbl.273	224	228	282	286	210	214	270	296	206	206	276	294	110	114
05rbl.275	226	232	282	290	208	218	286	294	150	162	276	290	110	114
05rbl.284	224	224	284	290	214	218	256	260	150	214	262	276	110	110
05rbl.297	214	230	284	284	212	218	286	286	192	202	282	284	110	110
05rbl.298	216	224	284	284	210	216	280	294	208	208	288	288	114	114
05rbl.301	214	226	282	288	214	220	280	298	204	212	274	282	110	110
05rbl.302	222	222	286	290	214	236	282	292	170	192	282	298	110	110
05rbl.305	214	242	284	292	222	222	274	294	170	170	274	286	110	110
05rbl.306	222	228	284	286	204	214	272	294	194	202	268	296	110	110
05rbl.313	224	232	280	290	214	230	272	294	190	198	276	300	110	112
05rbl.314	226	250	284	290	214	218	274	294	150	170	270	274	110	110
05rbl.316	0	0	280	280	210	216	280	310	192	194	268	288	110	110
05rbl.320	222	230	284	286	214	216	254	284	168	170	274	292	110	112
05rbl.322	222	222	282	286	210	212	272	290	202	202	272	288	110	110
05rbl.324	212	214	284	286	214	222	272	314	0	0	274	284	110	114
05rbl.329	220	254	282	282	212	218	288	288	168	168	278	286	110	112
05rbl.331	214	238	290	290	202	214	272	282	170	222	286	288	110	110
05rbl.334	214	222	286	292	214	218	282	308	198	198	274	286	110	110
05rbl.343	226	248	284	286	214	218	294	306	168	176	266	288	110	110
05rbl.345	224	226	292	292	212	212	278	288	172	174	284	296	110	114
05rbl.349	224	226	288	290	204	212	274	298	198	198	290	298	110	112
05rbl.350	214	214	284	294	214	222	288	300	168	194	280	292	110	114
05rbl.351	214	232	284	284	218	218	278	296	170	170	280	298	110	110
05rbl.357	224	228	288	292	214	236	256	260	168	196	284	294	110	112
05rbl.362	222	224	288	288	210	222	282	290	196	196	288	292	110	110
05rbl.366	212	244	288	290	214	216	286	312	170	214	266	276	110	110
05rbl.367	216	226	284	288	214	216	290	314	176	202	282	284	114	114
05rbl.369	214	224	282	290	206	212	282	296	192	200	280	284	110	110
05rbl.370	224	230	282	286	204	208	252	294	190	226	280	284	110	110
05rbl.373	224	248	286	290	208	214	272	290	190	196	280	286	110	110
05rbl.377	214	214	282	288	214	214	254	276	150	170	288	292	110	110
05rbl.387	222	224	284	286	208	210	274	296	196	224	280	286	110	110
05rbl.390	226	226	290	290	214	222	274	274	196	196	276	282	110	112
05rbl.395	218	232	284	294	214	218	270	306	170	202	288	292	110	110
05rbl.398	224	224	290	290	218	230	278	296	174	174	282	282	112	112
05rbl.407	224	224	282	290	214	236	276	282	200	202	268	304	110	110
05rbl.408	214	218	288	288	210	226	284	300	178	204	286	300	110	110

05rbl.409	214	216	282	282	218	218	272	282	168	168	284	288	110	112
05rbl.414	220	224	282	282	214	218	286	286	168	204	288	290	110	112
05rbl.415	214	214	286	290	214	216	284	298	202	220	274	278	110	110
05rbl.416	224	224	284	288	212	214	274	274	166	170	288	290	110	112
05rbl.419	222	252	284	290	212	232	286	294	166	214	282	288	110	112
05rbl.421	214	224	282	282	214	218	280	298	168	182	276	300	110	110
05rbl.427	222	224	284	286	212	236	254	310	170	188	280	284	110	110
05rbl.429	212	212	284	292	210	218	220	284	170	208	284	310	110	110
06rbl.037	224	224	282	286	208	214	288	288	168	168	274	278	110	110
06rbl.043	224	254	282	292	212	212	286	290	170	208	280	296	110	112
06rbl.056	222	228	290	292	216	218	296	314	176	190	288	298	110	110
06rbl.057	224	224	284	290	210	214	292	296	170	170	296	296	110	112
06rbl.059	214	224	282	290	214	230	280	290	170	170	270	286	110	112
06rbl.061	226	228	290	292	216	218	294	296	194	194	270	290	110	112
06rbl.064	228	232	290	292	204	216	286	298	196	196	280	290	110	112
06rbl.075	214	224	284	284	212	214	274	290	150	172	272	300	110	110
06rbl.083	226	242	282	290	214	218	254	296	172	230	284	284	110	110
06rbl.087	214	226	282	286	208	214	292	296	168	172	278	296	110	112
06rbl.089	216	224	282	282	214	214	286	296	162	176	270	290	110	112
06rbl.090	212	224	282	282	210	222	280	282	150	200	284	302	110	110
06rbl.094	214	224	284	292	214	218	250	306	170	222	284	296	110	112
06rbl.095	232	232	280	286	208	216	270	276	150	220	276	284	110	110
06rbl.098	214	214	286	290	216	218	256	272	192	220	294	298	110	110
06rbl.103	226	226	282	290	214	214	284	308	168	172	286	292	110	112
06rbl.107	228	228	282	292	208	214	272	274	200	200	270	284	110	110
06rbl.109	224	224	280	286	218	218	282	290	190	206	274	284	110	110
06rbl.110	218	256	282	284	214	214	272	296	172	172	284	302	110	110
06rbl.119	224	228	282	288	214	214	256	284	170	194	280	286	110	110
06rbl.122	214	214	282	282	214	214	268	296	194	210	292	302	110	112
06rbl.127	224	252	284	286	214	214	272	286	204	204	280	282	110	112
06rbl.130	214	226	290	290	204	218	268	298	170	170	274	296	110	112
06rbl.133	232	232	290	290	216	222	286	292	168	212	276	282	110	112
06rbl.141	214	248	282	290	210	212	244	254	170	214	284	300	110	110
06rbl.144	226	228	286	290	214	214	284	288	192	200	266	290	110	110
06rbl.147	214	222	286	290	220	226	298	304	180	204	264	300	110	112
06rbl.158	220	226	282	284	204	210	270	290	176	200	280	290	110	112
06rbl.159	222	228	282	284	212	220	220	256	170	208	276	294	110	110

06rbl.161	214	234	282	290	214	214	294	298	178	200	262	288	110	112
06rbl.162	214	222	284	284	210	212	256	282	170	170	278	294	110	110
06rbl.163	222	234	284	290	204	214	270	284	150	204	288	290	110	112
06rbl.167	214	222	284	284	218	220	266	290	178	220	294	296	110	110
06rbl.168	214	222	284	290	210	220	256	270	166	176	278	294	110	112
06rbl.180	222	224	282	288	214	214	290	312	166	202	284	286	110	110
06rbl.187	214	214	284	290	220	222	282	286	200	204	274	298	110	110
06rbl.191	218	232	282	290	214	218	294	294	170	174	276	290	110	112
06rbl.201	226	226	282	286	214	218	286	292	192	212	268	290	110	114
06rbl.203	214	222	282	290	220	230	252	270	188	204	274	286	110	112
06rbl.206	224	252	286	290	214	222	252	290	168	192	290	310	110	110
06rbl.211	212	214	282	286	218	218	274	308	172	188	280	284	110	110
06rbl.231	224	230	290	290	216	220	286	286	168	194	294	294	110	110
06rbl.245	216	232	284	286	216	216	286	286	192	202	290	292	110	110
06rbl.247	218	218	282	290	204	236	300	304	172	172	288	292	110	110
06rbl.251	224	224	282	282	214	222	278	282	196	196	282	286	110	110
06rbl.252	212	224	282	286	214	218	274	284	198	198	270	302	110	110
06rbl.257	222	224	282	286	212	214	294	294	176	208	290	298	110	110
06rbl.258	222	226	282	286	212	218	278	300	170	198	286	304	110	110
06rbl.271	222	228	290	290	202	212	292	310	174	194	270	284	110	114
06rbl.273	222	226	286	290	204	214	252	274	184	198	272	286	110	112
06rbl.278	214	232	282	286	208	216	286	300	176	228	284	284	110	110
06rbl.283	220	222	282	290	212	216	274	274	170	194	286	294	110	110
06rbl.286	218	224	284	290	208	214	288	310	168	210	290	290	112	112
06rbl.288	228	232	282	284	216	222	282	298	170	202	278	284	110	110
06rbl.295	224	228	282	290	218	222	282	312	172	194	270	278	110	110
06rbl.301	234	252	284	284	210	212	284	292	170	192	268	276	110	110
06rbl.315	228	228	282	282	214	214	300	304	204	216	278	286	110	110
06rbl.317	216	224	290	290	210	210	256	274	202	214	292	302	110	112
06rbl.319	224	224	282	292	218	218	286	294	194	206	272	276	110	110
06rbl.320	218	232	282	282	212	214	272	306	216	226	272	282	110	110
06rbl.321	214	250	284	286	212	220	292	306	168	204	264	268	110	112
06rbl.322	218	230	282	286	214	214	290	300	170	196	276	282	110	110
06rbl.329	224	232	284	294	210	212	286	286	166	222	278	278	110	112
06rbl.336	214	226	284	286	210	214	272	284	190	202	276	280	110	112
06rbl.339	218	232	282	288	212	214	256	296	166	168	270	278	110	112
06rbl.341	214	224	282	286	216	218	252	296	166	174	278	298	110	112

06rbl.349	222	226	282	282	202	214	278	294	186	186	286	300	110	114
06rbl.355	224	224	282	282	210	228	272	284	198	226	280	292	110	114
06rbl.356	222	230	282	290	214	216	300	300	212	212	278	282	110	112
06rbl.372	226	230	286	286	212	214	298	298	214	214	282	296	110	112
06rbl.375	216	218	282	290	216	216	286	306	150	196	276	284	110	110
06rbl.382	228	248	290	290	208	210	312	314	150	150	276	278	114	114
06rbl.383	220	222	282	290	210	218	288	296	150	170	276	284	110	110
06rbl.386	220	222	286	286	208	214	296	296	168	196	284	292	110	116
06rbl.388	224	248	282	286	214	218	288	290	188	196	280	284	110	110
06rbl.389	224	226	282	282	214	230	304	308	172	172	300	300	110	114
06rbl.390	214	224	282	286	212	216	272	288	174	174	290	308	110	110
06rbl.391	222	226	282	290	214	214	288	288	188	200	284	292	110	112
06rbl.395	218	220	290	290	208	210	296	314	150	150	276	286	110	114
06rbl.397	220	222	284	288	210	216	288	314	196	212	278	280	110	116
06rbl.420	222	224	286	290	208	214	290	294	162	202	276	300	110	110
06rbl.421	224	226	282	282	208	214	246	282	170	206	286	290	110	112
06rbl.424	222	224	292	292	210	212	266	272	170	196	282	288	110	112
06rbl.452	214	222	282	284	214	218	274	292	166	214	276	280	110	110
06rbl.460	212	232	282	292	210	218	294	298	168	178	270	280	110	110
06rbl.469	222	222	286	292	216	230	260	274	168	200	284	302	110	110
06rbl.471	224	246	284	284	214	232	254	290	178	212	296	296	110	110
06rbl.475	228	228	282	282	214	218	272	292	170	170	268	292	110	110
06rbl.478	214	220	282	292	204	218	290	294	182	182	284	306	110	112
06rbl.481	246	246	282	282	216	232	272	282	150	170	276	298	110	110
06rbl.488	216	222	284	284	216	228	292	312	170	170	262	276	110	110
06rbl.495	214	226	284	286	212	218	282	288	168	202	292	308	110	110
06rbl.501	214	238	282	282	214	216	264	286	202	202	266	290	110	110
06rbl.508	226	232	282	290	210	218	290	304	172	172	282	294	110	114
06rbl.509	214	218	286	290	214	218	286	292	196	196	284	288	110	110
06rbl.515	222	222	292	292	216	218	254	292	190	190	274	290	110	110
06rbl.522	224	224	282	282	214	218	284	286	170	192	282	294	110	110
06rbl.526	212	216	284	292	206	214	284	288	194	194	276	288	110	110
06rbl.534	220	238	282	290	214	218	284	290	172	196	290	292	110	110
06rbl.536	214	224	282	290	216	220	292	306	168	200	288	290	110	110
06rbl.545	212	214	282	290	212	218	294	306	168	196	280	286	110	110
06rbl.548	224	224	282	290	212	212	294	306	202	202	284	286	110	110
06rbl.551	218	224	286	286	218	220	274	274	194	196	272	286	110	110

06rbl.552	214	226	288	290	210	214	286	292	168	206	268	306	110	112
06rbl.553	224	224	282	286	212	218	214	288	170	178	282	282	110	112
06rbl.565	216	222	284	290	214	214	264	286	202	208	280	304	110	112
06rbl.568	222	222	282	284	214	216	254	278	166	202	274	288	110	112
06rbl.577	214	224	282	290	214	218	288	288	202	206	274	282	110	110
06rbl.579	212	218	282	286	214	214	290	298	190	190	272	290	110	112
06rbl.580	218	224	282	286	214	232	272	306	186	188	286	294	110	114
06rbl.581	224	224	284	294	214	230	292	294	166	166	272	276	110	110
06rbl.583	222	226	282	282	214	214	280	282	216	216	286	292	110	110
06rbl.584	226	230	288	292	208	236	286	310	150	150	284	294	110	112
06rbl.588	246	256	286	286	210	214	288	290	150	194	282	296	110	110
06rbl.590	228	250	286	292	214	220	304	304	174	196	272	288	110	110
06rbl.598	224	224	282	282	214	218	272	294	196	214	284	286	110	110
06rbl.600	224	224	284	290	214	222	266	294	150	210	276	276	110	110
06rbl.611	214	222	282	286	214	216	252	298	174	174	280	298	110	112
06rbl.623	218	226	282	290	210	218	274	290	200	200	266	282	110	112
06rbl.628	214	224	282	292	216	218	250	264	174	216	266	266	110	112
07rbl.001	212	224	282	292	216	218	290	298	178	220	274	288	110	110
07rbl.003	224	226	282	288	202	202	252	292	174	202	270	288	110	112
07rbl.007	214	228	282	290	214	220	266	282	170	186	268	282	110	110
07rbl.008	214	226	286	292	218	236	256	276	150	192	292	294	110	110
07rbl.009	224	224	282	288	208	214	288	300	150	200	288	300	110	110
07rbl.010	218	224	286	290	212	212	300	304	150	216	266	294	110	112
07rbl.014	226	244	286	286	214	218	268	272	188	200	284	286	110	112
07rbl.015	224	224	282	284	214	236	272	304	180	196	272	300	110	112
07rbl.016	224	224	284	286	202	234	258	292	170	210	294	298	110	110
07rbl.019	216	228	286	290	210	216	220	300	170	210	286	288	110	110
07rbl.021	224	232	282	282	214	214	272	304	196	196	278	294	110	110
07rbl.023	224	226	284	288	214	216	272	284	196	208	282	286	112	112
07rbl.024	214	216	286	290	214	218	272	286	200	212	274	296	110	110
07rbl.028	214	226	286	290	212	212	296	298	172	172	284	292	110	110
07rbl.029	224	226	286	286	210	224	312	312	172	196	286	294	110	112
07rbl.033	214	238	282	290	212	214	286	290	168	212	274	282	110	110
07rbl.045	214	218	282	290	214	214	280	294	166	174	280	286	110	112
07rbl.051	214	226	282	284	212	212	252	268	188	208	272	286	112	112
07rbl.054	214	226	288	290	212	218	254	254	150	150	284	298	110	112
07rbl.055	212	224	286	286	210	214	250	290	150	170	282	284	110	110

07rbl.057	212	228	292	292	208	222	252	286	170	170	270	286	110	114
07rbl.058	214	214	290	290	212	214	278	292	176	196	274	296	110	112
07rbl.060	222	248	282	282	214	218	272	304	182	208	286	294	110	112
07rbl.063	212	222	286	290	208	214	256	290	150	160	262	290	110	112
07rbl.067	222	222	282	286	202	212	284	302	150	206	272	278	110	110
07rbl.069	212	218	288	290	218	222	256	280	208	214	272	288	110	112
07rbl.070	222	222	282	282	212	222	256	302	170	180	278	288	112	112
07rbl.072	222	228	282	286	212	214	306	306	172	196	286	310	110	110
07rbl.073	214	230	288	290	214	236	292	292	186	214	262	296	110	112
07rbl.076	224	236	282	290	214	214	272	288	166	172	298	310	110	110
07rbl.078	212	224	286	290	212	228	286	300	150	214	266	282	110	110
07rbl.080	214	218	282	282	208	214	292	300	172	212	274	302	110	110
07rbl.082	224	226	282	286	208	222	260	286	174	196	266	286	110	112
07rbl.083	232	232	282	290	218	222	278	312	198	206	274	284	110	110
07rbl.085	224	228	282	292	202	214	252	298	150	170	292	298	110	110
07rbl.089	224	224	290	290	222	222	268	288	150	204	270	298	110	112
07rbl.091	214	214	286	288	218	218	292	304	200	212	270	280	110	114
07rbl.094	228	228	282	286	214	214	256	270	190	204	270	280	110	110
07rbl.095	222	224	284	290	210	222	288	306	178	216	272	292	112	112
07rbl.097	222	226	286	294	208	214	288	292	194	202	290	294	110	110
07rbl.099	212	214	282	290	214	220	290	300	150	168	282	300	110	112
07rbl.106	226	226	288	294	214	216	288	292	168	198	290	298	110	110
07rbl.110	212	224	282	286	214	218	256	270	206	208	274	292	110	110
07rbl.111	212	218	284	286	212	214	286	290	202	206	292	300	110	110
07rbl.113	214	228	290	292	210	218	280	300	172	208	270	286	110	110
07rbl.118	214	224	282	292	214	220	288	304	150	184	288	294	110	112
07rbl.122	226	226	290	292	202	214	274	290	194	206	280	294	110	110
07rbl.123	224	232	282	298	210	212	286	294	174	202	282	290	112	112
07rbl.124	214	226	282	284	208	214	272	294	166	178	262	280	110	112
07rbl.125	212	232	282	284	212	214	290	298	212	216	280	296	110	110
07rbl.130	218	226	286	292	214	214	214	308	196	200	272	282	110	110
07rbl.132	214	224	282	286	214	214	298	302	168	192	284	296	110	110
07rbl.136	214	224	280	290	210	212	256	286	168	204	266	266	110	110
07rbl.139	224	230	284	284	214	218	256	286	192	192	280	294	110	110
07rbl.144	220	224	282	290	210	218	292	304	172	204	266	292	110	110
07rbl.152	224	224	282	290	214	216	220	272	190	208	268	278	110	110
07rbl.158	224	224	282	292	210	218	282	298	192	202	288	296	110	110

07rbl.159	222	232	282	290	208	214	278	280	188	202	270	272	110	110
07rbl.164	212	238	290	290	216	216	276	308	150	190	286	288	112	114
07rbl.167	222	224	288	290	214	218	274	286	166	192	280	306	110	112
07rbl.168	222	224	290	292	214	218	258	312	150	170	280	282	110	110
07rbl.171	214	224	288	292	210	226	286	298	178	200	278	286	110	110
07rbl.179	224	248	288	288	214	214	276	290	168	208	266	276	110	110
07rbl.180	222	226	286	286	218	230	280	286	174	188	288	298	110	112
07rbl.182	214	226	286	286	218	222	274	290	170	214	284	286	110	112
07rbl.183	228	232	282	286	210	212	262	286	168	216	280	290	110	110
07rbl.186	220	222	282	290	212	228	274	300	176	192	280	292	110	110
07rbl.187	226	230	282	288	218	232	268	278	196	212	284	286	110	110
07rbl.188	214	224	282	286	214	218	298	298	198	204	268	286	110	110
07rbl.189	226	228	284	286	214	214	306	314	194	202	290	296	110	114
07rbl.192	216	226	284	292	204	214	250	304	198	214	288	288	110	112
07rbl.195	222	224	282	288	212	212	256	294	170	174	278	284	110	110
07rbl.196	214	232	290	296	210	214	256	282	190	198	284	290	110	110
07rbl.203	246	254	290	292	216	232	262	284	170	196	282	286	110	110
07rbl.205	228	254	282	290	218	218	266	284	212	212	282	288	110	110
07rbl.210	216	224	284	292	236	236	256	258	174	196	272	290	110	112
07rbl.211	224	244	286	292	214	220	252	314	150	178	298	298	110	114
07rbl.212	222	226	286	292	214	216	272	290	150	166	288	298	110	110
07rbl.213	224	224	284	286	210	214	286	288	150	168	282	296	112	114
07rbl.214	224	228	282	286	204	214	292	298	182	204	268	280	110	110
07rbl.215	218	228	286	292	214	218	290	302	174	174	290	290	110	112
07rbl.217	224	224	288	290	214	222	284	302	168	198	280	302	110	112
07rbl.219	224	252	282	292	216	222	282	304	208	208	280	282	110	110
07rbl.220	224	226	288	290	202	218	292	312	194	206	270	276	110	112
07rbl.221	214	222	282	282	210	230	254	288	174	174	288	300	110	110
07rbl.225	224	230	282	282	216	222	272	306	176	186	288	296	110	110
07rbl.229	224	226	284	286	218	218	262	266	150	198	278	288	110	110
07rbl.233	224	226	286	292	214	222	290	300	170	182	274	294	110	110
07rbl.235	224	228	286	290	208	218	270	284	150	190	284	296	110	112
07rbl.236	228	232	282	286	208	218	268	294	168	202	284	292	110	112
07rbl.238	222	226	282	284	214	222	274	274	200	216	284	288	110	110
07rbl.240	222	234	286	286	216	218	294	304	150	178	282	286	110	112
07rbl.242	224	230	284	290	212	218	286	312	198	210	274	284	110	110
07rbl.243	222	224	280	282	214	218	282	304	150	168	290	290	110	112

07rbl.245	214	224	290	292	214	218	216	272	150	168	266	286	110	110
07rbl.251	224	224	282	286	216	218	258	286	150	178	266	276	110	110
07rbl.255	226	230	282	286	210	212	298	298	186	192	272	272	110	112
07rbl.258	224	224	280	280	202	202	296	304	170	190	274	274	110	112
07rbl.259	226	226	284	284	210	234	250	286	168	210	288	294	110	110
07rbl.262	222	226	290	290	216	220	268	302	172	178	294	300	110	110
07rbl.264	224	224	282	282	214	218	284	288	168	210	296	300	110	114
07rbl.266	222	230	284	286	208	218	294	296	174	196	280	286	110	112
07rbl.269	222	230	282	282	216	222	252	296	204	222	274	298	110	112
07rbl.271	224	228	290	298	208	210	252	292	174	174	266	280	112	112
07rbl.272	214	218	282	286	210	214	270	290	170	176	286	286	110	110
07rbl.275	220	224	290	290	218	218	284	284	160	170	280	290	110	110
07rbl.278	212	218	290	290	202	218	252	272	186	202	262	284	110	110
07rbl.279	224	224	284	284	220	228	252	294	198	198	276	280	110	112
07rbl.281	214	222	290	290	214	218	272	312	168	198	290	292	110	110
07rbl.284	224	248	290	292	218	236	252	272	206	206	288	302	110	110
07rbl.286	218	222	282	292	204	218	264	304	172	194	268	286	110	112
07rbl.290	212	224	282	282	206	218	260	274	200	212	284	308	110	110
07rbl.295	218	226	286	290	212	214	288	298	194	220	270	276	110	110
07rbl.296	212	228	286	286	212	214	220	290	188	208	290	302	110	110
07rbl.300	222	224	286	290	208	214	282	290	150	160	276	284	110	110
07rbl.301	222	224	290	290	214	230	280	282	150	160	264	276	110	110
07rbl.302	226	228	282	288	214	222	286	290	206	208	288	292	110	112
07rbl.303	218	224	282	290	216	222	248	272	162	168	270	288	110	112
07rbl.304	216	226	284	292	218	218	288	300	168	190	284	284	110	110
07rbl.309	214	224	290	290	212	214	250	302	202	204	284	288	110	110
08rbl.011	214	228	284	288	208	210	256	304	150	192	282	300	110	112
08rbl.015	226	246	282	284	212	214	270	298	182	200	268	282	110	110
08rbl.020	214	228	282	286	208	226	256	256	194	220	276	294	112	112
08rbl.021	222	224	282	288	214	218	276	314	170	196	280	284	110	110
08rbl.023	214	218	282	290	214	220	252	294	174	178	280	296	110	110
08rbl.025	222	224	282	290	212	214	284	304	220	220	276	278	110	110
08rbl.027	224	232	282	288	214	218	276	284	170	190	290	294	110	110
08rbl.033	228	228	282	284	210	220	282	306	170	200	276	294	110	110
08rbl.034	232	232	284	292	212	212	298	310	174	182	272	278	110	110
08rbl.041	216	224	284	292	214	220	252	298	170	174	274	278	110	110
08rbl.045	224	228	284	286	214	220	268	292	170	210	286	296	110	110

08rbl.054	226	228	282	282	212	230	254	312	168	174	276	300	110	110
08rbl.055	218	226	288	292	212	218	278	290	162	192	270	282	110	110
08rbl.059	226	248	290	292	212	214	260	294	168	182	266	296	110	110
08rbl.061	212	218	284	284	208	208	268	300	150	198	274	292	110	112
08rbl.064	226	228	286	286	214	224	252	264	168	192	286	286	112	112
08rbl.070	226	254	292	292	208	214	268	282	188	198	276	300	110	110
08rbl.074	214	226	286	292	210	216	280	288	170	200	274	296	110	110
08rbl.079	226	230	284	284	214	218	284	306	0	0	294	302	110	110
08rbl.081	214	224	288	290	216	220	284	298	194	202	282	288	110	110
08rbl.085	228	258	284	284	210	216	270	304	168	224	276	290	110	114
08rbl.124	224	252	286	288	212	218	286	286	166	186	298	300	110	112
08rbl.129	224	228	290	302	208	212	272	298	178	180	270	290	110	112
08rbl.144	222	224	282	290	214	218	264	274	172	228	266	296	110	114
08rbl.153	226	248	282	282	208	214	304	304	174	182	284	284	110	110
08rbl.155	224	232	280	292	222	222	276	276	204	216	282	284	110	110
08rbl.156	212	230	282	290	214	214	256	294	200	224	270	282	110	110
08rbl.159	218	224	286	290	202	214	268	298	170	174	284	294	110	112
08rbl.162	224	224	282	288	212	216	284	298	170	174	268	286	110	114
08rbl.165	212	224	284	284	208	214	272	274	170	194	280	280	110	110
08rbl.167	224	230	284	302	218	220	288	290	184	200	276	280	110	110
08rbl.170	218	226	284	284	216	222	294	294	174	204	286	286	110	110
08rbl.174	218	226	284	288	218	222	286	298	170	186	280	284	110	112
08rbl.175	216	228	284	288	212	236	286	292	170	200	270	298	110	110
08rbl.176	218	228	282	292	204	218	268	300	196	202	266	266	110	110
08rbl.177	224	226	290	302	208	214	272	300	170	178	296	296	110	110
08rbl.182	214	258	290	290	202	214	286	292	176	212	264	264	110	110
08rbl.189	222	228	282	284	212	218	286	288	208	226	286	294	110	112
08rbl.196	224	224	284	284	214	222	282	306	150	202	292	292	110	112
08rbl.210	224	224	284	290	202	214	266	304	162	216	286	286	112	112
08rbl.211	232	246	282	286	210	218	252	306	170	202	266	278	110	110
08rbl.215	222	224	282	290	212	214	268	304	202	212	292	298	110	110
08rbl.225	212	224	284	284	206	232	290	294	202	212	266	298	110	112
08rbl.231	224	230	282	284	212	220	298	304	174	176	288	288	110	110
08rbl.242	226	226	284	286	214	218	272	274	178	220	262	296	110	110
08rbl.244	214	226	284	286	204	214	282	292	178	216	274	286	110	110
08rbl.245	212	224	282	292	208	214	294	296	170	194	276	276	110	114
08rbl.248	214	214	286	290	202	218	268	300	178	178	282	286	110	110

08rbl.260	222	224	284	292	208	214	292	292	168	170	294	294	112	112
08rbl.277	224	232	284	284	204	214	256	262	174	192	288	290	110	110
08rbl.284	224	224	282	282	212	226	260	260	150	196	282	288	110	110
08rbl.289	214	218	282	284	214	224	266	292	192	202	280	296	110	110
08rbl.295	214	228	284	292	218	218	220	294	190	190	274	288	110	110
08rbl.302	224	248	286	290	216	222	220	288	172	202	268	290	110	110
08rbl.303	224	226	284	288	216	236	282	298	174	202	270	282	110	110
08rbl.308	226	228	282	288	212	222	284	284	166	214	280	306	110	114
08rbl.313	212	216	286	292	212	222	270	282	174	214	266	306	110	114
08rbl.315	224	224	288	290	210	216	278	282	200	202	270	284	110	110
08rbl.318	216	226	292	292	218	220	300	300	150	194	282	298	110	110
08rbl.325	224	248	284	284	214	214	252	274	150	184	286	298	110	110
08rbl.327	226	226	286	286	212	212	252	290	194	202	266	290	110	112
08rbl.337	224	228	286	286	214	216	286	290	166	202	278	288	110	112
08rbl.347	224	238	282	290	204	214	288	288	168	170	278	284	110	110
08rbl.356	222	224	282	282	218	218	282	300	194	200	268	286	110	110
08rbl.360	236	254	300	300	214	214	286	286	186	200	262	274	110	112
08rbl.363	218	226	282	284	216	222	274	286	172	182	266	286	110	110
08rbl.368	226	254	288	292	204	218	286	286	170	192	266	274	110	112
08rbl.371	226	254	284	290	212	222	214	214	170	206	274	286	110	112
08rbl.374	224	228	284	290	214	214	272	276	168	190	278	292	110	110
08rbl.380	214	228	284	290	216	218	272	288	198	214	280	288	110	112
08rbl.384	214	216	286	292	216	220	278	278	168	170	264	270	110	110
08rbl.397	214	224	284	292	212	218	290	304	188	190	286	290	110	110
08rbl.406	214	226	286	292	218	218	290	290	168	198	288	304	110	110
08rbl.412	222	226	288	290	214	218	256	290	204	204	284	286	110	110
08rbl.415	228	228	288	288	220	220	0	0	168	168	272	276	110	110
08rbl.418	224	228	286	292	216	222	290	290	200	200	278	288	110	110
08rbl.425	218	222	282	292	214	218	274	308	150	194	284	290	110	110
08rbl.430	222	224	286	292	216	220	250	250	168	202	270	302	110	110
08rbl.442	216	216	282	292	206	218	256	290	170	178	272	272	110	110
08rbl.446	218	232	284	286	214	224	270	292	204	206	286	302	110	110
08rbl.447	226	234	284	294	212	222	284	304	184	220	282	292	110	110
08rbl.449	216	246	286	286	214	220	280	300	192	198	268	282	110	110
08rbl.450	246	252	286	292	212	214	294	298	168	198	270	296	110	110
08rbl.459	216	216	284	294	202	222	286	312	204	206	284	300	110	110
08rbl.466	216	246	284	292	214	218	286	294	168	208	270	296	110	112

08rbl.467	220	224	286	290	204	222	278	284	170	170	266	288	110	110
08rbl.470	222	224	282	292	214	236	286	294	190	190	298	306	110	110
08rbl.475	224	224	288	292	214	218	290	298	150	208	270	298	110	110
08rbl.482	224	224	282	282	216	218	300	304	192	216	292	300	110	110
08rbl.487	212	224	286	288	228	230	290	296	150	202	276	296	110	110
08rbl.493	212	224	292	292	228	230	290	296	208	216	290	294	110	110
08rbl.497	226	254	288	294	214	236	288	292	162	196	276	304	110	110
08rbl.502	212	228	282	284	214	220	268	282	170	192	282	288	110	110
08rbl.508	224	224	284	288	214	234	270	290	208	210	288	304	110	114
08rbl.509	212	224	282	290	208	214	292	294	208	212	274	288	110	110
08rbl.511	216	238	292	292	214	218	244	298	172	172	290	298	110	110
08rbl.513	224	234	282	290	216	216	282	292	0	0	280	300	110	112
08rbl.516	224	228	284	292	216	236	258	286	168	176	288	296	110	110
08rbl.523	226	228	284	284	216	236	288	294	170	192	280	298	110	110
08rbl.530	214	226	284	292	212	214	298	298	178	188	284	284	110	110
08rbl.531	238	254	286	290	210	210	266	270	172	196	292	302	110	110
08rbl.532	218	236	282	284	214	222	270	300	198	206	276	290	110	112
08rbl.534	216	228	282	282	208	208	258	284	186	194	290	302	110	110
08rbl.542	214	218	284	290	202	204	276	298	196	228	280	302	110	110
08rbl.553	218	232	290	292	214	230	256	290	150	206	300	302	110	110
08rbl.554	224	224	288	292	216	218	252	282	150	168	270	278	110	112
08rbl.565	224	250	286	286	204	232	292	308	170	170	290	310	110	112
08rbl.569	228	232	284	284	214	216	266	284	174	196	262	278	110	112
08rbl.573	212	214	284	286	204	210	270	286	170	190	282	282	110	110
08rbl.574	216	226	282	286	210	210	272	272	170	190	290	304	110	110
08rbl.608	216	226	286	288	218	222	272	274	170	170	288	290	110	110
08rbl.613	214	222	280	286	212	220	292	304	170	176	294	310	110	110
08rbl.614	214	222	288	292	218	230	296	304	170	202	274	294	110	110
08rbl.619	218	224	280	286	214	214	272	296	168	202	276	302	110	110
08rbl.620	214	220	290	292	204	222	286	292	188	190	282	282	110	112
08rbl.621	238	252	282	286	210	212	298	302	196	206	282	282	110	112
08rbl.624	214	224	286	292	212	214	310	310	150	222	266	290	110	110
08rbl.625	224	224	286	290	208	208	298	298	150	170	268	276	110	110
08rbl.628	212	218	286	286	202	214	288	292	170	208	276	284	110	110
08rbl.629	214	218	282	290	214	214	272	290	170	196	288	302	110	110
09rbl.001	212	212	290	290	218	222	256	264	200	208	274	282	110	110
09rbl.004	218	224	282	290	214	216	272	288	150	170	270	280	110	112

09rbl.005	214	228	282	286	212	236	298	310	170	196	278	290	110	110
09rbl.007	218	224	280	290	212	218	280	298	178	214	284	290	112	112
09rbl.009	228	228	284	286	210	218	220	284	198	208	284	288	110	110
09rbl.013	224	224	282	284	214	216	262	314	150	200	284	296	110	114
09rbl.014	214	226	284	284	210	222	300	300	150	150	296	300	110	110
09rbl.018	226	228	284	284	214	218	254	298	150	172	266	274	110	110
09rbl.026	226	226	288	290	214	232	262	294	170	194	276	284	110	112
09rbl.030	218	228	286	290	212	218	282	294	150	214	276	294	110	110
09rbl.031	228	228	284	288	214	218	260	294	168	202	278	300	110	112
09rbl.032	224	224	284	290	214	230	260	314	150	182	282	296	110	114
09rbl.033	224	226	286	288	212	214	256	276	170	192	286	300	110	110
09rbl.036	224	224	282	284	214	218	294	298	150	150	276	300	110	112
09rbl.040	224	224	282	282	208	208	278	300	192	214	264	276	110	110
09rbl.043	216	226	282	286	218	218	254	288	174	196	288	294	112	112
09rbl.045	224	228	282	284	214	228	286	314	200	202	280	298	110	114
09rbl.047	214	226	286	286	210	218	254	298	174	186	288	298	110	110
09rbl.048	214	224	282	282	218	218	260	288	174	196	286	294	112	112
09rbl.082	214	252	282	282	216	222	262	298	166	168	292	296	110	110
09rbl.086	222	222	282	298	214	214	282	286	174	206	276	304	110	112
09rbl.092	228	228	286	290	210	212	282	298	170	190	272	276	110	110
09rbl.105	224	232	290	290	214	214	288	298	170	200	274	296	110	110
09rbl.109	222	224	282	286	214	218	296	296	150	186	278	282	110	110
09rbl.111	214	232	282	282	210	212	290	294	170	200	278	294	110	110
09rbl.112	228	252	282	292	218	218	266	288	172	182	276	292	110	112
09rbl.113	224	252	290	290	218	218	288	312	168	194	288	290	110	110
09rbl.117	228	228	282	282	214	236	276	310	192	214	284	300	110	110
09rbl.118	222	228	290	290	214	218	256	288	168	172	284	302	110	110
09rbl.121	214	214	282	286	214	216	272	282	150	178	262	268	110	110
09rbl.123	222	228	282	282	214	218	276	282	214	216	266	282	110	110
09rbl.125	214	224	280	282	214	216	284	296	168	202	280	300	110	110
09rbl.133	224	224	286	290	210	218	282	294	188	190	274	296	110	110
09rbl.134	218	224	286	288	212	216	260	308	150	194	276	288	110	112
09rbl.137	214	214	282	292	214	218	252	298	202	224	294	302	112	114
09rbl.139	224	226	286	290	214	216	252	278	206	212	272	278	110	110
09rbl.153	214	226	286	290	214	214	272	272	168	214	280	302	110	112
09rbl.157	212	228	282	290	214	214	272	282	190	206	266	286	110	110
09rbl.159	222	226	282	284	202	212	288	290	194	206	274	284	110	112

09rbl.167	214	226	286	290	218	230	282	298	170	222	276	278	110	112
09rbl.168	214	226	286	288	214	218	256	288	168	204	284	294	110	112
09rbl.170	228	238	284	290	214	214	284	292	166	206	272	286	110	110
09rbl.171	224	226	284	292	210	218	286	312	150	190	288	294	110	110
09rbl.173	224	238	282	284	208	218	296	310	172	222	274	288	110	110
09rbl.186	224	232	284	286	212	218	252	296	196	220	274	292	110	112
09rbl.190	214	238	282	290	218	222	274	284	168	210	282	292	112	112
09rbl.203	224	224	282	290	218	220	252	264	174	198	292	294	110	110
09rbl.207	222	226	282	286	204	212	272	310	166	172	266	288	110	110
09rbl.209	224	224	284	286	212	224	280	286	168	168	262	288	110	110
09rbl.211	212	252	290	292	212	214	264	284	208	224	278	296	110	110
09rbl.214	224	226	282	284	202	212	274	288	170	192	276	280	110	110
09rbl.219	214	224	282	288	214	214	274	308	192	222	274	310	112	112
09rbl.222	224	224	284	286	212	214	300	300	186	206	286	286	110	110
09rbl.228	214	224	282	288	212	214	304	314	170	184	274	274	110	110
09rbl.232	224	226	282	286	212	218	288	298	166	194	268	276	110	110
09rbl.233	222	222	282	288	204	210	292	300	168	194	288	288	110	110
09rbl.234	224	224	284	290	210	222	274	304	172	214	302	302	110	110
09rbl.235	222	224	282	284	214	222	248	284	162	198	270	284	110	112
09rbl.244	214	246	290	290	214	218	248	294	162	166	270	282	110	112
09rbl.245	224	228	290	290	212	230	288	294	196	216	262	300	110	110
09rbl.248	214	224	284	290	210	214	272	300	172	190	282	294	110	110
09rbl.253	214	230	282	284	202	218	286	292	168	196	280	290	110	112
09rbl.254	224	248	282	286	214	230	274	294	166	210	274	276	110	110
09rbl.256	222	224	282	282	216	216	272	300	170	194	274	276	110	110
09rbl.266	212	238	284	288	212	214	254	290	170	178	280	288	110	112
09rbl.269	218	228	282	290	216	220	284	302	180	198	270	270	110	110
09rbl.270	228	244	282	284	208	214	298	298	168	204	266	284	110	112
09rbl.276	224	224	286	288	218	218	270	270	186	196	268	268	110	110
09rbl.284	214	248	282	290	210	214	256	262	168	170	284	286	110	112
09rbl.289	218	224	284	290	218	218	268	284	172	172	276	288	110	110
09rbl.295	214	222	282	290	210	218	288	302	204	204	284	286	110	112
09rbl.297	222	238	282	284	210	212	286	286	190	214	274	286	110	110
09rbl.299	216	232	286	290	214	216	294	302	196	224	286	286	110	112
09rbl.309	218	228	286	286	216	236	272	290	166	196	286	298	110	110
09rbl.314	222	224	282	282	212	232	272	284	188	206	282	298	110	112
09rbl.316	212	224	284	292	214	232	282	290	180	202	280	292	110	110

09rbl.319	222	224	286	290	210	218	272	294	150	172	276	298	110	110
09rbl.320	222	226	286	288	214	222	266	308	176	196	282	292	110	110
09rbl.321	222	224	282	282	214	222	254	254	196	208	274	286	110	110
09rbl.323	212	226	282	290	218	222	290	304	172	200	266	286	110	110
09rbl.327	214	238	286	288	210	212	272	288	168	226	268	284	110	110
09rbl.331	242	244	282	284	208	232	272	292	166	170	282	288	110	112
09rbl.333	218	224	282	290	204	218	278	286	174	192	262	292	110	112
09rbl.350	224	256	282	288	212	226	290	306	170	178	274	288	110	110
09rbl.364	214	226	282	284	212	214	280	280	202	206	284	290	110	112
09rbl.371	214	224	286	290	218	230	268	294	192	196	268	300	110	110
09rbl.372	222	222	284	288	214	222	252	274	168	186	288	300	110	112
09rbl.380	212	236	282	286	212	214	256	272	170	172	278	282	110	110
09rbl.385	214	222	284	288	212	218	294	304	166	204	278	302	110	110
09rbl.391	214	218	282	286	216	222	304	312	180	194	266	300	110	110
09rbl.394	232	242	286	288	212	214	272	292	150	196	266	282	110	112
09rbl.401	214	224	286	286	214	214	284	284	204	204	274	300	110	112
09rbl.406	212	232	282	292	214	218	252	300	170	204	276	288	110	112
09rbl.413	228	238	290	290	216	218	256	300	162	190	284	296	110	110
09rbl.415	212	228	282	284	214	218	296	302	168	202	290	298	110	110
09rbl.418	232	232	286	290	214	214	300	304	194	214	266	266	110	110
09rbl.420	224	226	282	284	212	218	270	290	170	204	278	292	110	112
09rbl.425	228	232	282	288	202	202	302	304	202	206	294	296	110	112
09rbl.434	224	232	282	290	212	216	284	292	168	210	276	282	110	110
09rbl.435	218	226	284	286	206	218	296	298	170	170	268	300	110	110
09rbl.440	224	226	282	290	214	230	274	274	198	198	290	298	110	110
09rbl.442	220	232	286	290	210	214	268	306	150	186	290	290	110	110
09rbl.447	222	228	286	286	214	214	264	292	192	208	264	282	110	110
09rbl.453	226	234	290	290	202	214	286	294	202	206	276	278	110	110
09rbl.455	214	244	284	286	204	220	278	292	196	196	276	292	110	110
09rbl.460	226	238	282	290	216	232	300	304	170	172	282	300	110	114
09rbl.462	212	222	290	290	214	214	274	290	168	212	292	302	110	110
09rbl.463	224	232	282	290	212	214	276	296	192	202	282	288	110	112
09rbl.466	214	222	282	282	202	214	304	304	168	202	286	300	110	110
09rbl.468	228	234	282	290	214	218	300	300	150	150	280	298	110	112
09rbl.476	214	222	284	290	216	218	256	302	172	172	284	294	110	112
09rbl.490	224	226	282	290	208	214	290	294	196	196	270	274	110	112
09rbl.494	222	230	282	286	204	216	272	304	170	184	282	294	110	110

09rbl.501	224	224	286	290	212	218	264	288	200	200	274	292	110	110
09rbl.503	214	222	290	290	210	218	288	294	170	176	286	286	110	110
09rbl.510	254	254	282	286	208	214	266	286	172	196	266	302	110	110
09rbl.511	224	226	286	290	214	222	258	308	168	170	276	298	110	114
09rbl.514	224	228	282	286	204	218	298	308	172	202	268	300	110	114
09rbl.516	222	226	282	290	208	210	274	288	176	196	292	298	110	112
09rbl.518	212	218	286	290	212	230	266	282	196	202	292	302	110	112
10rbl.001	214	232	282	290	218	218	252	260	174	208	272	288	110	112
10rbl.004	226	252	290	290	218	236	256	272	192	194	272	278	110	110
10rbl.006	226	228	288	292	218	218	220	292	188	192	278	290	110	112
10rbl.007	224	232	286	290	214	218	272	276	170	192	276	288	110	110
10rbl.008	222	232	282	288	218	236	256	294	204	206	272	278	110	112
10rbl.016	222	226	288	288	218	232	290	292	192	194	272	278	110	112
10rbl.017	226	226	286	286	214	218	272	288	170	206	278	288	110	112
10rbl.018	226	228	290	290	218	218	294	312	192	192	278	288	110	110
10rbl.019	218	230	282	286	212	216	292	302	174	208	272	284	110	110
10rbl.021	224	230	282	290	212	214	296	300	178	208	268	288	110	110
10rbl.028	214	224	282	282	208	212	220	294	178	178	270	280	110	110
10rbl.031	222	236	282	290	216	218	290	290	168	226	266	278	110	112
10rbl.035	244	252	292	292	220	232	274	308	170	200	288	302	110	110
10rbl.038	224	228	282	282	204	210	290	296	194	196	288	298	110	110
10rbl.049	214	222	282	282	214	218	264	274	170	172	278	290	110	110
10rbl.056	216	252	282	286	216	218	260	300	150	190	268	286	112	112
10rbl.058	226	232	282	290	210	216	268	286	170	222	288	288	110	110
10rbl.060	224	228	282	282	216	222	294	294	170	208	286	286	110	110
10rbl.064	222	228	286	290	214	218	256	290	190	190	270	298	110	112
10rbl.065	218	232	282	282	216	222	294	302	184	212	274	296	110	112
10rbl.066	214	232	282	292	214	222	294	308	170	210	284	306	110	110
10rbl.067	224	230	288	288	210	212	290	298	190	190	286	290	110	110
10rbl.081	230	242	284	290	212	230	272	272	168	216	286	290	110	112
10rbl.082	224	252	282	290	210	214	272	294	174	206	272	276	110	112
10rbl.083	220	228	282	290	216	222	290	304	172	172	268	298	110	112
10rbl.084	218	222	286	286	214	232	274	286	150	170	290	292	110	110
10rbl.088	224	228	284	290	214	214	264	298	150	196	282	282	110	110
10rbl.091	214	236	282	290	214	218	270	280	170	202	280	296	110	110
10rbl.095	226	226	282	286	214	218	282	312	168	202	286	294	110	110
10rbl.099	222	242	282	286	214	214	286	304	172	194	268	270	110	110

10rbl.112	222	258	282	284	214	222	280	290	170	178	276	276	110	112
10rbl.115	214	230	282	290	208	214	256	292	150	198	274	284	110	112
10rbl.117	224	246	282	286	214	216	264	294	170	200	272	300	110	112
10rbl.122	212	212	282	290	202	212	272	298	172	194	268	298	110	112
10rbl.131	232	252	282	286	214	218	270	274	170	170	266	298	110	110
10rbl.133	212	226	282	282	214	218	266	268	198	202	300	308	110	110
10rbl.139	226	252	290	290	216	216	292	308	192	202	286	294	110	110
10rbl.141	214	224	282	290	214	216	284	298	194	194	284	286	110	110
10rbl.144	214	228	284	284	208	216	274	294	166	194	282	282	110	110
10rbl.150	226	228	284	292	210	212	220	264	188	198	276	284	110	110
10rbl.151	226	232	286	290	208	214	290	302	168	196	292	292	110	112
10rbl.155	214	224	292	292	212	212	272	306	216	216	270	286	110	110
10rbl.156	226	252	286	286	212	214	282	298	170	194	282	296	110	110
10rbl.159	214	228	284	284	216	218	286	286	192	216	288	292	110	112
10rbl.161	212	222	282	286	218	220	302	302	174	194	260	288	110	110
10rbl.162	214	228	282	292	216	218	260	296	170	196	276	294	110	110
10rbl.166	218	220	282	286	212	216	284	288	174	224	278	288	110	112
10rbl.168	246	250	284	284	204	214	266	272	152	220	284	290	110	112
10rbl.171	216	218	282	288	214	222	256	296	168	208	288	296	110	110
10rbl.172	214	224	286	290	210	210	280	294	170	174	278	282	106	110
10rbl.179	214	224	290	290	208	222	250	292	198	212	262	298	110	110
10rbl.182	224	236	282	302	212	218	286	296	170	170	280	286	110	110
10rbl.192	228	248	282	302	208	210	272	294	178	178	272	280	110	110
10rbl.200	230	252	282	290	214	218	256	266	168	194	268	290	110	110
10rbl.203	218	222	286	286	204	208	296	306	158	198	286	290	110	110
10rbl.208	222	248	282	286	212	218	270	294	190	194	276	290	112	112
10rbl.214	214	214	292	302	208	214	298	308	178	222	284	296	110	110
10rbl.215	214	214	282	284	212	212	250	250	168	172	280	284	110	112
10rbl.216	224	230	286	286	212	214	266	298	170	170	280	286	110	110
10rbl.220	222	224	282	282	210	220	256	288	170	174	286	294	110	110
10rbl.221	214	228	282	286	216	236	264	286	174	174	290	300	110	110
10rbl.224	224	232	286	286	202	214	282	296	204	204	266	284	110	110
10rbl.225	222	226	286	292	214	214	300	300	170	170	282	282	110	110
10rbl.227	230	236	282	290	210	214	272	280	172	190	282	296	110	112
10rbl.232	214	222	282	282	212	214	286	308	166	166	276	288	110	112
10rbl.233	224	232	286	290	202	216	250	308	150	176	274	282	110	112
10rbl.238	226	228	286	290	212	216	290	292	172	176	282	288	110	110

10rbl.240	214	228	286	290	208	216	250	288	176	196	282	286	110	110
10rbl.241	224	226	282	292	210	220	270	308	172	190	276	288	110	110
10rbl.242	224	228	286	290	208	212	250	302	170	196	270	274	110	110
10rbl.244	222	224	282	282	214	216	252	272	172	196	272	274	110	110
10rbl.246	218	228	288	288	214	218	288	310	194	196	284	290	110	110
10rbl.253	214	218	290	290	218	218	266	298	170	170	294	298	110	110
10rbl.257	222	222	282	286	218	218	274	302	166	208	272	274	110	110
10rbl.258	224	224	290	290	214	214	276	302	150	150	284	292	110	110
10rbl.265	228	244	282	286	202	210	284	292	200	210	272	294	110	110
10rbl.267	224	226	290	290	212	218	282	310	180	180	264	288	110	110
10rbl.276	216	218	286	290	204	228	270	298	190	194	284	284	110	110
10rbl.278	218	220	282	290	216	220	286	286	190	216	288	292	110	112
10rbl.282	222	222	286	290	210	214	288	298	172	172	280	290	110	112
10rbl.283	222	246	282	286	212	214	272	286	172	172	272	300	110	110
10rbl.284	214	224	282	282	218	236	268	308	174	206	282	282	110	110
10rbl.288	220	222	282	282	210	220	264	290	210	216	278	288	112	112
10rbl.297	214	226	282	286	218	218	286	294	190	222	284	294	110	110
10rbl.311	224	228	292	292	216	230	298	298	150	170	284	300	110	110
10rbl.316	218	218	284	284	214	214	264	272	190	200	284	300	110	144
10rbl.321	218	224	290	290	214	230	272	296	168	170	290	300	110	110
10rbl.324	224	224	286	290	212	218	266	266	162	168	278	282	110	110
10rbl.325	224	228	286	290	214	218	280	300	166	172	272	278	110	110
10rbl.326	224	226	282	292	208	216	268	286	150	188	298	298	110	110
10rbl.327	214	226	282	290	214	222	268	282	174	208	286	294	110	110
10rbl.330	216	226	284	284	214	216	266	270	204	204	284	284	110	114
10rbl.339	224	232	290	290	214	218	300	300	192	214	272	296	110	110
10rbl.340	218	236	282	288	214	218	286	300	186	190	288	298	110	110
10rbl.348	212	244	286	292	218	230	304	304	170	170	284	284	110	112
10rbl.350	214	214	282	282	212	222	298	312	170	206	276	292	110	110
10rbl.352	212	224	282	286	214	230	284	310	150	174	286	286	110	110
10rbl.358	212	212	286	290	214	218	286	300	202	202	0	0	110	112
10rbl.360	226	226	290	290	210	218	214	270	170	178	298	300	110	110
10rbl.361	226	232	282	290	210	210	278	284	178	222	300	308	110	110
10rbl.363	212	224	284	284	202	210	250	286	170	190	276	290	110	110
10rbl.365	214	226	284	290	202	222	282	298	170	212	266	302	84	110
10rbl.366	214	224	284	284	214	218	272	294	200	202	272	304	110	110
10rbl.367	220	232	288	290	210	218	298	306	150	170	268	284	110	114

10rbl.376	214	222	286	290	210	214	268	278	194	200	272	288	110	110
10rbl.382	214	226	290	290	210	214	286	296	174	174	282	300	110	110
10rbl.387	214	224	282	288	204	230	272	276	178	200	274	284	110	114
10rbl.393	214	226	290	290	218	220	284	310	168	200	278	278	110	110
10rbl.398	214	242	292	292	214	232	252	284	178	204	266	288	110	110
10rbl.424	218	224	290	290	210	222	250	290	150	180	288	288	110	110
10rbl.425	222	222	290	290	214	214	268	272	0	0	262	274	110	114
10rbl.427	216	228	292	292	214	218	278	302	150	150	280	296	110	114
10rbl.438	214	226	286	290	214	216	266	292	194	194	282	282	110	110
10rbl.442	222	232	284	290	214	214	286	290	220	222	278	302	110	112
10rbl.444	224	244	288	290	214	216	300	304	172	172	262	286	110	110
10rbl.450	228	244	286	290	204	222	288	288	170	216	270	284	110	110
10rbl.453	222	226	284	288	212	234	290	290	150	206	274	284	110	110
10rbl.456	214	228	284	284	212	214	272	306	174	190	286	290	110	112
10rbl.477	216	224	284	284	210	212	268	284	200	210	280	280	110	110
10rbl.478	224	232	286	286	216	236	220	298	150	178	272	300	110	110
11rbl.002	214	226	286	286	210	218	286	290	174	192	290	292	110	110
11rbl.004	222	252	290	290	0	0	272	290	202	206	270	304	110	110
11rbl.011	248	252	282	288	210	214	286	286	182	192	276	288	110	110
11rbl.021	224	224	282	286	208	218	274	284	168	188	294	298	110	110
11rbl.022	214	224	282	284	210	214	288	292	192	198	286	290	110	110
11rbl.030	236	242	282	286	202	214	252	302	200	200	298	300	110	114
11rbl.033	224	250	282	290	214	216	274	296	190	202	276	296	110	110
11rbl.034	214	224	286	286	214	222	268	300	168	196	288	296	110	114
11rbl.035	222	222	290	290	214	214	270	270	170	170	286	292	110	110
11rbl.036	214	244	284	284	212	218	266	270	188	206	274	282	110	110
11rbl.040	222	222	284	290	212	220	286	292	188	202	270	276	110	110
11rbl.041	214	224	282	286	214	218	262	312	210	210	276	292	110	112
11rbl.045	214	214	282	290	214	218	262	298	200	222	272	286	110	110
11rbl.046	224	228	282	282	204	214	256	264	166	202	272	288	110	110
11rbl.049	222	256	286	290	214	236	290	304	184	210	286	290	110	110
11rbl.055	228	228	284	286	212	214	288	292	202	210	278	304	110	112
11rbl.060	222	224	282	290	218	236	254	256	196	216	290	300	110	110
11rbl.064	224	226	282	282	222	230	264	304	210	212	274	280	110	114
11rbl.067	214	222	290	290	208	216	292	298	192	198	276	294	110	110
11rbl.076	224	230	282	284	212	218	284	296	210	210	272	288	110	110
11rbl.077	222	260	284	286	214	218	254	256	174	208	272	286	110	110

11rbl.086	228	228	284	302	208	214	280	290	150	180	272	284	110	110
11rbl.087	226	226	282	286	212	218	284	298	170	210	278	288	110	110
11rbl.094	214	236	286	290	202	218	296	298	180	196	280	280	110	112
11rbl.101	226	226	286	290	214	218	272	280	170	170	286	286	110	112
11rbl.105	212	224	286	290	214	232	264	286	168	222	284	290	112	112
11rbl.115	212	226	282	284	202	210	282	286	174	184	264	298	110	110
11rbl.119	214	218	282	286	212	212	252	292	150	150	278	284	110	110
11rbl.138	218	260	282	290	212	218	278	304	170	174	272	296	110	112
11rbl.147	224	230	282	282	214	222	290	292	206	206	272	284	110	110
11rbl.151	214	232	286	292	212	212	254	294	176	208	276	290	110	110
11rbl.158	212	224	292	292	222	222	294	304	168	208	272	284	110	110
11rbl.159	214	230	286	290	204	230	272	288	154	170	290	302	110	112
11rbl.163	212	226	282	290	202	222	292	298	180	198	268	280	110	112
11rbl.164	226	236	282	282	208	218	272	290	154	154	284	286	110	110
11rbl.171	214	224	284	288	220	236	256	296	150	168	292	296	110	110
11rbl.176	226	232	282	288	216	222	260	304	170	196	270	298	110	110
11rbl.178	224	224	282	282	202	204	272	302	204	214	284	292	110	110
11rbl.182	214	224	282	284	208	222	264	290	168	200	280	286	110	110
11rbl.185	212	214	282	288	214	218	300	300	200	216	266	284	110	110
11rbl.187	242	246	284	286	212	218	272	282	178	196	286	296	110	110
11rbl.189	218	228	282	290	218	230	256	264	170	216	276	288	110	114
11rbl.194	220	232	282	302	214	220	286	302	174	180	270	306	110	112
11rbl.195	224	232	284	290	214	218	294	310	170	178	256	296	110	112
11rbl.211	224	252	282	290	212	222	284	288	150	150	276	286	110	110
11rbl.212	224	232	288	290	214	218	264	268	194	224	272	278	110	114
11rbl.213	224	226	284	290	208	212	282	282	192	192	280	290	110	110
11rbl.215	230	230	282	286	212	218	268	288	174	192	280	290	110	110
11rbl.216	218	222	282	282	202	212	288	290	150	214	280	298	110	110
11rbl.217	212	214	282	290	210	214	280	302	218	220	286	292	110	110
11rbl.223	222	222	282	286	204	214	260	282	180	204	290	294	110	110
11rbl.225	222	236	282	290	214	214	252	304	170	202	284	298	110	110
11rbl.230	224	230	282	282	210	220	272	292	174	202	268	290	110	112
11rbl.233	214	230	290	290	214	218	264	272	166	204	288	310	110	110
11rbl.238	222	228	282	290	210	214	256	286	194	194	286	296	110	112
11rbl.245	224	248	286	290	208	216	268	302	150	150	268	276	110	110
11rbl.247	222	250	282	284	208	216	260	288	178	208	298	304	110	112
11rbl.252	212	244	282	290	202	222	272	288	168	192	272	284	110	110

11rbl.261	224	224	280	282	218	230	256	282	166	200	280	280	110	112
11rbl.263	214	222	282	284	208	232	260	272	174	202	290	302	110	112
11rbl.265	214	250	284	284	204	216	280	280	200	214	280	296	110	112
11rbl.271	222	236	280	282	216	230	280	298	166	196	282	286	110	114
11rbl.282	222	228	280	282	212	214	250	286	180	198	286	290	110	110
11rbl.287	214	224	282	282	210	212	276	302	172	172	284	290	110	112
11rbl.290	224	224	292	292	226	232	254	298	170	202	280	294	110	112
11rbl.293	214	224	282	290	208	218	272	282	192	198	286	304	110	110
11rbl.299	214	228	290	292	218	218	268	298	192	198	292	296	110	110
11rbl.302	228	252	282	286	214	228	284	284	196	196	276	280	110	110
11rbl.303	222	226	282	292	204	208	272	274	150	180	272	290	110	112
11rbl.309	224	230	282	290	214	236	270	296	168	210	272	284	110	110
11rbl.310	212	220	282	286	204	218	294	296	190	202	268	294	110	110
11rbl.312	214	218	286	290	214	236	274	292	202	206	286	304	110	110
11rbl.313	214	232	286	290	218	230	286	292	168	198	288	290	110	110
11rbl.317	226	230	282	284	214	236	286	306	196	200	284	296	110	112
11rbl.318	226	248	282	290	214	214	266	300	192	196	280	298	110	110
11rbl.320	226	236	282	282	214	214	278	310	150	184	288	300	110	110
11rbl.321	212	232	282	290	208	222	258	304	166	192	272	276	110	110
11rbl.331	214	232	282	290	216	222	252	288	192	196	290	290	110	110
11rbl.338	222	222	282	292	210	218	290	298	204	214	284	296	110	112
11rbl.341	218	222	290	290	214	222	272	292	168	176	280	300	110	112
11rbl.345	214	222	284	286	202	214	288	288	196	222	288	294	110	110
11rbl.346	224	228	282	292	214	218	268	272	150	172	300	300	110	112
11rbl.352	214	216	284	286	210	214	256	298	168	178	300	300	110	110
11rbl.354	246	246	286	286	210	218	290	294	150	204	284	284	110	110
11rbl.356	212	232	280	282	214	218	288	308	188	188	270	272	110	110
11rbl.360	222	226	282	284	212	212	286	288	190	198	286	292	110	110
11rbl.361	212	226	290	290	214	218	282	286	170	170	268	304	110	110
11rbl.367	212	214	282	286	214	222	256	288	216	216	276	284	110	112
11rbl.368	218	222	284	284	212	212	284	286	150	202	280	280	110	112
11rbl.375	224	232	282	282	214	214	290	298	200	200	272	286	110	110
11rbl.376	214	224	286	286	214	214	298	304	150	214	268	288	110	112
11rbl.390	224	228	282	286	216	220	260	270	168	168	294	300	110	114
11rbl.400	212	224	282	282	214	218	252	270	150	224	276	278	110	112
11rbl.404	222	222	286	290	202	210	296	296	174	218	264	272	110	110
11rbl.406	212	222	282	290	202	218	274	304	166	192	276	284	110	112

11rbl.411	222	254	290	292	218	236	298	304	204	204	278	292	110	110
11rbl.415	216	224	282	286	216	216	286	286	174	202	268	286	110	110
11rbl.421	218	224	290	290	210	214	248	298	150	210	270	298	110	112
11rbl.422	212	226	288	288	210	214	298	308	150	186	266	286	112	114
11rbl.429	222	232	286	290	214	220	288	294	170	192	292	300	110	112
11rbl.436	224	224	282	290	218	218	254	298	170	170	290	290	110	132
11rbl.441	222	244	290	290	210	214	272	286	200	224	268	282	110	110
11rbl.442	218	248	282	290	208	212	272	288	188	188	270	284	110	110
11rbl.448	216	228	282	284	208	214	290	306	170	180	292	294	110	112
11rbl.451	224	252	280	290	210	218	248	280	168	196	268	282	110	110
11rbl.452	214	254	290	290	210	214	294	304	168	168	282	292	112	114
11rbl.454	212	214	282	290	216	218	282	292	170	202	272	290	110	110
11rbl.463	216	232	282	286	206	210	282	286	190	208	268	282	110	110
11rbl.466	218	224	286	286	210	214	284	304	154	170	294	294	110	110
11rbl.469	214	248	286	288	222	222	290	290	190	196	296	296	110	112
11rbl.482	226	236	282	290	214	218	284	286	196	196	268	282	110	112
11rbl.491	224	248	282	292	214	222	252	298	198	204	284	288	110	112
11rbl.501	222	230	282	286	218	218	286	300	204	204	280	290	110	112
11rbl.502	224	226	290	290	206	214	278	286	196	214	282	290	110	110
11rbl.507	226	244	282	284	204	212	252	300	192	210	286	292	110	110
11rbl.510	224	224	282	286	204	216	274	310	202	210	0	0	110	110
11rbl.516	214	224	286	290	214	222	280	286	172	172	282	282	110	114
11rbl.521	218	224	282	286	218	218	262	292	196	210	284	288	110	110
11rbl.522	212	216	282	286	212	222	256	298	168	206	270	294	110	112
11rbl.529	212	216	286	290	208	212	294	302	178	194	276	290	110	110

Supplemental Table 5. Estimates of allelic richness estimated from 15 microsatellite loci for razorback sucker from Lake Mohave, Arizona and Nevada, from the years 1997-2011.

Locus	1997	1998	1999	2000	2001	2002	2003	2004	2005	2006	2007	2008	2009	2010	2011
Xte1	2.00	2.00	2.00	2.00	2.00	2.00	2.00	2.00	2.00	2.00	2.00	2.00	2.00	2.00	2.00
Xte2	4.00	4.00	3.95	3.00	4.00	4.00	3.98	4.00	4.00	4.00	4.00	4.00	4.00	4.00	5.00
Xte7	19.82	20.87	19.82	14.87	19.92	17.85	17.92	14.90	17.87	17.87	13.95	15.87	16.92	17.97	19.92
Xte8	19.95	19.95	18.97	21.90	22.87	18.95	21.95	20.90	18.95	21.92	18.92	21.87	21.90	19.90	21.92
Xte10	10.00	10.00	10.00	11.97	11.00	10.97	11.98	11.98	12.00	11.95	10.98	12.00	9.98	10.98	10.97
Xte11	17.87	15.87	16.92	17.88	17.95	19.87	14.87	16.90	16.87	14.97	14.92	17.85	16.83	16.87	13.95
Xte12	12.90	11.97	13.92	10.97	12.92	10.98	13.97	11.95	10.98	11.97	10.98	12.98	13.93	9.97	13.95
Xte16	31.77	26.92	25.92	30.00	30.00	26.92	29.95	23.98	27.95	25.87	29.82	26.97	27.87	25.90	30.90
Xte17	21.87	19.95	19.92	19.97	19.95	21.95	16.97	21.90	20.97	19.95	18.90	19.97	20.90	18.95	20.97
Xte18	8.98	8.98	8.95	8.00	7.98	7.98	7.00	7.00	8.97	8.00	9.97	10.00	7.97	7.00	8.00
Xte19	17.90	15.92	15.93	15.97	16.97	14.95	15.97	14.97	15.92	15.95	17.92	17.97	16.90	14.95	15.97
Xte20	29.92	33.92	35.80	35.97	31.95	34.92	34.87	34.95	34.90	33.85	35.90	35.91	33.95	30.98	32.90
Xte22	34.92	31.98	28.90	34.87	32.95	32.87	32.97	31.95	30.95	32.90	30.92	32.97	31.95	32.87	31.90
Xte24	22.00	22.95	23.93	23.98	22.95	23.87	23.90	22.97	23.90	24.97	23.92	24.00	22.95	24.93	23.95
Xte25	4.95	3.98	3.00	4.00	3.00	3.00	4.00	3.00	3.00	4.00	3.00	3.00	3.00	5.93	3.98

Supplemental Table 6. Estimates of gene diversity estimated from 15 microsatellite loci for razorback sucker from Lake Mohave, Arizona and Nevada, from the years 1997-2011.

Locus	1997	1998	1999	2000	2001	2002	2003	2004	2005	2006	2007	2008	2009	2010	2011
Xte1	0.316	0.220	0.321	0.132	0.200	0.250	0.279	0.146	0.146	0.080	0.187	0.226	0.326	0.279	0.301
Xte2	0.171	0.150	0.104	0.148	0.112	0.172	0.171	0.112	0.178	0.171	0.193	0.157	0.192	0.226	0.165
Xte7	0.731	0.722	0.686	0.546	0.717	0.699	0.758	0.553	0.589	0.635	0.641	0.609	0.732	0.739	0.723
Xte8	0.900	0.898	0.885	0.910	0.912	0.907	0.904	0.907	0.906	0.894	0.895	0.904	0.914	0.883	0.892
Xte10	0.859	0.857	0.861	0.858	0.839	0.842	0.849	0.854	0.863	0.867	0.857	0.874	0.842	0.847	0.853
Xte11	0.882	0.869	0.876	0.865	0.871	0.877	0.865	0.864	0.858	0.866	0.866	0.888	0.870	0.855	0.866
Xte12	0.815	0.795	0.821	0.751	0.837	0.823	0.850	0.838	0.828	0.861	0.828	0.863	0.827	0.806	0.826
Xte16	0.941	0.934	0.924	0.933	0.935	0.934	0.932	0.922	0.935	0.930	0.932	0.937	0.937	0.932	0.940
Xte17	0.869	0.894	0.891	0.891	0.912	0.907	0.879	0.858	0.888	0.887	0.860	0.887	0.874	0.904	0.903
Xte18	0.800	0.792	0.797	0.844	0.817	0.825	0.792	0.828	0.835	0.777	0.817	0.841	0.786	0.795	0.772
Xte19	0.872	0.848	0.880	0.829	0.863	0.859	0.882	0.859	0.851	0.841	0.857	0.884	0.846	0.864	0.873
Xte20	0.955	0.953	0.957	0.963	0.960	0.962	0.958	0.959	0.959	0.955	0.962	0.959	0.963	0.961	0.957
Xte22	0.955	0.959	0.937	0.945	0.954	0.949	0.954	0.953	0.948	0.953	0.956	0.949	0.951	0.945	0.956
Xte24	0.944	0.935	0.939	0.944	0.939	0.927	0.931	0.940	0.941	0.945	0.945	0.952	0.948	0.943	0.942
Xte25	0.357	0.356	0.340	0.284	0.313	0.311	0.321	0.362	0.316	0.378	0.381	0.275	0.362	0.306	0.346

Supplemental Table 7. Mean *F*-statistics and their standard errors for razorback sucker from Lake Mohave, Arizona and Nevada, (obtained by jackknifing across populations) calculated for each locus, including minimum and maximum values. The total estimate was obtained by jackknifing across loci.

	F		Θ		*f*	
Locus	Mean	SE	Mean	SE	Mean	SE
Xte1	0.043	0.026	0.019	0.007	0.025	0.024
Xte2	-0.055	0.007	0.000	0.001	-0.055	0.007
Xte7	0.043	0.021	0.010	0.003	0.034	0.020
Xte8	0.038	0.011	0.002	0.001	0.037	0.011
Xte10	0.104	0.025	0.027	0.009	0.079	0.023
Xte11	0.054	0.023	0.001	0.001	0.053	0.023
Xte12	0.044	0.009	0.008	0.002	0.036	0.009
Xte16	0.030	0.009	0.001	0.001	0.028	0.009
Xte17	0.072	0.012	0.003	0.001	0.069	0.011
Xte18	0.091	0.021	0.007	0.003	0.085	0.021
Xte19	0.021	0.007	0.002	0.001	0.019	0.006
Xte20	0.081	0.010	0.001	0.000	0.080	0.010
Xte22	0.165	0.018	0.002	0.001	0.164	0.018
Xte24	0.051	0.010	0.002	0.001	0.049	0.010
Xte25	0.007	0.023	0.000	0.001	0.007	0.022
Total	0.063	0.011	0.005	0.002	0.058	0.011
